# Tuneable access to indole, indolone, and cinnoline derivatives from a common 1,4-diketone Michael acceptor

**DOI:** 10.3762/bjoc.16.144

**Published:** 2020-07-17

**Authors:** Dalel El-Marrouki, Sabrina Touchet, Abderrahmen Abdelli, Hédi M’Rabet, Mohamed Lotfi Efrit, Philippe C Gros

**Affiliations:** 1 Université de Lorraine, CNRS, L2CM, F-5400 Nancy, France; 2 Université de Tunis El Manar, SOHES-LR17ES01, Tunis, Tunisia

**Keywords:** cinnoline, 1,4-diketone, indole, indolone, *N*-heterocycle

## Abstract

A convergent strategy is reported for the construction of nitrogen-containing heterocycles from common substrates: 1,4-diketones and primary amines. Indeed, by just varying the substrates, the substituents, or the heating mode, it is possible to selectively synthesize indole, indolone (1,5,6,7-tetrahydroindol-4-one), or cinnoline (5,6,7,8-tetrahydrocinnoline) derivatives in moderate to excellent yields.

## Introduction

**Figure 1:**
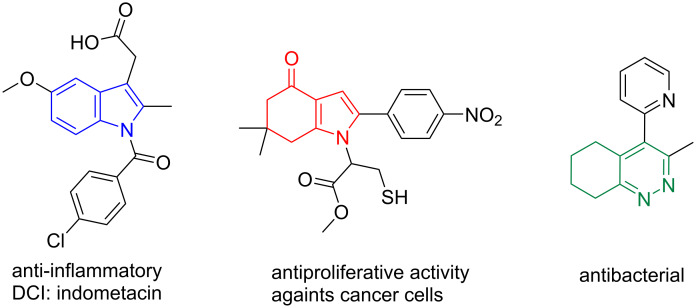
Examples of bioactive nitrogen-containing heterocycles (indole [9], indolone [10], and cinnoline [11] derivatives).

Nitrogen-containing heterocycles are widespread in plenty of molecules of interest, either in materials science, optics, electronics, or biology [1–4]. They are also very useful building blocks to create more sophisticated organic molecules. Therefore, the search for efficient methods for the synthesis of nitrogen-containing heterocycles is crucial to both organic and medicinal chemists. Among these, indole, indolone (1,5,6,7-tetrahydroindol-4-one), and cinnoline (5,6,7,8-tetrahydrocinnoline) derivatives are important classes of functionalized compounds, having biological and medicinal activities of interest (Figure 1) [5–8].

Indeed, indole ring-containing compounds have various biological and pharmacological activities and are part of many marketed drugs used as anticancer, antiemetic, antihypertensive, antidepressant, anti-inflammatory, or anti-HIV agents, among others [9]. In contrast, concerning indolone and cinnoline derivatives, there are very few marketed drugs, but many molecules are under investigations for their activities as antibacterial, antifungal, anticancer, or anti-inflammatory agents or even on the central nervous system [7,12–13].

Several routes have been reported to access these key compounds, the most developed being for the indole [14] derivatives using the Fischer indole synthesis involving sigmatropic rearrangements [15–18], nucleophilic and electrophilic cyclizations [19–25], reductive and oxidative cyclizations [26–27], and transition-metal catalysis [28–37]. There are fewer ways to access indolone derivatives, mainly based on the use of di- [12,38–39] or triketones [10,13] and enaminones [40–43] as starting materials. For the synthesis of cinnoline derivatives, aryldiazenes and aryltriazenes are substrates of choice for transition-metal-catalyzed (Rh, Pd, Cu) cross-coupling reactions, followed by intramolecular cyclizations [44–47]. Moreover, arylhydrazones and arylhydrazines/hydrazines can be used as well, respectively, as partners in [4 + 2] cyclization reactions [48–51] or by reacting mostly with carbonyl derivatives [52–55].

**Scheme 1:**
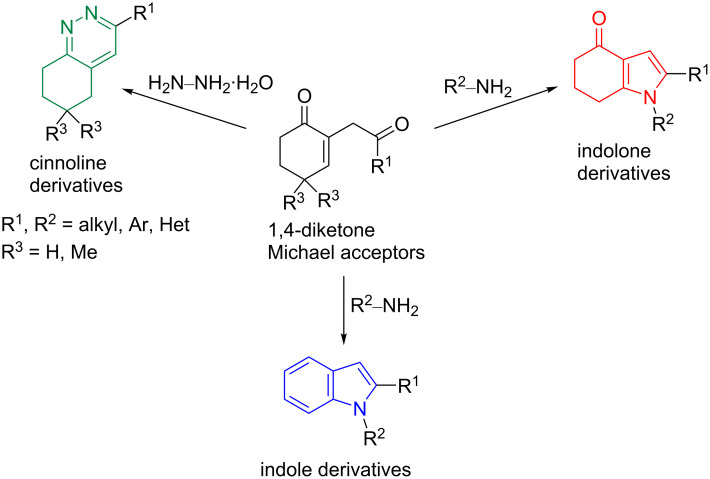
General strategy to access indole, indolone, and cinnoline derivatives from 1,4-diketones.

From the state-of-the-art, a strategy that promotes the synthesis of indole, indolone, or cinnoline derivatives from the same starting material is not yet available. To reach this goal, the Michael reaction between 1,4-diketones and primary amines seems particularly attractive because of its straightforward and metal-free properties, and because they can be performed under air. Herein, we report our investigations on this reaction, and we have shown that it can be selectively directed towards the synthesis of indole, indolone, or cinnoline derivatives by just changing the substrates, substituents, or heating mode (Scheme 1).

## Results and Discussion

**Scheme 2:**
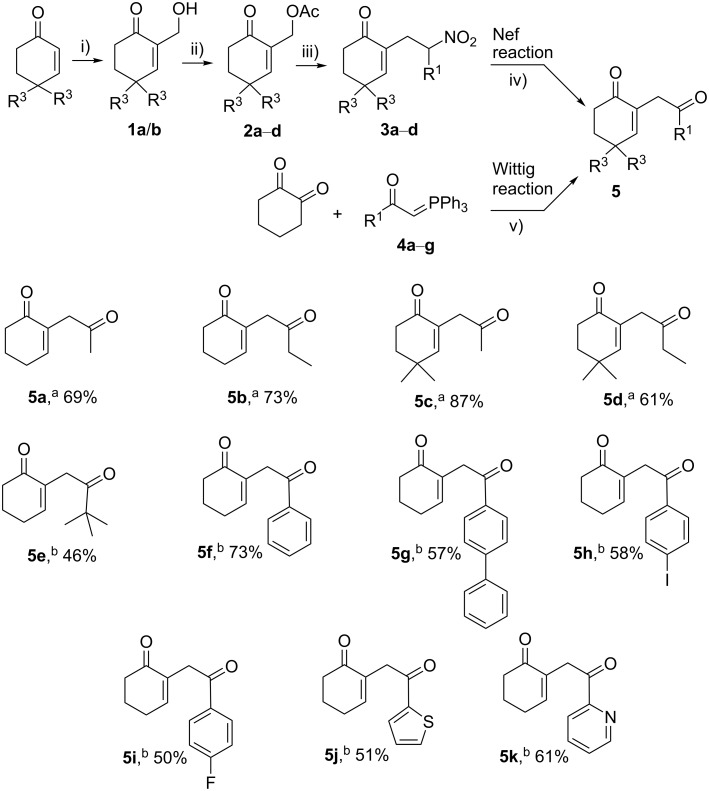
Synthesis of the 1,4-diketones **5a–k** via the Nef reaction or the Wittig reaction. i) HCHO (aq), DMAP, THF, rt, 24 h; ii) Ac_2_O, Et_3_N, DMAP, 0 °C, then 2 h, rt; iii) R^1^–CH_2_–NO_2_, Et_3_N, EtOH, reflux, 24 h; iv) EtONa, EtOH, rt, 3 h, then H_2_SO_4_, −50 °C, 1 h; v) toluene or DCM, reflux, 48 h, then rt, 2 d. ^a^Via the Nef reaction. ^b^Via the Wittig reaction.

The synthesis of the target compounds required the prior preparation of a panel of variously substituted 1,4-diketones **5**. The 1,4-diketones **5** have been prepared either by a Nef reaction [56] from the corresponding nitroenone **3** or a Wittig reaction [57] from 1,2-cyclohexanedione and the corresponding ylide **4** (Scheme 2).

The nitrenones **3a**–**d** were obtained in three steps from the appropriate commercially available cyclohexenones (Scheme 2). First, a Baylis–Hillman reaction between cyclohexanone and formaldehyde led to the formation of the corresponding Baylis–Hillman alcohols **1a**/**b** in good yield [58], followed by a DMAP-catalyzed acetylation of these alcohols, which gave the corresponding acetates **2a**–**d** [59]. The nitrenones **3a**–**d** were finally obtained in an acceptable yield by reacting the acetate derivatives with the appropriate nitroalkanes [60]. The next step was the transformation of the nitro group of **3a**–**d** via the Nef reaction, using sodium ethoxide in ethanol, followed by the hydrolysis with concentrated sulfuric acid at a low temperature [56], leading to the corresponding new γ-diketones **5a**–**d** in 61–87% yield (Scheme 2).

Despite the efficiency of the Nef reaction, the diversity at the R^1^ position via this synthetic route remains limited in terms of chemical diversity as it depends on the availability of the corresponding nitro derivative. It was thus decided to move on to the Wittig reaction [57], offering a much more straightforward and efficient route to a panel of new diketones, **5e**–**k**, from 1,2-cyclohexanedione and the corresponding Wittig ylides **4a**–**g** (readily accessible from the corresponding halogenated derivatives, Scheme 2). Indeed, the Wittig reaction leads exclusively to the isomerized products **5**.

**Scheme 3:**
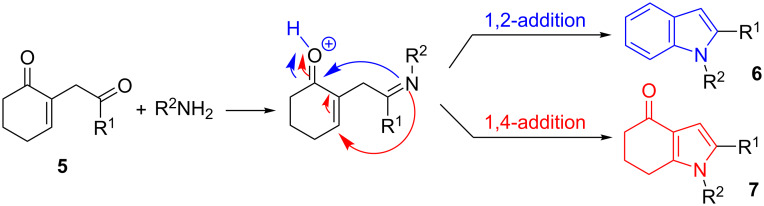
Mechanism of the formation of indole and indolone derivatives.

With the 1,4-diketones **5** in hand, we first investigated the synthesis of indole and indolone derivatives. The reaction mechanism shown in Scheme 3 involves the formation of an imine upon the reaction of the primary amine with the most reactive carbonyl moiety (nonconjugated and exocyclic carbonyl function). Then, depending on the reaction conditions, the imine can react following a 1,2- or 1,4-addition process, leading respectively to an indole **6** (after dehydration and aromatization) or an indolone **7**. The reaction was first investigated by mixing the diketone **5b** as the Michael acceptor and benzylamine under various conditions (Table 1).

**Table 1:** Effects of solvent and heating mode on the **6b**:**7b** ratio.

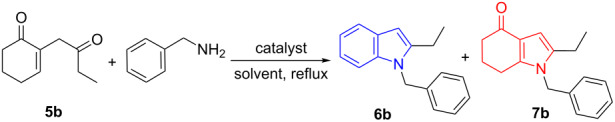						

entry^a^	catalyst	solvent	*t* (h)	**6b** (%)^b^	**7b** (%)^b^	**6b**:**7b**

1	TfOH (3%)	toluene	16 h	–	–	–
2	AgOTf (3%)	toluene	16 h	–	–	–
3	TFA (3%)	toluene	16 h	–	–	–
4	*p*TsOH (3%)	toluene	16 h	–	–	–
5	AcOH (3%)	toluene	16 h	47	10	83:17
6	–	toluene	16 h	33	8	80:20
7	–	CH_2_Cl_2_	16 h	–	–	–
8	–	THF	16 h	–	–	–
9	–	ethanol	16 h	13	16	45:55
10	–	propanol	16 h	6	31	16:84
11	–	butanol	16 h	10	43	19:81
12	–	pentanol	16 h	7	28	20:80
13^c^	–	butanol	MWI 3 h	–	60	0:100

^a^Reaction conditions: **5b** (0.54 mmol), primary amine (0.81 mmol), 4 mL solvent, and catalyst (0.02 mmol) unless otherwise specified (in column 2, the catalyst percentage corresponds to 0.02 mmol). ^b^Isolated yield. ^c^13 mL of butanol, MWI 100 °C.

**Scheme 4:**
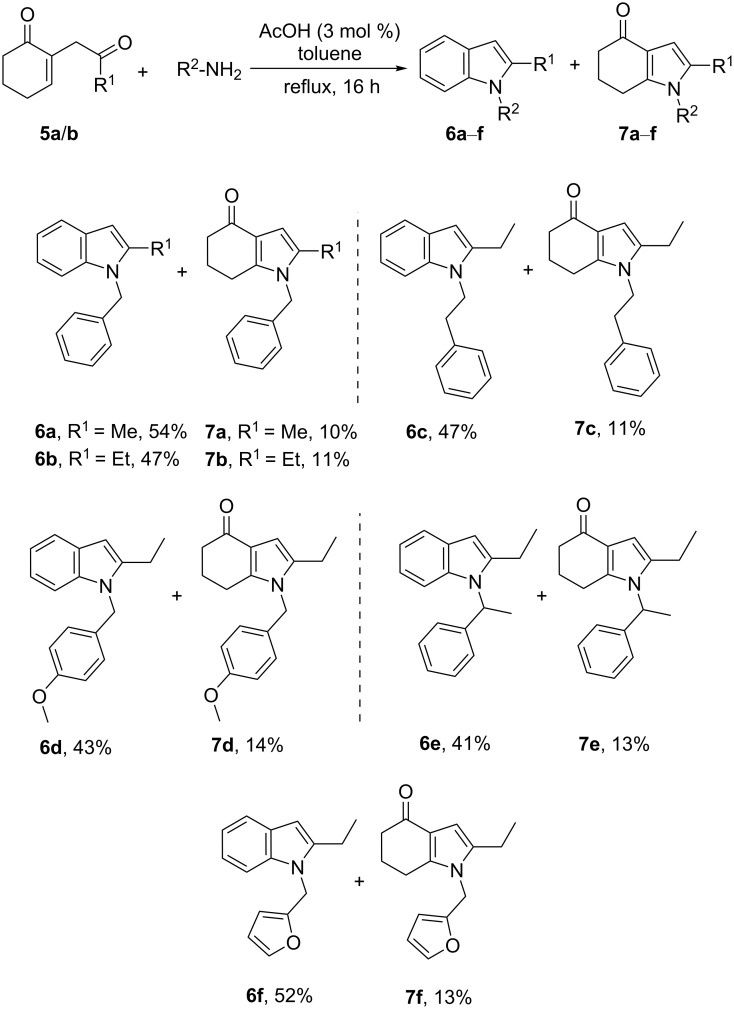
Synthesis of the indoles **6a**–**f** and the corresponding side product indolones **7a**–**f**.

We first investigated the reactivity in the presence of a set of catalysts with different acidities (Table 1, entries 1–5). Among them, only acetic acid afforded reaction products, while the others only produced complex mixtures of degradation products. Under the conditions of entry 5, the indole **6b** was isolated in 47%, and the indolone **7b** was also formed concomitantly in 10% yield. The experiments in Table 1, entries 6–11 were next performed with the aim to favor the 1,4-addition process, and thus to form the indolone **7b**. Removing the acid catalyst from the reaction mixture (Table 1, entry 6) did not affect the **6b**:**7b** ratio obtained in Table 1, entry 5. While in aprotic solvents, other than toluene (Table 1, entries 7 and 8), the reaction produced complex mixtures of degradation products, and using alcohols had a notable impact on the reaction contents. Indeed, going from ethanol to propanol, and thus increasing the refluxing temperature, led to the indolone **7b** as the main product, with the best yield being obtained in butanol (43%, Table 1, entry 11), however, with the formation of **6b** occurring only in 10% yield. Switching to microwave irradiation formed exclusively **7b** in 60% yield after 3 h (Table 1, entry 13). Note, that to check the effect of a shorter reaction time on the reaction outcome, we reduced the time to 3 h also under the classical refluxing conditions from Table 1, entry 11 and obtained a partial conversion of the starting diketone **5b**. Despite the side formation of the indolone **7b** in Table 1, entry 5, these conditions were applied to several amines, producing the corresponding substituted indoles **6a** and **6c**–**f** in 41–54% yield (Scheme 4). The yield of the indolones **7a** and **7c**–**f** was found almost constant (10–14%) with all amines involved. It is worthy of note that the two compounds were easily separated using usual chromatographic techniques.

**Scheme 5:**
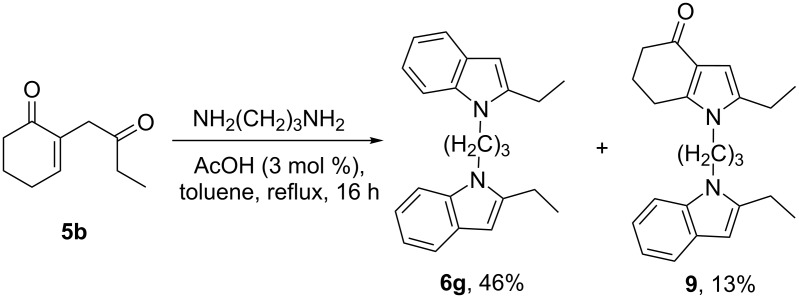
Reaction of **5b** with a diamine.

The reaction was also applied to a diamine (Scheme 5). When 1,3-diaminopropane was used, the bisindole **6g** was isolated in 46% yield. Interestingly, the mixed indolone/indole compound **9** was also obtained as a side product. However, no traces of the bisindolone derivative were detected.

**Scheme 6:**
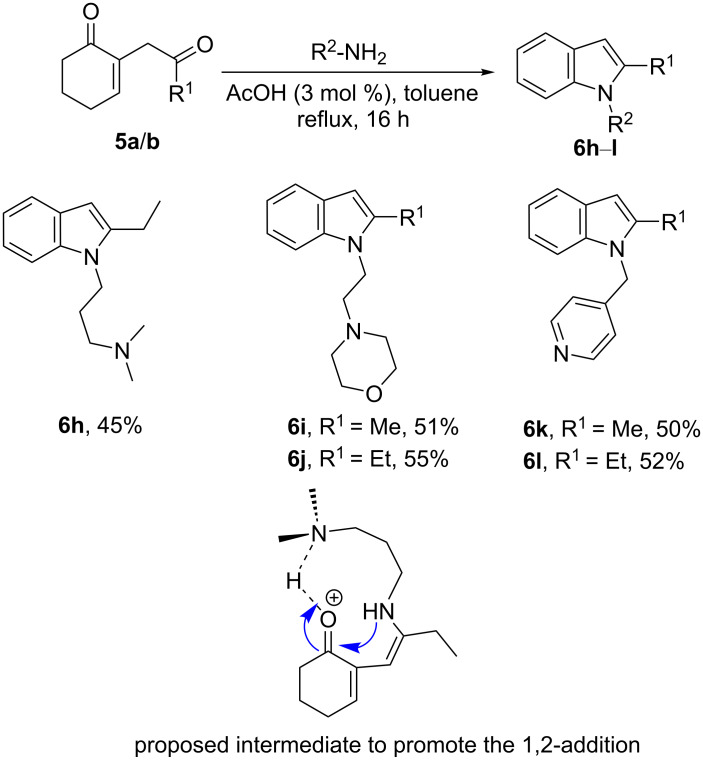
Synthesis of the indoles **6h**–**l**.

We then succeeded in directing the reaction exclusively towards indole formation by reacting the diketone with appropriate substrates, combining a primary amine with a tertiary amine or a pyridine separated by several spacer arms (Scheme 6).

Under these conditions, the functional indoles **6h**–**l** were obtained exclusively in 45–55% yield. We assume that the tertiary amine would interact with the protonated intermediate, and thus promoting the 1,2-addition (Scheme 6). However, for the pyridine substituent (compounds **6k** and **6l**), another intermediate may be involved as the shape of this diamine does not allow enough flexibility to achieve the conformation of the proposed intermediate.

**Scheme 7:**
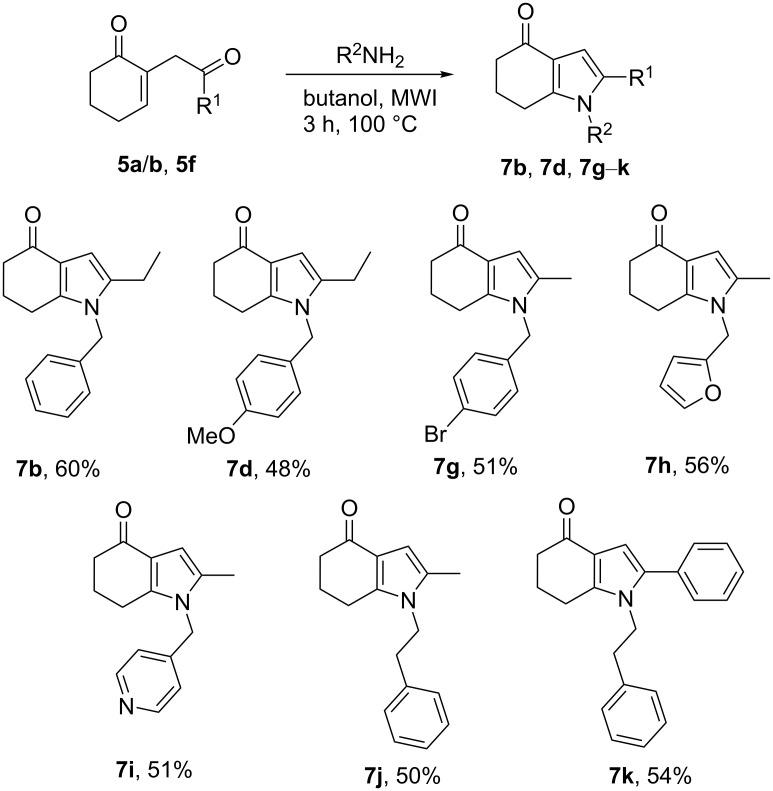
Synthesis of the indolone derivatives **7b**, **7d**, and **7g**–**k**.

We next examined the preparation of a set of indolones under the microwave conditions determined in Table 1, entry 13. These conditions were applied to several amines, producing exclusively the corresponding substituted indolones **7d** and **7g**–**k** in 48–56% yield (Scheme 7).

Here again, the amount of indolone was found to be almost constant, whatever the amine involved was, suggesting that the reaction is not dependent on the nature of the amine. It is worth to notice that, for a substrate combining a primary amine with a pyridine separated by a spacer arm, only the indolone derivative is obtained in those conditions as well (compound **7i**, Scheme 7). Based on these results, we found it important to check whether the indole **6b** resulted from a 1,2-addition and not from a degradation of the indolone **7b**. For this purpose, the indolone **7b** was refluxed overnight with acetic acid in toluene, under these conditions producing mainly the indole (Table 1, entry 5). The indolone **7b** was found unchanged, with no trace of the indole **6b** being detected (see Supporting Information File 1, chapter I), indicating that the indole was formed intramolecularly by a 1,2-addition of the intermediately formed imine to the Michael acceptor (Scheme 3).

We then investigated the synthesis of cinnoline derivatives by mixing the diketone **5a** and hydrazine monohydrate under various conditions (Table 2). We first investigated the reactivity in ethanol, as a protic solvent, at room temperature (Table 2, entry 1). Under these conditions, the expected cinnoline **8a** was obtained in a low yield of 20% that could be increased up to 40% upon refluxing the mixture (Table 2, entry 2). Switching to toluene (Table 2, entry 3) did not improve the reaction outcome. However, the addition of a catalytic amount of acetic acid in refluxing ethanol while shortening the reaction time dramatically increased the yield of **8a** up to 82% (Table 2, entry 4).

**Table 2:** Optimization of the reaction conditions for the synthesis of the cinnolines **8**.

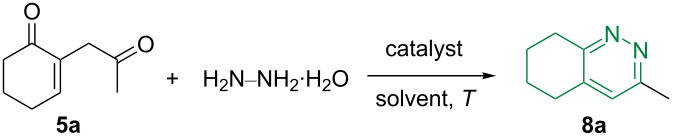					

entry^a^	catalyst	solvent	*T* (°C)	*t* (h)	**8a** (%)^b^

1	–	EtOH	rt	48	20
2	–	EtOH	reflux	48	40
3	–	toluene	reflux	48	35
4	AcOH (3 mol %)	EtOH	reflux	16	82

^a^Reaction conditions: **5a** (1 mmol), hydrazine monohydrate (1.5 mmol), solvent (6 mL), catalyst (0.03 mmol), unless otherwise specified. The reported catalyst percentage exactly corresponds to 0.03 mmol. ^b^Isolated yield.

**Scheme 8:**
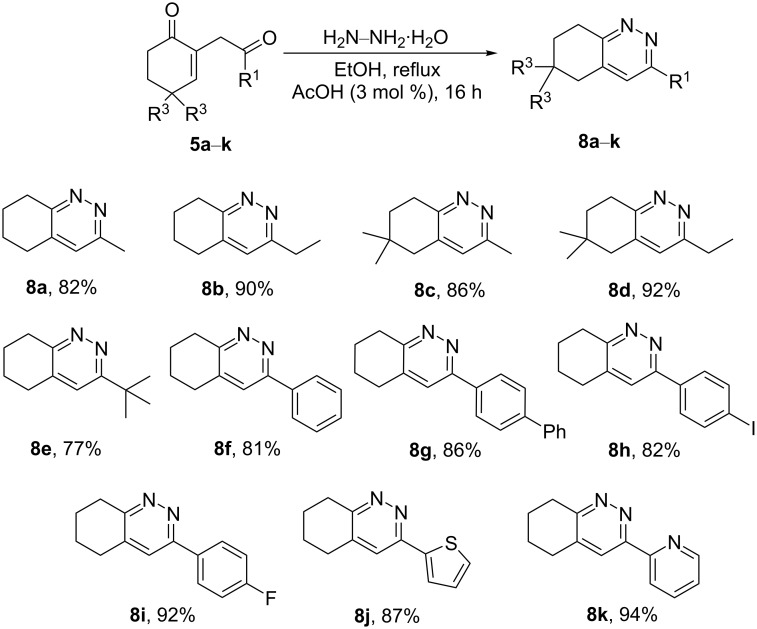
Synthesis of the cinnoline derivatives **8a**–**k**.

These optimized conditions were then applied to the previously synthesized 1,4-diketones **5a**–**k** (Scheme 8). As a general observation, the reaction was found to be efficient for producing the expected cinnoline derivatives **8a**–**k** in good to excellent yield (77–94%) and tolerated alkyl, aromatic, and heteroaromatic groups as R^1^.

**Scheme 9:**
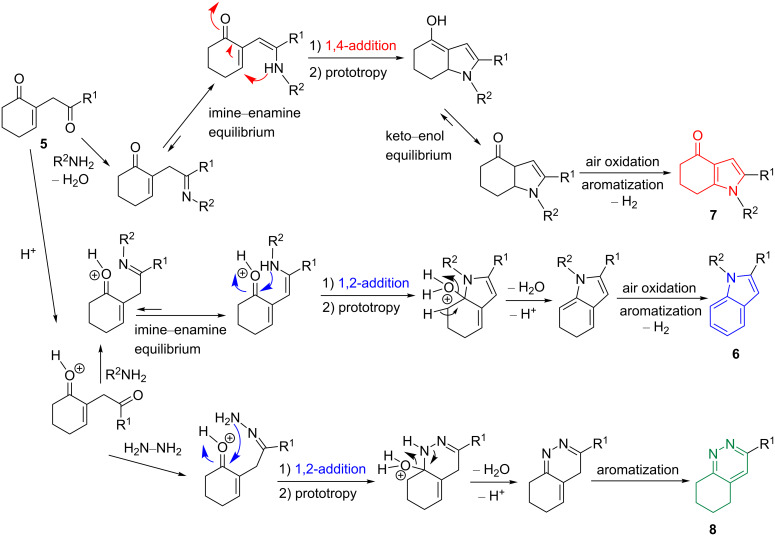
Proposed mechanism for the preparation of the compounds **6**, **7**, and **8**.

The success of our convergent strategy here can be explained through the mechanism suggested in Scheme 9. The synthetic pathway leading to the formation of the indolone **7** starts with an imine formation between the secondary amine and the nonconjugated carbonyl from the 1,4-diketone. After an imine–enamine equilibrium, an intramolecular 1,4-addition to the Michael acceptor part of the molecule occurs, followed by a prototropy, leading to an intermediate enol that, after a keto–enol equilibrium and aromatization, gives the indolone **7**. For the indole **6** and the cinnoline **8**, the synthesis starts with the protonation of the oxygen atom of the conjugated carbonyl group of the 1,4-diketone, followed by an imine formation between the secondary amine and the nonconjugated carbonyl unit. Next, an intramolecular 1,2-addition to the Michael acceptor part of the molecule, previously activated by acid catalysis, takes place (after an imine–enamine equilibrium for the indole pathway), followed by a prototropy, the release of a water molecule, the recovery of the proton catalyst, and atmospheric oxygen aromatization, leading to the indole **6** or the cinnoline **8**.

## Conclusion

In summary, we have successfully developed a straightforward and metal-free strategy for the synthesis of nitrogen-containing heterocyclic moieties of biological interest; indoles **6**, indolones **7**, and cinnolines **8**, starting from common substrates 1,4-diketones **5** and primary amines. The protocols developed here used mild conditions, were functional-group tolerant, transition-metal-free, proceeded in moderate to good yield, and could therefore easily be used in medicinal chemistry projects for the rapid access to a wide range of variously substituted compounds for structure–activity relationship studies. The biological activity of the molecules is currently being studied.

## Supporting Information

File 1 Experimental procedures, characterization data, and copies of the spectra of all compounds.
