# Supplementary material for: Tuneable access to indole, indolone, and cinnoline derivatives from a common 1,4-diketone Michael acceptor
Source: Beilstein J Org Chem. 2020 Jul 17;16:1722–31. doi: 10.3762/bjoc.16.144 (PMC7372239; doi:10.3762/bjoc.16.144)
Supplement: File 1 — Experimental procedures, characterization data, and copies of the spectra of all compounds. [file Beilstein_J_Org_Chem-16-1722-s001.pdf]

BEILSTEIN JOURNAL OF ORGANIC CHEMISTRY

Supporting Information

for

Tuneable access to indole, indolone, and cinnoline derivatives from a common 1,4-diketone Michael acceptor

Dalel El-Marrouki, Sabrina Touchet, Abderrahmen Abdelli, Hédi M'Rabet,
Mohamed Lotfi Efrit and Philippe C. Gros

Beilstein J. Org. Chem. **2020,** *16,* 1722–1731. doi:10.3762/bjoc.16.144

Experimental procedures, characterization data, and copies of the spectra of all compounds

License and Terms: This is a supporting information file under the terms of the Creative Commons Attribution License (http://creativecommons.org/licenses/by/4.0). Please note that the reuse, redistribution and reproduction in particular requires that the authors and source are credited.
The license is subject to the *Beilstein Journal of Organic Chemistry* terms and conditions: (https://www.beilstein-journals.org/bjoc)

Contents

I. GC–MS study and related spectra S2

II. General experimental methods S5

III. Generals procedures S5

IV. Characterization data S8

V. NMR spectra S37

VI. References S95

I. GC–MS study and related spectra

We found it important to make sure that the indole **6b** actually resulted from a 1,2-addition and not from a degradation of the indolone **7b**. For this purpose, the indolone **7b** was refluxed overnight (16 h) with 3 mol % of acetic acid in toluene as in Table 1, entry 5 (Main Manuscript, conditions to produce mainly the indole), and the reaction was followed by GC–MS.

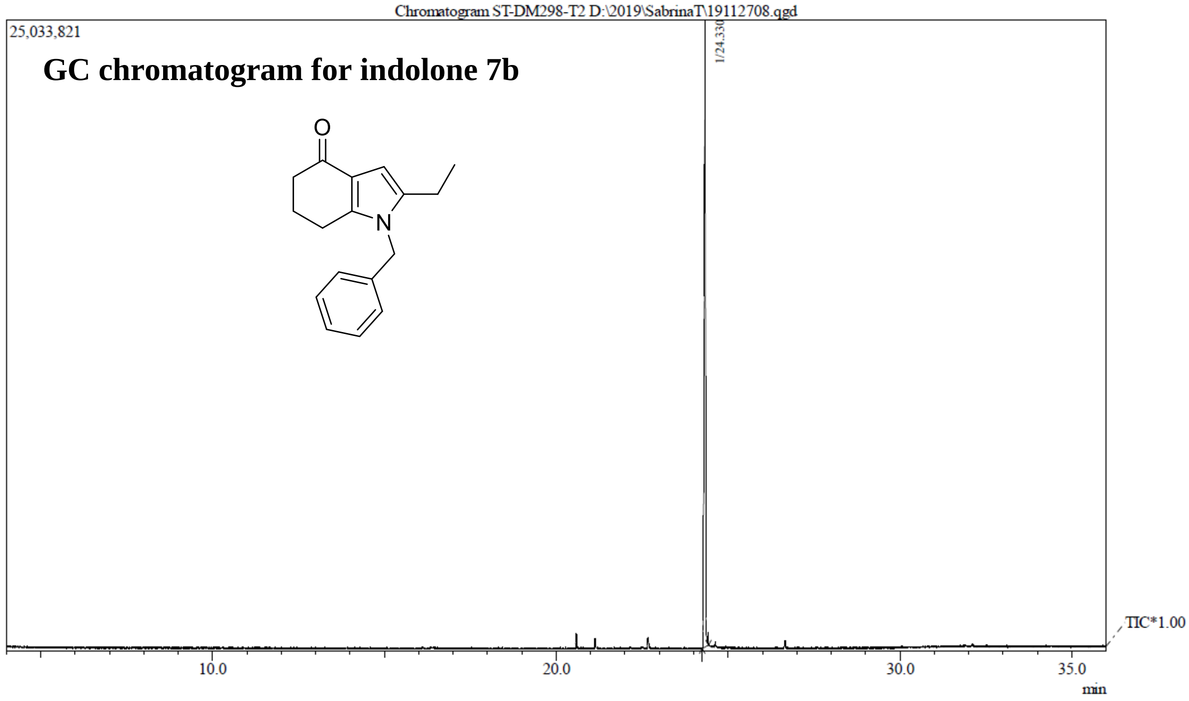

MS spectrum for the indolone **7b** peak at t = 24.33 min, M = 253.

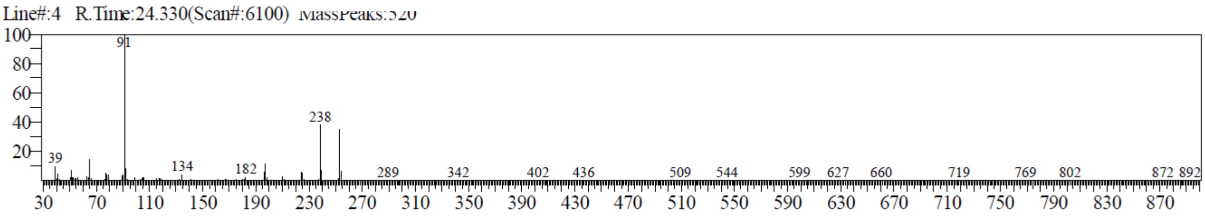

►**GC chromatogram after 16 h reaction time:**

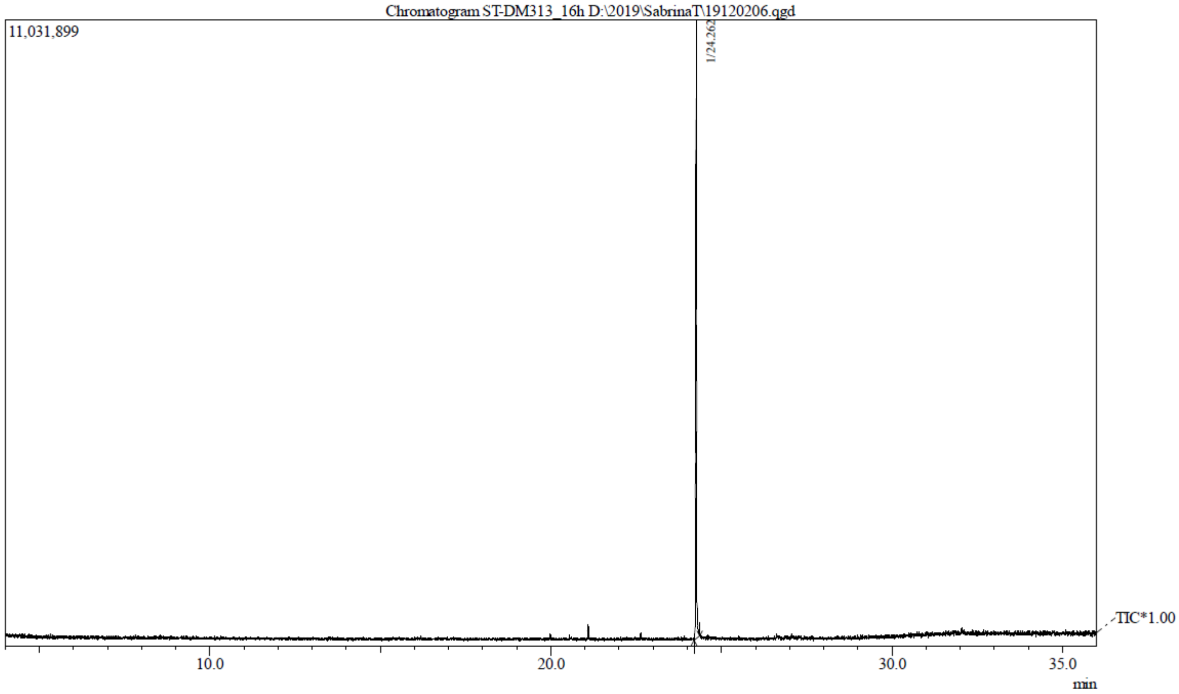

MS spectrum for the peak at t = 24.26 min.

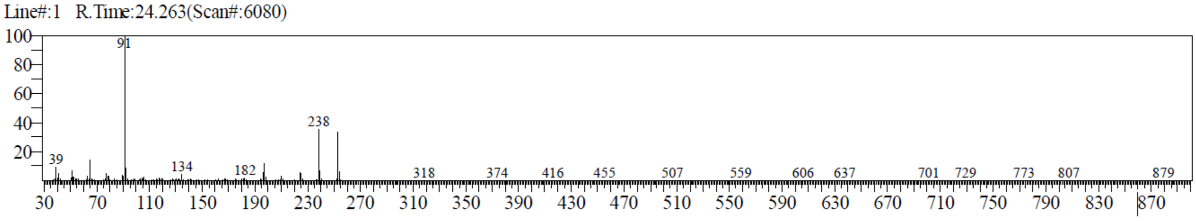

➔After 16 h reaction time, only the indolone **7b** was detected. No traces of the indole **6b**. We carried on the reaction up to 24 h to check.

► GC chromatogram after 24 h reaction time:

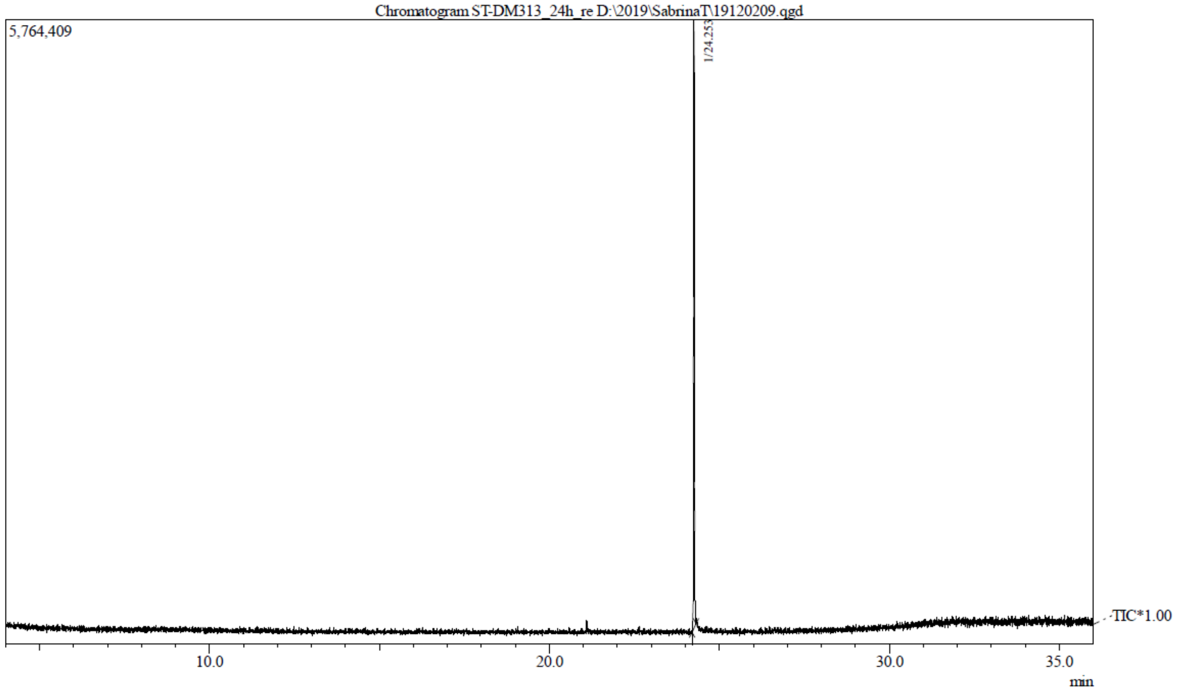

MS spectrum for the peak at *t* = 24.25 min.

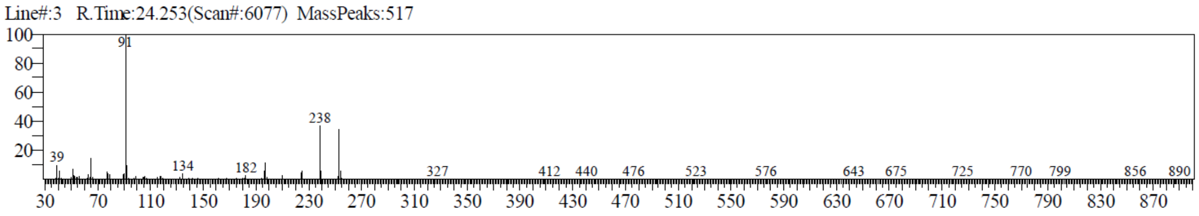

➔After 24 h reaction time, only indolone **7b** is detected. No traces of indole **6b**.

So under these conditions, the indolone **7b** remained unchanged, with no trace of the indole **6b** detected, indicating that the indole was formed intramolecularly by an 1,2-addition of the intermediately formed imine to the Michael acceptor (Schema 5 from the Main Manuscript).

II. General experimental methods

All chemicals were used as received otherwise notice.

All the NMR spectra were recorded on a Bruker Advance III 400, 300, or 200 wide bore. ^{1}H and ^{13}C NMR spectra were recorded, respectively, at 400 MHz and 101 MHz or at 300 MHz and 76 MHz or at 200 MHz and 50 MHz. ^{19}F NMR spectra were recorded at 376 MHz. ^{31}P NMR spectra were recorded at 162 MHz. ^{1}H and ^{13}C chemical shifts were reported in ppm from the residual solvent peak. Data are presented as follows: chemical shift (ppm), multiplicity (s = singlet, d = doublet, t = triplet, td = triplet of doublet, q = quadruplet, p = pentet, dd = doublet of doublets, ddd = doublet of doublets of doublets, m = multiplet), coupling constant *J* (Hz), and integral.

High-resolution mass spectra (HMRS) were recorded on a Bruker micrOTOF-Q spectrometer.

Gas chromatography–mass spectrometry (GC–MS) spectra were recorded on a Shimadzu QP2010.

Chromatography purifications were performed on a Fluka 60 silica column (0.063–0.2 mm) or a Grace Reveleris^{TM} with Reveleris^{TM} flash cartridges silica 40 µM.

Analytical thin-layer chromatography was performed on Merck Silica Gel 60 F254 plates.

The irradiation with a microwave was carried out in the cavity of a Discover system from CEM.

III. Generals procedures

a. General procedure A for the preparation of the hydroxyalkylcyclohex-2-en-1-ones 1a/b

DMAP (15 mmol, 0.20 equiv) was added to a solution of cyclohex-2-en-1-one (75 mmol, 1 equiv) or 4,4-dimethylcyclohex-2-en-1-one (75 mmol, 1 equiv) and aq HCHO (75 mmol, 1 equiv) in 15 mL of THF. The reaction mixture was stirred for 24 h at room temperature, followed by the addition of a solution of HCl (1 N, 50 mL). The organic phase was collected, and the aqueous phase was extracted 3 times with CH_2Cl_2 (3 × 30 mL). The organic phases

were combined and dried over Na_2SO_4. The solvent was evaporated, and the crude product was purified by silica gel chromatography diethyl ether/petroleum ether, 75:25. **1a** and **1b** were prepared according to literature methods [1].

b. General procedure B for the preparation of the acetyloxalkylcyclohex-2-en-1-ones 2a/b

DMAP (5 mmol, 0.12 equiv) was added to a solution of 2-hydroxymethylcyclohex-2-en-1-one (**1a**, 40 mmol, 1 equiv) or 2-(hydroxymethyl)-4,4-dimethylcyclohex-2-en-1-one (**1b**, 40 mmol, 1 equiv), acetic anhydride (40 mmol, 1 equiv), and Et_3N (40 mmol, 1 equiv) at 0 °C. After 10 min, the reaction mixture was stirred for 2 h at room temperature, followed by the addition of a solution of HCl (1 N, 50 mL). The organic phase was collected, and the aqueous phase was extracted 3 times with CH_2Cl_2 (3 × 30 mL). The organic phases were combined and dried over Na_2SO_4. The solvent was evaporated, and the crude product was purified by silica gel chromatography using diethyl ether/petroleum ether, 75:25. **2a** and **2b** were prepared according to literature methods [2].

c. General procedure C for the preparation of the nitroalkylcyclohex-2-en-1-ones 3a–d

To a solution of 2-(acetyloxymethyl)cyclohex-2-en-1-one (**2a**, 10 mmol, 1 equiv) or (2-(acetyloxymethyl)-4,4-dimethylcyclohex-2-en-1-one (**2b**, 10 mmol, 1 equiv) in 15 mL EtOH, were added nitroalkane (12 mmol, 1.2 equiv) and Et_3N (12 mmol, 1.2 equiv). The reaction mixture was heated under reflux and stirred for 24 h then dried over Na_2SO_4. The solvent was evaporated and the crude product was purified by silica gel chromatography diethyl ether/petroleum ether 50:50. **3a** and **3b** were prepared according to literature methods [2,3].

d. General procedure D for the 1,4-diketones 5a–d from a Nef reaction

To a solution of sodium (15.51 mmol, 1 equiv) in anhydrous ethanol (15 mL) were added 2-(2-nitroalkyl)cyclohex-2-en-1-one (**3**, 5.17 mmol, 0.33 equiv) dissolved in 10 mL of absolute ethanol. The reaction mixture was stirred for 3 h at room temperature. Then, 3 mL of H_2SO_4 dissolved in 10 mL of absolute ethanol was introduced at −50 °C. After 1 h, 10 mL of water were added at −50 °C and the reaction mixture was warmed up room temperature. After the completion of the reaction, the solvent was evaporated under reduced pressure and the reaction mixture was extracted three times with 50 mL of dichloromethane. The combined

organic phases were washed with a solution of NaOH (1%) and then dried with Na_2SO_4 or $MgSO_4$, and the solvent was evaporated under reduced pressure. The crude material was purified by silica gel column chromatography or flash chromatography to afford the desired product [4].

e. General procedure E for the preparation of the ylides 4a–g

A solution of PPh_3 (6.4 mmol, 1.01 equiv) and a bromide derivative (6.3 mmol, 1 equiv) in 13 mL THF was heated under reflux and stirred for 4 hours. Then, the reaction mixture was cooled to room temperature and the crude phosphonium bromide was filtered and washed with THF (3 × 20 mL). Then, 25 mL of CH_2Cl_2 and aqueous NaOH (20 wt %, 25 mL) were added, and the mixture was stirred for 10 minutes. The organic phase was separated, and the aqueous phase was extracted with CH_2Cl_2 (3 × 10 mL). The combined organic phases were dried over anhydrous $MgSO_4$, filtered, and concentrated under reduced pressure to give the corresponding phosphonium ylide [5].

f. General procedure F for the 1,4-diketones 5e–k from a Wittig reaction

To a solution of compound **3** (1 equiv) in CH_2Cl_2 or toluene (0.20 mol/L) was added cyclohexane-1,2-dione (1.05 equiv). The reaction mixture was heated under reflux and stirred for 48 hours and 2 days at room temperature. After the completion of the reaction, the solvent was evaporated under reduced pressure, and the crude material was purified by flash chromatography to afford the desired product [6].

g. General procedure G for the preparation of the indoles 6a–l

To a solution of the 1,4-diketone **5** (0.54 mmol, 1 equiv) in 4 mL toluene were added acetic acid (3 mol %, 1,7 µL) and the primary amine (0.81 mmol, 1.5 equiv). The reaction mixture was heated under reflux and stirred for 16 h. After the completion of the reaction, the reaction was dried over $MgSO_4$, and the solvent was evaporated under reduced pressure. Then, the crude material was purified by flash chromatography using a silica cartridge to afford the desired product.

h. General procedure H for the preparation of the indolone derivatives (1,5,6,7-tetrahydroindol-4-ones) 7b, 7d, and 7g–k

To a solution of the 1,4-diketone **5** (1 equiv) in butanol (*c* = 0.1 M) was added the primary amine (1.5 equiv). The reaction mixture was stirred and heated inside a microwave cavity at 100 °C for 3 h. After 3 hours, the reaction mixture was cooled and dried over $MgSO_4$, and the solvent was evaporated under reduced pressure. Then, the crude material was purified by flash chromatography using a silica cartridge to afford the desired product.

i. General procedure I for the preparation of the cinnoline derivatives (5,6,7,8-tetrahydrocinnolines) 8a–k

To a solution of the 1,4-diketone **5** (1 mmol, 1 equiv) in 6 mL of absolute ethanol were added hydrazine monohydrate (1.5 mmol, 1.5 equiv, 75 mg) and 3 mol % (1.7 μL) acetic acid (AcOH). The reaction mixture was heated under reflux and stirred for 16 hours. After the completion of the reaction, the reaction was dried with $MgSO_4$, and the solvent was evaporated under reduced pressure. Then, the crude material was purified by flash chromatography to afford the desired product.

IV. Characterization data

a. Hydroxyalkylcyclohex-2-en-1-ones 1a/b

- ***2-(Hydroxymethyl)cyclohex-2-en-1-one (1a)*** *[1]*

Compound **1a** was prepared according to the general procedure **A** using cyclohex-2-en-1-one (75 mmol, 7.2 mL), DMAP (15 mmol, 1.83 g) and $(HCHO)_{aq}$ (75 mmol, 15 mL) in 15 mL THF. The title compound was obtained after silica gel chromatography as a yellow oil. The spectral data are in good agreement with previous reports [1]. Yield = 80% (7.5 g). Chromatography conditions: diethyl ether/petroleum ether 50/50. R_f = 0.60 (diethyl ether/petroleum ether 75/25).

^{1}H NMR (300 MHz, $CDCl_3$) δ = 1.94-2.02 (m, 2H), 2.34-2.52 (m, 4H), 4.21 (s, 2H), 6.90 (t, *J* = 4.1 Hz, 1H).

- ***2-(Hydroxymethyl)-4,4-dimethylcyclohex-2-en-1-one (1b)*** *[7]*

Compound **1b** was prepared according to the general procedure **A** using 4,4-dimethylcyclohex-2-en-1-one (20 mmol, 2.5 g), DMAP (4 mmol, 0.48 g) and $(HCHO)_{aq}$ (20

mmol, 4 mL) in 4 mL THF. The title compound was obtained after silica gel chromatography as a yellow oil. Yield = 82% (2.5 g). The spectral data are in good agreement with previous reports [7]. Yield = 80% (7.5 g). Chromatography conditions: diethyl ether/petroleum ether 50/50. R_f = 0.60 (diethyl ether/petroleum ether 75/25).

^{1}H NMR (400 MHz, $CDCl_3$) δ = 1.12 (s, 6H), 1.81 (t, *J* = 6.9 Hz, 2H), 2.43 (t, *J* = 6.8 Hz, 2H), 4.16 (s, 2H), 6.57 (s, 1H).

^{13}C NMR (101 MHz, $CDCl_3$) δ = 200.4 (C), 155.8 (CH), 135.2 (C), 61.4 (CH_2), 35.9 (CH_2), 34.6 (CH_2), 32.8 (C), 27.8 (2 x CH_3).

b. Acetyloxalkylcyclohex-2-en-1-ones 2a/b

- ***2-(Acetyloxymethyl)cyclohex-2-en-1-one (2a)*** *[2]*

Compound **2a** was prepared according to the general procedure **B** using 2-(hydroxymethyl)cyclohex-2-en-1-one **1a** (40 mmol, 5.04 g), acetic anhydride (40 mmol, 4.08 g) and Et_3N (40 mmol, 4.04 g). The title compound was obtained after silica gel chromatography as a yellow oil. The spectral data are in good agreement with previous reports [2]. Yield = 80% (5.37 g). Chromatography conditions: diethyl ether/petroleum ether 50/50. R_f = 0.40 (diethyl ether/petroleum ether 50/50).

^{1}H NMR (300 MHz, $CDCl_3$) δ = 1.96 (s, 3H), 2.00-2.16 (m, 2H), 2.17-2.50 (m, 4H), 4.22 (s, 2H), 6.96 (t, *J* = 4.1 Hz, 1H).

- ***2-(Acetyloxymethyl)-4,4-dimethylcyclohex-2-en-1-one (2b)*** *[8]*

Compound **2b** was prepared according to the general procedure **B** using 2-(hydroxymethyl)-4,4-dimethylcyclohex-2-en-1-one **1b** (20 mmol, 3.04 g), acetic anhydride (20 mmol, 2.04 g) and Et_3N (20 mmol, 2.02 g). Yield = 82% (3.2 g). Chromatography conditions: diethyl ether/petroleum ether 50/50. R_f = 0.40 (diethyl ether/petroleum ether 50/50). Although this compound was reported in the literature it was not fully characterized and we completed its characterization.

^{1}H NMR (400 MHz, $CDCl_3$) δ = 1.16 (s, 6H), 1.82-1.87 (m, 2H), 2.05 (s, 3H), 2.43-2.49 (m, 2H), 4.67 (d, *J* = 1.2 Hz, 2H), 6.63 (s, 1H).

^{13}C NMR (101 MHz, $CDCl_3$) δ = 21.1 (CH_3), 27.9 (2 x CH_3), 33.1 (C), 34.6 (CH_2), 36.0 (CH_2), 61.5 (CH_2), 131.5 (C), 157.5 (CH), 170.7 (C), 197.9 (C).

HRMS (ESI, m/z) calcd for $C_{11}H_{17}O_3$ $[M+H]^+$ = 197.1172 found 197.1186 and for $C_{11}H_{16}NaO_3$ $[M+Na]^+$ = 219.0992 found 219.1038.

c. Nitroalkylcyclohex-2-en-1-ones 3b–d

- ***2-(2-Nitropropyl)cyclohex-2-en-1-one (3a)*** *[2]*

Compound **3a** was prepared according to the general procedure **C** using 2-(acetyloxymethyl)cyclohex-2-en-1-one **2a** (10 mmol, 1.6 g) and nitroethane (12 mmol, 0.9 g) in 15 mL EtOH. The title compound was obtained after silica gel chromatography as a brown oil. The spectral data are in good agreement with previous reports [2]. Yield = 56% (1.02 g). Chromatography conditions: diethyl ether/petroleum ether 50/50. R_f = 0.50 (diethyl ether/petroleum ether 50/50).

^{1}H NMR (300 MHz, $CDCl_3$) δ = 1.53 (d, *J* = 6.7 Hz, 3H), 1.94-2.03 (m, 2H), 2.36 (q, *J* = 4.8 Hz, 2H), 2.42-2.45 (m, 2H), 2.55-2.63 (m, 1H), 2.73-2.81 (m, 1H), 4.71-4.86 (m, 1H), 6.80 (t, *J* = 4.1 Hz, 1H).

- ***2-(2-Nitrobutyl)cyclohex-2-en-1-one (3b)*** *[3]*

Compound **3b** was prepared according to the general procedure **C** using 2-(acetyloxymethyl)cyclohex-2-en-1-one **2a** (10 mmol, 1.6 g) and nitropropane (12 mmol, 1.1 g) in 15 mL EtOH. The title compound was obtained after silica gel chromatography as a brown oil. Yield = 48% (0.94 mg). Chromatography conditions: diethyl ether/petroleum ether 50/50. R_f = 0.50 (diethyl ether/petroleum ether 50/50). Although this compound was reported in the literature it was not fully characterized and we completed its characterization.

^{1}H NMR (300 MHz, $CDCl_3$) δ = 0.96 (t, *J* = 7.4 Hz, 3H), 1.77-1.85 (m, 1H), 1.91-2.03 (m, 3H), 2.32-2.37 (m, 2H), 2.41-2.46 (m, 2H), 2.48-2.53 (m, 1H), 2.78-2.87 (m, 1H), 4.56-4.64 (m, 1H), 6.78 (t, *J* = 4.1 Hz, 1H).

^{13}C NMR (75 MHz, $CDCl_3$) δ = 10.4 (CH_3), 23.0 (CH_2), 26.2 (CH_2), 27.4 (CH_2), 35.0 (CH_2), 38.3 (CH_2), 89.5 (CH), 134.3 (C), 149.5 (CH), 199.0 (C).

HRMS (ESI, m/z) calcd for $C_{10}H_{15}NNaO_3$ $[M+Na]^+$ = 220.0944 found 220.0965.

- ***4,4-Dimethyl-2-(2-nitropropyl)cyclohex-2-en-1-one (3c)***

Compound **3c** was prepared according to the general procedure **C** using (2-(acetyloxymethyl)-4,4-dimethylcyclohex-2-en-1-one **2b** (10 mmol, 1.96 g) and nitroethane (12 mmol, 0.9 mg) in 15 mL EtOH. The title compound was obtained after silica gel chromatography as a yellow oil. Yield = 58% (1.21 g). Chromatography conditions: diethyl ether/petroleum ether 50/50. R_f = 0.50 (diethyl ether/petroleum ether 50/50).

^{1}H NMR (400 MHz, $CDCl_3$) δ = 1.09 (d, *J* = 8.4 Hz, 6H), 1.48 (d, *J* = 6.6 Hz, 3H), 1.79 (t, *J* = 6.8 Hz, 2H), 2.39-2.72 (m, 3H), 2.72 (dd, *J* = 4.4, 13.8 Hz, 1H), 4.68-4.73 (m, 1H), 6.39 (s, 1H).

^{13}C NMR (101 MHz, $CDCl_3$) δ = 18.9 (CH_3), 27.47 (CH_3), 27.51 (CH_3), 32.9 (C), 34.2 (CH_2), 35.7 (CH_2), 35.8 (CH_2), 82.5 (CH), 130.9 (C), 158.4 (CH), 198.3 (C).

HRMS (ESI, m/z) calcd for $C_{11}H_{17}NNaO_3$ $[M+Na]^+$ = 234.1101 found 234.1110 and for $C_{11}H_{17}NKO_3$ $[M+K]^+$ = 250.0840 found 250.0836.

- ***4,4-Dimethyl-2-(2-nitrobutyl)cyclohex-2-en-1-one (3d)***

Compound **3d** was prepared according to the general procedure **C** using (2-(acetyloxymethyl)-4,4-dimethylcyclohex-2-en-1-one **2b** (10 mmol, 1.96 g) and nitropropane (12 mmol, 1.07 g) in 15 mL EtOH. The title compound was obtained after silica gel chromatography as a yellow oil. Yield = 61% (1.31 g). Chromatography conditions: diethyl ether/petroleum ether 50/50. R_f = 0.50 (diethyl ether/petroleum ether 50/50).

^{1}H NMR (400 MHz, $CDCl_3$) δ = 0.95 (t, *J* = 7.4 Hz, 3H), 1.11 (d, *J* = 8.7 Hz, 6H), 1.81 (t, *J* = 6.9 Hz, 3H), 1.90-2.00 (m, 1H), 2.36 (dd, *J* = 10.6, 13.8 Hz, 1H), 2.45 (td, *J* = 3.2, 6.6 Hz, 2H), 1.81 (dd, *J* = 3.6, 13.8 Hz, 1H), 4.51-4.58 (m, 1H), 6.40 (s, 1H).

^{13}C NMR (101 MHz, $CDCl_3$) δ = 10.4 (CH_3), 27.3 (CH_2), 27.8 (CH_3), 27.9 (CH_2), 33.3 (CH_2), 34.6 (CH_2), 34.7 (C), 36.1 (CH_2), 89.7 (CH), 131.2 (CH), 158.7 (C), 198.7 (C).

HRMS (ESI, m/z) calcd for $C_{12}H_{19}NNaO_3$ $[M+Na]^+$ = 248.1257 found 248.1295.

d. Ylides 4a–g

- ***3,3-Dimethyl-1-(triphenylphosphoranylidene)butan-2-one (4a)*** *[9,10]*

Compound **4a** was prepared according to the general procedure **E** using 1-bromo-3,3-dimethylbutan-2-one (14 mmol, 2.5 g) and PPh_3 (14.1 mmol, 3.70 g) in 30 mL THF. Basic aqueous treatment (aqueous NaOH – 20 wt %, 58 mL) of the isolated phosphonium bromine yielded the phosphonium ylide **4a** without purification as a yellow solid. The spectral data are in good agreement with previous reports [9,10]. Yield = 50% (2.65 g).

^{1}H NMR (400 MHz, $CDCl_3$) δ = 1.15 (s, 9H), 3.74 (d, J_{PH} = 27.1 Hz, 1H), 7.21-7.37 (m, 9H), 7.47-7.60 (m, 6H). R_f = 0.05 (EtOAc 100%).

- ***1-Phenyl-2-(triphenylphosphoranylidene)ethan-1-one (4b)*** *[5]*

Compound **4b** was prepared according to the general procedure **E** using 2-bromo-1-phenylethan-1-one (25 mmol, 5 g) and PPh_3 (25.25 mmol, 6.72 g) in 54 mL THF. Basic aqueous treatment (aqueous NaOH - 20 wt %, 104 mL) of the isolated phosphonium bromine yielded the phosphonium ylide **4b** without purification as a white solid. The spectral data are in good agreement with previous reports [5]. Yield = 93% (8.81 g).

^{1}H NMR (400 MHz, $CDCl_3$) δ = 4.45 (d, J_{PH} = 24.4 Hz, 1H), 7.30-7.38 (m, 3H), 7.37-7.62 (m, 9H), 7.64-7.82 (m, 6H), 7.92-8.05 (m, 2H). R_f = 0.05 (EtOAc 100%).

- ***1-([1,1'-Biphenyl]-4-yl)-2-(triphenylphosphoranylidene)ethan-1-one (4c)*** *[11]*

Compound **4c** was prepared according to the general procedure **E** using 1-([1,1'-biphenyl]-4-yl)-2-bromoethan-1-one (10 mmol, 2.75 g) and PPh_3 (10.1 mmol, 2.67 g) in 22 mL THF. Basic aqueous treatment (aqueous NaOH - 20 wt %, 42 mL) of the isolated phosphonium bromine yielded the phosphonium ylide **4c** without purification as a yellow solid. The spectral data are in good agreement with previous reports [11]. Yield = 66% (3 g).

^{1}H NMR (400 MHz, $CDCl_3$) δ = 4.39 (d, J_{PH} = 24.4 Hz, 1H), 7.20-7.26 (m, 1H), 7.34-7.42 (m, 7H), 7.43-7.55 (m, 8H), 7.62-7.69 (m, 7H), 7.97 (d, J = 7.9 Hz, 2H). R_f = 0.05 (EtOAc 100%).

- ***1-(4-Iodophenyl)-2-(triphenylphosphoranylidene)ethan-1-one (4d)*** *[12]*

Compound **4d** was prepared according to the general procedure **E** using 2-bromo-1-(4-iodophenyl)ethan-1-one[5,13] (9.3 mmol, 3 g) and PPh_3 (9.4 mmol, 2.46 g) in 20 mL THF. Basic aqueous treatment (aqueous NaOH - 20 wt %, 38 mL) of the isolated phosphonium bromine yielded the phosphonium ylide **4d** without purification as a yellow solid. The spectral data are in good agreement with previous reports [12]. Yield = 86% (3.8 g).

^{1}H NMR (400 MHz, $CDCl_3$) δ = 4.39 (d, J_{PH} = 23.5 Hz, 1H), 7.43-7.49 (m, 6H), 7.53-7.56 (m, 3H), 7.65-7.75 (m, 10H). R_f = 0.05 (EtOAc 100%).

- ***1-(4-Fluorophenyl)-2-(triphenylphosphoranylidene)ethan-1-one (4e)*** *[5,14]*

Compound **4e** was prepared according to the general procedure **E** using 2-bromo-1-(4-fluorophenyl)ethan-1-one (5 mmol, 1.1 g) and PPh_3 (5.1 mmol, 1.34 g) in 11 mL THF. Basic aqueous treatment (aqueous NaOH - 20 wt %, 21 mL) of the isolated phosphonium bromine yielded the phosphonium ylide **4e** without purification as a white solid. The spectral data are in good agreement with previous reports [5,14]. Yield = 85% (1.70 g).

^{1}H NMR (400 MHz, $CDCl_3$) δ = 4.27 (d, J_{PH} = 24.0 Hz, 1H), 6.86-6.96 (m, 2H), 7.36-7.40 (m, 6H), 7.42-7.51 (m, 3H), 7.55-7.69 (m, 6H), 7.82-7.92 (m, 2H). R_f = 0.05 (EtOAc 100%).

- ***1-(Thiophen-2-yl)-2-(triphenylphosphoranylidene)ethan-1-one (4f)*** *[4]*

Compound **4f** was prepared according to the general procedure **E** using 2-bromo-1-(thiophen-2-yl)ethan-1-one[15,16] (7.8 mmol, 1.60 g) and PPh_3 (7.9 mmol, 2.1 g) in 17 mL THF. Basic aqueous treatment (aqueous NaOH - 20 wt %, 32 mL) of the isolated phosphonium bromine yielded the phosphonium ylide **4f** without purification as a yellow solid. The spectral data are in good agreement with previous reports.[4] Yield = 65% (1.73 g).

^{1}H NMR (400 MHz, $CDCl_3$) δ = 4.23 (d, J_{PH} = 21.2 Hz, 1H), 6.93-6.96 (m, 1H), 7.17-7.23 (m, 1H), 7.38-7.42 (m, 6H), 7.44-7.53 (m, 4H), 7.59-7.68 (m, 6H). R_f = 0.05 (EtOAc 100%).

- ***1-(Pyridin-2-yl)-2-(triphenylphosphoranylidene)ethan-1-one (4g)*** *[17]*

Compound **4g** was prepared according to the general procedure **E** using 2-(2-bromoacetyl)pyridin-1-ium bromide[18] (20 mmol, 4 g) and PPh_3 (20.2 mmol, 5.30 g) in 44 mL THF. Basic aqueous treatment (aqueous NaOH - 20 wt %, 82 mL) of the isolated

phosphonium bromine yielded the phosphonium ylide **4g** without purification as a yellow solid. Yield = 47% (3.60 g). R_f = 0.05 (EtOAc 100%).

^{1}H NMR (400 MHz, $CDCl_3$) δ = 4.97-5.20 (m, 1H), 7.06 (dd, *J* = 7.6, 5.0 Hz, 1H), 7.23-7.30 (m, 6H), 7.31-7.38 (m, 3H), 7.54 (dd, *J* = 13.0, 7.5 Hz, 7H), 7.94 (d, *J* = 7.9 Hz, 1H), 8.38 (d, *J* = 4.7 Hz, 1H).

^{13}C NMR (101 MHz, $CDCl_3$) δ = 51.8 (d, J_{C-P} = 111.1 Hz, CH, P=CH), 120.6 (d, J_{C-P} = 1.6 Hz, CH), 124.2 (CH), 126.7 (d, J_{C-P} = 91.3 Hz, 3 × C), 128.9 (d, J_{C-P} = 12.3 Hz, 6 x CH), 132.2 (d, J_{C-P} = 3.0 Hz, 3 × CH), 133.3 (d, J_{C-P} = 10.3 Hz, 6 x CH), 136.6 (CH), 148.1 (CH), 157.6 (d, J_{C-P} = 14.1 Hz, C), 182.9 (d, J_{C-P} = 4.8 Hz, C, C=O). ^{31}P (^{1}H) NMR ($CDCl_3$, 162 MHz) δ = 17.4.

HRMS (ESI, m/z) calcd for $C_{25}H_{21}NOP$ $[M+H]^+$ = 382.1355 found 382.1378 and for $C_{25}H_{20}NNaOP$ $[M+Na]^+$ = 404.1175 found 404.1195.

e. 1,4-Diketones 5a–k

- ***2-(2-Oxopropyl)cyclohex-2-en-1-one (5a)*** *[19]*

Compound **5a** was prepared according to the general procedure **D** using 2-(2-nitropropyl)cyclohex-2-en-1-one **3a** (5.17 mmol, 0.90 g). The title compound was obtained after silica gel chromatography as a yellow oil. Yield = 69% (540 mg). Chromatography conditions: diethyl ether/petroleum ether 75/25. R_f = 0.40 (diethyl ether/petroleum ether 75/25).

^{1}H NMR (200 MHz, $CDCl_3$) δ = 2.03 (p, *J* = 6.2 Hz, 2H), 2.19 (s, 3H), 2.34-2.53 (m, 4H), 3.27 (s, 2H), 6.81 (t, *J* = 4.2 Hz, 1H).

^{13}C NMR (75 MHz, $CDCl_3$) δ = 22.9 (CH_2), 26.0 (CH_2), 29.9 (CH_3), 37.8 (CH_2), 44.3 (CH_2), 134.0 (C), 148.9 (CH), 198.5 (C), 206.9 (C).

HRMS (ESI, m/z) calcd for $C_9H_{12}NaO_2$ $[M+Na]^+$ = 175.0730 found 175.0737.

- ***2-(2-Oxobutyl)cyclohex-2-en-1-one (5b)***

Compound **5b** was prepared according to the general procedure **D** using 2-(2-nitrobutyl)cyclohex-2-en-1-one **3b** (5.17 mmol, 1.02 g). The title compound was obtained

after silica gel chromatography as a yellow oil. Yield = 73% (630 mg). Chromatography conditions: diethyl ether/petroleum ether 75/25. R_f = 0.45 (diethyl ether/petroleum ether 75/25).

^{1}H NMR (300 MHz, $CDCl_3$) δ = 0.97 (t, *J* = 7.3 Hz, 3H), 1.92-2.00 (m, 2H), 2.33-2.49 (m, 4H), 2.44 (q, *J* = 7.3 Hz, 2H), 3.18 (s, 2H), 6.74 (t, *J* = 4.1 Hz, 1H).

^{13}C NMR (75 MHz, $CDCl_3$) δ = 7.7 (CH_3), 22.9 (CH_2), 26.1 (CH_2), 35.9 (CH_2), 37.9 (CH_2), 43.0 (CH_2), 134.3 (C), 148.7 (CH), 198.5 (C), 208.6 (C).

HRMS (ESI, m/z) calcd for $C_{10}H_{14}NaO_2$ $[M+Na]^+$ = 189.0886 found 189.0917.

- ***4,4-Dimethyl-2-(2-oxopropyl)cyclohex-2-en-1-one (5c)***

Compound **5c** was prepared according to the general procedure **D** using 4,4-dimethyl-2-(2-nitropropyl)cyclohex-2-en-1-one **3c** (5.17 mmol, 1.10 g). The title compound was obtained after silica gel chromatography as a yellow oil. Yield = 87% (810 mg). Chromatography conditions: diethyl ether/petroleum ether 75/25. R_f = 0.40 (diethyl ether/petroleum ether 75/25).

^{1}H NMR (400 MHz, $CDCl_3$) δ = 1.14 (s, 6H), 1.84 (t, *J* = 6.8 Hz , 2H), 2.13 (s, 3H), 2.44 (t, *J* = 6.8 Hz, 2H), 3.18 (s, 2H), 6.43 (s, 1H).

^{13}C NMR (101 MHz, $CDCl_3$) δ = 27.9 (2 x CH_3), 29.9 (CH_3), 33.3 (C), 34.3 (CH_2), 36.2 (CH_2), 44.1 (CH_2), 131.1 (C), 158.1 (CH), 198.3 (C), 205.8 (C).

HRMS (ESI, m/z) calcd for $C_{11}H_{16}NaO_2$ $[M+Na]^+$ = 203.1043 found 203.1087.

- ***4,4-Dimethyl-2-(2-oxobutyl)cyclohex-2-en-1-one (5d)***

Compound **5d** was prepared according to the general procedure **D** using 4,4-dimethyl-2-(2-nitrobutyl)cyclohex-2-en-1-one **3d** (5.17 mmol, 1.16 g). The title compound was obtained after silica gel chromatography as a yellow oil. Yield = 61% (611 mg). Chromatography conditions: diethyl ether/petroleum ether 75/25. R_f = 0.40 (diethyl ether/petroleum ether 75/25).

^{1}H NMR (400 MHz, $CDCl_3$) δ = 0.97 (t, *J* = 7.3 Hz ,3H), 1.11 (s, 6H), 1.81 (t, *J* = 6.7 Hz ,2H), 2.39-2.45 (m, 4H), 3.14 (s, 2H), 6.41 (s, 1H).

^{13}C NMR (101 MHz, $CDCl_3$) δ = 7.7 (CH_3), 27.9 (2 x CH_3), 33.3 (C), 34.2 (CH_2), 35.8 (CH_2), 36.2 (CH_2), 42.9 (CH_2), 131.1 (C), 157.9 (CH), 198.3 (C), 208.4 (C).

HRMS (ESI, m/z) calcd for $C_{12}H_{19}O_2$ $[M+H]^+$ = 195.1380 found 195.1339 and for $C_{12}H_{18}NaO_2$ $[M+Na]^+$ = 217.1199 found 217.1223.

- ***2-(3,3-Dimethyl-2-oxobutyl)cyclohex-2-en-1-one (5e)***

Compound **5e** was prepared according to the general procedure **F** using 3,3-dimethyl-1-(triphenylphosphoranylidene)butan-2-one **4a** (7.4 mmol, 2.65 g) and cyclohexan-1,2-dione (7.77 mmol, 0.87 g) in 37 mL CH_2Cl_2. The title compound was obtained after flash chromatography as a yellow oil. Yield = 46% (0.66 g). Flash chromatography conditions: column 40g, flow rate= 20 mL/min, cyclohexane/EtOAc 100/0 to 50/50. R_f = 0.40 (cyclohexane/EtOAc 50/50).

^1H NMR (400 MHz, $CDCl_3$) δ = 1.17 (s, 9H), 2.01 (p, *J* = 6.3 Hz, 2H), 2.37-2.49 (m, 4H), 3.34 (s, 2H), 6.73 (t, *J* = 4.3 Hz, 1H).

^{13}C NMR (101 MHz, $CDCl_3$) δ = 23.1 (CH_2), 26.2 (CH_2), 26.6 (3 × CH_3), 37.7 (CH_2), 38.1 (CH_2), 44.5 (C), 134.9 (C), 148.5 (CH), 198.5 (C), 213.1 (C).

HRMS (ESI, m/z) calcd for $C_{12}H_{18}NaO_2$ $[M+Na]^+$ = 217.1199 found 217.1240.

- ***2-(2-Oxo-2-phenylethyl)cyclohex-2-en-1-one (5f)*** *[20]*

Compound **5f** was prepared according to the general procedure **F** using 1-phenyl-2-(triphenylphosphoranylidene)ethan-1-one **4b** (23 mmol, 8.80 g) and cyclohexan-1,2-dione (24.15 mmol, 2.7 g) in 115 mL CH_2Cl_2. The title compound was obtained after flash chromatography as a yellow oil. Yield = 73% (3.58 g). Flash chromatography conditions: column 40 g, flow rate= 20 mL/min, cyclohexane/EtOAc 100/0 to 50/50. R_f = 0.40 (cyclohexane/EtOAc 50/50). Although this compound was reported in the literature it was not fully characterized and we completed its characterization.

^1H NMR (400 MHz, $CDCl_3$) δ = 2.05 (dt, *J* = 12.4, 6.1 Hz, 2H), 2.40-2.45 (m, 2H), 2.47-2.52 (m, 2H), 3.85 (s, 2H), 6.87 (t, *J* = 4.2 Hz, 1H), 7.45(dd, *J* = 7.6, 7.6 Hz, 2H), 7.49-7.60 (m, 1H), 7.97 (dd, *J* = 8.4, 1.3 Hz, 2H).

^{13}C NMR (76 MHz, $CDCl_3$) δ = 23.1 (CH_2), 26.3 (CH_2), 38.1 (CH_2), 39.0 (CH_2), 128.5 (2 x CH), 128.7 (2 x CH), 133.2 (CH), 134.2 (C), 136.8 (C), 148.7 (CH), 197.7 (C), 198.4 (C).

HRMS (ESI, m/z) calcd for $C_{14}H_{14}NaO_2$ $[M+Na]^+$ = 237.0886 found 237.0893 and for $C_{14}H_{14}KO_2$ $[M+K]^+$ = 253.0625 found 253.0629.

- ***2-(2-([1,1'-Biphenyl]-4-yl)-2-oxoethyl)cyclohex-2-en-1-one (5g)***

Compound **5g** was prepared according to the general procedure **F** using 1-([1,1'-biphenyl]-4-yl)-2-(triphenylphosphoranylidene)ethan-1-one **4c** (4.25 mmol, 1.94 g) and cyclohexan-1,2-dione (4.46 mmol, 0.5 g) in 21 mL CH_2Cl_2. The title compound was obtained after flash chromatography as a yellow solid. Yield = 57% (700 mg). mp 135 °C. Flash chromatography conditions: column 40g, flow rate= 20 mL/min, cyclohexane/EtOAc 100/0 to 50/50. R_f = 0.40 (cyclohexane/EtOAc 50/50).

^{1}H NMR (400 MHz, $CDCl_3$) δ = 2.02-2.09 (m, 2H), 2.44(td, *J* = 6.0, 4.4 Hz, 2H), 2.48-2.53 (m, 2H), 3.83 (s, 2H), 6.89 (t, *J* = 4.2 Hz, 1H), 7.39 (dd, *J* = 7.3, 7.3 Hz, 1H), 7.47 (dd, *J* = 7.4, 7.4 Hz, 2H), 7.62 (d, *J* = 7.1 Hz, 2H), 7.68 (d, *J* = 8.4 Hz, 2H), 8.06 (d, *J* = 8.4 Hz, 2H).

^{13}C NMR (101 MHz, $CDCl_3$) δ = 23.1 (CH_2), 26.3 (CH_2), 38.2 (CH_2), 39.0 (CH_2), 127.3 (2 x CH), 127.4 (2 x CH), 128.3 (CH), 129.0 (2 x CH), 129.1 (2 x CH), 134.3 (C), 136.5 (C), 140.0 (C), 145.9 (C), 148.9 (CH), 197.3 (C), 198.4 (C).

HRMS (ESI, m/z) calcd for $C_{20}H_{18}NaO_2$ $[M+Na]^+$ = 313.1199 found 313.1199 and for $C_{20}H_{18}KO_2$ $[M+K]^+$ = 329.0938 found 329.0943.

- ***2-(2-(4-Iodophenyl)-2-oxoethyl)cyclohex-2-en-1-one (5h)***

Compound **5h** was prepared according to the general procedure **F** using 1-(4-iodophenyl)-2-(triphenylphosphoranylidene)ethan-1-one **4d** (7.62 mmol, 3.85 g) and cyclohexan-1,2-dione (8 mmol, 0.90 g) in 38 mL CH_2Cl_2. The title compound was obtained after flash chromatography as a yellow solid. Yield = 58% (1.50 g). mp 85 °C. Flash chromatography conditions: column 40g, flow rate= 20 mL/min, cyclohexane/EtOAc 100/0 to 50/50. R_f = 0.40 (cyclohexane/EtOAc 50/50).

^{1}H NMR (400 MHz, $CDCl_3$) δ = 2.05 (p, *J* = 6.3 Hz, 2H), 2.43 (q, *J* = 5.3 Hz, 2H), 2.49 (t, *J* = 6.7 Hz, 2H), 3.79 (s, 2H), 6.87 (t, *J* = 4.3 Hz, 1H), 7.68(d, *J* = 8.2 Hz, 2H), 7.81(d, *J* = 8.1 Hz, 2H).

^{13}C NMR (101 MHz, $CDCl_3$) δ = 23.1 (CH_2), 26.3 (CH_2), 38.1 (CH_2), 38.9 (CH_2), 101.2 (C), 129.3 (2 x CH), 134.0 (C), 136.1 (C), 138.0 (2 x CH), 149.1 (CH), 197.0 (C), 198.4 (C).

HRMS (ESI, m/z) calcd for $C_{14}H_{13}INaO2$ $[M+Na]^+$ = 362.9852 found 362.9896.

- ***2-(2-(4-Fluorophenyl)-2-oxoethyl)cyclohex-2-en-1-one (5i)***

Compound **5i** was prepared according to the general procedure **F** using 1-(4-fluorophenyl)-2-(triphenylphosphoranylidene)ethan-1-one **4e** (4.3 mmol, 1.70 g) cyclohexan-1,2-dione (4.15 mmol, 0.50 g) in 22 mL CH_2Cl_2. The title compound was obtained after flash chromatography as a yellow oil. Yield = 50% (0.5 g). Flash chromatography conditions: column 40g, flow rate= 20 mL/min, cyclohexane/EtOAc 100/0 to 50/50. R_f = 0.40 (cyclohexane/EtOAc 50/50).

^{1}H NMR (400 MHz, $CDCl_3$) δ = 2.03 (p, *J* = 6.1Hz, 2H), 2.36-2.44 (m, 2H), 2.45-2.50 (m, 2H), 3.79 (s, 2H), 6.86 (t, *J* = 4.1 Hz, 1H), 7.10 (dd, *J* = 8.9, 8.4 Hz, 2H), 7.93-8.03 (m, 2H).

^{13}C NMR (101 MHz, $CDCl_3$) δ = 23.1 (CH_2), 26.3 (CH_2), 38.1 (CH_2), 38.9 (CH_2), 115.0 (d, *J* = 21.8 Hz, 2 x CH), 131.1 (d, J_{C-F} = 9.4 Hz, 2 x CH), 133.2 (d, J_{C-F} = 3.1 Hz, C), 134.0 (C), 149.0 (CH), 165.8 (d, J_{C-F} = 254.6 Hz, C), 196.1 (C), 198.4 (C).

^{19}F NMR (376 MHz, $CDCl_3$) δ = -105.3 (s).

HRMS (ESI, m/z) calcd for $C_{14}H_{14}$FO2 $[M+H]^+$ = 233.0972 found 233.0973.

- ***2-(2-Oxo-2-(thiophen-2-yl)ethyl)cyclohex-2-en-1-one (5j)***

Compound **5j** was prepared according to the general procedure **F** using 1-(thiophen-2-yl)-2-(triphenylphosphoranylidene)ethan-1-one **4f** (3.6 mmol, 1.38 g) and cyclohexan-1,2-dione (3.32 mmol, 371 mg) in 18 mL CH_2Cl_2. The title compound was obtained after flash chromatography as a yellow solid. Yield = 51% (400 mg). mp 60 °C. Flash chromatography conditions: column 40g, flow rate= 20 mL/min, cyclohexane/EtOAc 100/0 to 50/50. R_f = 0.40 (cyclohexane/EtOAc 50/50).

^{1}H NMR (400 MHz, $CDCl_3$) δ = 2.02 (p, *J* = 6.1 Hz, 2H), 2.34-2.42(m, 2H), 2.44-2.51 (m, 2H), 3.77 (s, 2H), 6.91 (t, *J* = 4.2 Hz, 1H), 7.10 (dd, *J* = 5.0, 3.8 Hz, 1H), 7.61 (dd, *J* = 4.9, 1.2 Hz, 1H), 7.78 (dd, *J* = 3.8, 1.2 Hz, 1H).

^{13}C NMR (101 MHz, $CDCl_3$) δ = 23.0 (CH_2), 26.3 (CH_2), 38.1 (CH_2), 39.4 (CH_2), 128.2 (CH), 132.7 (CH), 133.7 (C), 133.9 (CH), 144.0 (C), 149.1 (CH), 190.4 (C), 198.2 (C).

HRMS (ESI, m/z) calcd for $C_{12}H_{13}O_2S$ $[M+H]^+$ = 221.0631 found 221.0594 and for $C_{12}H_{12}NaO_2S$ $[M+Na]^+$ = 243.0450 found 243.0488.

- ***2-(2-Oxo-2-(pyridin-2-yl)ethyl)cyclohex-2-en-1-one (5k)***

Compound **5k** was prepared according to the general procedure **F** using 1-(pyridin-2-yl)-2-(triphenylphosphoranylidene)ethan-1-one **4g** (4 mmol, 1.52 g) and cyclohexan-1,2-dione (4.2 mmol, 470 mg) in 20 mL toluene. The title compound was obtained after flash chromatography as a yellow solid. Yield = 61% (522 mg). mp 76 °C. Flash chromatography conditions: column 40g, flow rate= 20 mL/min, cyclohexane/EtOAc 100/0 to 50/50. R_f = 0.40 (cyclohexane/EtOAc 50/50).

^{1}H NMR (400 MHz, $CDCl_3$) δ = 2.02 (p, *J* = 6.1 Hz, 2H), 2.37-2.41(m, 2H), 2.43-2.50 (m, 2H), 4.07 (s, 2H), 6.82 (t, *J* = 4.1 Hz, 1H), 7.42 (ddd, *J* = 7.6, 4.8, 1.3 Hz, 1H), 7.77 (ddd, *J* = 7.7, 7.7, 1.7 Hz, 1H), 7.96 (ddd, *J* = 7.9, 1.1, 1.1 Hz, 1H), 8.63 (ddd, *J* = 4.8, 1.8, 0.9 Hz, 1H).

^{13}C NMR (101 MHz, $CDCl_3$) δ = 23.4 (CH_2), 26.5 (CH_2), 38.4 (CH_2), 39.1 (CH_2), 122.3 (CH), 127.5 (CH), 135.1 (C), 137.2 (CH), 148.9 (CH), 149.2 (CH), 153.5 (C), 198.7 (C), 199.1 (C).

HRMS (ESI, m/z) calcd for $C_{13}H_{14}NO_2$ $[M+H]^+$ = 216.1019 found 216.1043 and for $C_{13}H_{13}NNaO_2$ $[M+Na]^+$ = 238.0838 found 238.0877.

f. Indoles 6a–l

- ***1-Benzyl-2-methyl-1H-indole (6a)*** *[21]*

Compound **6a** was prepared according to the general procedure **G** using 2-(2-oxopropyl)cyclohex-2-en-1-one **5a** (0.59 mmol, 90 mg) and benzylamine (0.88 mmol, 94 mg) in 4.5 mL toluene. The title compound was obtained after flash chromatography as a yellow oil. Yield = 54% (65 mg). Flash chromatography conditions: column 12 g, flow rate = 18 mL/min, cyclohexane/EtOAc 100/0 to 65/35. R_f = 0.50 (cyclohexane/EtOAc 90/10).

^{1}H NMR (400 MHz, $CDCl_3$) δ = 2.35 (s, 3H), 5.29 (s, 2H), 6.31 (s, 1H), 6.96 (d, *J* = 6.5 Hz, 2H), 7.04-7.10 (m, 2H), 7.17-7.25 (m, 4H), 7.51-7.57 (m, 1H).

^{13}C NMR (101 MHz, $CDCl_3$) δ = 12.9 (CH_3), 47.0 (CH_2), 100.6 (CH), 109.3 (CH), 119.7 (CH), 119.8 (CH), 120.9 (CH), 126.1 (2 x CH), 127.4 (CH), 128.3 (C), 128.9 (2 x CH), 136.8 (C), 137.3 (C), 138.0 (C).

HRMS (APCI, m/z) calcd for $C_{16}H_{16}N$ $[M+H]^+$ = 222.1277 found 222.1305.

- ***1-Benzyl-2-ethyl-1H-indole (6b)*** *[21]*

Compound **6b** was prepared according to the general procedure **G** using -(2-oxobutyl)cyclohex-2-en-1-one **5b** (0.54 mmol, 90 mg) and phenylmethanamine (0.81 mmol, 86 mg) in 4 mL toluene. The title compound was obtained after flash chromatography as a yellow oil. Yield = 47% (60 mg). Flash chromatography conditions: column 12 g, flow rate = 18 mL/min, cyclohexane/EtOAc 100/0 to 65/35. R_f = 0.50 (cyclohexane/EtOAc 90/10).

^{1}H NMR (400 MHz, $CDCl_3$) δ = 1.24 (t, *J* = 7.4 Hz, 3H), 2.61 (q, *J* = 7.8 Hz, 2H), 5.24 (s, 2H), 6.28 (s, 1H), 6.88 (d, *J* = 7.2 Hz, 2H), 6.97-7.04 (m, 2H), 7.12-7.19 (m, 4H), 7.48-7.52 (m, 1H).

^{13}C NMR (101 MHz, $CDCl_3$) δ = 12.7 (CH_3), 20.1 (CH_2), 46.5 (CH_2), 98.7 (CH), 104.3 (CH), 119.6 (CH), 120.0 (CH), 121.0 (CH), 126.1 (2 x CH), 127.3 (CH), 128.3 (C), 128.9 (2 x CH), 137.4 (C), 138.1 (C), 143.0 (C).

HRMS (APCI, m/z) calcd for $C_{17}H_{18}N$ $[M+H]^+$ = 236.1434 found 236.1429 and for $C_{17}H_{17}NNa$ $[M+Na]^+$ = 258.1253 found 258.1275.

- ***2-Ethyl-1-phenethyl-1H-indole (6c)***

Compound **6c** was prepared according to the general procedure **G** using 2-(2-oxobutyl)cyclohex-2-en-1-one **5b** (0.54 mmol, 90 mg) and 2-phenethylamine (0.81 mmol, 98 mg) in 4 mL toluene. The title compound was obtained after flash chromatography as a yellow oil. Yield = 47% (63 mg). Flash chromatography conditions: column 12 g, flow rate = 18 mL/min, cyclohexane/EtOAc 100/0 to 65/35. R_f = 0.50 (cyclohexane/EtOAc 90/10).

^{1}H NMR (400 MHz, $CDCl_3$) δ = 1.19 (t, *J* = 7.6 Hz, 3H), 2.42 (q, *J* = 7.4 Hz 2H), 2.94 (t, *J* = 7.6 Hz , 2H), 4.19 (t, *J* = 7.6 Hz, 2H), 6.16 (s, 1H), 6.99-7.03 (m, 3H), 7.07 (t, *J* = 7.6 Hz, 2H), 7.19 (d, *J* = 7.5 Hz, 1H), 7.24 (d, *J* = 8.3 Hz, 2H), 7.48 (d, *J* = 7.7 Hz, 1H).

^{13}C NMR (101 MHz, $CDCl_3$) δ = 12.6 (CH_3), 19.8 (CH_2), 36.5 (CH_2), 44.9 (CH_2), 98.1 (CH), 109.0 (CH), 119.4 (CH), 120.0 (CH), 120.7 (CH), 126.8 (CH), 128.4 (C), 128.8 (2 x CH), 129.0 (2 x CH), 136.5 (C), 138.8 (C), 142.8 (C).

HRMS (APCI, m/z) calcd for $C_{18}H_{20}N$ $[M+H]^+$ = 250.1590 found 250.1597.

- ***2-Ethyl-1-(4-methoxybenzyl)-1H-indole (6d)***

Compound **6d** was prepared according to the general procedure **G** using 2-(2-oxobutyl)cyclohex-2-en-1-one **5b** (0.54 mmol, 90 mg) and 4-methoxybenzylamine (0.81 mmol, 111 mg) in 4 mL toluene. The title compound was obtained after flash chromatography as a yellow oil. Yield = 43% (62 mg). Flash chromatography conditions: column 12 g, flow rate = 18 mL/min, cyclohexane/EtOAc 100/0 to 65/35. R_f = 0.50 (cyclohexane/EtOAc 90/10).

^{1}H NMR (400 MHz, $CDCl_3$) δ = 1.24 (t, *J* = 7.5 Hz, 3H), 2.62 (q, *J* = 7.5 Hz, 2H), 3.67 (s, 3H), 5.18 (s, 2H), 6.26 (s, 1H), 6.71 (d, *J* = 8.5 Hz, 2H), 6.82 (d, *J* = 8.5 Hz, 2H), 6.97-7.05 (m, 2H), 7.13 (d, *J* = 7.1 Hz, 1H), 7.50 (dd, *J* = 6.9, 1.7 Hz, 1H).

^{13}C NMR (101 MHz, $CDCl_3$) δ = 11.5 (CH_3), 18.9 (CH_2), 44.8 (CH_2), 54.2 (CH_3), 97.4 (CH), 108.2 (CH), 113.1 (2 x CH), 118.4 (CH), 118.8 (CH), 119.7 (CH), 126.1 (2 x CH), 127.1 (C), 128.9 (C), 136.2 (C), 141.8 (C), 157.7 (C).

HRMS (ESI, m/z) calcd for $C_{18}H_{19}NNaO$ $[M+Na]^+$ = 288.1359 found 288.1370.

- ***2-Ethyl-1-(1-phenylethyl)-1H-indole (6e)*** *[22]*

Compound **6e** was prepared according to the general procedure **G** using 2-(2-oxobutyl)cyclohex-2-en-1-one **5b** (0.54 mmol, 90 mg) and methylbenzylamine (0.81 mmol, 98 mg) in 4 mL toluene. The title compound was obtained after flash chromatography as a yellow oil. Yield = 41% (55 mg). Flash chromatography conditions: column 12 g, flow rate = 18 mL/min, cyclohexane/EtOAc 100/0 to 65/35. R_f = 0.50 (cyclohexane/EtOAc 90/10).

^{1}H NMR (400 MHz, $CDCl_3$) δ = 1.33 (t, *J* = 7.4 Hz, 3H), 1.93 (d, *J* = 7.1 Hz, 3H), 2.66-2.77 (m, 2H), 5.73 (q, *J* = 7.1 Hz, 2H), 6.32 (s, 1H), 6.89-6.96 (m, 2H), 6.97-7.04 (m, 1H), 7.15 (d, *J* = 7.5 Hz, 2H), 7.20-7.30 (m, 3H), 7.53 (d, *J* = 7.8 Hz, 1H).

^{13}C NMR (101 MHz, $CDCl_3$) δ = 13.2 (CH_3), 18.7 (CH_3), 20.9 (CH_2), 52.3 (CH), 99.0 (CH), 111.5 (CH), 119.2 (CH), 120.0 (CH), 120.4 (CH), 126.4 (2 x CH), 127.4 (CH), 128.7 (2 x CH), 128.7 (C), 128.9 (CH), 135.8 (C), 141.6 (C), 143.0 (C).

HRMS (APCI, m/z) calcd for $C_{18}H_{20}N$ $[M+H]^+$ = 250.1590 found 250.1628.

- ***2-Ethyl-1-(furan-2-ylmethyl)-1H-indole (6f)***

Compound **6f** was prepared according to the general procedure **G** using 2-(2-oxobutyl)cyclohex-2-en-1-one **5b** (0.54 mmol, 90 mg) and 3-furylmethylamine (0.81 mmol,

79 mg) in 4 mL toluene. The title compound was obtained after flash chromatography as a yellow oil. Yield = 52% (69 mg). Flash chromatography conditions: column 12 g, flow rate = 18 mL/min, cyclohexane/EtOAc 100/0 to 65/35. R_f = 0.50 (cyclohexane/EtOAc 90/10).

^{1}H NMR (400 MHz, $CDCl_3$) δ = 1.35 (t, *J* = 7.4 Hz, 3H), 2.78 (q, *J* = 7.4 Hz, 2H), 5.16 (s, 2H), 6.01 (d, *J* = 3.3 Hz, 1H), 6.21 (dd, *J* = 3.3, 1.8 Hz, 1H), 6.28 (s, 1H), 7.06 (t, *J* = 7.4 Hz, 1H), 7.13 (t, *J* = 7.6 Hz, 1H) , 7.26 (d, *J* = 1.3 Hz, 1H) , 7.32 (dd, *J* = 8.2, 8.2 Hz, 1H) , 7.53 (dd, *J* = 7.7, 7.7 Hz, 1H).

^{13}C NMR (101 MHz, $CDCl_3$) δ = 13.0 (CH_3), 19.9 (CH_2), 40.1 (CH_2), 98.7 (CH), 107.4 (CH), 109.2 (CH), 110.4 (CH), 119.6 (CH), 119.9 (CH), 120.9 (CH), 128.3 (C), 137.1 (C), 142.2 (CH), 142.6 (C), 151.0 (C).

HRMS (APCI, m/z) calcd for $C_{15}H_{16}NO$ $[M+H]^+$ = 226.1226 found 226.1210 and for $C_{15}H_{15}NNaO$ $[M+Na]^+$ = 248.1046 found 248.1050.

- ***1,3-Bis(2-ethyl-1H-indol-1-yl)propane (6g)***

Compound **6g** was prepared according to the adapted general procedure **G** using -(2-oxobutyl)cyclohex-2-en-1-one **5b** (0.91 mmol, 1 equiv, 152 mg) and 1,3-diaminopropane (0.45 mmol, 0.5 equiv, 34 mg) in 8 mL toluene. The title compound was obtained after flash chromatography as a yellow oil. Yield = 46% (68 mg). Flash chromatography conditions: column 12 g, flow rate = 18 mL/min, cyclohexane/EtOAc 100/0 to 65/35. R_f = 0.50 (cyclohexane/EtOAc 90/10).

^{1}H NMR (400 MHz, $CDCl_3$) δ = 1.31 (t, *J* = 7.4 Hz, 6H), 2.18-2.26 (m, 2H), 2.62 (q, *J* = 7.4 Hz, 4H), 4.09 (t, *J* = 7.2 Hz, 4H), 6.28 (s, 2H), 7.04-7.08 (m, 2H), 7.09-7.15 (m, 4H), 7.55 (d, *J* = 7.5 Hz, 2H).

^{13}C NMR (101 MHz, $CDCl_3$) δ = 13.1 (2 x CH_3), 20.3 (2 x CH_2), 31.0 (CH_2), 40.8 (2 x CH_2), 99.0 (2 x CH), 109.1 (2 x CH), 119.8 (2 x CH), 120.5 (2 x CH), 121.2 (2 x CH), 128.6 (2 x C), 137.0 (2 x C), 142.6 (2 x C).

HRMS (APCI, m/z) calcd for $C_{23}H_{27}N_2$ $[M+H]^+$ = 331.2169 found 331.2162.

- ***3-(2-Ethyl-1H-indol-1-yl)-N,N-dimethylpropan-1-amine (6h)***

Compound **6h** was prepared according to the general procedure **G** using using 2-(2-oxobutyl)cyclohex-2-en-1-one **5b** (1 mmol, 166 mg) and 3-(dimethylamino)-1-propylamine

(1.5 mmol, 153 mg) in 7.5 mL toluene. The title compound was obtained after flash chromatography as a yellow oil. Yield = 45% (103 mg). Flash chromatography conditions: column 12 g, flow rate = 18 mL/min, cyclohexane/EtOAc 100/0 to 65/35. R_f = 0.30 (cyclohexane/EtOAc 60/40).

^{1}H NMR (400 MHz, $CDCl_3$) δ = 1.38 (t, *J* = 7.4 Hz, 3H), 1.92-1.99 (m, 2H), 2.28 (s, 6H), 2.35 (t, *J* = 7.0 Hz, 2H), 2.77 (q, *J* = 7.5 Hz, 2H), 4.14 (t, *J* = 7.3 Hz, 2H), 6.27 (s, 1H), 7.06 (t, *J* = 7.4 Hz, 1H), 7.13 (t, *J* = 7.6 Hz, 1H), 7.30 (d, *J* = 8.2 Hz, 1H), 7.54 (d, *J* = 7.7 Hz, 1H).

^{13}C NMR (101 MHz, $CDCl_3$) δ = 12.8 (CH_3), 20.0 (CH_2), 28.0 (CH_2), 41.0 (CH_2), 45.3 (2 x CH_3), 56.7 (CH_2), 98.2 (CH), 109.1 (CH), 119.3 (CH), 120.0 (CH), 120.7 (CH), 128.3 (C), 136.8 (C), 142.7 (C).

HRMS (ESI, m/z) calcd for $C_{15}H_{23}N_2$ $[M+H]^+$ = 231.1856 found 231.1842.

- ***4-(2-(2-Methyl-1H-indol-1-yl)ethyl)morpholine (6i)*** *[23]*

Compound **6i** was prepared according to the general procedure **G** using 2-(2-oxopropyl)cyclohex-2-en-1-one **5a** (1 mmol, 152 mg) and 4-(2-aminoethyl)morpholine (1.5 mmol, 195 mg) in 7.5 mL toluene. The title compound was obtained after flash chromatography as a yellow oil. Yield = 51% (122 mg). Flash chromatography conditions: column 12 g, flow rate = 18 mL/min, cyclohexane/EtOAc 100/0 to 65/35. R_f = 0.30 (cyclohexane/EtOAc 60/40). Although this compound was reported in the literature it was not fully characterized and we completed its characterization.

^{1}H NMR (400 MHz, $CDCl_3$) δ = 2.55 (s, 3H), 2.60 (t, *J* = 7.4 Hz, 4H), 2.75 (t, *J* = 6.7 Hz, 2H), 3.82 (t, *J* = 7.5 Hz, 4H), 4.30 (t, *J* = 6.5 Hz, 2H), 6.34 (s, 1H), 7.16 (ddd, *J* = 8, 7, 1.1 Hz, 1H), 7.24 (ddd, *J* = 8.2, 7.1, 1.3 Hz, 1H), 7.37 (dd, *J* = 8.1, 1 Hz, 1H), 7.62 (d, *J* = 7.7 Hz, 1H).

^{13}C NMR (101 MHz, $CDCl_3$) δ = 12.9 (CH_3), 41.1 (CH_2), 54.2 (CH_2), 58.0 (CH_2), 67.0 (CH_2), 100.3 (CH), 108.9 (CH), 119.5 (CH), 119.9 (CH), 120.6 (CH), 128.3 (C), 136.5 (C), 136.6 (C).

HRMS (ESI, m/z) calcd for $C_{15}H_{21}N_2O$ $[M+H]^+$ = 245.1648 found 245.1676.

- ***4-(2-(2-Ethyl-1H-indol-1-yl)ethyl)morpholine (6j)*** *[24]*

Compound **6j** was prepared according to the general procedure **G** using 2-(2-oxobutyl)cyclohex-2-en-1-one **5b** (1 mmol, 166 mg) and 4-(2-aminoethyl)morpholine (1.5 mmol, 195 mg) in 8 mL toluene. The title compound was obtained after flash chromatography as a yellow oil. Yield = 55% (142 mg). Flash chromatography conditions: column 12 g, flow rate = 18 mL/min, cyclohexane/EtOAc 100/0 to 65/35. R_f = 0.30 (cyclohexane/EtOAc 60/40). Although this compound was reported in the literature it was not fully characterized and we completed its characterization.

^{1}H NMR (200 MHz, $CDCl_3$) δ = 1.38 (t, *J* = 7.5 Hz, 3H), 2.50 (t, *J* = 6.7 Hz, 4H), 2.64 (t, *J* = 6.7 Hz, 2H), 2.77 (q, *J* = 7.4 Hz, 2H), 3.71 (t, *J* = 6.5 Hz, 4H), 4.20 (t, *J* = 6.7 Hz, 2H), 6.26 (s, 1H), 7.00-7.19 (m, 2H), 7.28 (d, *J* = 7.9 Hz, 1H), 7.53 (d, *J* = 7.9 Hz, 1H).

^{13}C NMR (50 MHz, $CDCl_3$) δ = 12.7 (CH_3), 20.0 (CH_2), 40.9 (CH_2), 54.2 (2 x CH_2), 57.9 (CH_2), 67.0 (2 x CH_2), 98.3 (CH), 108.9 (CH), 119.4 (CH), 120.0 (CH), 120.7 (CH), 128.3 (C), 136.7 (C), 142.6 (C).

HRMS (ESI, m/z) calcd for $C_{16}H_{23}N_2O$ $[M+H]^+$ = 259.1805 found 259.1804.

- ***2-Methyl-1-(pyridin-4-ylmethyl)-1H-indole (6k)***

Compound **6k** was prepared according to the general procedure **G** using 2-(2-oxopropyl)cyclohex-2-en-1-one **5a** (1 mmol, 152 mg) and 4-(aminomethyl)pyridine (1.5 mmol, 162 mg) in 7.5 mL toluene. The title compound was obtained after flash chromatography as a yellow oil. Yield = 50% (111 mg). Flash chromatography conditions: column 12 g, flow rate = 18 mL/min, cyclohexane/EtOAc 100/0 to 65/35. R_f = 0.30 (cyclohexane/EtOAc 60/40).

^{1}H NMR (400 MHz, $CDCl_3$) δ = 2.35 (s, 3H), 5.27 (s, 2H), 6.38 (s, 1H), 6.85 (d, *J* = 5.8 Hz, 2H), 7.09-7.15 (m, 3H), 7.55-7.63 (m, 1H), 8.49 (d, *J* = 6.1 Hz, 2H).

^{13}C NMR (101 MHz, $CDCl_3$) δ = 12.7 (CH_3), 45.5 (CH_2), 101.2 (CH), 108.9 (CH), 120.0 (CH), 120.1 (2 x CH), 121.1 (CH), 121.2 (CH), 128.3 (C), 137.4 (C), 147.1 (C), 147.3 (C), 150.3 (2 x CH).

HRMS (ESI, m/z) calcd for $C_{15}H_{15}N_2$ $[M+H]^+$ = 223.1230 found 223.1229.

- ***2-Ethyl-1-(pyridin-4-ylmethyl)-1H-indole (6l)***

Compound **6l** was prepared according to the general procedure **G** using 2-(2-oxobutyl)cyclohex-2-en-1-one **5b** (1 mmol, 166 mg) and 4-(aminomethyl)pyridine (1.5 mmol, 162 mg) in 7.5 mL toluene. The title compound was obtained after flash chromatography as a yellow oil. Yield = 52% (110 mg). Flash chromatography conditions: column 12 g, flow rate = 18 mL/min, cyclohexane/EtOAc 100/0 to 65/35. R_f = 0.30 (cyclohexane/EtOAc 60/40).

^{1}H NMR (200 MHz, $CDCl_3$) δ = 1.24 (t, *J* = 7.4 Hz, 3H), 2.57 (q, *J* = 7.4 Hz, 2H), 5.21 (s, 2H), 6.31 (s, 1H), 6.76 (d, *J* = 5.4 Hz, 2H), 7.03 (d, *J* = 3.2 Hz, 3H), 7.45-7.59 (m, 1H), 8.40 (d, *J* = 5.8 Hz, 2H).

^{13}C NMR (50 MHz, $CDCl_3$) δ = 12.7 (CH_3), 20.0 (CH_2), 45.5 (CH_2), 99.3 (CH), 108.9 (CH), 120.0 (CH), 120.2 (CH), 121.1 (2 x CH), 121.3 (CH), 128.3 (C), 137.1 (C), 142.6 (C), 147.3 (C), 150.3 (2 x CH).

HRMS (ESI, m/z) calcd for $C_{16}H_{17}N_2$ $[M+H]^+$ = 237.1386 found 237.1371.

g. Indolones 7a–k

- ***1-Benzyl-2-methyl-1,5,6,7-tetrahydro-4H-indol-4-one (7a)*** *[25]*

Compound **7a** was prepared according to the general procedure **G** using 2-(2-oxopropyl)cyclohex-2-en-1-one **5a** (0.59 mmol, 90 mg) and benzylamine (0.88 mmol, 94 mg) in 4.5 mL toluene. The title compound was obtained after flash chromatography as a yellow oil. Yield = 10% (14 mg). Flash chromatography conditions: column 12 g, flow rate = 18 mL/min, cyclohexane/EtOAc 100/0 to 65/35. R_f = 0.30 (cyclohexane/EtOAc 60/40).

^{1}H NMR (400 MHz, $CDCl_3$) δ = 2.08-2.13 (m, 2H), 2.14 (s, 3H), 2.48 (t, *J* = 7.4 Hz, 2H), 2.63 (t, *J* = 6.2 Hz, 2H), 5.03 (s, 2H), 6.35 (s, 1H), 6.92 (d, *J* = 7.5 Hz, 2H), 7.29-7.35 (m, 3H).

^{13}C NMR (101 MHz, $CDCl_3$) δ = 12.1 (CH_3), 22.1 (CH_2), 23.8 (CH_2), 37.8 (CH_2), 47.2 (CH_2), 103.8 (CH), 120.1 (CH), 125.7 (2 x CH), 127.6 (C), 129.0 (2 x CH), 130.7 (C), 136.7 (C), 143.8 (C), 194.1 (C).

HRMS (ESI, m/z) calcd for $C_{16}H_{18}NO$ $[M+H]^+$ = 240.1383 found 240.1409 and calcd for $C_{16}H_{17}NNaO$ $[M+Na]^+$ = 262.1202 found 262.1234.

- ***1-Benzyl-2-ethyl-1,5,6,7-tetrahydro-4H-indol-4-one (7b)*** *[26]*

Compound **7b** was prepared according to the general procedure **H** using -(2-oxobutyl)cyclohex-2-en-1-one **5b** (0.42 mmol, 70 mg) and phenylmethanamine (0.63 mmol, 67 mg) in 10 mL butanol. The title compound was obtained after flash chromatography as a yellow oil. Yield = 60% (64 mg). Flash chromatography conditions: column 12 g, flow rate = 18 mL/min, cyclohexane/EtOAc 100/0 to 65/35. R_f = 0.30 (cyclohexane/EtOAc 60/40).

^{1}H NMR (400 MHz, $CDCl_3$) δ = 1.19 (t, *J* = 7.5 Hz, 3H), 2.10 (p, *J* = 6.9 Hz, 2H), 2.41-2.46 (m, 4H), 2.62 (t, *J* = 6.3 Hz, 2H), 5.04 (s, 2H), 6.38 (s, 1H), 6.88-6.92 (m, 2H), 7.26-7.34 (m, 3H).

^{13}C NMR (101 MHz, $CDCl_3$) δ = 12.4 (CH_3), 19.4 (CH_2), 22.1 (CH_2), 23.9 (CH_2), 37.9 (CH_2), 47.1 (CH_2), 102.0 (CH), 120.1 (C), 125.7 (2 x CH), 127.7 (CH), 129.1 (2 x CH), 136.9 (C), 137.3 (C), 144.1 (C), 194.3 (C).

HRMS (ESI, m/z) calcd for $C_{17}H_{20}NO$ $[M+H]^+$ = 254.1539 found 254.1491.

- ***2-Ethyl-1-phenethyl-1,5,6,7-tetrahydro-4H-indol-4-one (7c)***

Compound **7c** was prepared according to the general procedure **G** using 2-(2-oxobutyl)cyclohex-2-en-1-one **5b** (0.54 mmol, 90 mg) and 2-phenethylamine (0.81 mmol, 98 mg) in 4 mL toluene. The title compound was obtained after flash chromatography as a yellow oil. Yield = 11% (15 mg). Flash chromatography conditions: column 12 g, flow rate = 18 mL/min, cyclohexane/EtOAc 100/0 to 65/35. R_f = 0.30 (cyclohexane/EtOAc 60/40).

^{1}H NMR (400 MHz, $CDCl_3$) δ = 1.26 (t, *J* = 7.4 Hz, 3H), 1.91-1.96 (m, 2H), 2.28 (t, *J* = 6.2 Hz , 2H), 2.35 (t, *J* = 7.1 Hz, 2H), 2.48 (q, *J* = 7.4 Hz, 2H), 2.90 (t, *J* = 7.0 Hz, 2H), 3.97 (t, *J* = 7.0 Hz, 2H), 6.32 (s, 1H), 6.97 (d, *J* = 7.7 Hz, 2H), 7.24-7.26 (m, 3H).

^{13}C NMR (101 MHz, $CDCl_3$) δ = 12.5 (CH_3), 19.4 (CH_2), 22.0 (CH_2), 23.9 (CH_2), 37.2 (CH_2), 37.8 (CH_2), 45.6 (CH_2), 102.1 (CH), 119.7 (C), 127.1 (CH), 128.9 (2 x CH), 129.0 (2 x CH), 136.5 (C), 137.8 (C), 144.1 (C), 194.3 (C).

HRMS (ESI, m/z) calcd for $C_{18}H_{22}NO$ $[M+H]^+$ = 268.1696 found 268.1676 and for $C_{18}H_{21}NNaO$ $[M+Na]^+$ = 290.1515 found 290.1491.

- ***2-Ethyl-1-(4-methoxybenzyl)-1,5,6,7-tetrahydro-4H-indol-4-one (7d)***

Compound **7d** was prepared according to the general procedure **H** using 2-(2-oxobutyl)cyclohex-2-en-1-one **5b** (0.30 mmol, 50 mg) and 4-methoxybenzylamine (0.45 mmol, 62 mg) in 9 mL butanol. The title compound was obtained after flash chromatography as a yellow oil. Yield = 48% (41 mg). Flash chromatography conditions: column 12 g, flow rate = 18 mL/min, cyclohexane/EtOAc 100/0 to 65/35. R_f = 0.30 (cyclohexane/EtOAc 60/40).

^{1}H NMR (400 MHz, $CDCl_3$) δ = 1.21 (t, *J* = 7.4 Hz, 3H), 2.11 (p, *J* = 6.2 Hz, 2H), 2.42-2.48 (m, 4H), 2.63 (t, *J* = 6.2 Hz, 2H), 3.78 (s, 3H), 4.97 (s, 2H), 6.38 (s, 1H), 6.84 (s, 4H).

^{13}C NMR (101 MHz, $CDCl_3$) δ = 12.5 (CH_3), 19.5 (CH_2), 22.2 (CH_2), 24.0 (CH_2), 37.9 (CH_2), 46.7 (CH_2), 55.4 (CH_3), 102.0 (CH), 114.5 (2 x CH), 120.1 (C), 127.0 (2 x CH), 128.9 (C), 137.3 (C), 144.1 (C), 159.2 (C), 194.3 (C).

HRMS (ESI, m/z) calcd for $C_{18}H_{22}NO_2$ $[M+H]^+$ = 284.1645 found 284.1629 and for $C_{18}H_{21}NNaO_2$ $[M+Na]^+$ = 306.1464 found 306.1442.

- ***2-Ethyl-1-(1-phenylethyl)-1,5,6,7-tetrahydro-4H-indol-4-one (7e)***

Compound **7e** was prepared according to the general procedure **G** using 2-(2-oxobutyl)cyclohex-2-en-1-one **5b** (0.54 mmol, 90 mg) and methylbenzylamine (0.81 mmol, 98 mg) in 4 mL toluene. The title compound was obtained after flash chromatography as a yellow oil. Yield = 13% (19 mg). Flash chromatography conditions: column 12 g, flow rate = 18 mL/min, cyclohexane/EtOAc 100/0 to 65/35. R_f = 0.30 (cyclohexane/EtOAc 60/40).

^{1}H NMR (400 MHz, $CDCl_3$) δ = 1.22 (t, *J* = 7.4 Hz, 3H), 1.88 (d, *J* = 7.2 Hz, 3H), 1.95-2.04 (m, 2H), 2.22-2.29 (m, 2H), 2.37-2.41 (m, 2H), 2.50-2.63 (m, 2H), 5.50 (q, *J* = 7.2 Hz, 2H), 6.38 (s, 1H), 7.04-7.07 (m, 2H), 7.28-7.36 (m, 3H).

^{13}C NMR (101 MHz, $CDCl_3$) δ = 13.2 (CH_3), 19.7 (CH_3), 20.7 (CH_2), 24.1 (CH_2), 24.5 (CH_2), 38.1 (CH_2), 53.2 (CH), 102.6 (CH), 121.0 (2 x CH), 126.3 (C), 127.9 (2 x CH), 129.2 (CH), 137.7 (C), 141.2 (C), 144.0 (C), 194.7 (C).

HRMS (ESI, m/z) calcd for $C_{18}H_{22}NO$ $[M+H]^+$ = 268.1696 found 268.1699 and for $C_{18}H_{21}NNaO$ $[M+Na]^+$ = 290.1515 found 290.1519.

- ***2-Ethyl-1-(furan-2-ylmethyl)-1,5,6,7-tetrahydro-4H-indol-4-one (7f)*** *[26]*

Compound **7f** was prepared according to the general procedure **G** using 2-(2-oxobutyl)cyclohex-2-en-1-one **5b** (0.54 mmol, 90 mg) and 3-furylmethylamine (0.81 mmol,

79 mg) in 4 mL toluene. The title compound was obtained after flash chromatography as a yellow oil. Yield = 13% (17 mg). Flash chromatography conditions: column 12 g, flow rate = 18 mL/min, cyclohexane/EtOAc 100/0 to 65/35. R_f = 0.30 (cyclohexane/EtOAc 60/40).

^{1}H NMR (300 MHz, $CDCl_3$) δ = 1.25 (t, *J* = 7.4 Hz, 3H), 2.14 (p, *J* = 6.3 Hz, 2H), 2.43-2.47 (m, 2H), 2.55-2.63 (m, 2H), 2.80 (t, *J* = 6.2 Hz, 2H), 4.93 (s, 2H), 6.10 (dd, *J* = 3.3, 0.9 Hz , 1H), 6.30-6.32 (m, 2H), 7.35 (dd, *J* = 1.9, 0.8 Hz, 1H).

^{13}C NMR (75 MHz, $CDCl_3$) δ = 12.3 (CH_3), 19.4 (CH_2), 22.1 (CH_2), 23.8 (CH_2), 37.8 (CH_2), 40.8 (CH_2), 101.8 (CH), 107.9 (CH), 110.6 (CH), 120.1 (C), 137.0 (C), 142.9 (CH), 144.0 (C), 149.8 (C), 194.2 (C).

HRMS (ESI, m/z) calcd for $C_{15}H_{18}NO_2$ $[M+H]^+$ = 244.1332 found 244.1347 and for $C_{15}H_{17}NNaO_2$ $[M+Na]^+$ = 266.1151 found 266.1164.

- ***1-(4-Bromobenzyl)-2-methyl-1,5,6,7-tetrahydro-4H-indol-4-one (7g)***

Compound **7g** was prepared according to the general procedure **H** using -(2-oxopropyl)cyclohex-2-en-1-one **5a** (0.33 mmol, 50 mg) and (4-bromophenyl)methanamine (0.49 mmol, 91 mg) in 8 mL butanol . The title compound was obtained after flash chromatography as a yellow oil. Yield = 51% (54 mg). Flash chromatography conditions: column 12 g, flow rate = 18 mL/min, cyclohexane/EtOAc 100/0 to 65/35. R_f = 0.30 (cyclohexane/EtOAc 60/40).

^{1}H NMR (400 MHz, $CDCl_3$) δ = 2.08-2.13 (m, 2H), 2.12 (s, 3H), 2.45 (t, *J* = 6.9 Hz, 2H), 2.61 (t, *J* = 6.2 Hz, 2H), 4.97 (s, 2H), 6.34 (s, 1H), 6.79 (d, *J* = 8.2 Hz, 2H), 7.45 (d, *J* = 8.4 Hz, 2H).

^{13}C NMR (101 MHz, $CDCl_3$) δ = 12.2 (CH_3), 22.2 (CH_2), 24.0 (CH_2), 38.0 (CH_2), 46.8 (CH_2), 104.1 (CH), 120.4 (C), 121.7 (C), 127.5 (2 x CH), 130.7 (C), 132.3 (2 x CH), 135.9 (C), 143.7 (C), 194.1 (C).

HRMS (ESI, m/z) calcd for $C_{16}H_{16}BrNNaO$ $[M+Na]^+$ = 340.0307 found 340.0297.

- ***1-(Furan-2-ylmethyl)-2-methyl-1,5,6,7-tetrahydro-4H-indol-4-one (7h)***

Compound **7h** was prepared according to the general procedure **H** using -(2-oxopropyl)cyclohex-2-en-1-one **5a** (0.33 mmol, 50 mg) and furan-2-ylmethanamine (0.49 mmol, 47 mg) in 8 mL butanol. The title compound was obtained after flash chromatography

as a yellow oil. Yield = 56% (42 mg). Flash chromatography conditions: column 12 g, flow rate = 18 mL/min, cyclohexane/EtOAc 100/0 to 65/35. R_f = 0.30 (cyclohexane/EtOAc 60/40).

^{1}H NMR (400 MHz, $CDCl_3$) δ = 2.14 (p, *J* = 6.3 Hz, 2H), 2.26 (s, 3H), 2.44 (t, *J* = 6.2 Hz, 2H), 2.79 (t, *J* = 6.2 Hz, 2H), 4.92 (s, 2H), 6.12 (d, *J* = 3.0 Hz, 1H), 6.27 (s, 1H), 6.31 (dd, *J* = 3.3, 1.9 Hz, 1H), 7.35 (d, *J* = 1.6 Hz, 1H).

^{13}C NMR (101 MHz, $CDCl_3$) δ = 12.2 (CH_3), 22.1 (CH_2), 23.8 (CH_2), 37.9 (CH_2), 40.9 (CH_2), 103.7 (CH), 107.9 (CH), 111.0 (CH), 120.1 (C), 130.6 (C), 142.9 (CH), 143.9 (C), 149.8 (C), 194.1 (C).

HRMS (ESI, m/z) calcd for $C_{14}H_{16}NO_2$ $[M+H]^+$ = 230.1176 found 230.1201 and for $C_{14}H_{15}NNaO_2$ $[M+Na]^+$ = 252.0995 found 252.0984.

- ***1-(Pyridin-4-ylmethyl)-2-methyl-1,5,6,7-tetrahydro-4H-indol-4-one (7i)***

Compound **7i** was prepared according to the general procedure **H** using 2-(2-oxopropyl)cyclohex-2-en-1-one **5a** (0.59 mmol, 90 mg) and 4-(aminomethyl)pyridine (0.89 mmol, 96 mg) in 15 mL butanol. The title compound was obtained after flash chromatography as a yellow oil. Yield = 51% (72 mg). Flash chromatography conditions: column 24 g, flow rate = 20 mL/min, cyclohexane/EtOAc 100/0 to 0/100. R_f = 0.11 (EtOAc 100%).

^{1}H NMR (400 MHz, $CDCl_3$) δ = 2.15 (s, 3H), 2.19-2.11 (m, 2H), 2.49 (t, *J* = 6.2 Hz, 2H), 2.62 (t, *J* = 6.2 Hz, 2H), 5.05 (s, 2H), 6.40 (s, 1H), 6.86 (d, *J* = 5.5 Hz, 2H), 8.59 (d, *J* = 5.7 Hz, 2H).

^{13}C NMR (101 MHz, $CDCl_3$) δ = 11.8 (CH_3), 22.0 (CH_2), 23.7 (CH_2), 37.7 (CH_2), 46.2 (CH_2), 104.4 (CH), 120.56 (2 x CH), 120.59 (C), 130.3 (C), 143.2 (C), 145.9 (C), 150.5 (2 x CH), 193.6 (C).

HRMS (APCI, m/z) calcd for $C_{15}H_{17}N_2O$ $[M+H]^+$ = 241.1335 found 241.1344.

- ***2-Methyl-1-phenethyl-1,5,6,7-tetrahydro-4H-indol-4-one (7j)***

Compound **7j** was prepared according to the general procedure **H** using 2-(2-oxopropyl)cyclohex-2-en-1-one **5a** (0.33 mmol, 50 mg) and 2-phenylethan-1-amine (0.49 mmol, 59 mg) in 8 mL butanol. The title compound was obtained after flash chromatography as a yellow oil. Yield = 50% (42 mg). Flash chromatography conditions: column 12 g, flow rate = 18 mL/min, cyclohexane/EtOAc 100/0 to 65/35. R_f = 0.30 (cyclohexane/EtOAc 60/40).

^{1}H NMR (400 MHz, $CDCl_3$) δ = 1.93 (p, *J* = 6.3 Hz ,2H), 2.16 (s, 3H), 2.27 (t, *J* = 6.2 Hz, 2H), 2.36 (t, *J* = 6.2 Hz, 2H), 2.91 (t, *J* = 6.9 Hz, 2H), 3.97 (t, *J* = 6.9 Hz, 2H), 6.27 (s, 1H), 6.96 (dd, *J* = 7.3, 2.2 Hz, 2H), 7.21-7.29 (m, 3H).

^{13}C NMR (101 MHz, $CDCl_3$) δ = 12.1 (CH_3), 22.0 (CH_2), 23.8 (CH_2), 37.1 (CH_2), 37.8 (CH_2), 45.7 (CH_2), 103.9 (CH), 119.8 (C), 127.1 (CH), 128.8 (2 x CH), 129.0 (2 x CH), 130.1 (C), 137.7 (C), 143.9 (C), 194.1 (C).

HRMS (ESI, m/z) calcd for $C_{17}H_{20}NO$ $[M+H]^+$ = 254.1539 found 254.1544 and for $C_{17}H_{19}NNaO$ $[M+Na]^+$ = 276.1359 found 276.1335.

- ***1-Phenethyl-2-phenyl-1,5,6,7-tetrahydro-4H-indol-4-one (7k)*** *[26]*

Compound **7k** was prepared according to the general procedure **H** using 2-(2-oxo-2-phenylethyl)cyclohex-2-en-1-one **5f** (0.23 mmol, 50 mg) and 2-phenylethan-1-amine (0.34 mmol, 41 mg) in 7 mL butanol. The title compound was obtained after flash chromatography as a yellow oil. Yield = 54% (40 mg). Flash chromatography conditions: column 12 g, flow rate = 18 mL/min, cyclohexane/EtOAc 100/0 to 65/35. R_f = 0.30 (cyclohexane/EtOAc 60/40). Although this compound was reported in the literature it was not fully characterized and we completed its characterization.

^{1}H NMR (400 MHz, $CDCl_3$) δ = 2.00 (p, *J* = 6.3 Hz, 2H), 2.37-2.46 (m, 4H), 2.68 (t, *J* = 7.1 Hz, 2H), 4.11 (t, *J* = 7.1 Hz, 2H), 6.58 (s, 1H), 6.82 (dd, *J* = 6.5, 3.0 Hz, 2H), 7.18-7.22 (m, 3H), 7.35-7.47 (m, 5H).

^{13}C NMR (101 MHz, $CDCl_3$) δ = 22.3 (CH_2), 23.8 (CH_2), 37.0 (CH_2), 38.0 (CH_2), 46.2 (CH_2), 106.2 (CH), 120.3 (C), 127.0 (CH), 128.0 (CH), 128.7 (2 x CH), 128.8 (2 x CH), 128.9 (2 x CH), 129.4 (2 x CH), 132.8 (C), 135.7 (C), 137.6 (C), 146.1 (C), 194.4 (C).

HRMS (ESI, m/z) calcd for $C_{22}H_{22}NO$ $[M+H]^+$ = 316.1696 found 316.1719 and for $C_{22}H_{21}NNaO$ $[M+Na]^+$ = 338.1515 found 338.1502.

h. Cinnolines 8a–k

- ***3-Methyl-5,6,7,8-tetrahydrocinnoline (8a)*** *[27]*

Compound **8a** was prepared according to the general procedure **I** using 2-(2-oxopropyl)cyclohex-2-en-1-one **5a** (1 mmol, 152 mg). The title compound was obtained after flash chromatography as a yellow oil. Yield = 82% (121 mg). Flash chromatography conditions: column 12 g, flow rate= 20 mL/min, cyclohexane/EtOAc 100/0 to 50/50. R_f = 0.20 (EtOAc 100%).

^{1}H NMR (300 MHz, $CDCl_3$) δ = 1.79-1.86 (m, 2H), 1.88-1.96 (m, 2H), 2.61 (s, 3H), 2.75 (t, *J* = 6.1 Hz, 2H), 3.09 (t, *J*= 6.4 Hz, 2H), 7.00 (s, 1H).

^{13}C NMR (75 MHz, $CDCl_3$) δ = 21.7 (CH_3), 21.9 (CH_2), 22.6 (CH_2), 27.9 (CH_2), 29.7 (CH_2), 126.6 (CH), 137.1 (C), 157.2 (C), 158.3 (C).

HRMS (ESI, m/z) calcd for $C_9H_{13}N_2$ $[M+H]^+$ = 149.1073 found 149.1105 for $C_9H_{12}N_2Na$ $[M+Na]^+$ = 171.0893 found 171.0913.

- ***3-Ethyl-5,6,7,8-tetrahydrocinnoline (8b)***

Compound **8b** was prepared according to the general procedure **I** using 2-(2-oxobutyl)cyclohex-2-en-1-one **5b** (1 mmol, 166 mg). The title compound was obtained after flash chromatography as a yellow oil. Yield = 90% (146 mg). Flash chromatography conditions: column 12 g, flow rate= 20 mL/min, cyclohexane/EtOAc 100/0 to 50/50. R_f = 0.20 (EtOAc 100%).

^{1}H NMR (300 MHz, $CDCl_3$) δ = 1.23 (t, *J* = 6 Hz, 3H), 1.67-1.75 (m, 2H), 1.78-1.86 (m, 2H), 2.67 (t, *J* = 6.3 Hz, 2H), 2.82 (q, *J* = 7.6 Hz, 2H), 2.99 (t, *J* = 6.4 Hz, 2H), 6.90 (s, 1H).

^{13}C NMR (75 MHz, $CDCl_3$) δ = 14.3 (CH_3), 22.3 (CH_2), 23.0 (CH_2), 28.4 (CH_2), 29.3 (CH_2), 30.1 (CH_2), 125.8 (CH), 137.5 (C), 158.8 (C), 162.9 (C).

HRMS (APCI, m/z) calcd for $C_{10}H_{15}N_2$ $[M+H]^+$ = 163.1230 found 163.1268.

- ***3,6,6-Trimethyl-5,6,7,8-tetrahydrocinnoline (8c)***

Compound **8c** was prepared according to the general procedure **I** using 4,4-dimethyl-2-(2-oxopropyl)cyclohex-2-en-1-one **5c** (1 mmol, 176 mg). The title compound was obtained after flash chromatography as a yellow oil. Yield = 86% (151 mg). Flash chromatography

conditions: column 12 g, flow rate= 20 mL/min, cyclohexane/EtOAc 100/0 to 50/50. R_f = 0.20 (EtOAc 100%).

^{1}H NMR (400 MHz, $CDCl_3$) δ = 0.97 (s, 6H), 1.68 (t, *J* = 6.9 Hz, 2H), 2.47 (s, 2H), 2.58 (s, 3H), 3.09 (t, *J* = 6.9 Hz, 2H), 6.93 (s, 1H).

^{13}C NMR (101 MHz, $CDCl_3$) δ = 21.8 (CH_3), 26.6 (CH_2), 27.9 (2 x CH_3), 29.2 (C), 35.3 (CH), 42.0 (CH_2), 127.2 (CH), 136.5 (C), 157.3 (C), 157.5 (C).

HRMS (ESI, m/z) calcd for $C_{11}H_{17}N_2$ $[M+H]^+$ = 177.1386 found 177.1418.

- ***3-Ethyl-6,6-dimethyl-5,6,7,8-tetrahydrocinnoline (8d)***

Compound **8d** was prepared according to the general procedure **I** using 4,4-dimethyl-2-(2-oxobutyl)cyclohex-2-en-1-one **5d** (1 mmol, 190 mg). The title compound was obtained after flash chromatography as a yellow solid. Yield = 92% (175 mg). mp 38 °C. Flash chromatography conditions: column 12 g, flow rate= 20 mL/min, cyclohexane/EtOAc 100/0 to 50/50. R_f = 0.20 (EtOAc 100%).

^{1}H NMR (400 MHz, $CDCl_3$) δ = 0.99 (s, 6H), 1.31 (t, *J* = 7.6 Hz, 3H), 1.70 (t, *J* = 6.9 Hz, 2H), 2.50 (s, 2H), 2.90 (q, *J* = 7.6 Hz, 2H), 3.12 (t, *J* = 6.9 Hz, 2H), 6.94 (s, 1H).

^{13}C NMR (101 MHz, $CDCl_3$) δ = 14.0 (CH_3), 26.7 (CH_2), 27.9 (2 x CH_3), 29.0 (CH_2), 29.2 (C), 35.4 (CH_2), 42.1 (CH_2), 126.0 (CH), 136.5 (C), 158.0 (C), 162.2 (C).

HRMS (ESI, m/z) calcd for $C_{12}H_{19}N_2$ $[M+H]^+$ = 191.1543 found 191.1583.

- ***3-(tert-Butyl)-5,6,7,8-tetrahydrocinnoline (8e)***

Compound **8e** was prepared according to the general procedure **I** using 2-(3,3-dimethyl-2-oxobutyl)cyclohex-2-en-1-one **5e** (1 mmol, 194 mg). The title compound was obtained after flash chromatography as a white solid. Yield = 77% (146 mg). mp 102 °C. Flash chromatography conditions: column 12 g, flow rate= 20 mL/min, cyclohexane/EtOAc 100/0 to 50/50. R_f = 0.20 (EtOAc 100%).

^{1}H NMR (400 MHz, $CDCl_3$) δ = 1.37 (s, 9H), 1.70-1.81 (m, 2H), 1.82-1.94 (m, 2H), 2.72 (t, *J* = 6.4 Hz, 2H), 3.05 (t, *J* = 6.5 Hz, 2H), 7.09 (s, 1H).

^{13}C NMR (101 MHz, $CDCl_3$) δ = 22.4 (CH_2), 23.0 (CH_2), 28.6 (CH_2), 30.1 (CH_2), 30.4 (3 × CH_3), 36.7 (C), 123.0 (CH), 137.0 (C), 158.4 (C), 167.9 (C).

HRMS (ESI, m/z) calcd for $C_{12}H_{19}N_2$ $[M+H]^+$ = 191.1543 found 191.1533 and for $C_{12}H_{18}N_2Na$ $[M+Na]^+$ = 213.1362 found 213.1350.

- ***3-Phenyl-5,6,7,8-tetrahydrocinnoline (8f)*** *[28]*

Compound **8f** was prepared according to the general procedure **I** using 2-(2-oxo-2-phenylethyl)cyclohex-2-en-1-one **5f** (1 mmol, 214 mg). The title compound was obtained after flash chromatography as a yellow solid. Yield = 81% (170 mg). mp 86 °C. Flash chromatography conditions: column 12 g, flow rate= 20 mL/min, cyclohexane/EtOAc 100/0 to 50/50. R_f = 0.20 (EtOAc 100%).

^{1}H NMR (400 MHz, $CDCl_3$) δ = 1.81-1.90 (m, 2H), 1.91-2.01 (m, 2H), 2.84 (t, *J* = 6.3 Hz, 2H), 3.17 (t, *J* = 6.4 Hz, 2H), 7.41-7.51 (m, 4H), 8.00-8.06 (m, 2H).

^{13}C NMR (101 MHz, $CDCl_3$) δ = 22.2 (CH_2), 22.8 (CH_2), 28.5 (CH_2), 30.1 (CH_2), 124.0 (CH), 127.1 (2 x CH), 129.1 (2 x CH), 129.7 (CH), 137.0 (C), 137.8 (C), 157.2 (C), 159.9 (C).

HRMS (ESI, m/z) calcd for $C_{14}H_{15}N_2$ $[M+H]^+$ = 211.1230 found 211.1240 and for $C_{14}H_{14}N_2Na$ $[M+Na]^+$ = 233.1049 found 233.1045.

- ***3-([1,1'-Biphenyl]-4-yl)-5,6,7,8-tetrahydrocinnoline (8g)***

Compound **8g** was prepared according to the general procedure **I** using 2-(2-([1,1'-biphenyl]-4-yl)-2-oxoethyl)cyclohex-2-en-1-one **5g** (1 mmol, 290 mg). The title compound was obtained after flash chromatography as a yellow solid. Yield = 86% (246 mg). mp 145 °C. Flash chromatography conditions: column 12 g, flow rate= 20 mL/min, cyclohexane/EtOAc 100/0 to 50/50. R_f = 0.20 (EtOAc 100%).

^{1}H NMR (400 MHz, $CDCl_3$) δ = 1.82-1.86 (m, 2H), 1.91-1.97 (m, 2H), 2.82 (t, *J* = 6.4 Hz, 2H), 3.16 (t, *J* = 6.4 Hz, 2H), 7.36 (dd, *J* = 7.3, 7.3 Hz, 1H), 7.42-7.50 (m, 3H), 7.64 (d, *J* = 8.4 Hz, 2H), 7.71 (d, *J* = 8.4 Hz, 2H), 8.11 (d, *J* = 8.3 Hz, 2H).

^{13}C NMR (101 MHz, $CDCl_3$) δ = 22.0 (CH_2), 22.6 (CH_2), 28.3 (CH_2), 29.9 (CH_2), 123.4 (CH), 127.1 (2 x CH), 127.3 (2 x CH), 127.5 (2 x CH), 127.7 (CH), 128.9 (2 x CH), 136.7 (C), 137.4 (C), 140.4 (C), 142.2 (C), 156.9 (C), 159.3 (C).

HRMS (ESI, m/z) calcd for $C_{20}H_{19}N_2$ $[M+H]^+$ = 287.1543 found 287.1559 and for $C_{20}H_{18}N_2Na$ $[M+H]^+$ = 309.1362 found 309.1335.

- ***3-(4-Iodophenyl)-5,6,7,8-tetrahydrocinnoline (8h)***

Compound **8g** was prepared according to the general procedure **I** using 2-(2-(4-iodophenyl)-2-oxoethyl)cyclohex-2-en-1-one **5h** (1 mmol, 340 mg). The title compound was obtained after flash chromatography as a yellow solid. Yield = 82% (275.5 mg). mp 155 °C. Flash chromatography conditions: column 12 g, flow rate= 20 mL/min, cyclohexane/EtOAc 100/0 to 50/50. R_f = 0.20 (EtOAc 100%).

^{1}H NMR (400 MHz, $CDCl_3$) δ = 1.82-1.91 (m, 2H), 1.93-2.01 (m, 2H), 2.85 (t, *J* = 6.6 Hz, 2H), 3.17 (t, *J* = 6.4 Hz, 2H), 7.47 (s, 1H), 7.73-7.90 (m, 4H).

^{13}C NMR (101 MHz, $CDCl_3$) δ = 22.01 (CH_2), 22.6 (CH_2), 28.4 (CH_2), 30.0 (CH_2), 96.1 (C), 123.4 (CH), 128.7 (2 x CH), 136.4 (C), 137.7 (C), 138.2 (2 x CH), 156.2 (C), 159.8 (C).

HRMS (ESI, m/z) calcd for $C_{14}H_{14}IN_2$ $[M+H]^+$ = 337.0196 found 337.0235 and for $C_{14}H_{13}IN_2Na$ $[M+Na]^+$ = 359.0016 found 358.9992.

- ***3-(4-Fluorophenyl)-5,6,7,8-tetrahydrocinnoline (8i)***

Compound **8i** was prepared according to the general procedure **I** using 2-(2-(4-fluorophenyl)-2-oxoethyl)cyclohex-2-en-1-one **5i** (1 mmol, 232 mg). The title compound was obtained after flash chromatography as a yellow solid. Yield = 92% (210 mg). mp 130 °C. Flash chromatography conditions: column 12 g, flow rate= 20 mL/min, cyclohexane/EtOAc 100/0 to 50/50. R_f = 0.20 (EtOAc 100%).

^{1}H NMR (400 MHz, $CDCl_3$) δ = 1.77-1.89 (m, 2H), 1.90-2.01 (m, 2H), 2.82 (t, *J* = 6.6 Hz, 2H), 3.15 (t, *J* = 6.4 Hz, 2H), 7.15 (dd, *J* = 8.7, 8.7 Hz, 2H), 7.43 (s, 1H), 8.01(dd, *J* = 8.9, 5.4 Hz, 2H).

^{13}C NMR (101 MHz, $CDCl_3$) δ = 22.0 (CH_2), 22.6 (CH_2), 28.4 (CH_2), 29.9 (CH_2), 115.9 (d, J_{C-H} = 21.7 Hz, 2 x CH), 123.4 (CH), 128.8 (d, J_{C-H} = 8.4 Hz, 2 x CH), 133.1 (d, J_{C-H} = 3.1 Hz, C), 137.6 (C), 156.1 (C), 159.4 (C), 163.9 (d, J_{C-H} = 249.1 Hz, C).

^{19}F NMR (376 MHz, $CDCl_3$) δ = -112.3-112.2 (m).

HRMS (ESI, m/z) calcd for $C_{14}H_{14}FN_2$ $[M+H]^+$ = 229.1136 found 229.1173.

- ***3-(Thiophen-2-yl)-5,6,7,8-tetrahydrocinnoline (8j)***

Compound **8j** was prepared according to the general procedure **I** using 2-(2-oxo-2-(thiophen-2-yl)ethyl)cyclohex-2-en-1-one **5j** (1 mmol, 220 mg). The title compound was obtained after

flash chromatography as a yellow solid. Yield = 87% (188 mg). mp 172 °C. Flash chromatography conditions: column 12 g, flow rate= 20 mL/min, cyclohexane/EtOAc 100/0 to 50/50. R_f = 0.20 (EtOAc 100%).

^{1}H NMR (400 MHz, $CDCl_3$) δ = 1.81-1.87 (m, 2H), 1.90-1.98 (m, 2H), 2.81 (t, *J* = 6.3 Hz, 2H), 3.12 (t, *J* = 6.4 Hz, 2H), 7.12(dd, *J* = 5.0, 3.7 Hz, 1H), 7.40 (s, 1H), 7.42 (dd, *J* = 5.3, 1.1 Hz, 1H), 7.59 (dd, *J* = 3.7, 1.2 Hz, 1H).

^{13}C NMR (101 MHz, $CDCl_3$) δ = 22.4 (CH_2), 22.9 (CH_2), 28.7 (CH_2), 30.3 (CH_2), 122.3 (CH), 125.7 (CH), 128.3 (CH), 128.8 (CH), 137.8 (C), 141.7 (C), 153.2 (C), 159.7 (C).

HRMS (ESI, m/z) calcd for $C_{12}H_{12}N_2NaS$ $[M+H]^+$ = 239.0613 found 239.0610.

- ***3-(Pyridin-2-yl)-5,6,7,8-tetrahydrocinnoline (8k)***

Compound **8k** was prepared according to the general procedure **I** using 2-(2-oxo-2-(pyridin-2-yl)ethyl)cyclohex-2-en-1-one **5k** (1 mmol, 284 mg). The title compound was obtained after flash chromatography as a yellow solid. Yield = 94% (198 mg). mp 91 °C. Flash chromatography conditions: column 12 g, flow rate= 20 mL/min, cyclohexane/EtOAc 100/0 to 50/50. R_f = 0.20 (EtOAc 100%).

^{1}H NMR (400 MHz, $CDCl_3$) δ = 1.78-1.89 (m, 2H), 1.89-2.00 (m, 2H), 2.87 (t, *J* = 6.4 Hz, 2H), 3.18 (t, *J* = 6.4 Hz, 2H), 7.33 (ddd, *J* = 7.5, 4.8, 1.2 Hz, 1H), 7.83 (ddd, *J* = 7.7, 7.7, 1.8 Hz, 1H), 8.17 (s, 1H), 8.63 (d, *J* = 7.9, 1H), 8.66 (m, 1H).

^{13}C NMR (101 MHz, $CDCl_3$) δ = 22.1 (CH_2), 22.6 (CH_2), 28.3 (CH_2), 30.1 (CH_2), 121.5 (CH), 124.1 (CH), 124.3 (CH), 137.2 (CH), 137.9 (C), 149.3 (CH), 154.3 (C), 156.3 (C), 160.7 (C).

HRMS (ESI, m/z) calcd for $C_{13}H_{14}N_3$ $[M+H]^+$ = 212.1182 found 212.1204 and for $C_{13}H_{13}N_3Na$ $[M+Na]^+$ = 234.1002 found 234.1005.

i. 2-Ethyl-1-(3-(2-ethyl-1*H*-indol-1-yl)propyl)-1,5,6,7-tetrahydro-4*H*-indol-4-one 9

- ***2-Ethyl-1-(3-(2-ethyl-1H-indol-1-yl)propyl)-1,5,6,7-tetrahydro-4H-indol-4-one (9)***

Compound **9** was prepared according to the adapted general procedure **G** using 2-(2-oxobutyl)cyclohex-2-en-1-one **5b** (0.91 mmol, 1 equiv, 152 mg) and 1,3-diaminopropane (0.45 mmol, 0.5 equiv, 34 mg) in 8 mL toluene. The title compound was obtained after flash chromatography as a yellow oil. Yield = 13% (20 mg). Flash chromatography conditions:

column 12 g, flow rate = 18 mL/min, cyclohexane/EtOAc 100/0 to 65/35. R_f = 0.30 (cyclohexane/EtOAc 60/40).

^{1}H NMR (400 MHz, $CDCl_3$) δ = 1.19 (t, *J* = 7.4 Hz, 3H), 1.37 (t, *J* = 7.4 Hz, 3H), 2.04-2.08 (m, 2H), 2.09-2.17 (m, 2H), 2.37 (q, *J* = 8.4 Hz, 2H), 2.42-2.47 (m, 2H), 2.50 (t, *J* = 6.2 Hz, 2H), 2.70 (q, *J* = 7.4 Hz, 2H), 3.73 (t, *J* = 7.2 Hz, 2H), 4.12 (t, *J* = 7.2 Hz, 2H), 6.31 (s, 1H), 7.05-7.09 (m, 1H), 7.12-7.15 (m, 2H), 7.26 (s, 1H), 7.55 (d, *J* = 7.6 Hz, 1H).

^{13}C NMR (101 MHz, $CDCl_3$) δ = 12.5 (CH_3), 12.8 (CH_3), 19.4 (CH_2), 20.1 (CH_2), 22.0 (CH_2), 23.9 (CH_2), 31.2 (CH_2), 37.7 (CH_2), 40.0 (CH_2), 41.2 (CH_2), 90.1 (CH), 102.1 (CH), 108.7 (CH), 119.7 (CH), 120.1 (C), 120.3 (CH), 121.1 (CH), 128.4 (C), 136.62 (C), 136.64 (C), 136.7 (C), 142.1 (C), 194.1 (C).

HRMS (ESI, m/z) calcd for $C_{23}H_{29}N_2O$ $[M+H]^+$ = 349.2274 found 349.2237.

V. NMR spectra

a. 2-(Acetyloxymethyl)-4,4-dimethylcyclohex-2-en-1-one (2b).

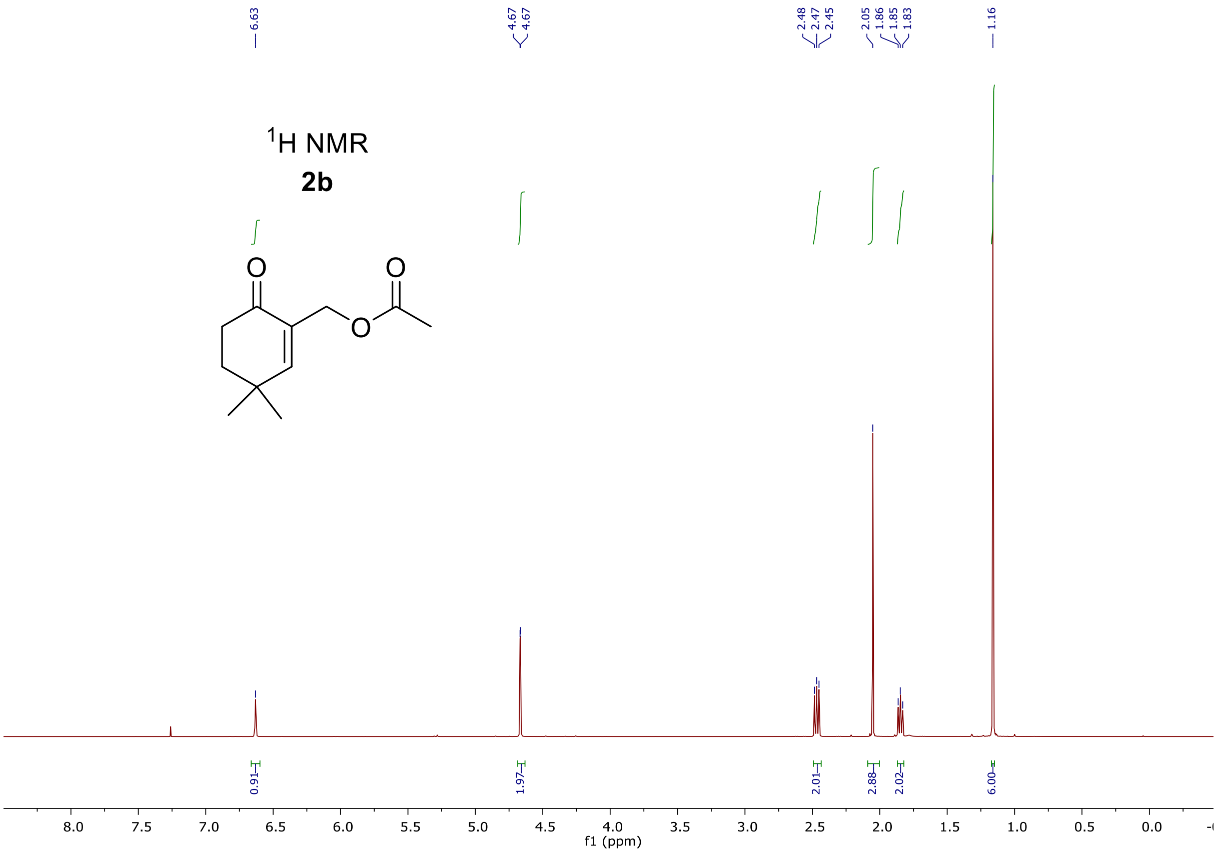

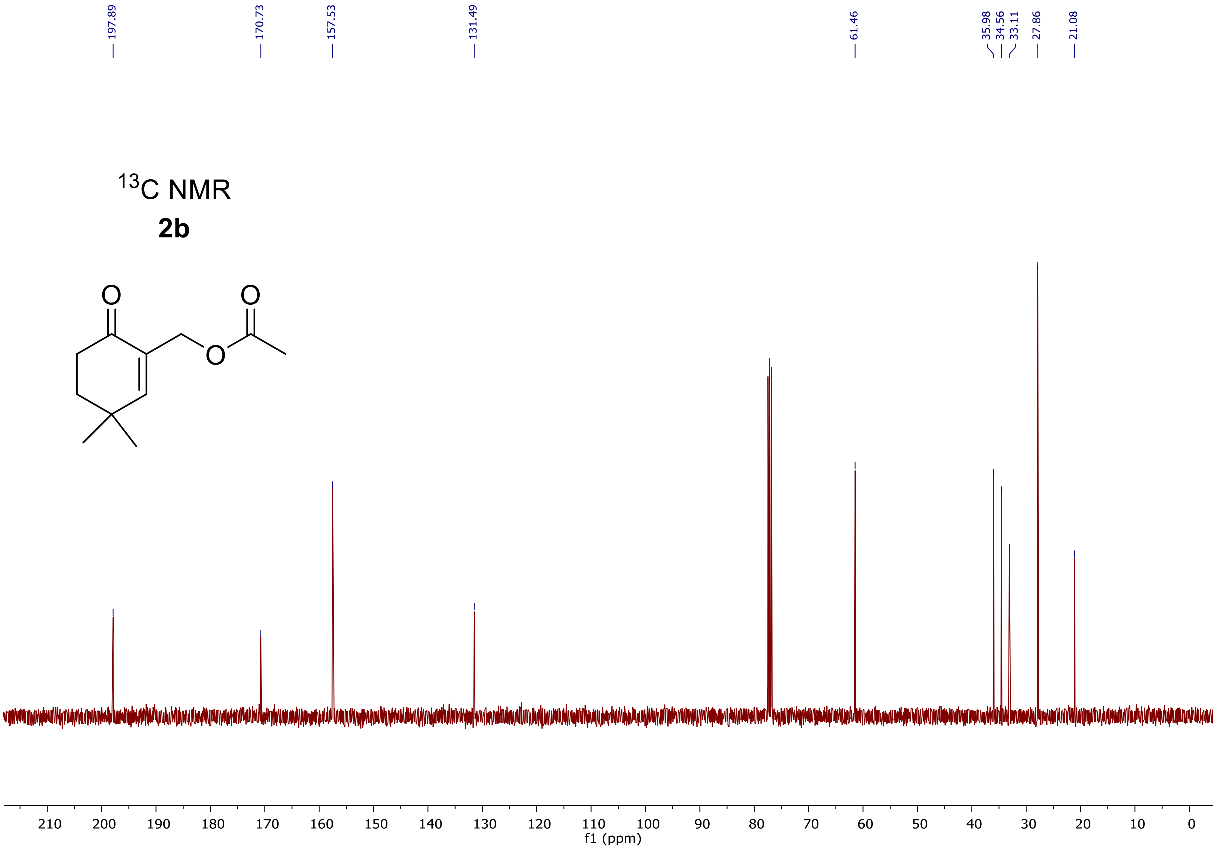

b. Nitroalkylcyclohex-2-en-1-ones 3b–d.

- ***2-(2-Nitrobutyl)cyclohex-2-en-1-one (3b)***

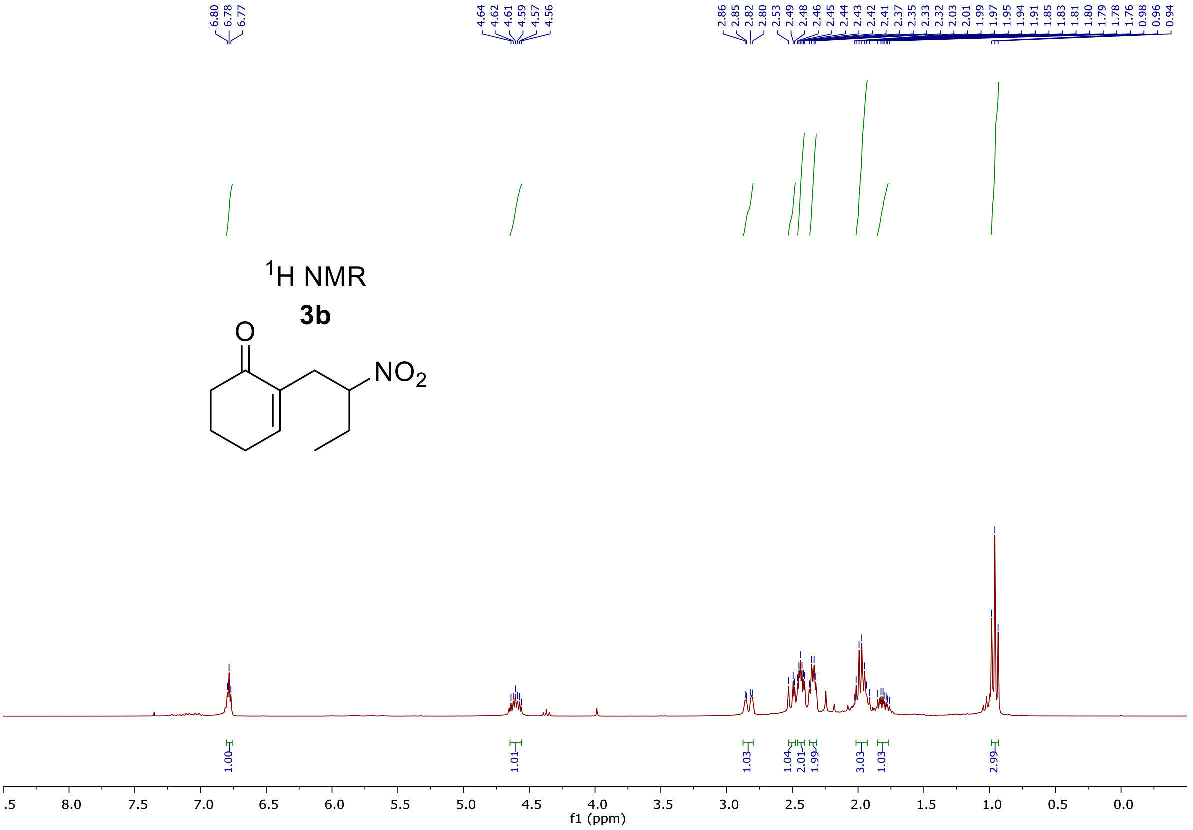

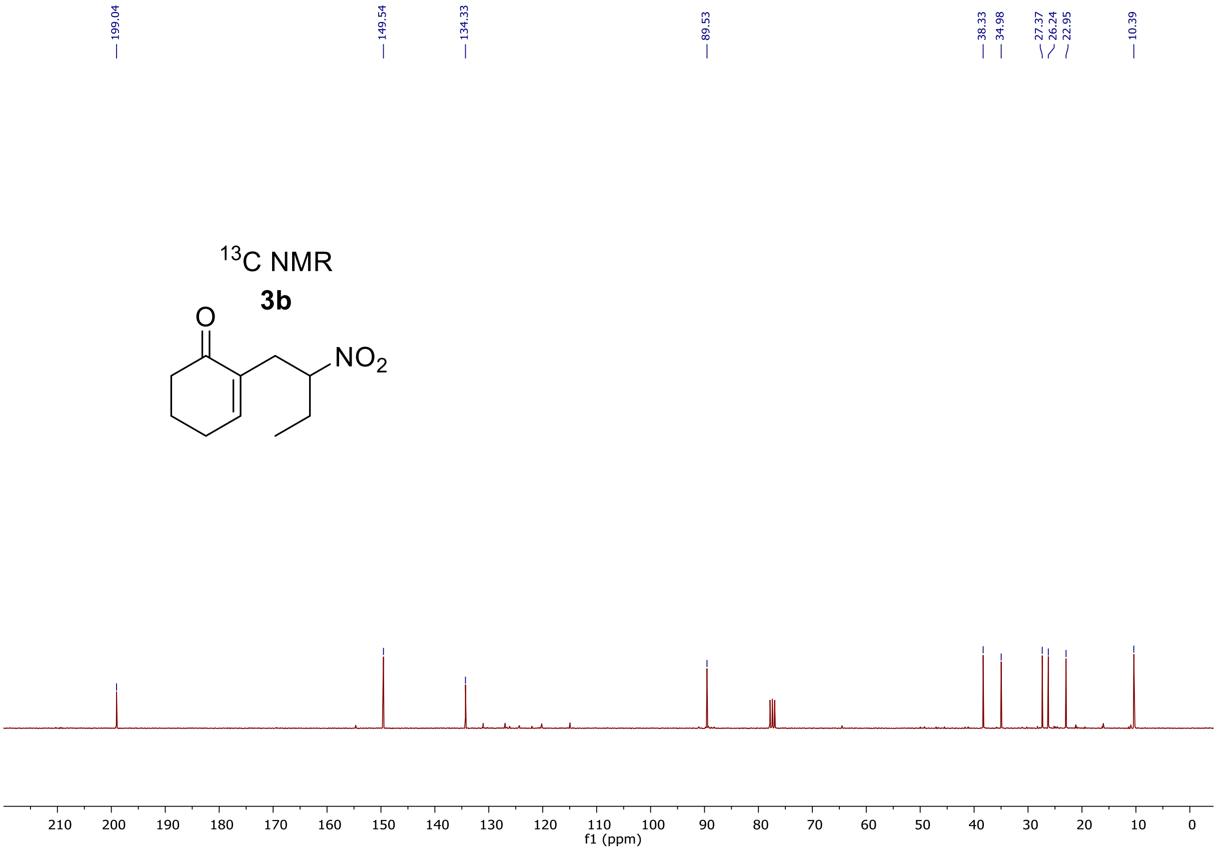

- ***4,4-Dimethyl-2-(2-nitropropyl)cyclohex-2-en-1-one (3c)***

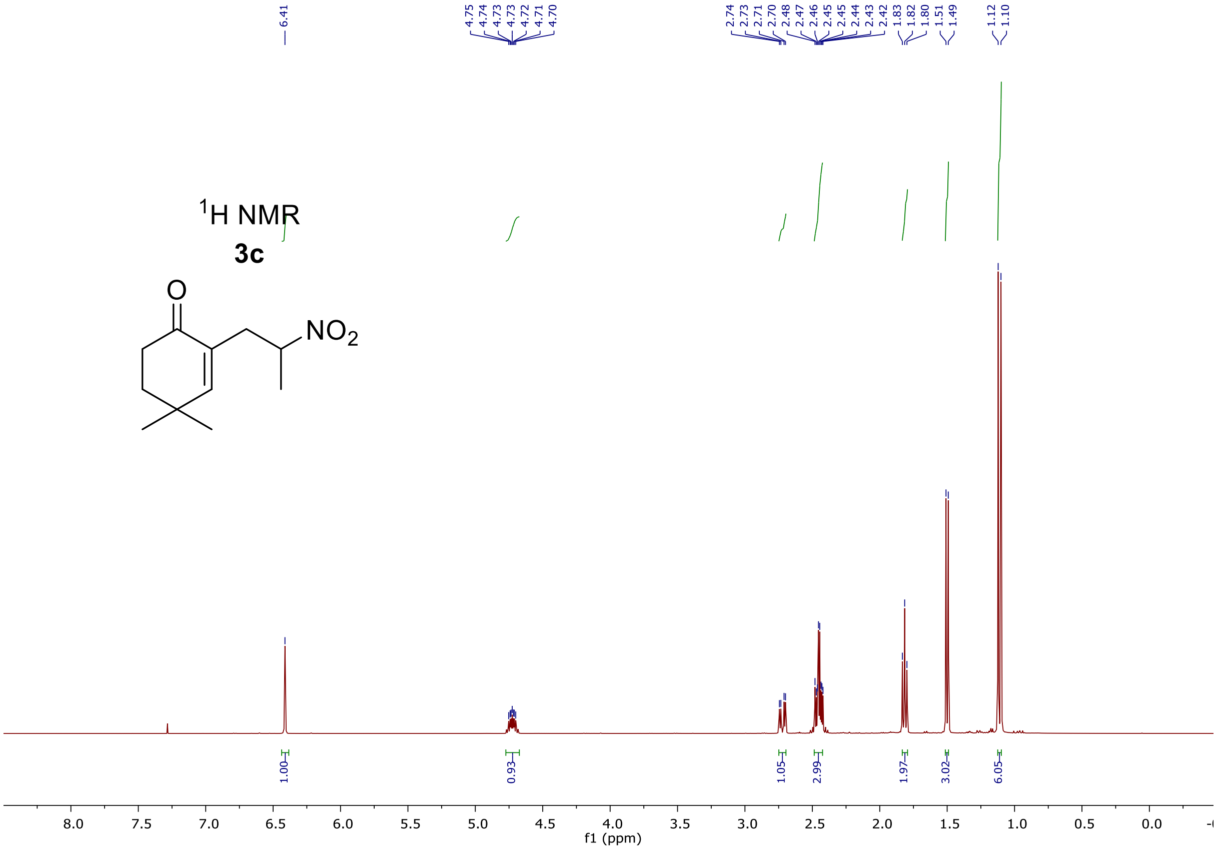

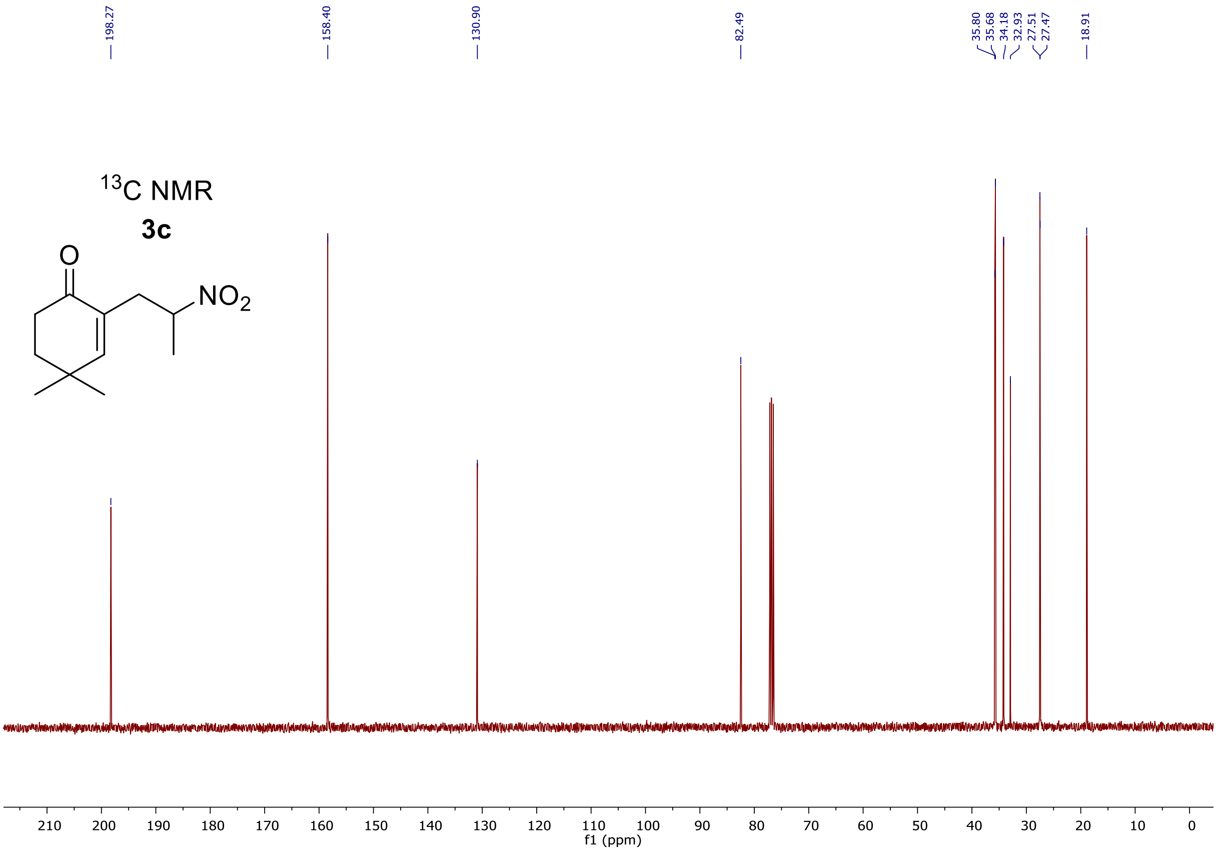

- ***4,4-Dimethyl-2-(2-nitrobutyl)cyclohex-2-en-1-one (3d)***

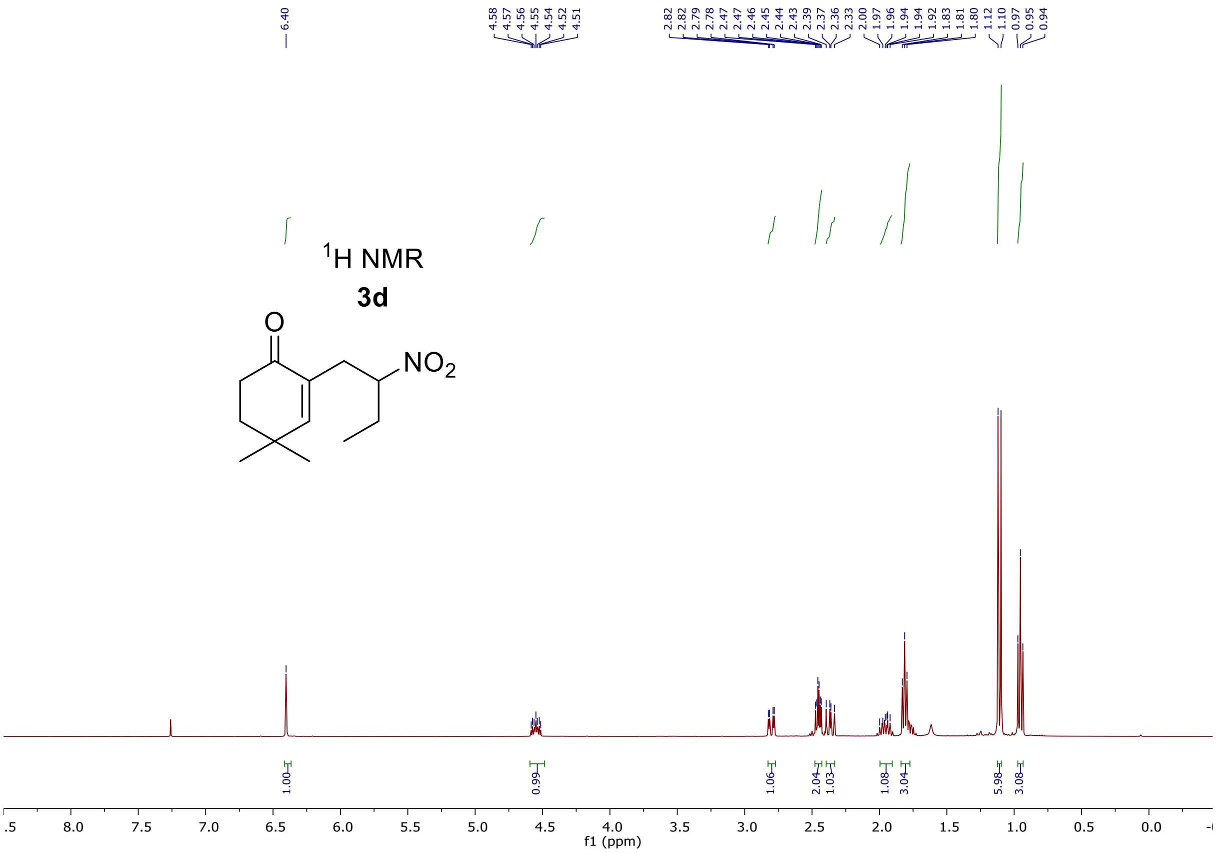

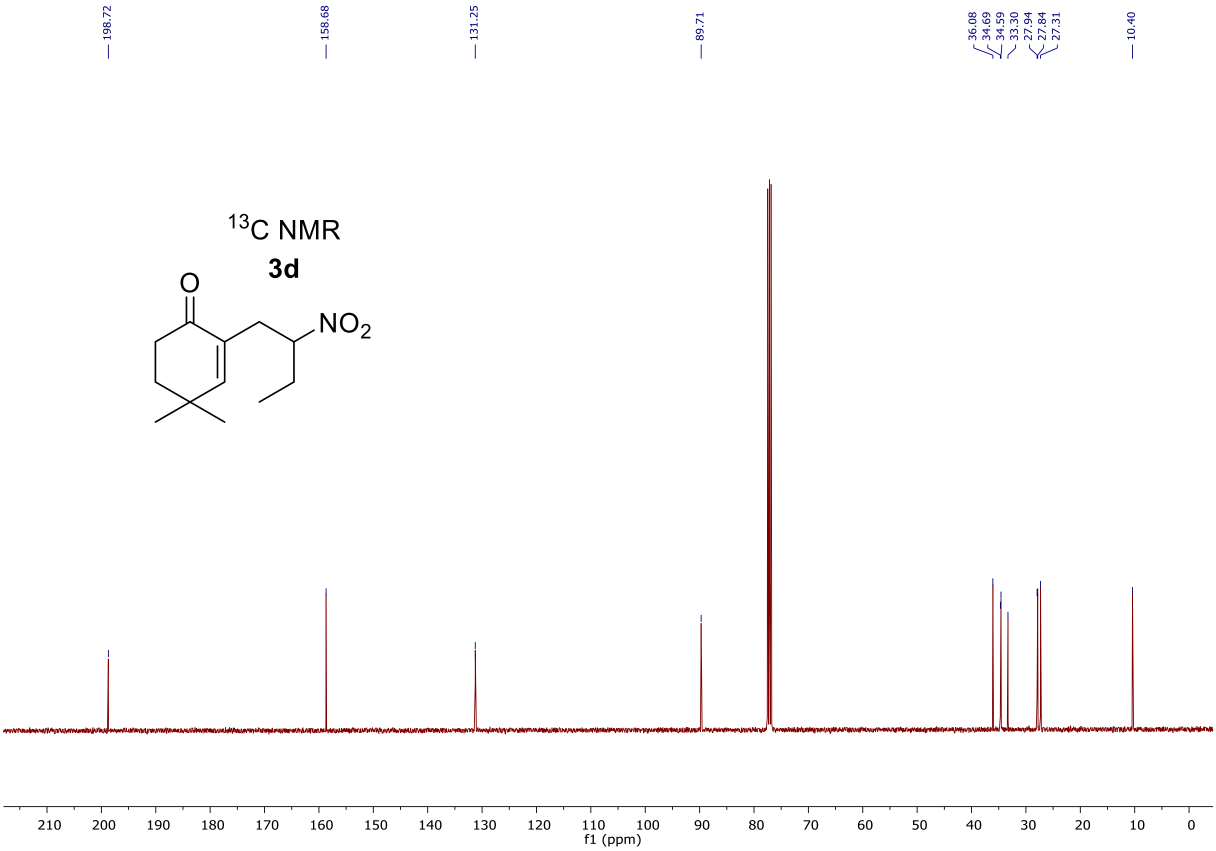

c. 1,4-Dicetones 5a–k.

- ***2-(2-Oxopropyl)cyclohex-2-en-1-one (5a)***

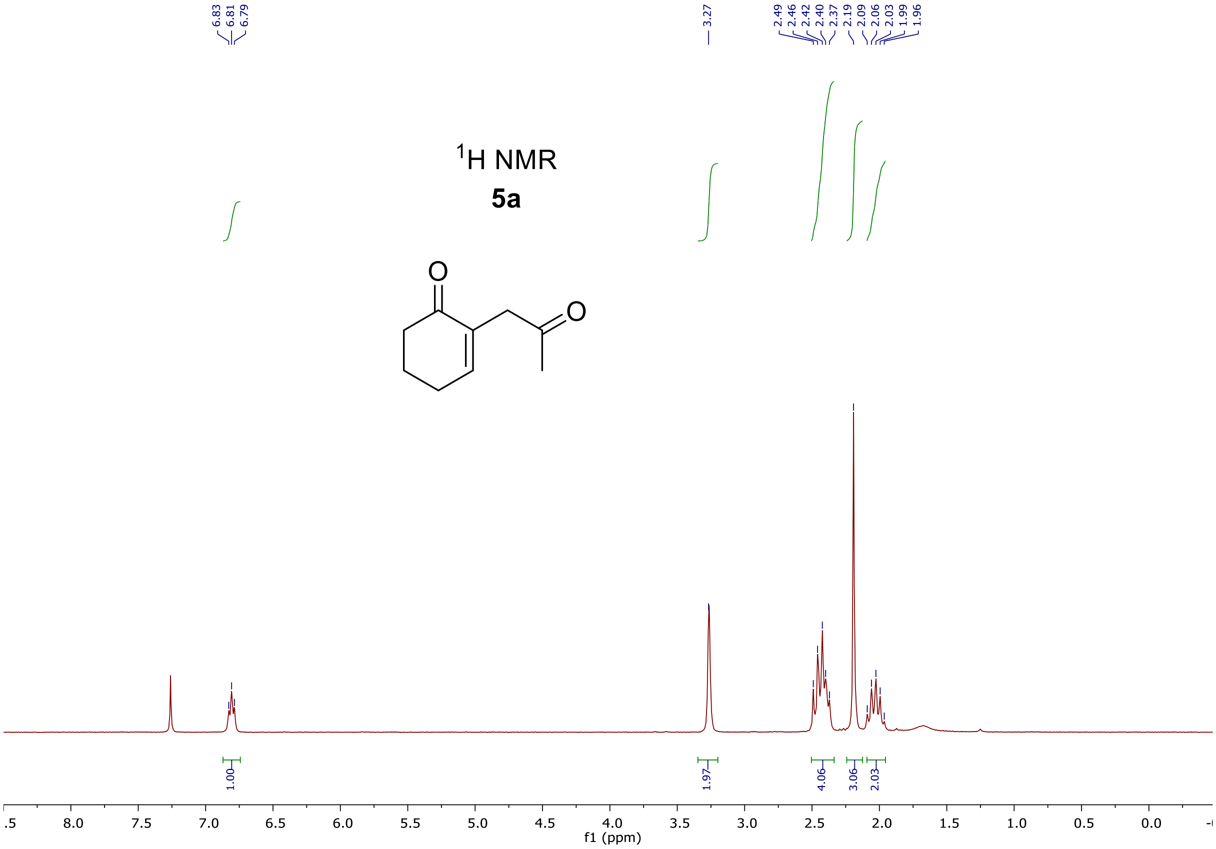

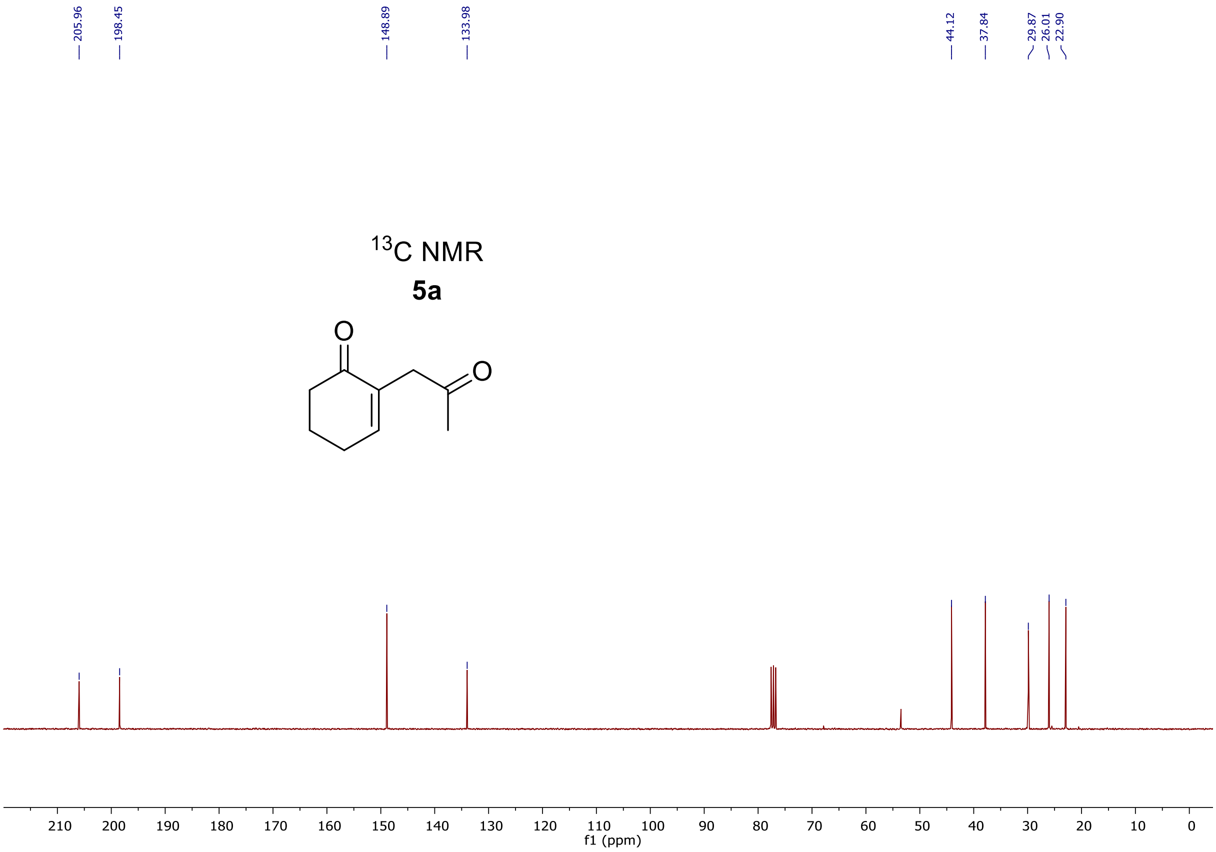

- ***2-(2-Oxobutyl)cyclohex-2-en-1-one (5b)***

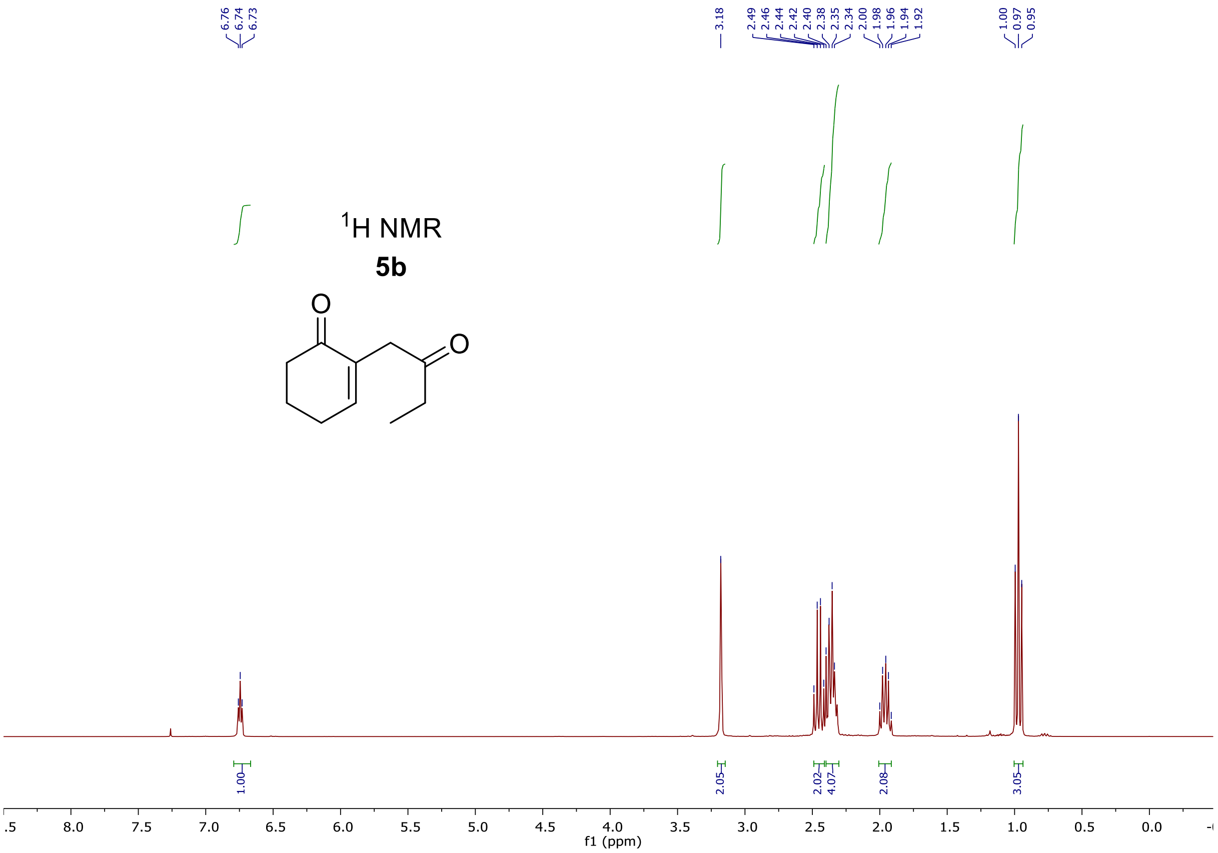

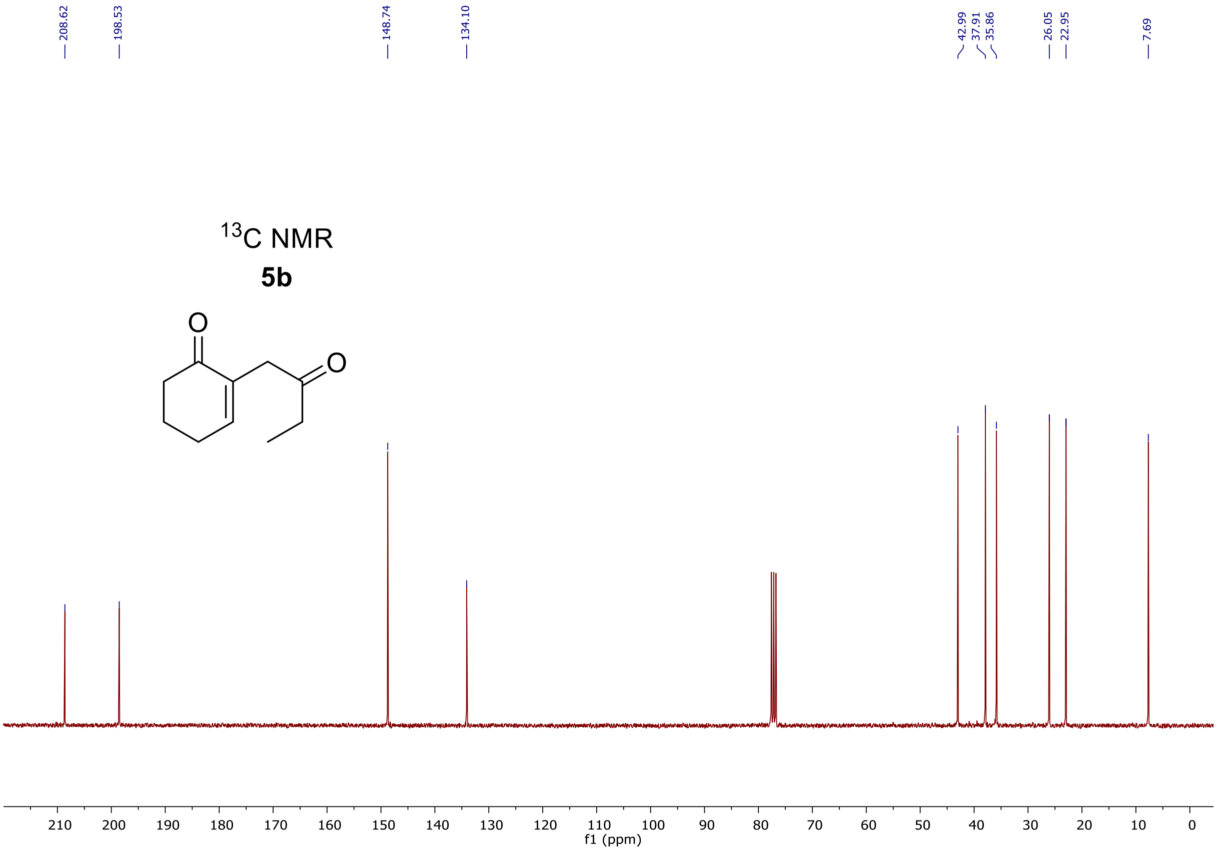

- ***4,4-Dimethyl-2-(2-oxopropyl)cyclohex-2-en-1-one (5c)***

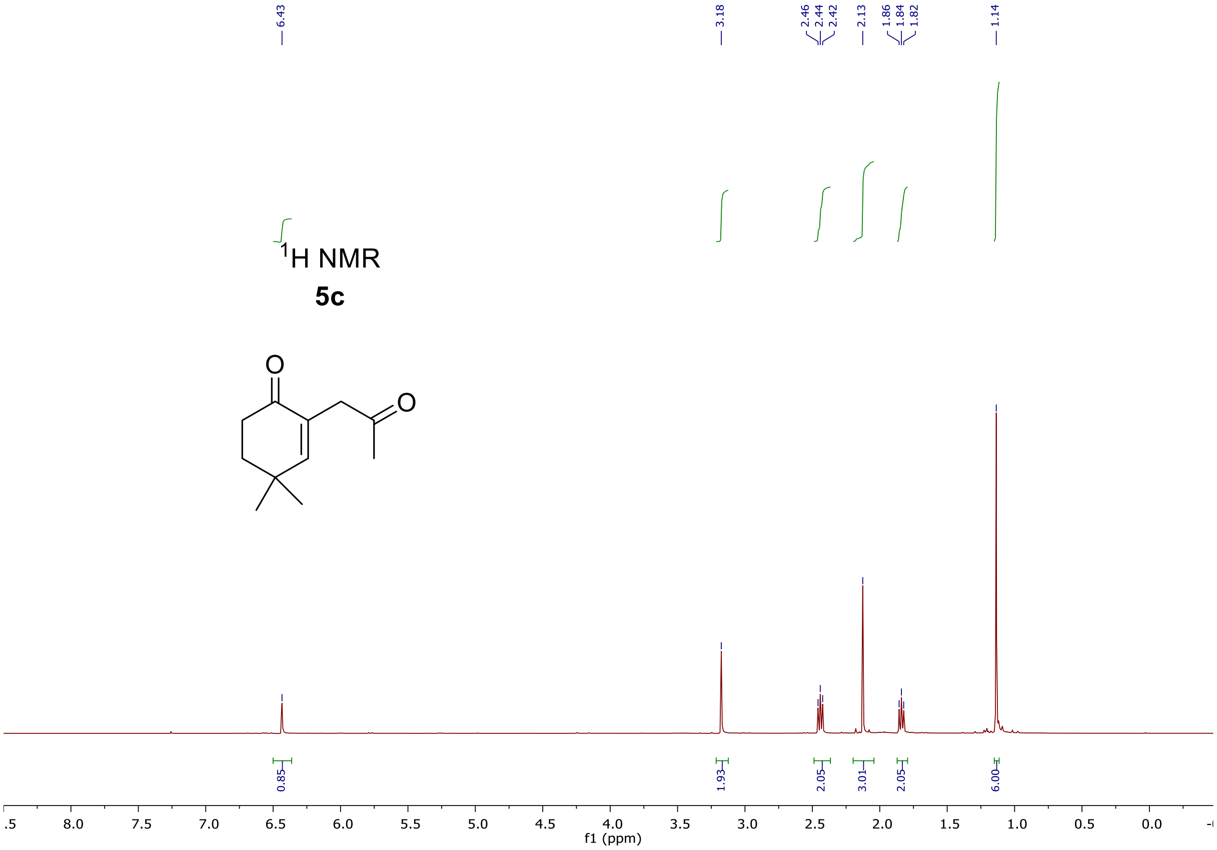

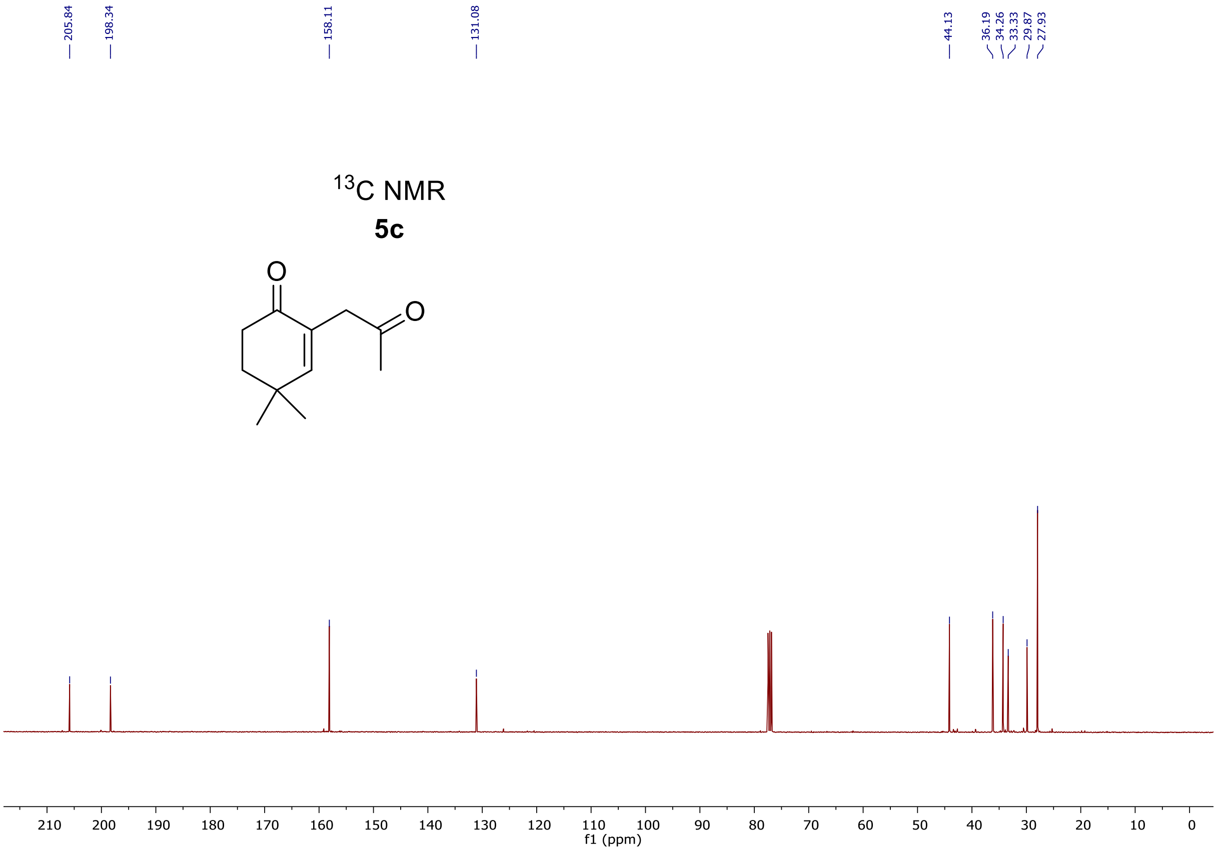

- ***4,4-Dimethyl-2-(2-oxobutyl)cyclohex-2-en-1-one (5d)***

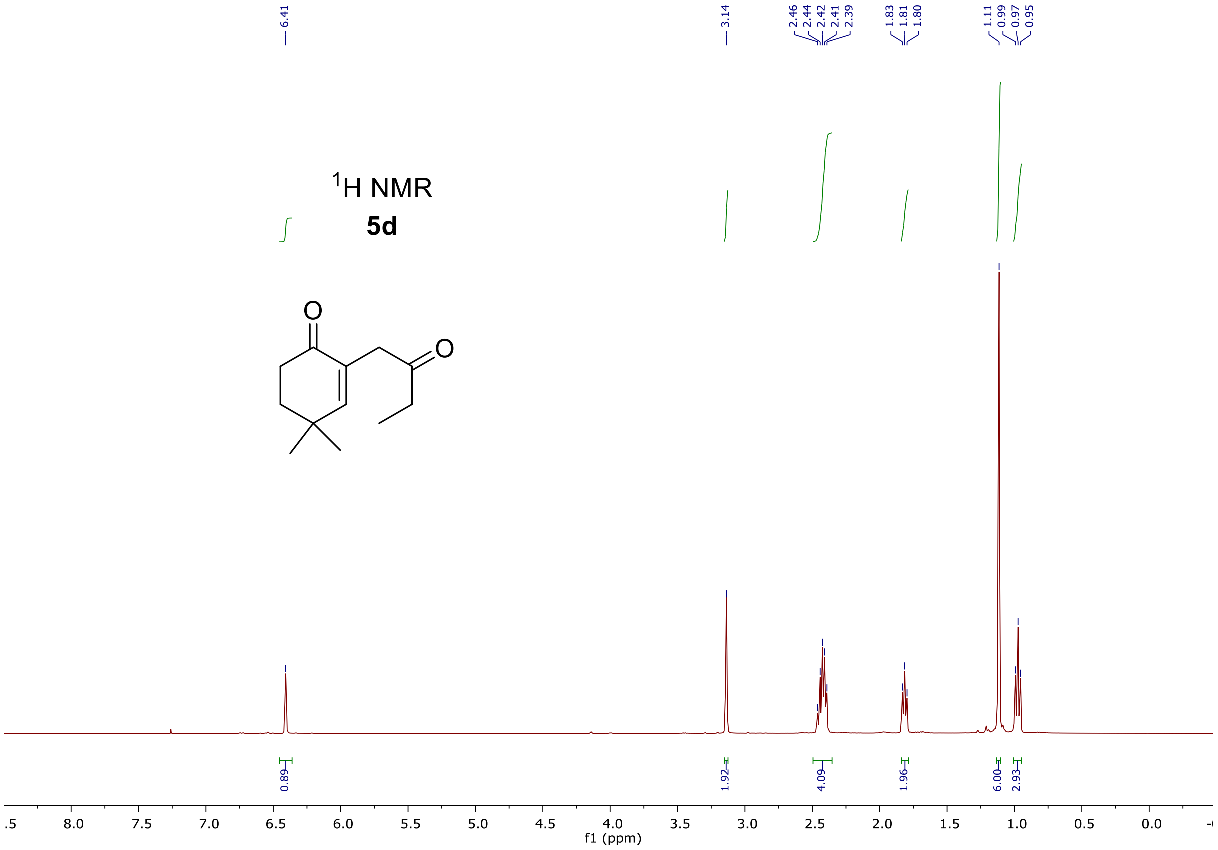

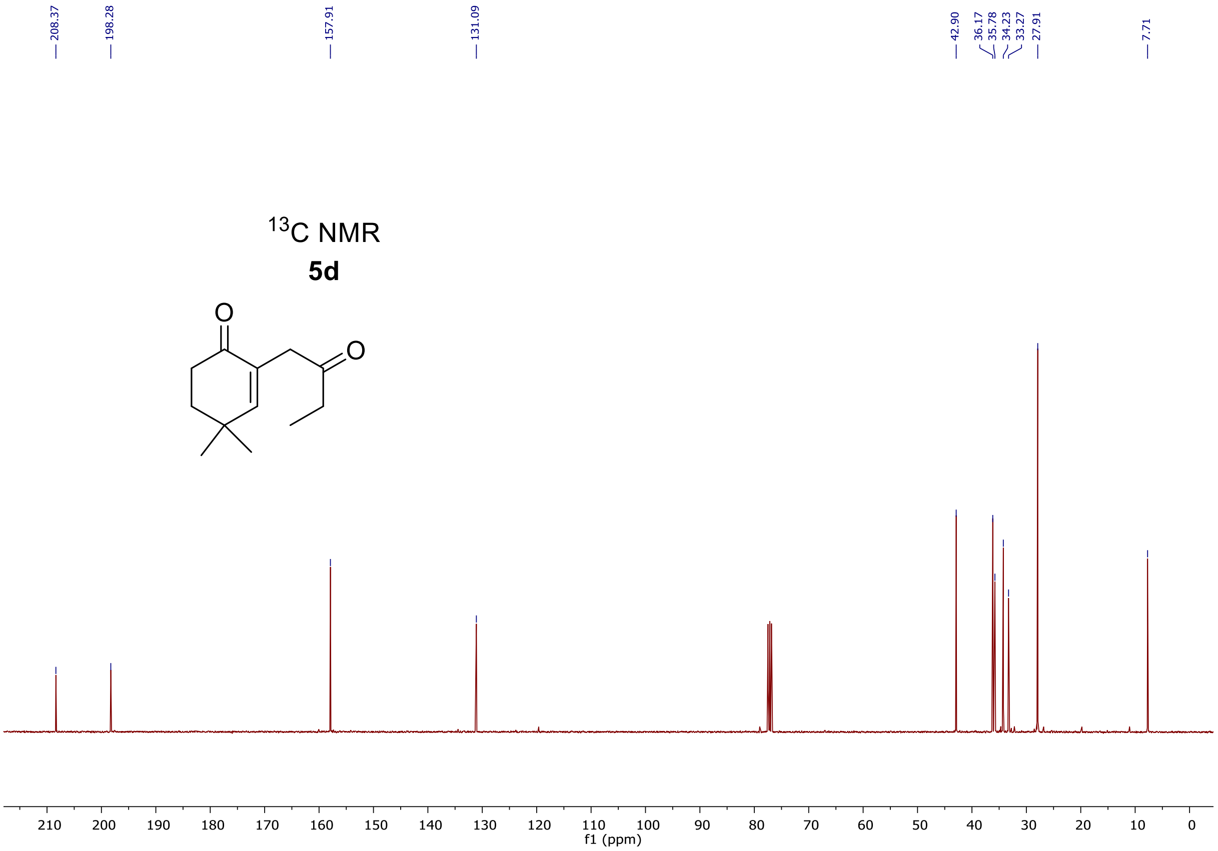

- ***2-(3,3-Dimethyl-2-oxobutyl)cyclohex-2-en-1-one (5e)***

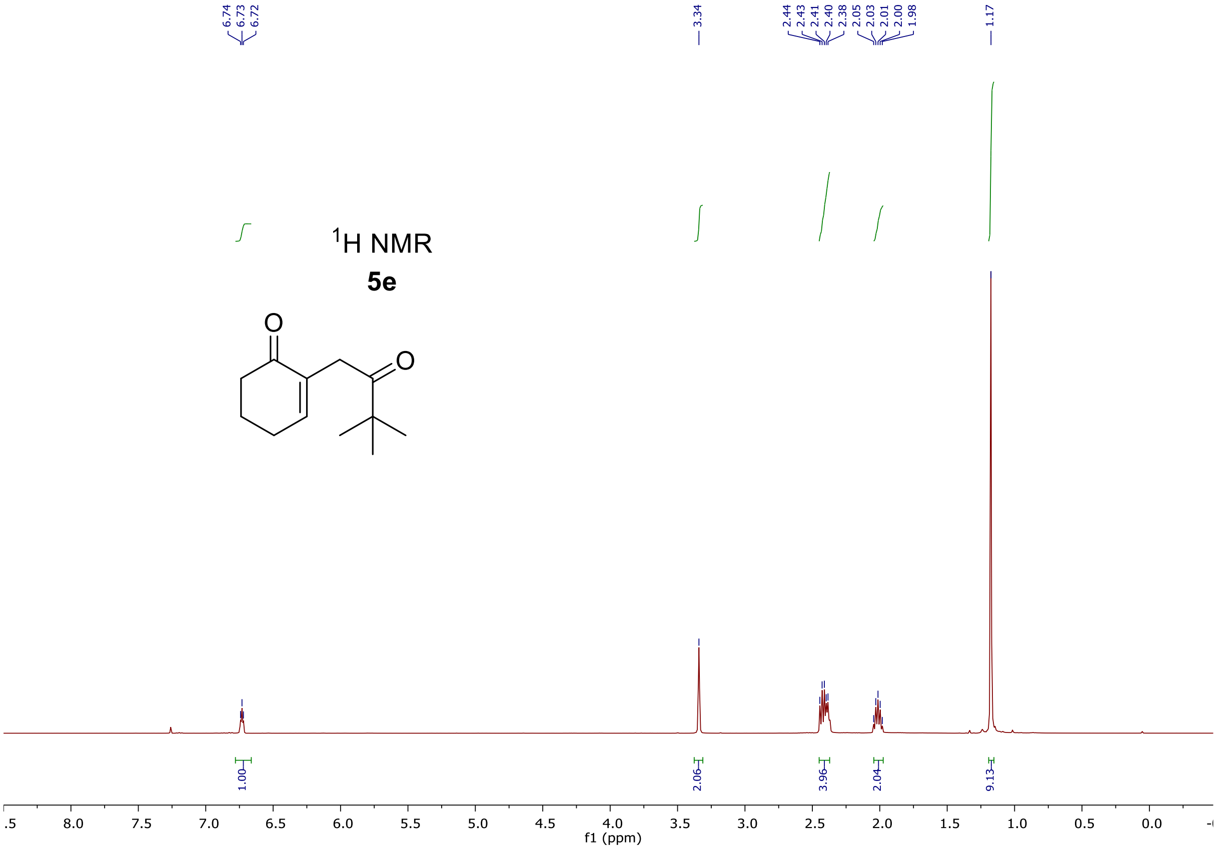

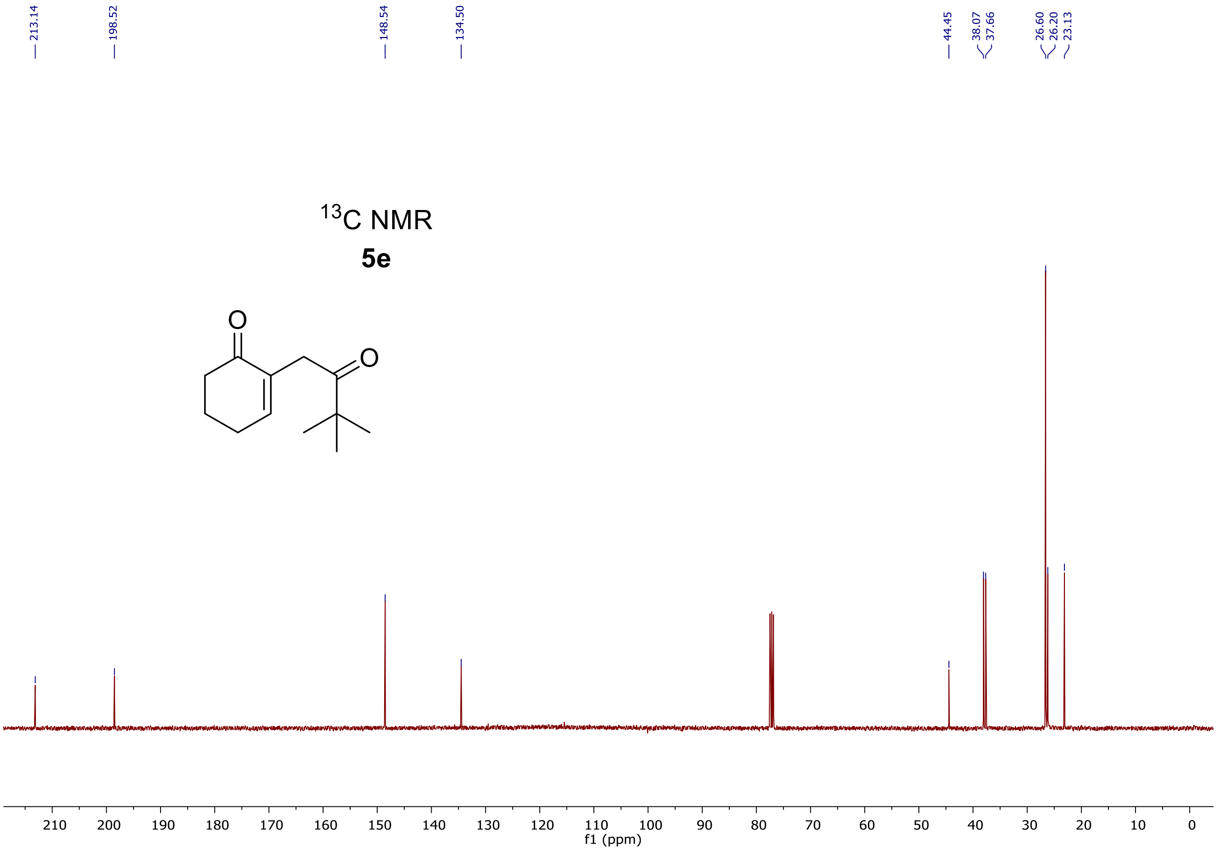

- ***2-(2-Oxo-2-phenylethyl)cyclohex-2-en-1-one (5f)***

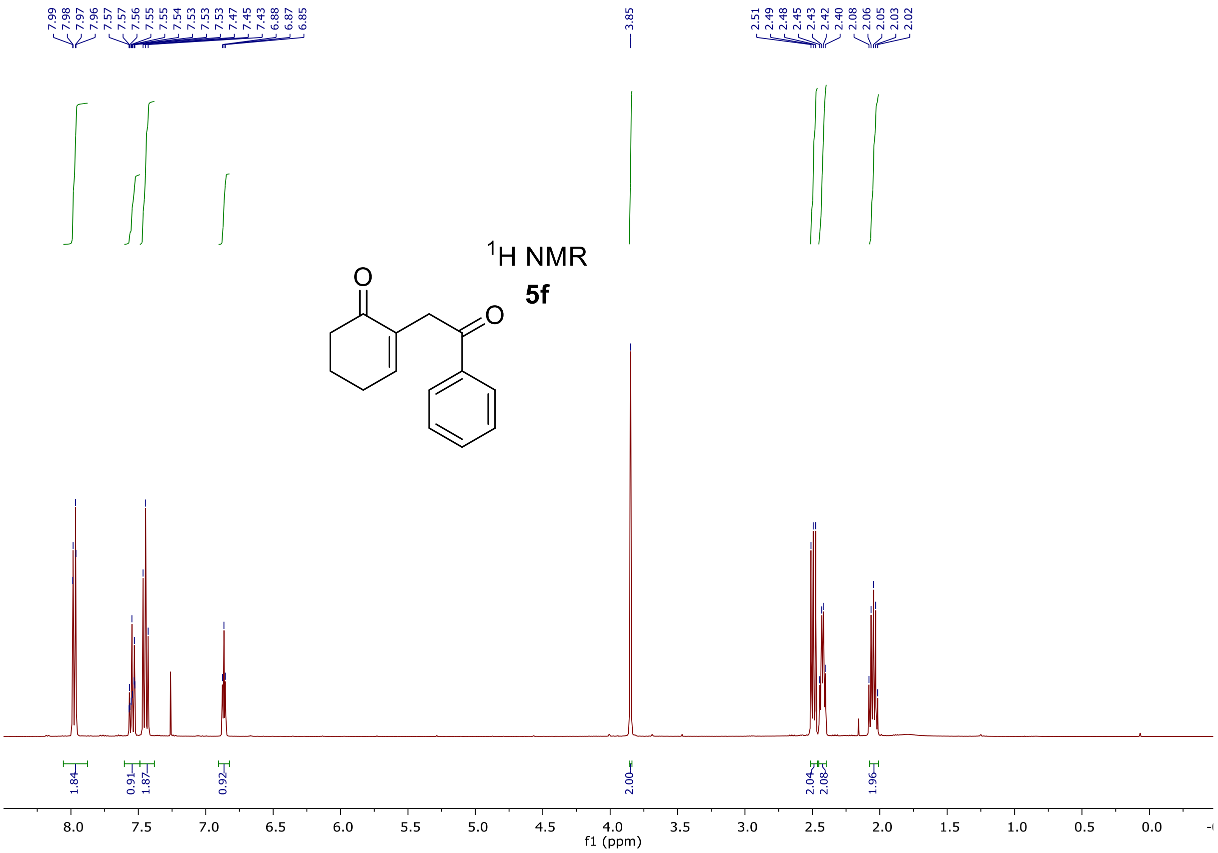

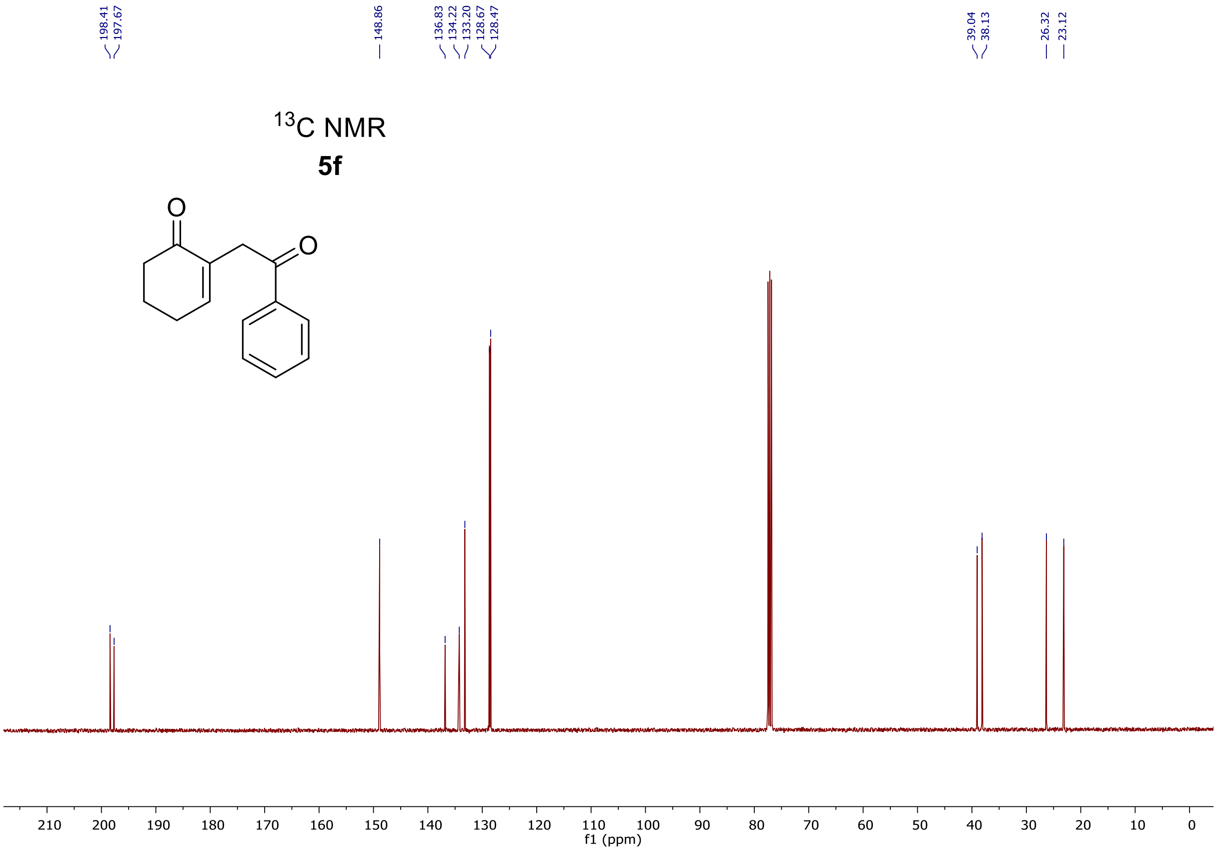

- ***2-(2-([1,1'-Biphenyl]-4-yl)-2-oxoethyl)cyclohex-2-en-1-one (5g)***

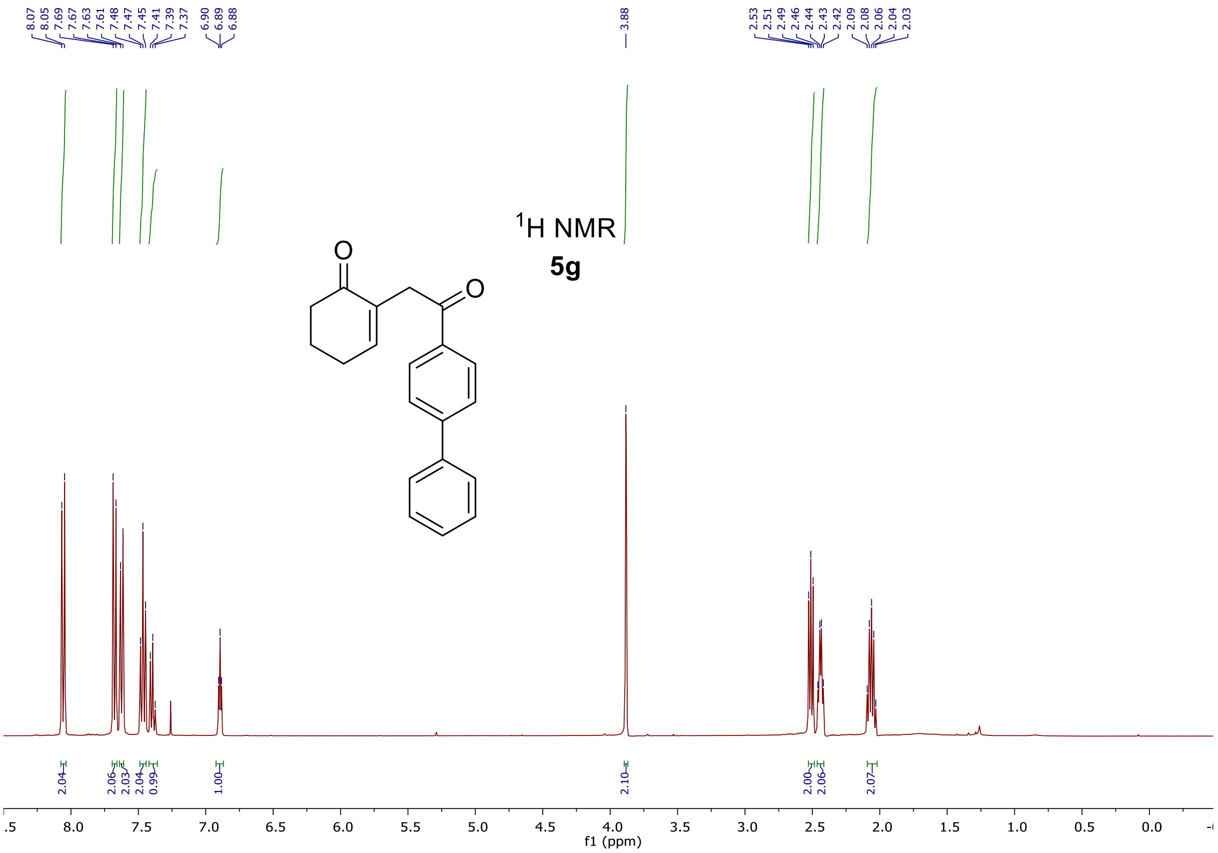

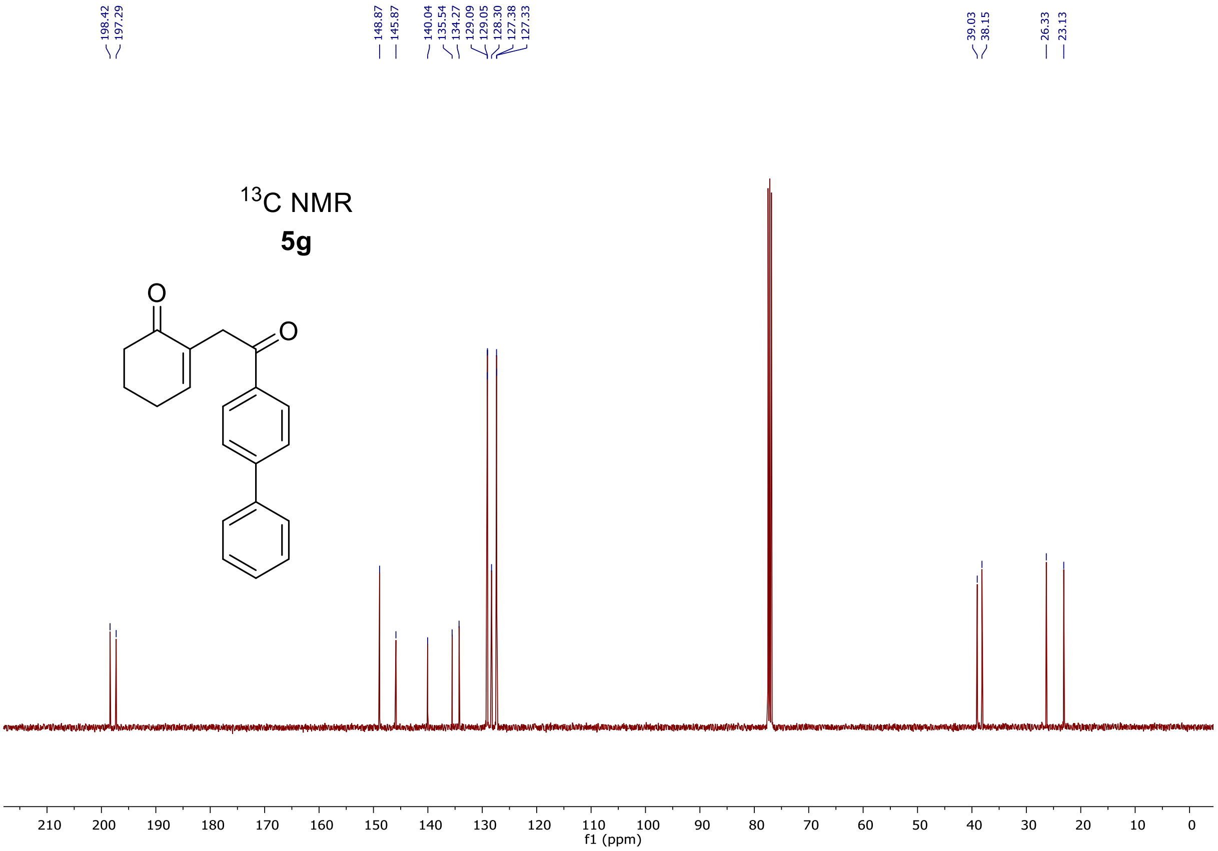

- ***2-(2-(4-Iodophenyl)-2-oxoethyl)cyclohex-2-en-1-one (5h)***

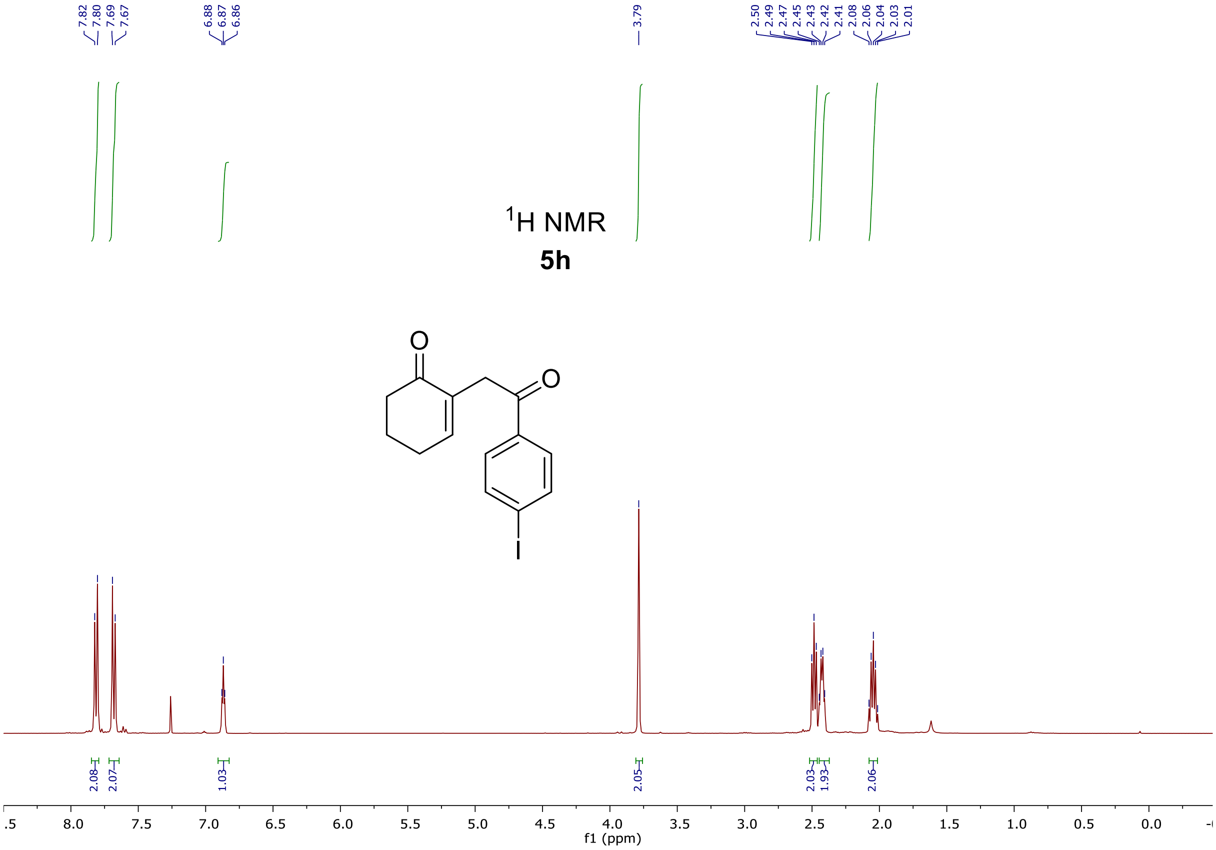

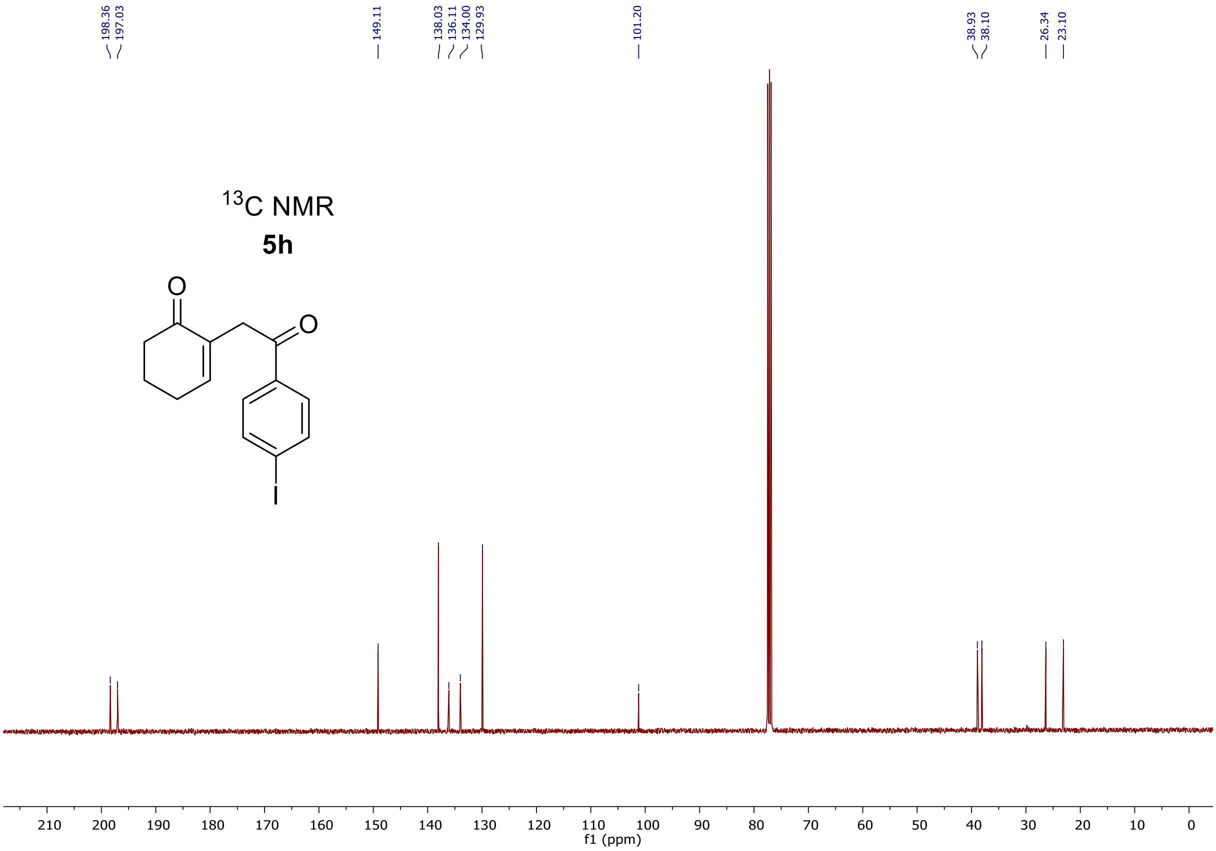

- ***2-(2-(4-Fluorophenyl)-2-oxoethyl)cyclohex-2-en-1-one (5i)***

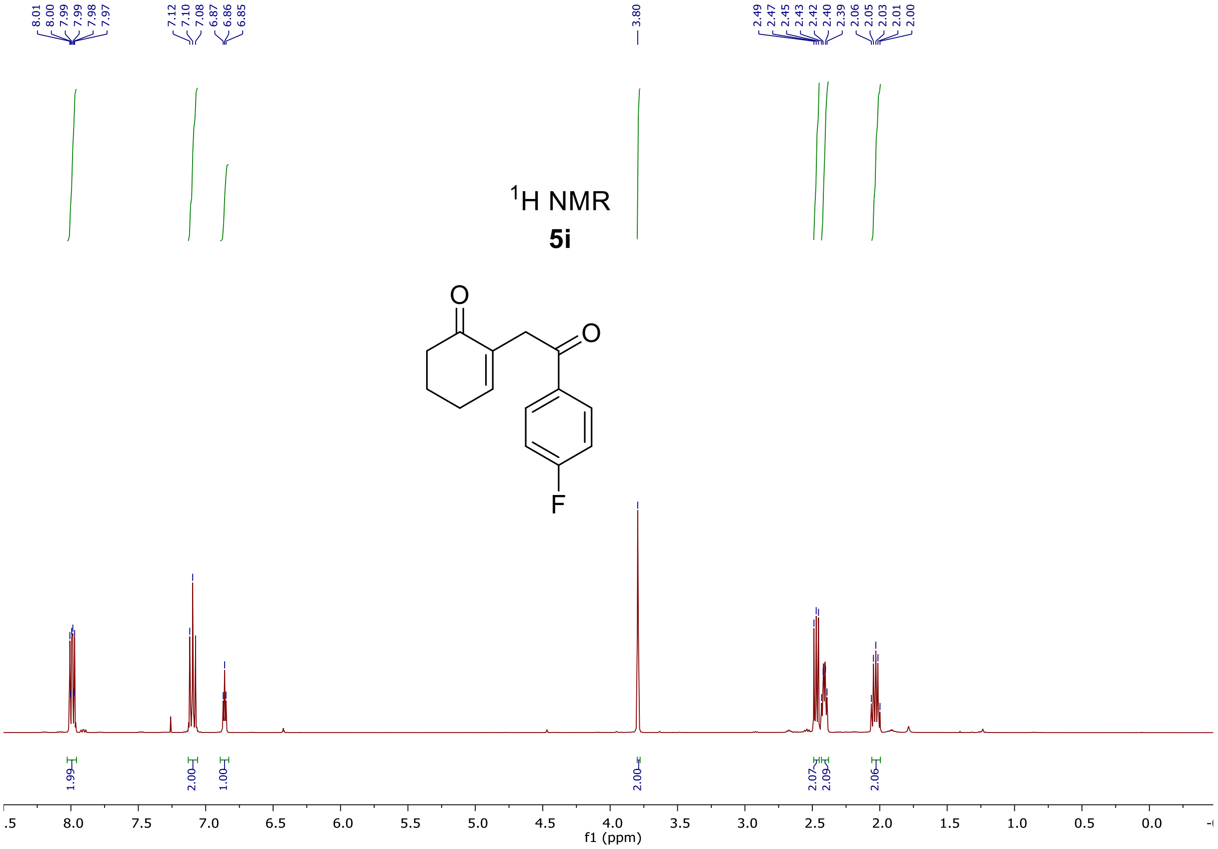

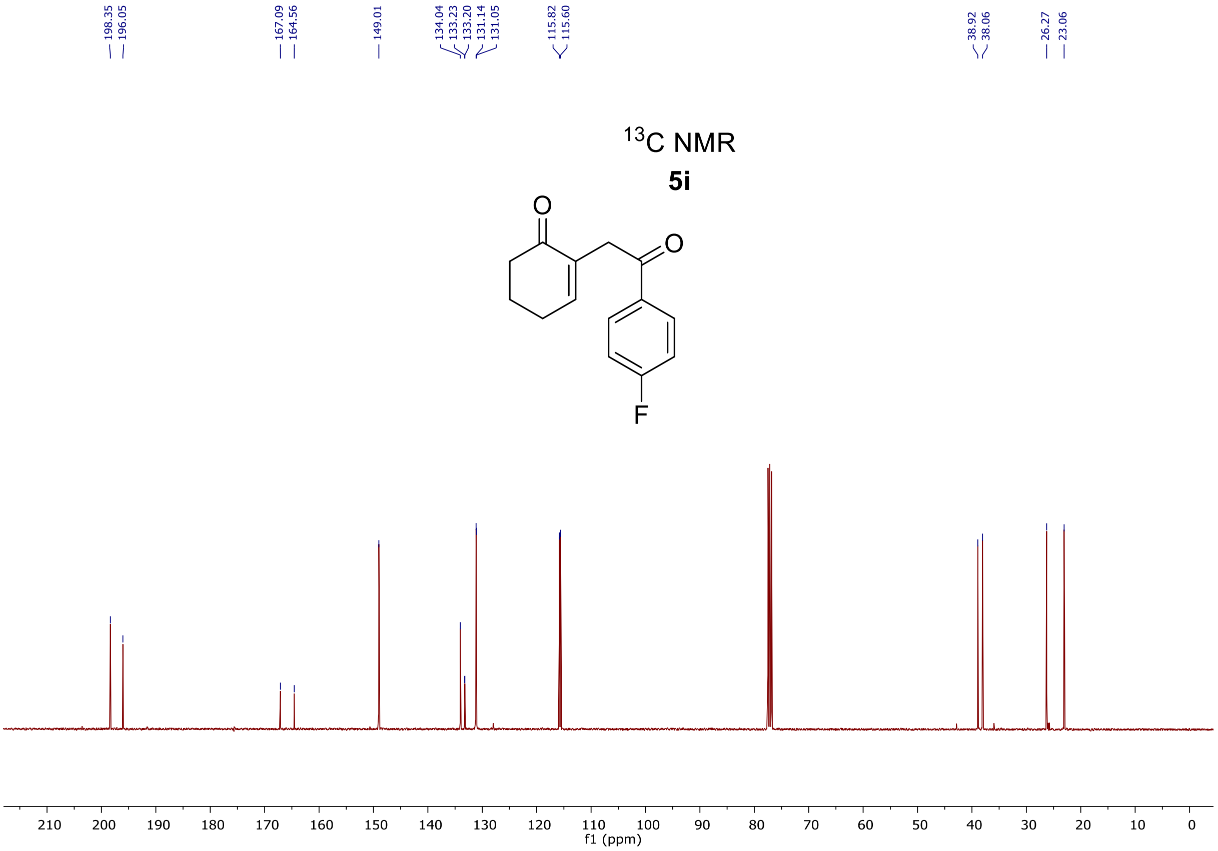

^{19}F NMR

5i

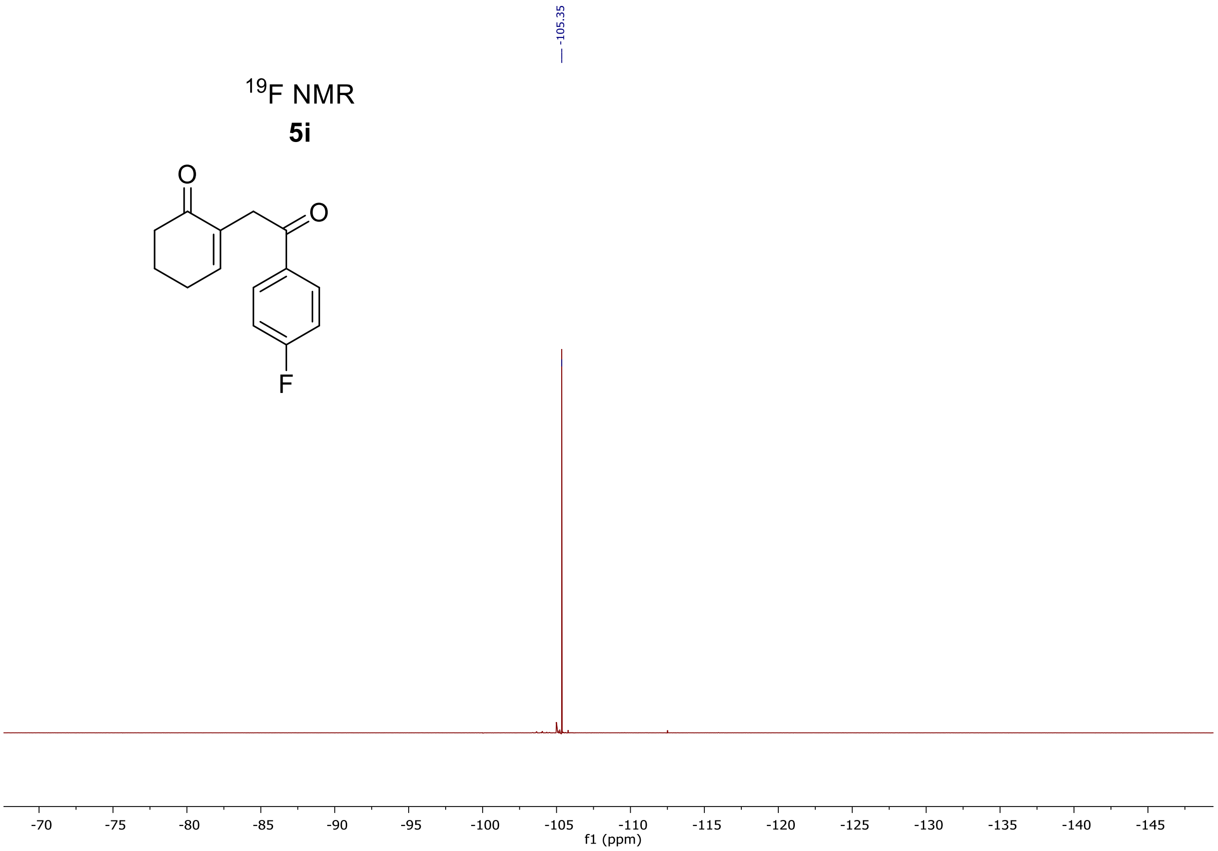
O
O
F
-105.35
-70
-75
-80
-85
-90
-95
-100
-105
-110
-115
-120
-125
-130
-135
-140
-145
f1 (ppm)

- ***2-(2-Oxo-2-(thiophen-2-yl)ethyl)cyclohex-2-en-1-one (5j)***

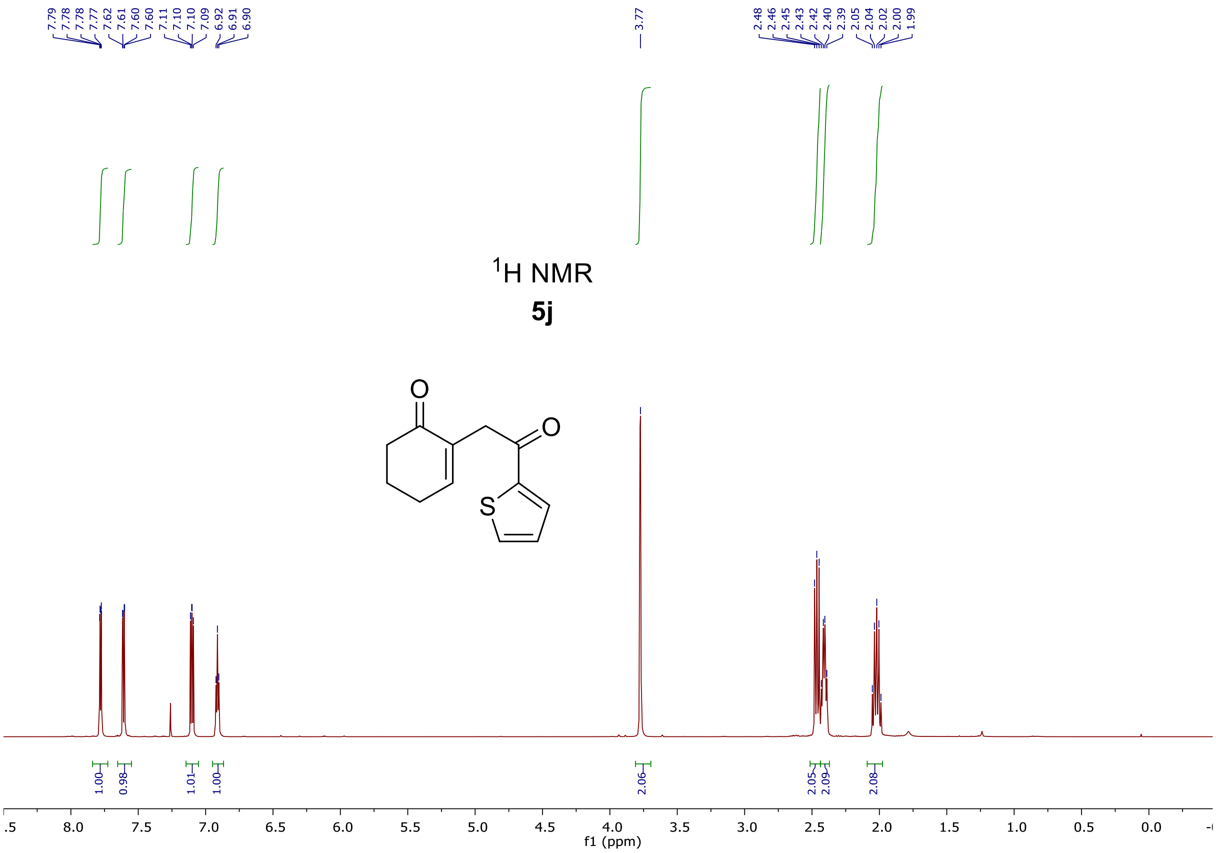

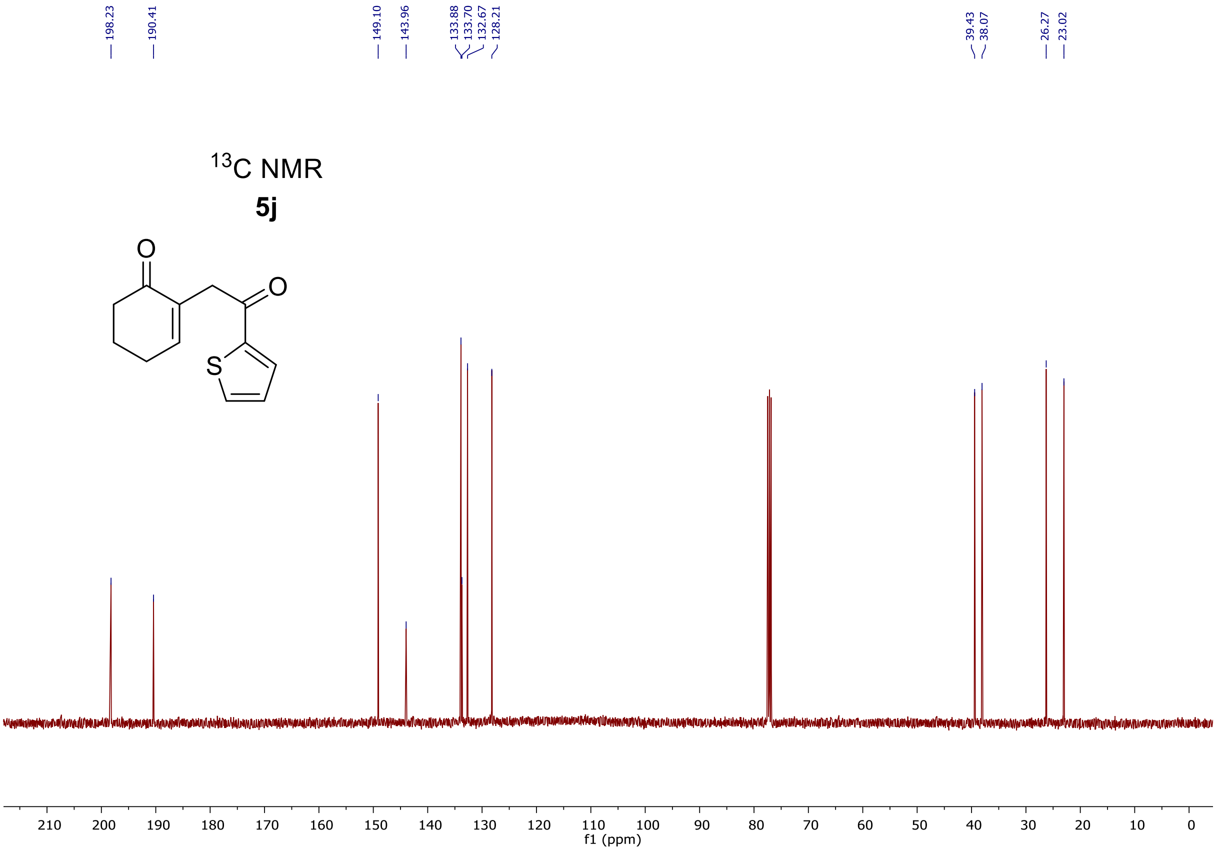

- ***2-(2-Oxo-2-(pyridin-2-yl)ethyl)cyclohex-2-en-1-one (5k)***

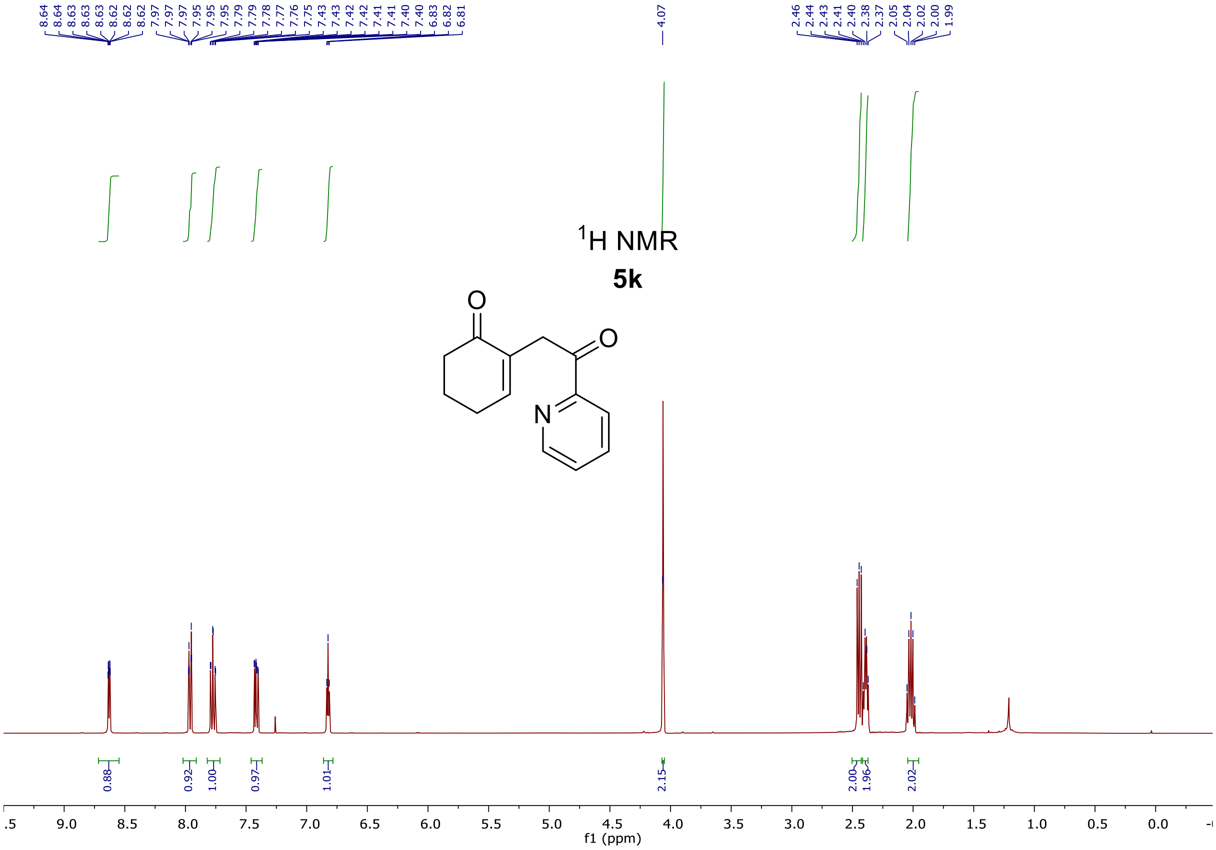

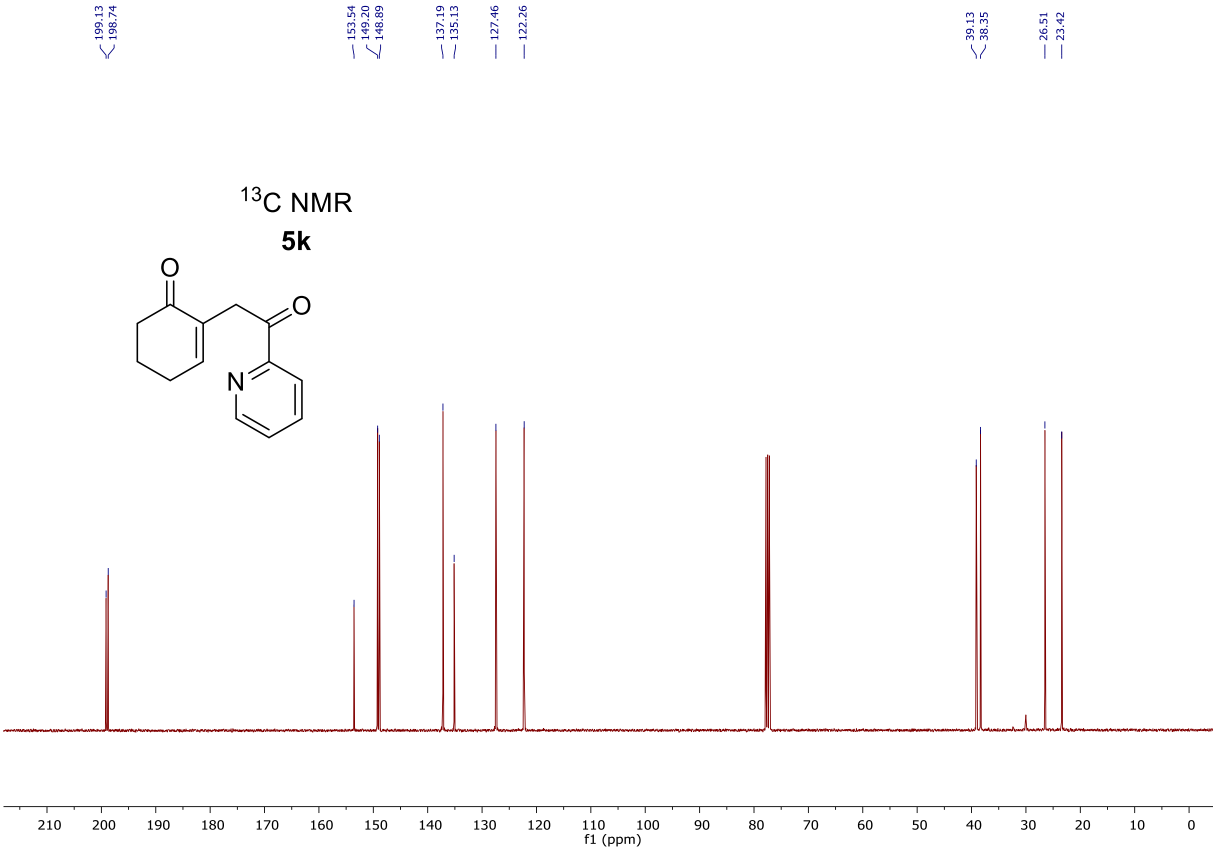

d. Indoles 6a–l.

- ***1-Benzyl-2-methyl-1H-indole (6a)***

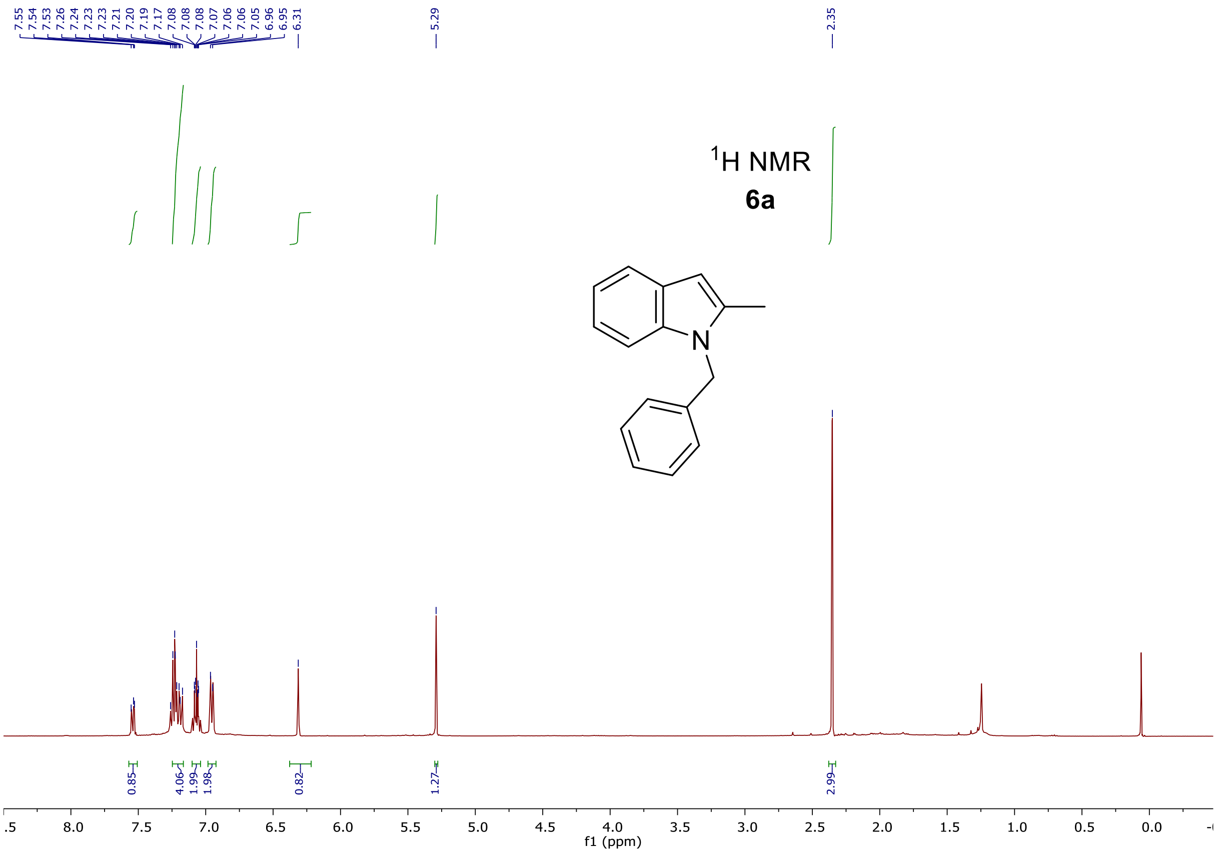

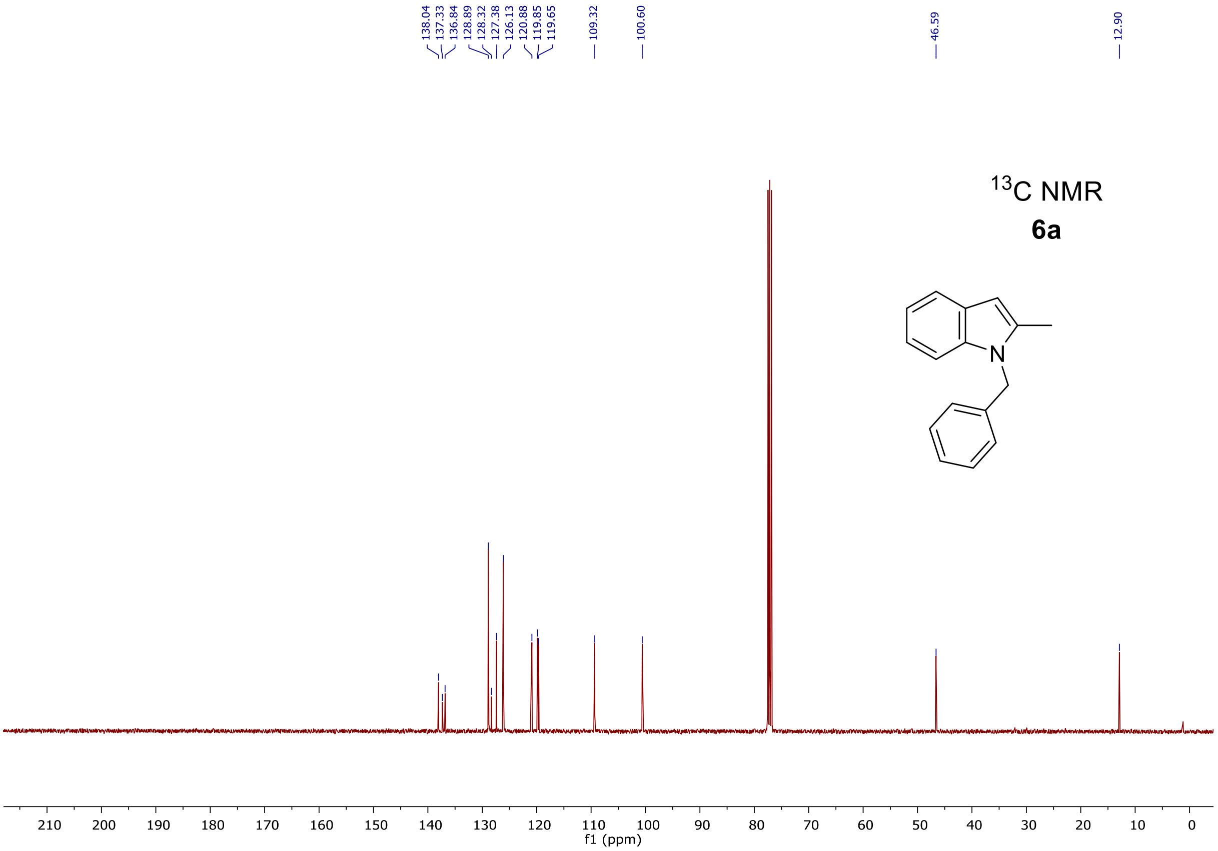

- ***1-Benzyl-2-ethyl-1H-indole (6b)***

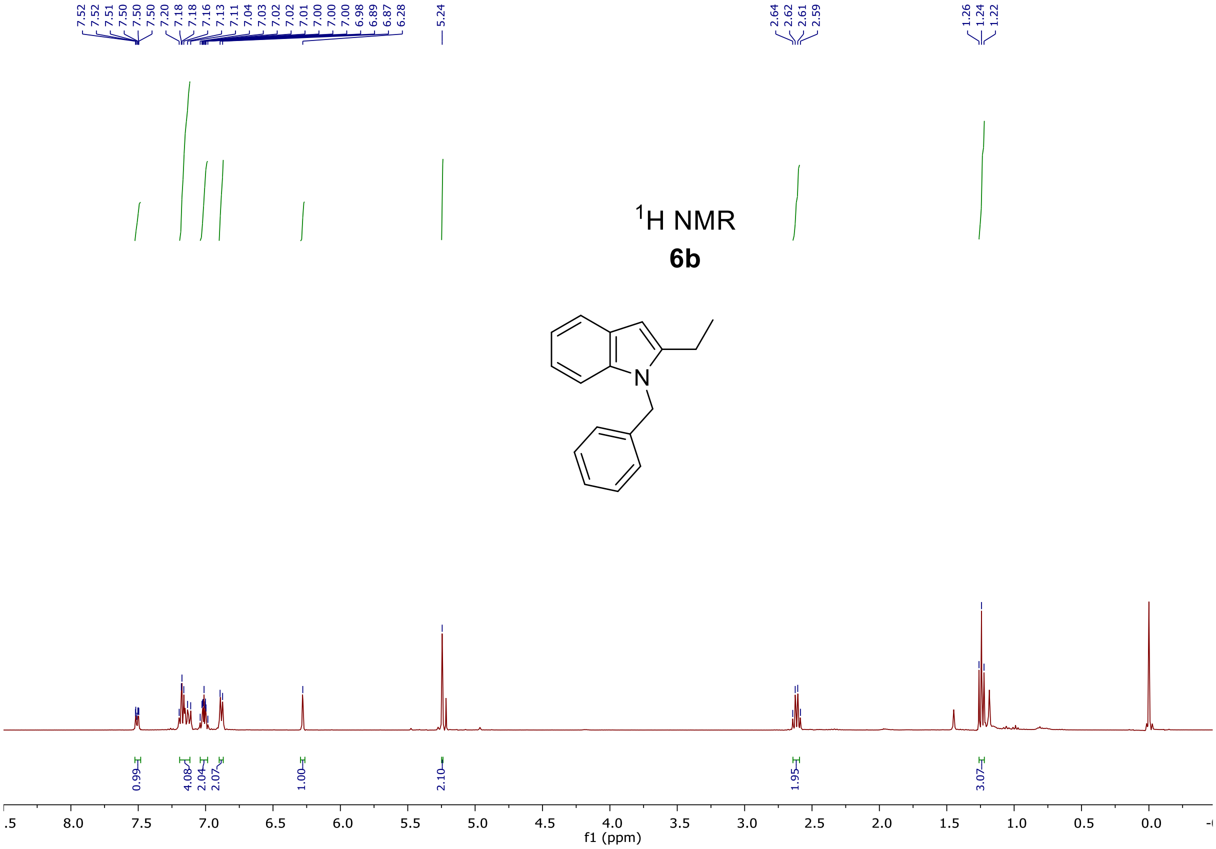

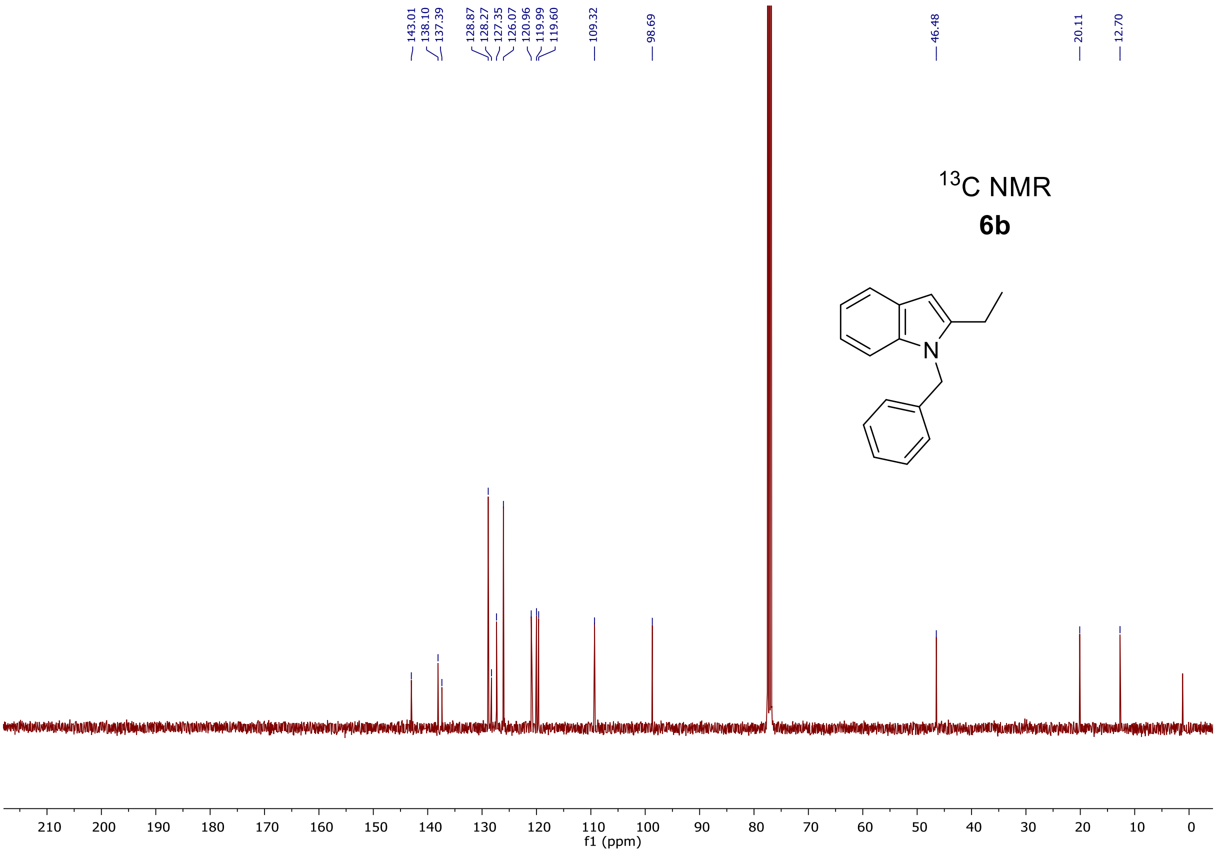

- ***2-Ethyl-1-phenethyl-1H-indole (6c)***

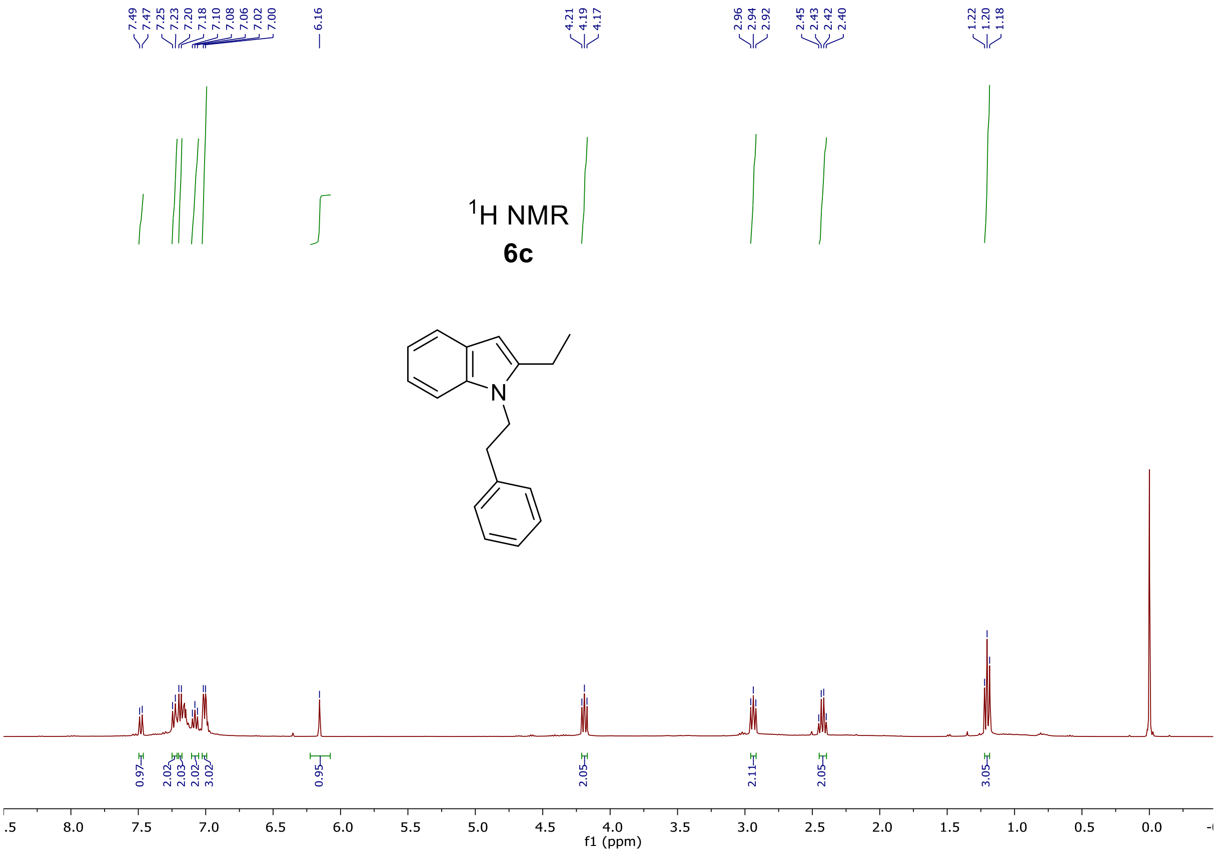

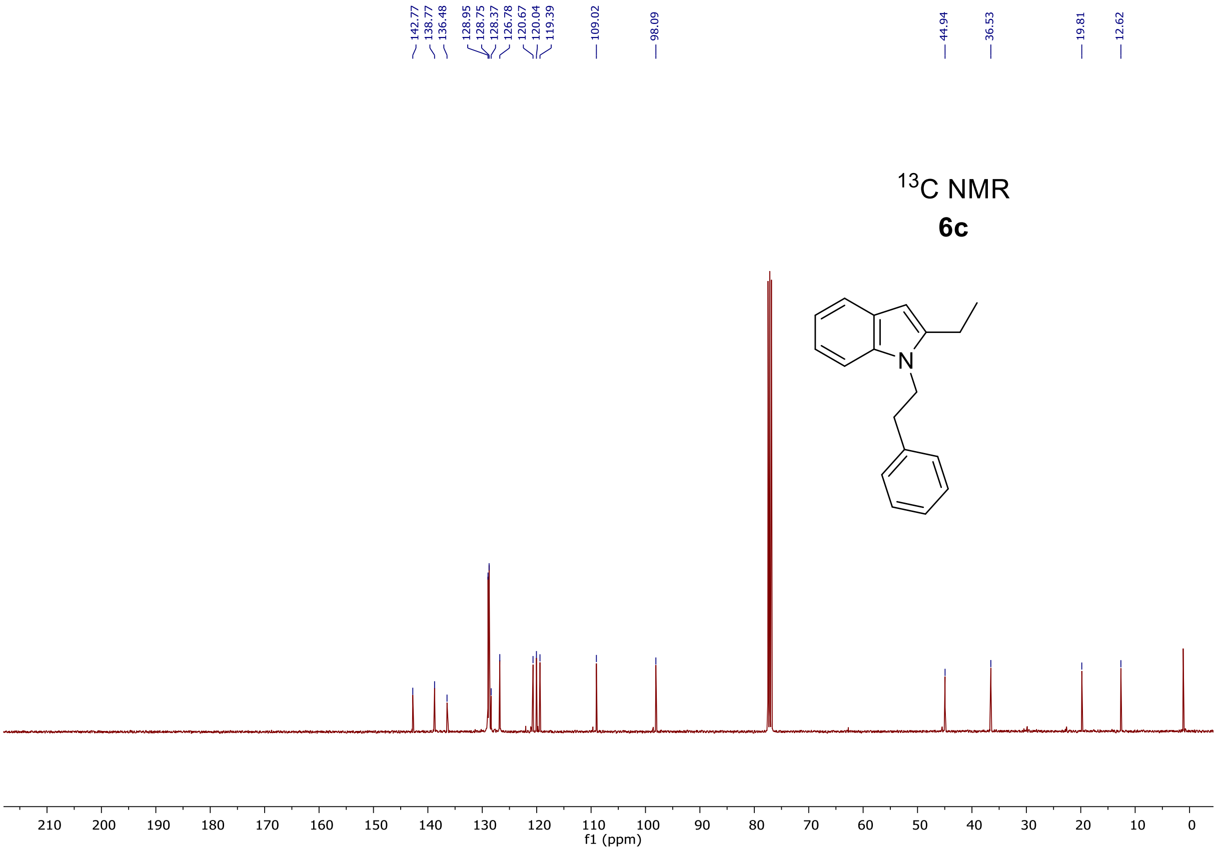

- ***2-Ethyl-1-(4-methoxybenzyl)-1H-indole (6d)***

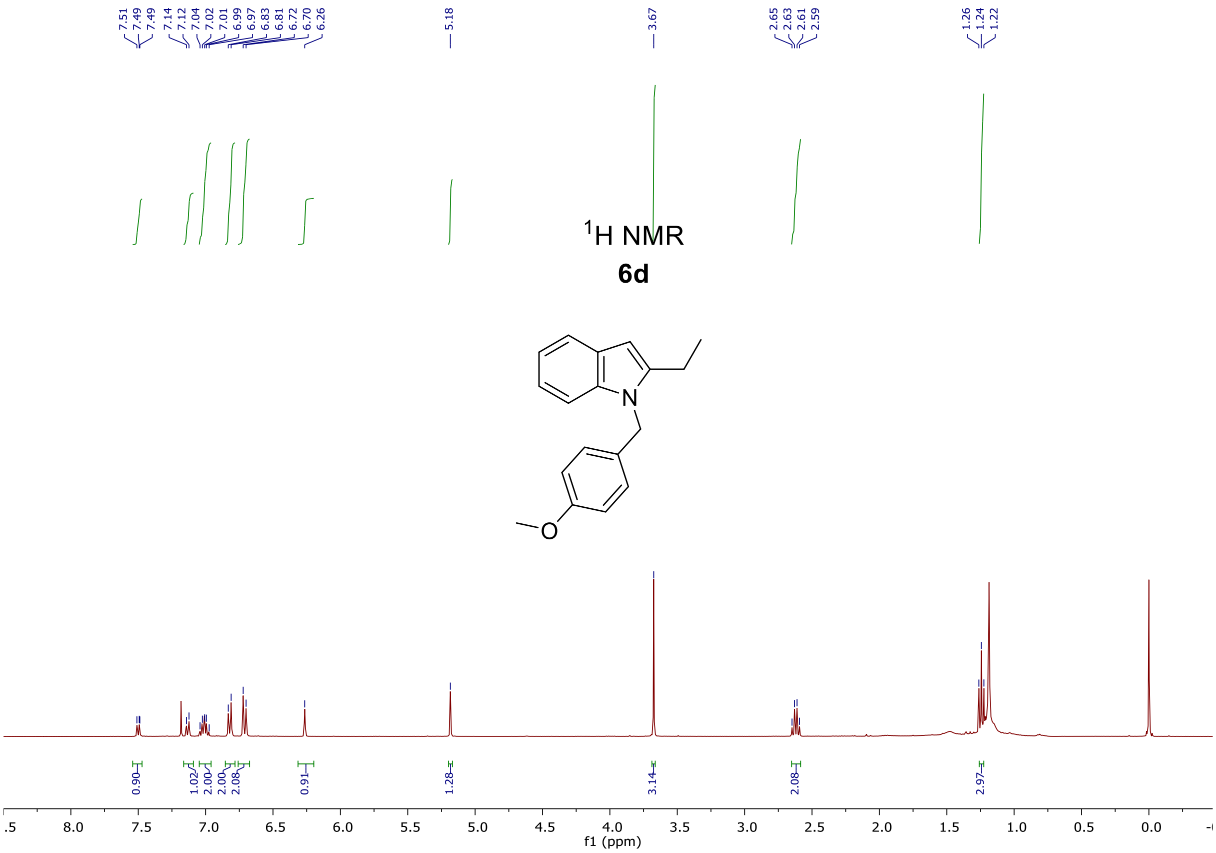

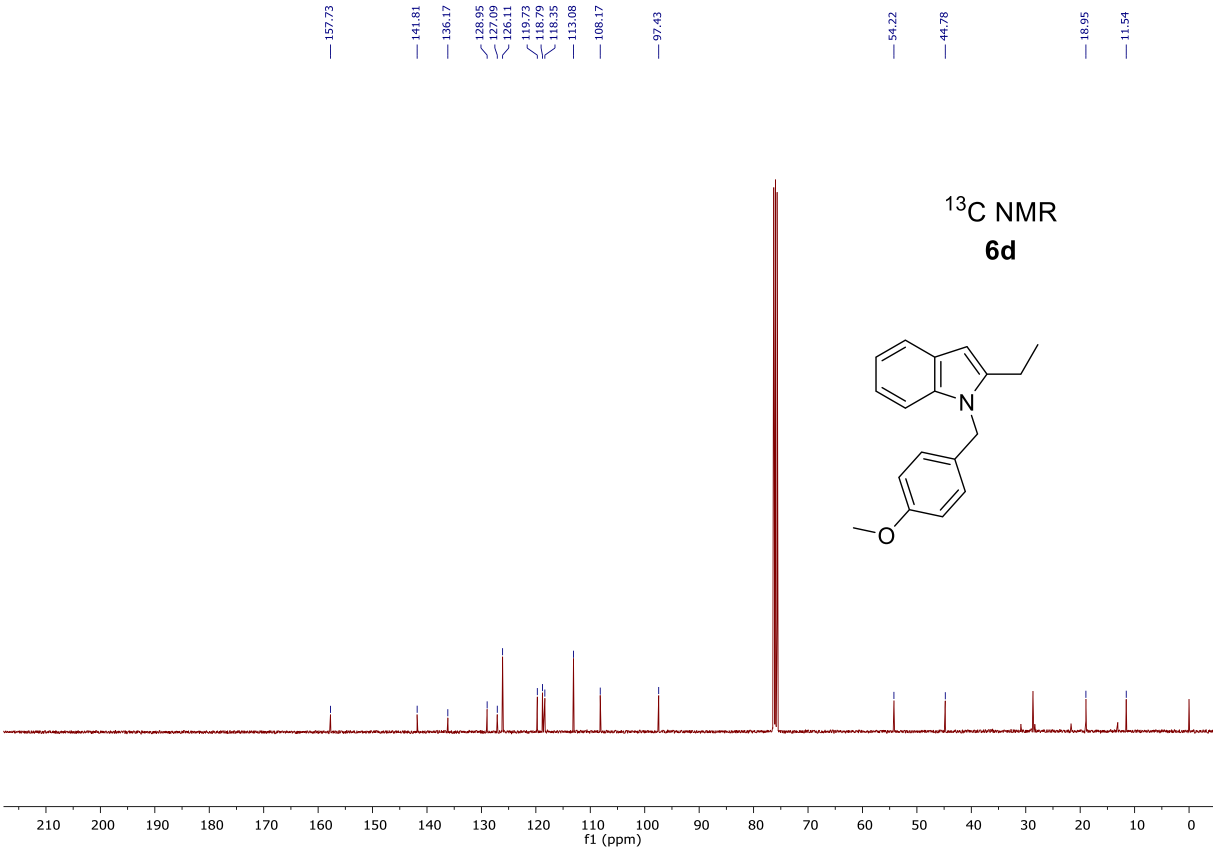

- ***2-Ethyl-1-(1-phenylethyl)-1,5,6,7-tetrahydro-4H-indol-4-one (6e)***

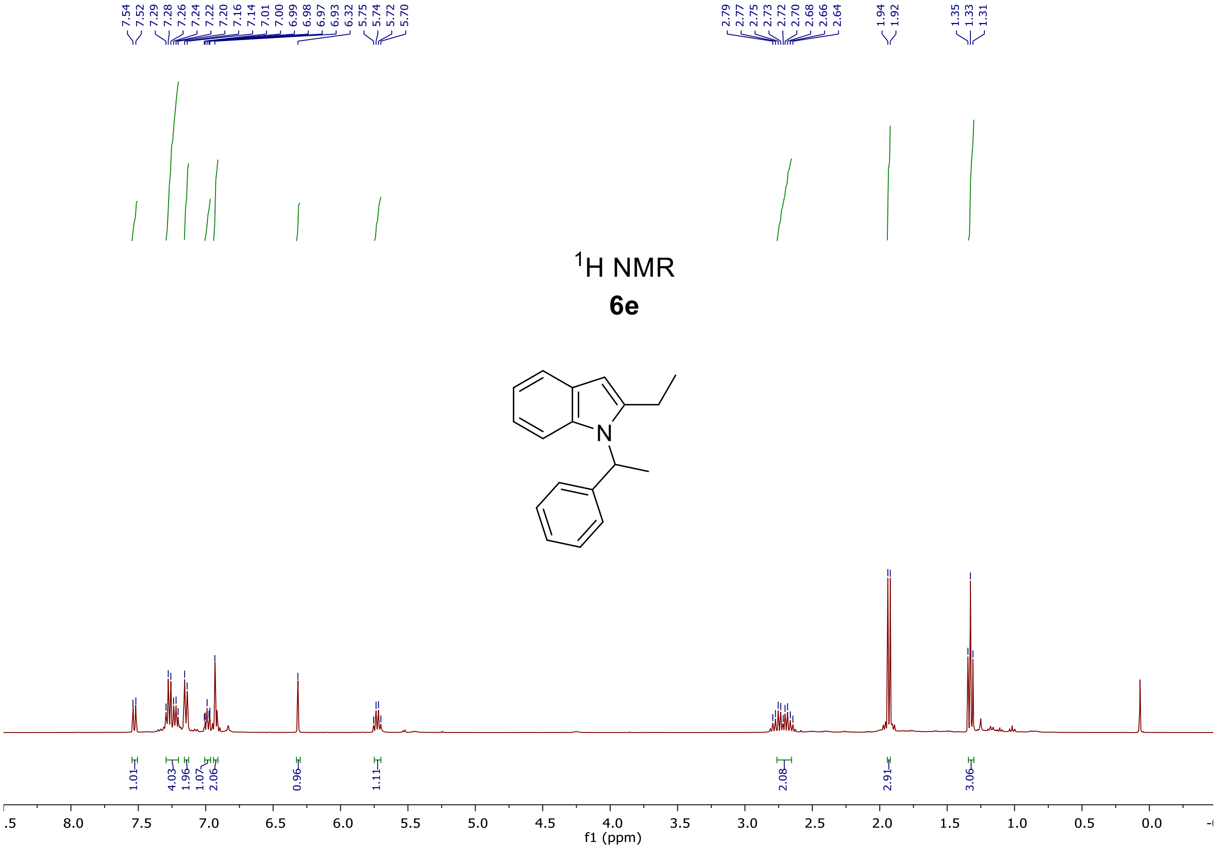

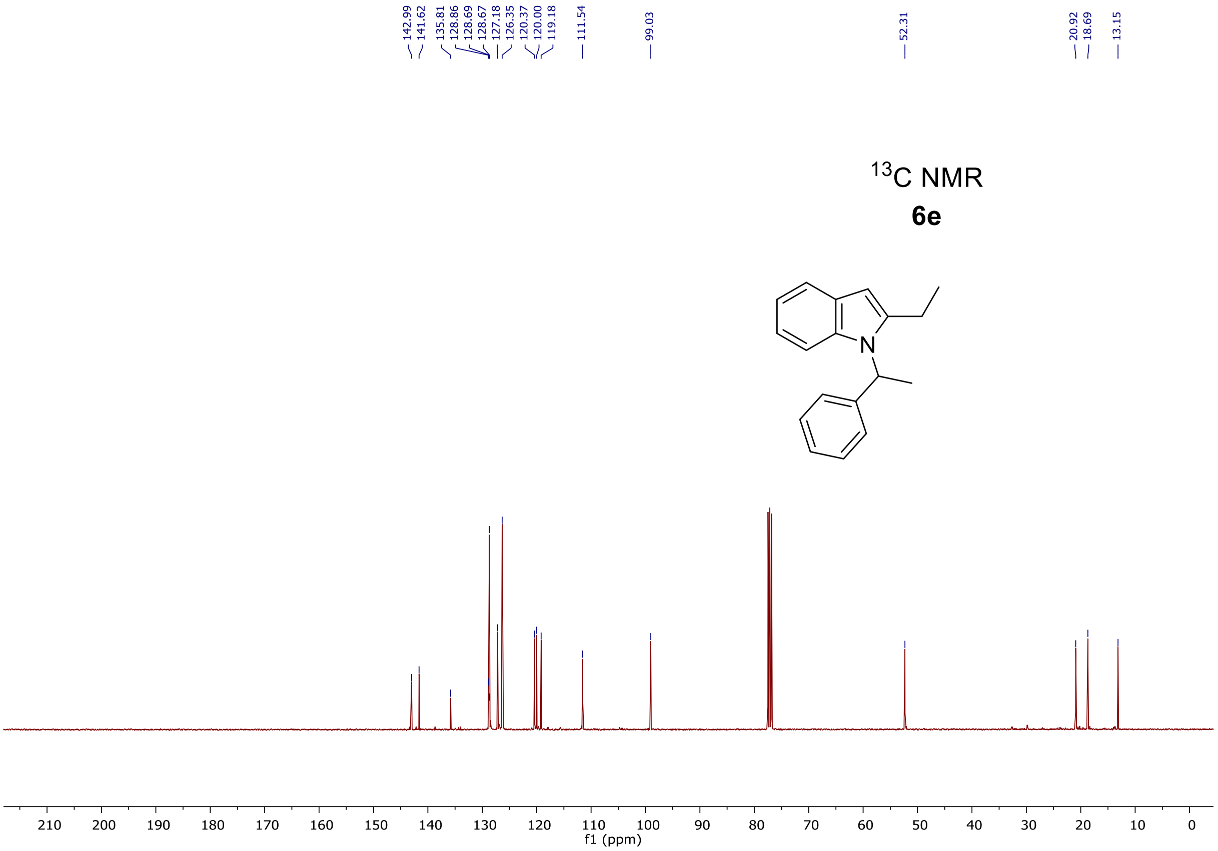

- ***2-Ethyl-1-(furan-3-ylmethyl)-1H-indole (6f)***

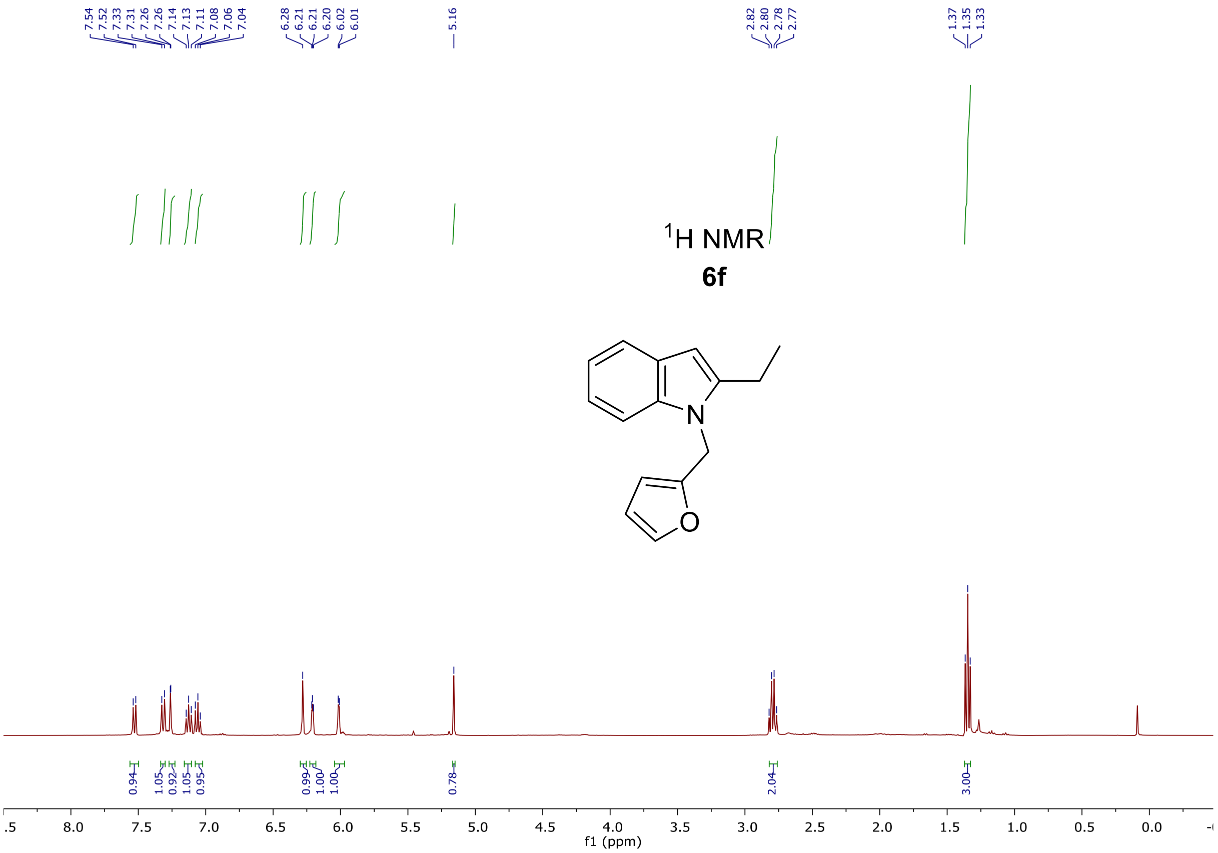

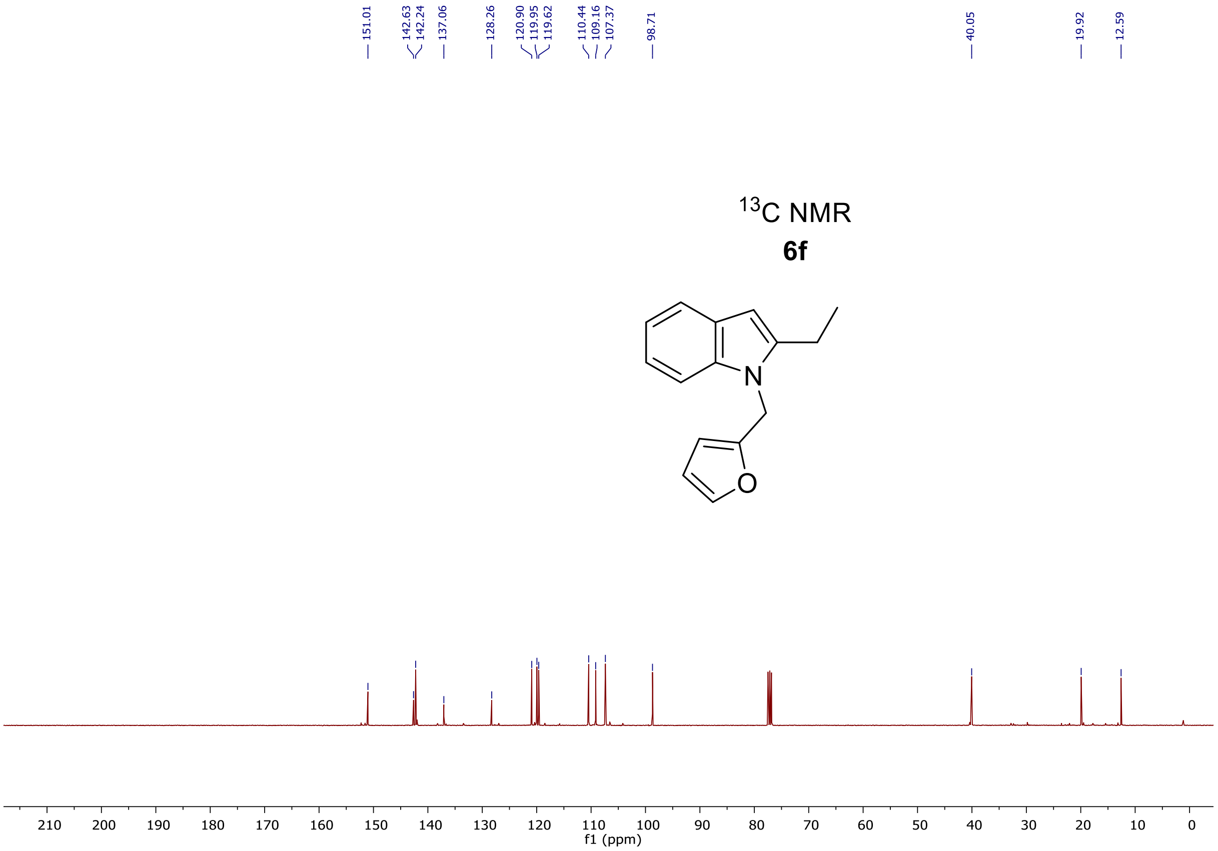

- ***1,3-Bis(2-ethyl-1H-indol-1-yl)propane (6g)***

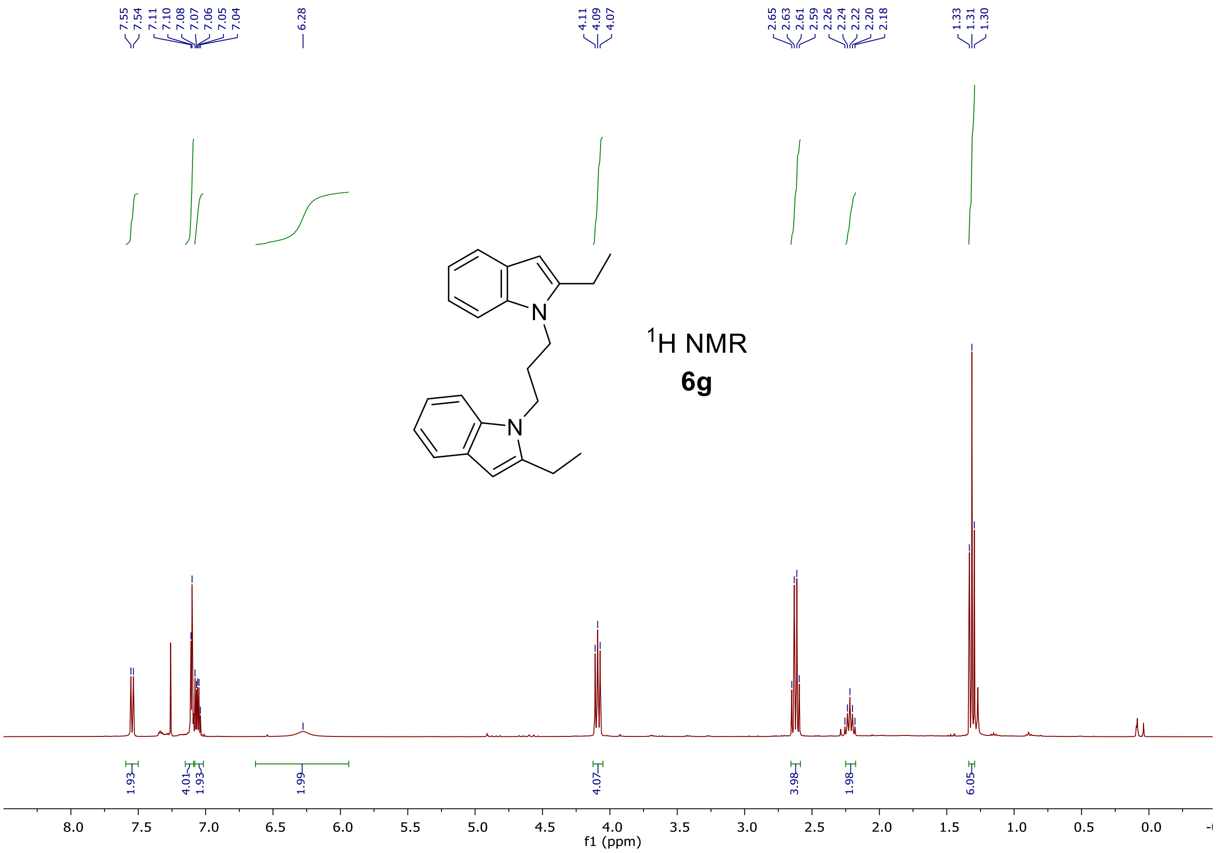

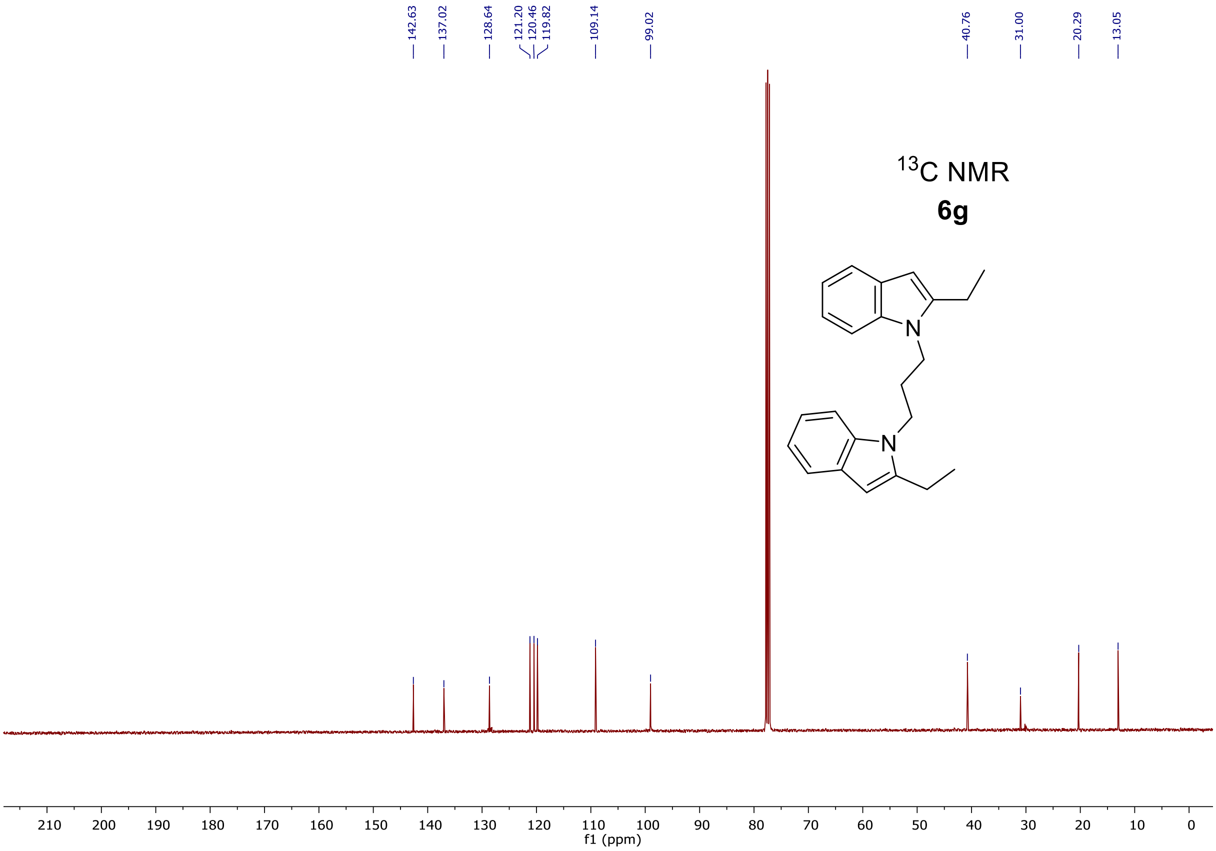

- ***3-(2-Ethyl-1H-indol-1-yl)-N,N-dimethylpropan-1-amine (6h)***

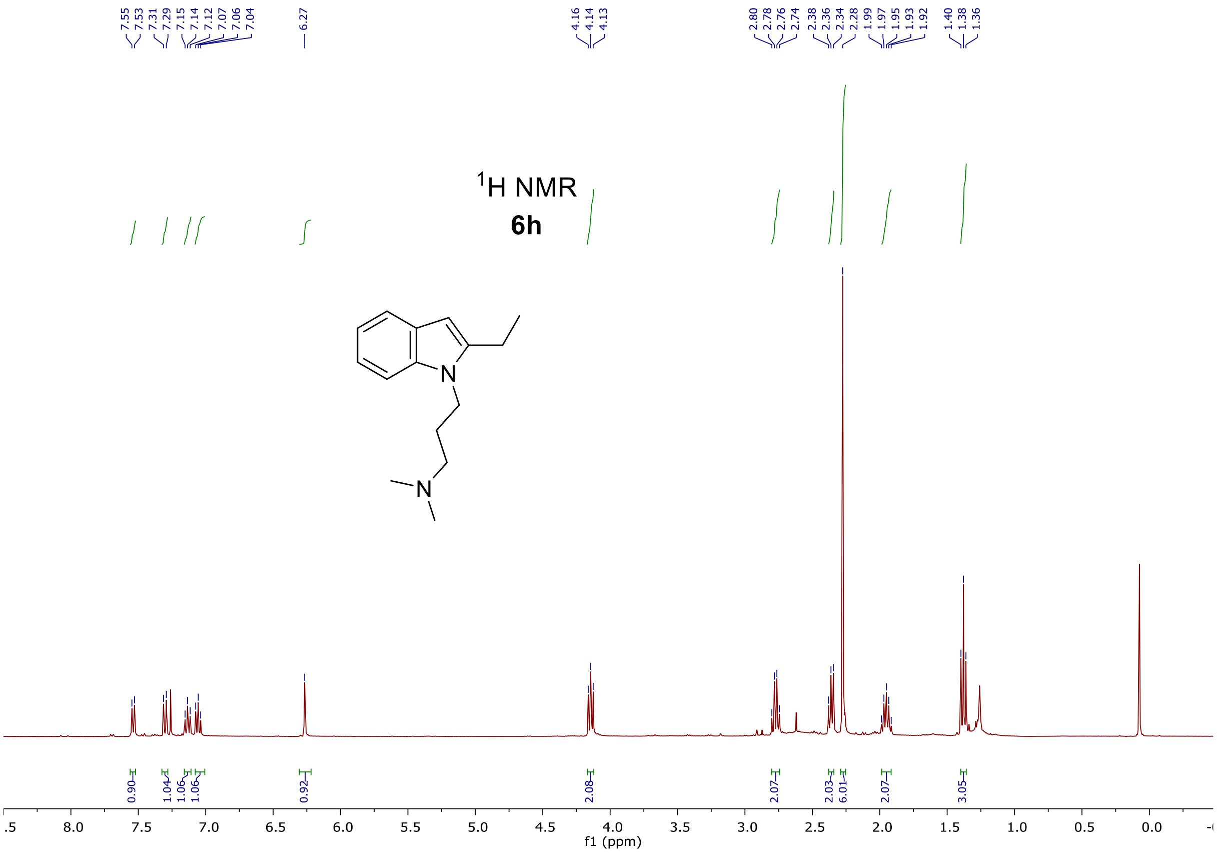

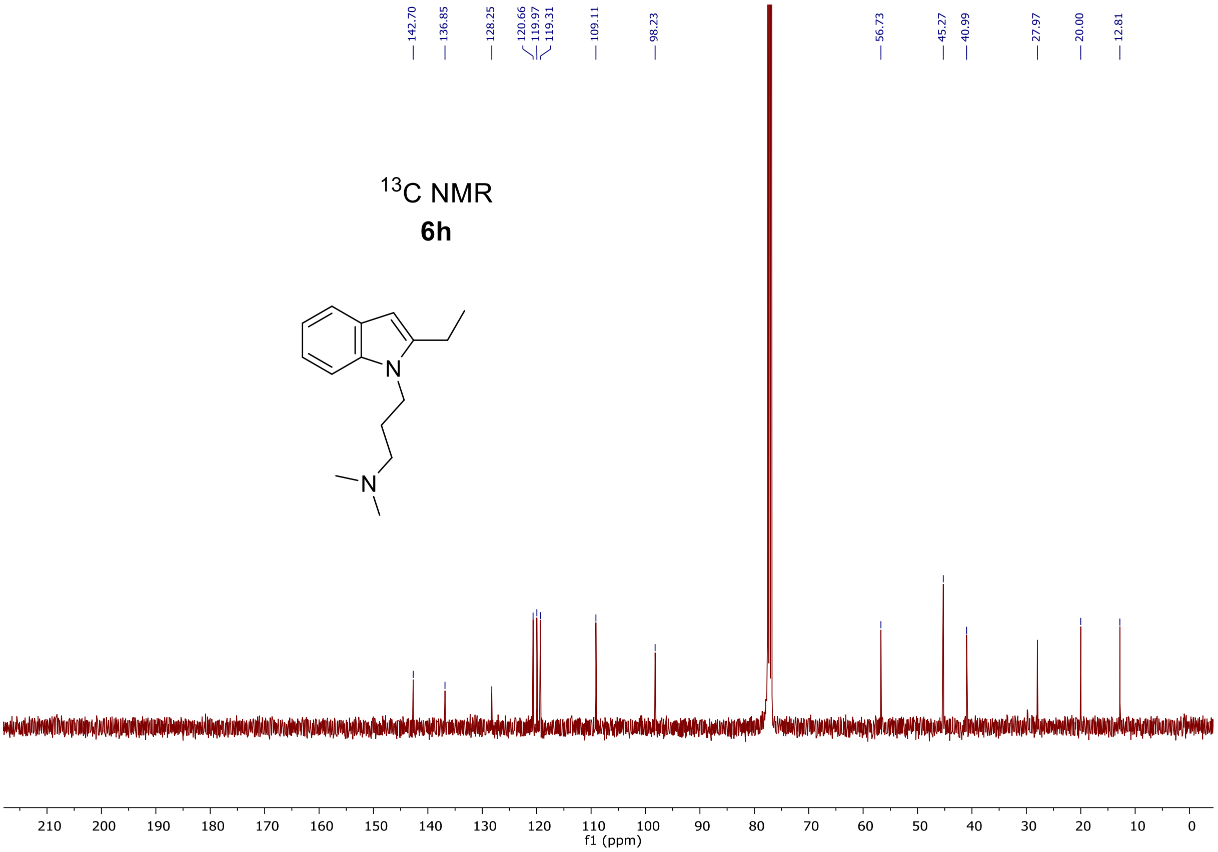

- ***4-(2-(2-Methyl-1H-indol-1-yl)ethyl)morpholine (6i)***

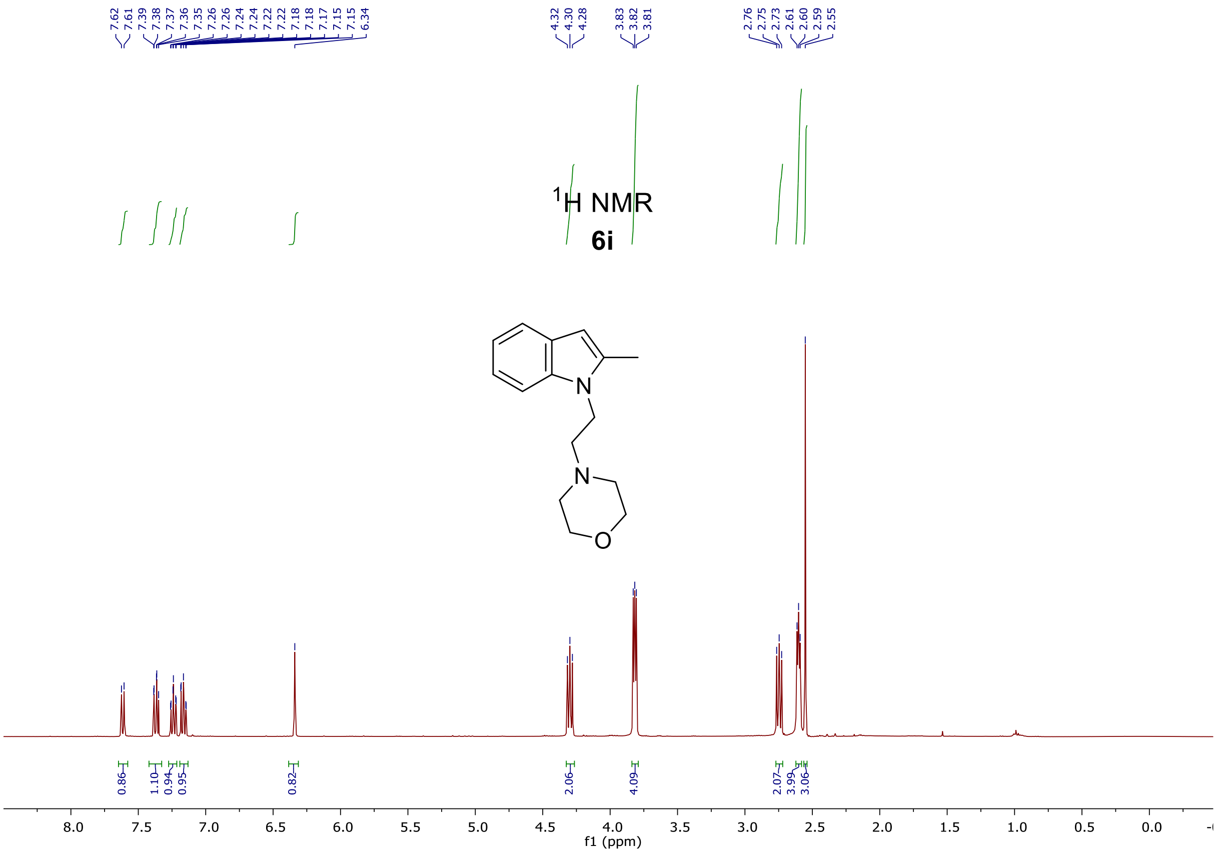

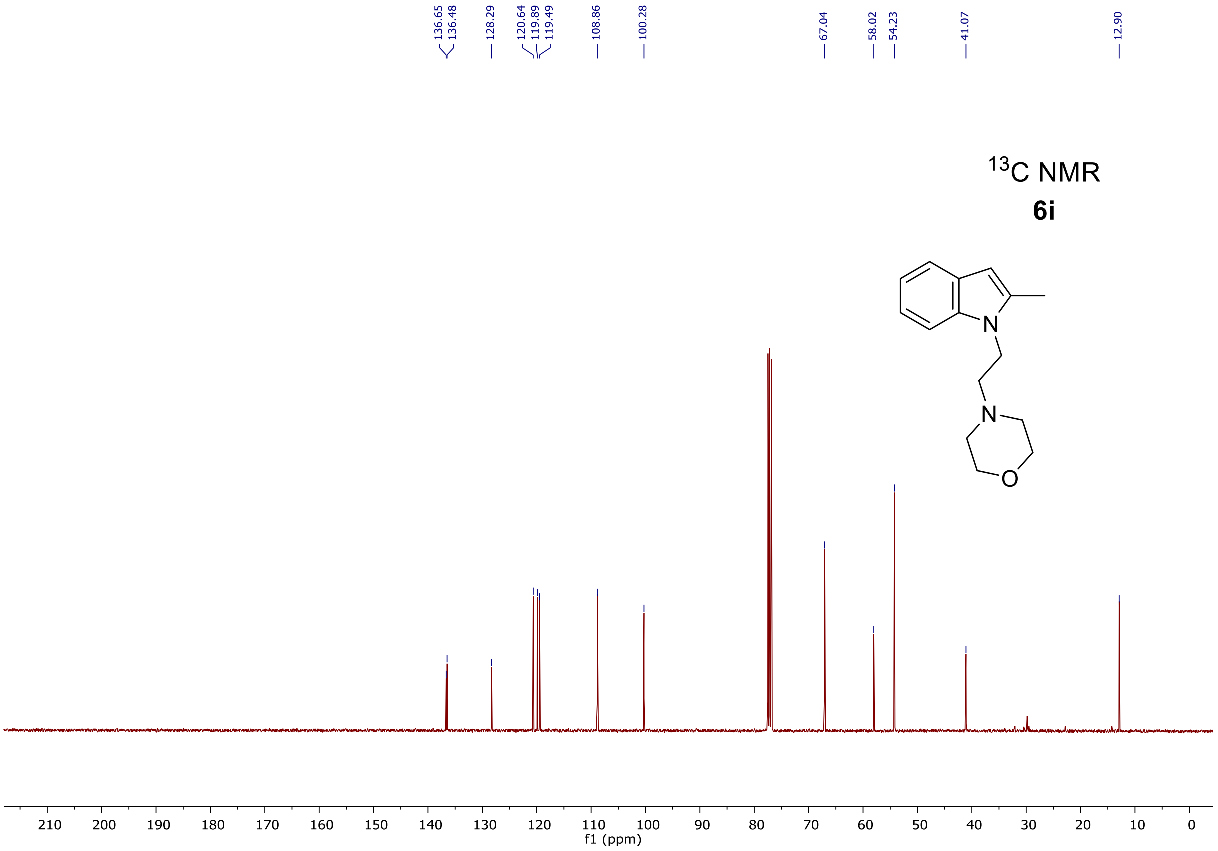

- ***4-(2-(2-Ethyl-1H-indol-1-yl)ethyl)morpholine (6j)***

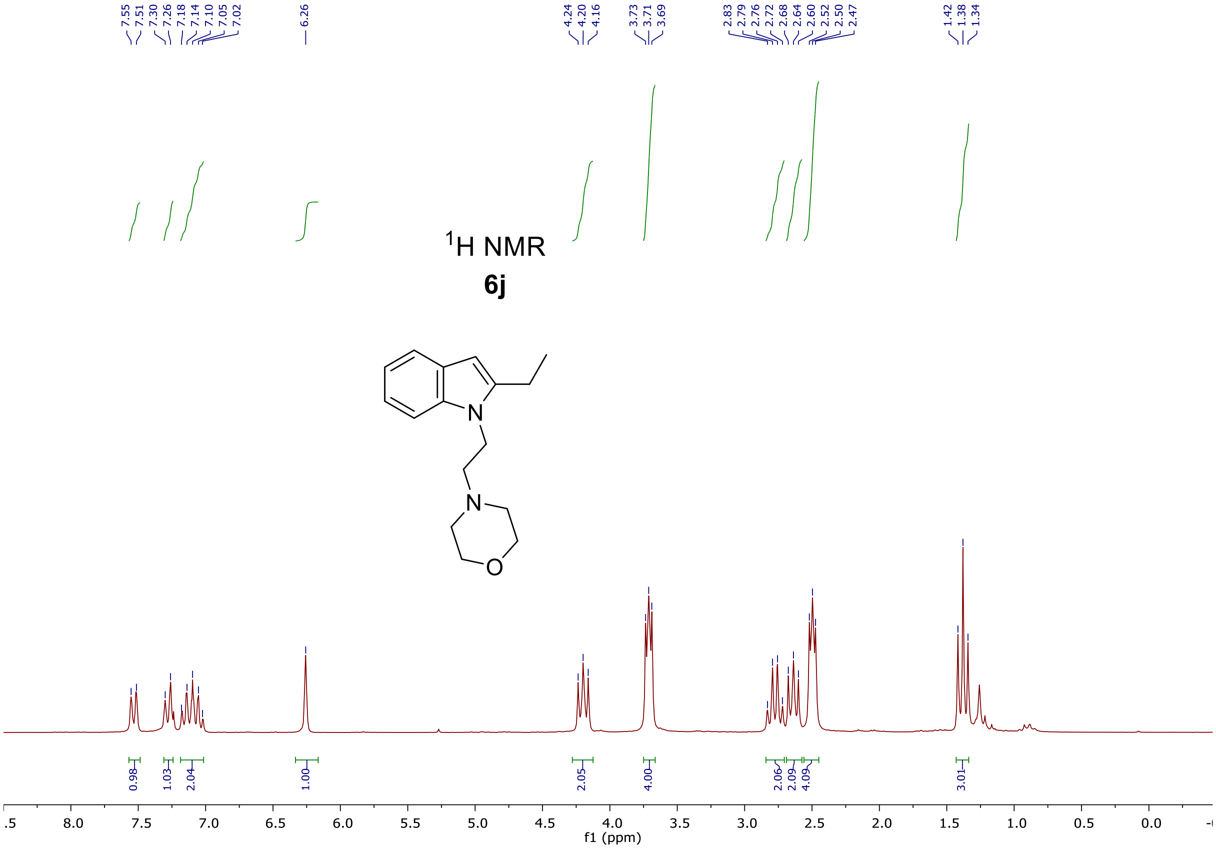

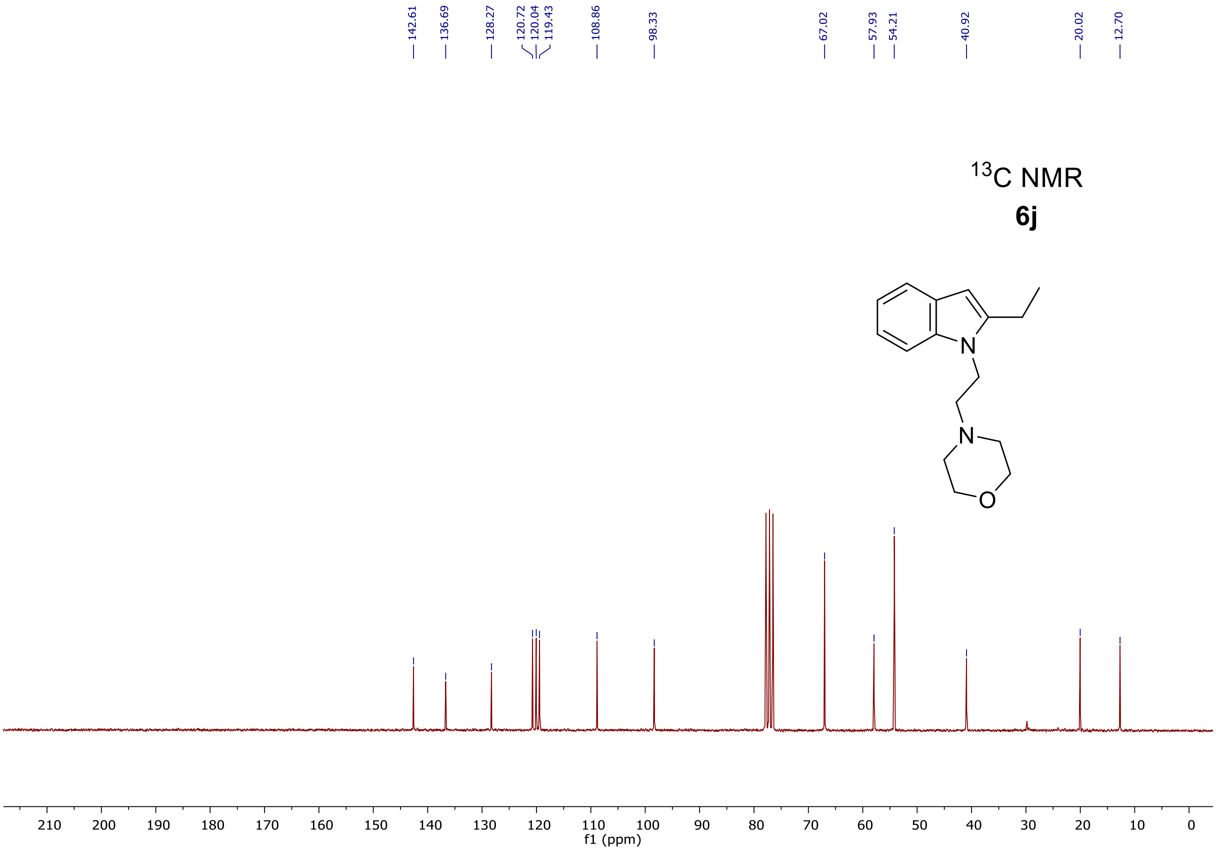

- ***2-Methyl-1-(pyridin-4-ylmethyl)-1H-indole (6k)***

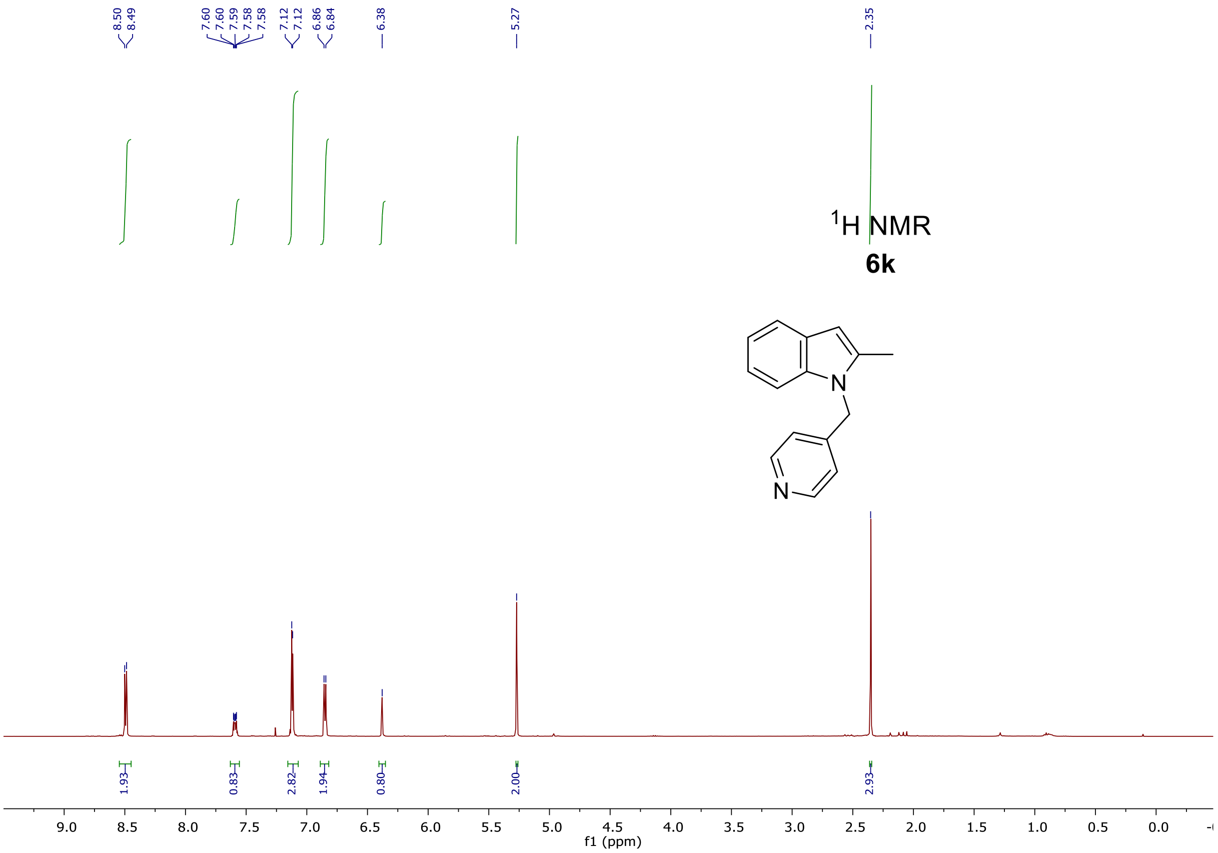

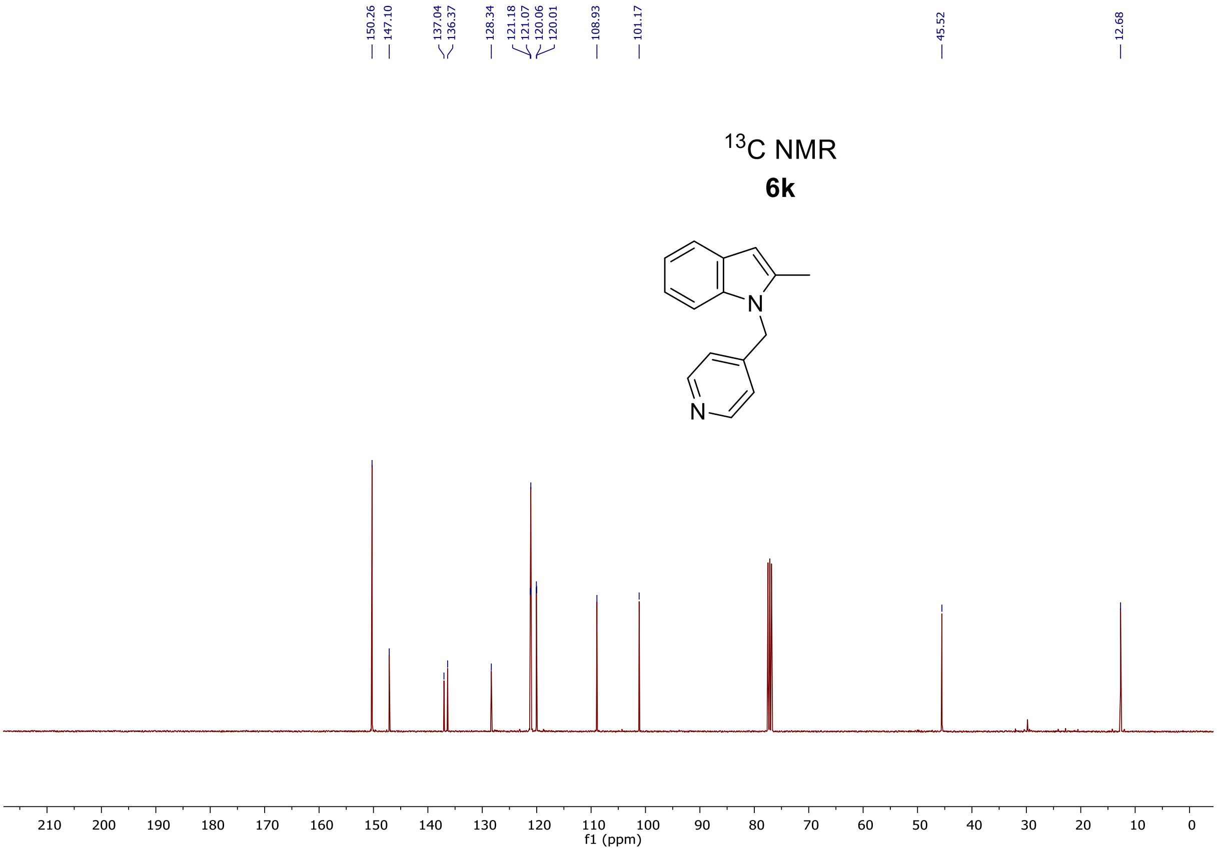

- ***2-Ethyl-1-(pyridin-4-ylmethyl)-1H-indole (6l)***

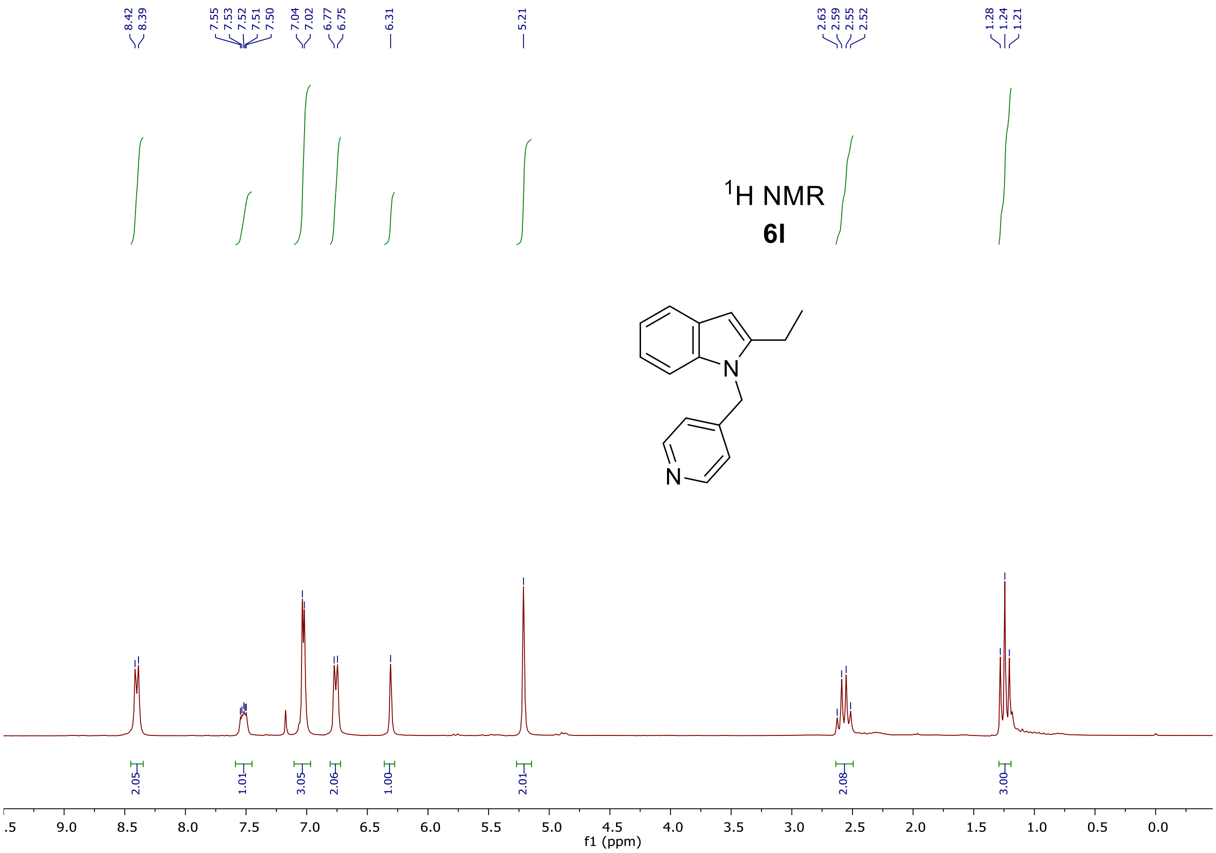

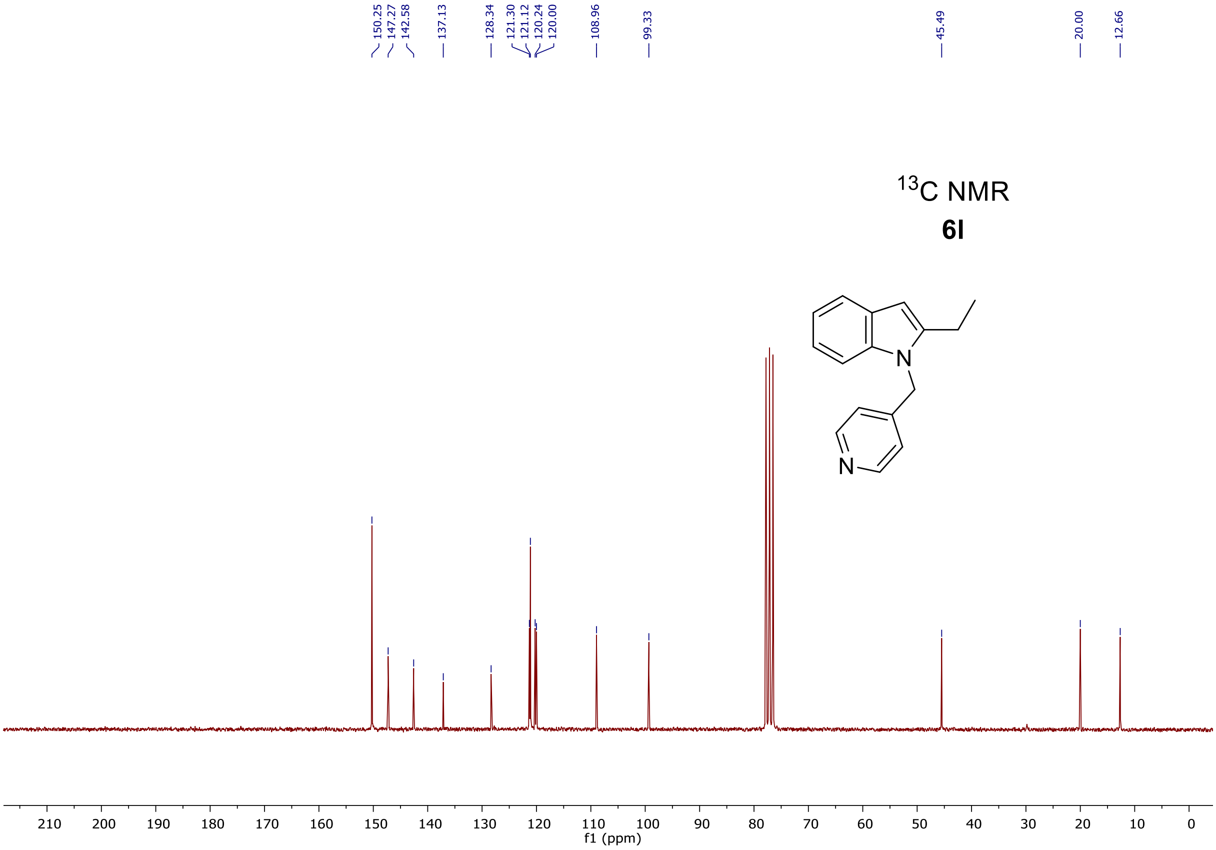

e. Indolones 7a–k.

- ***1-Benzyl-2-methyl-1,5,6,7-tetrahydro-4H-indol-4-one (7a)***

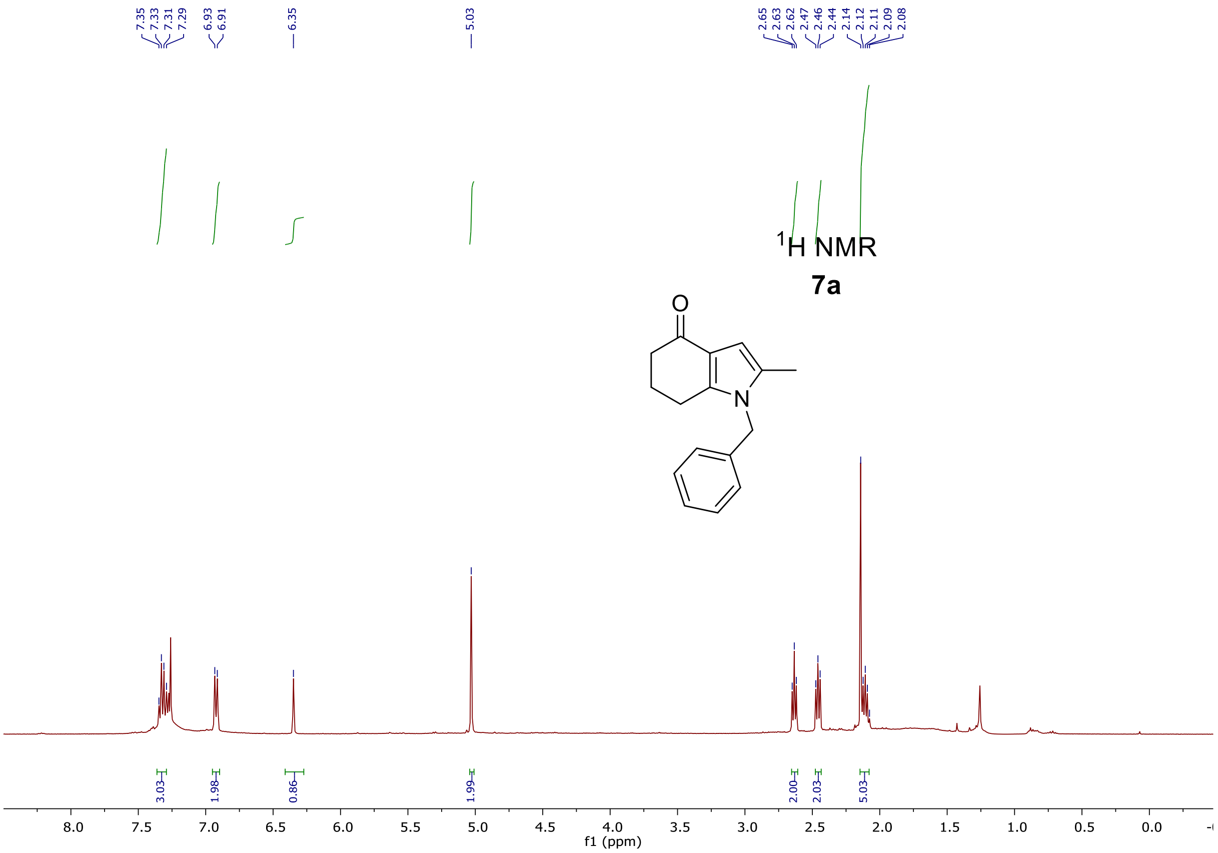

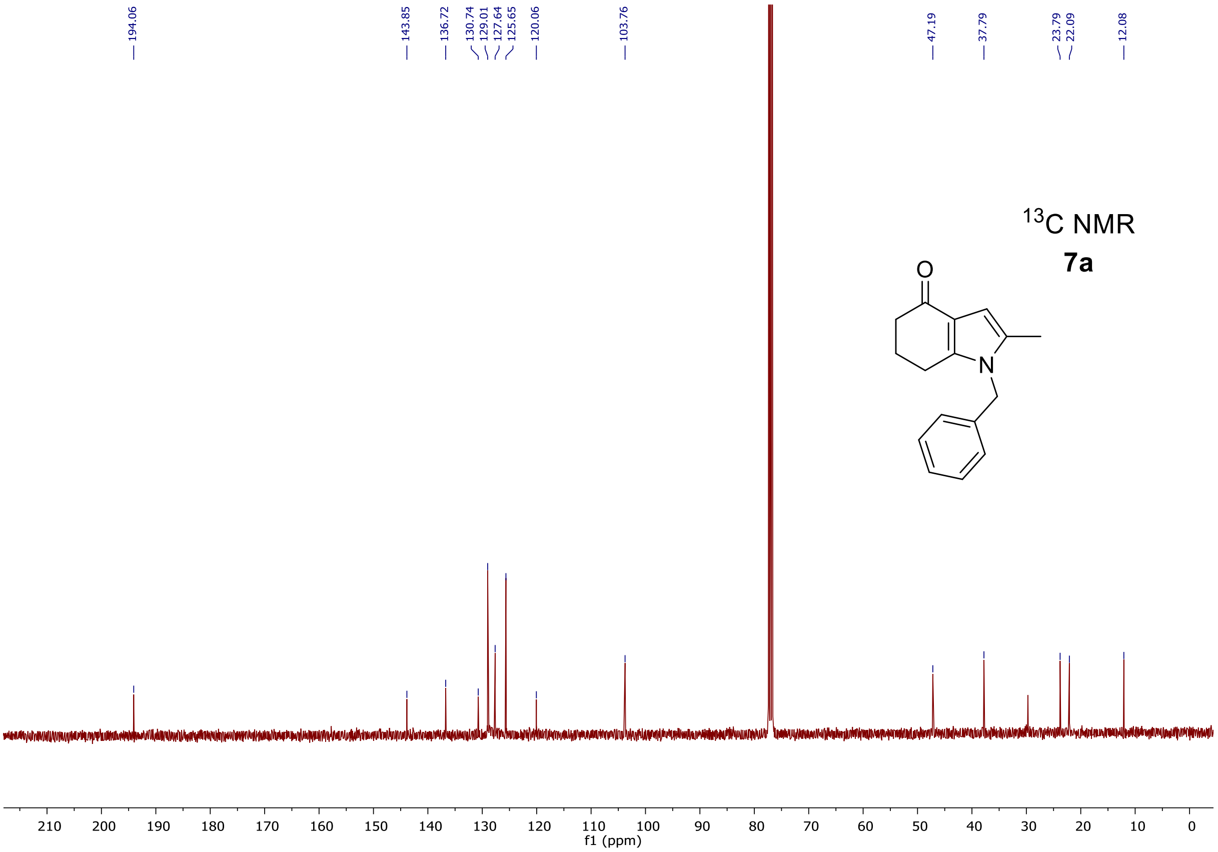

- ***1-Benzyl-2-ethyl-1,5,6,7-tetrahydro-4H-indol-4-one (7b)***

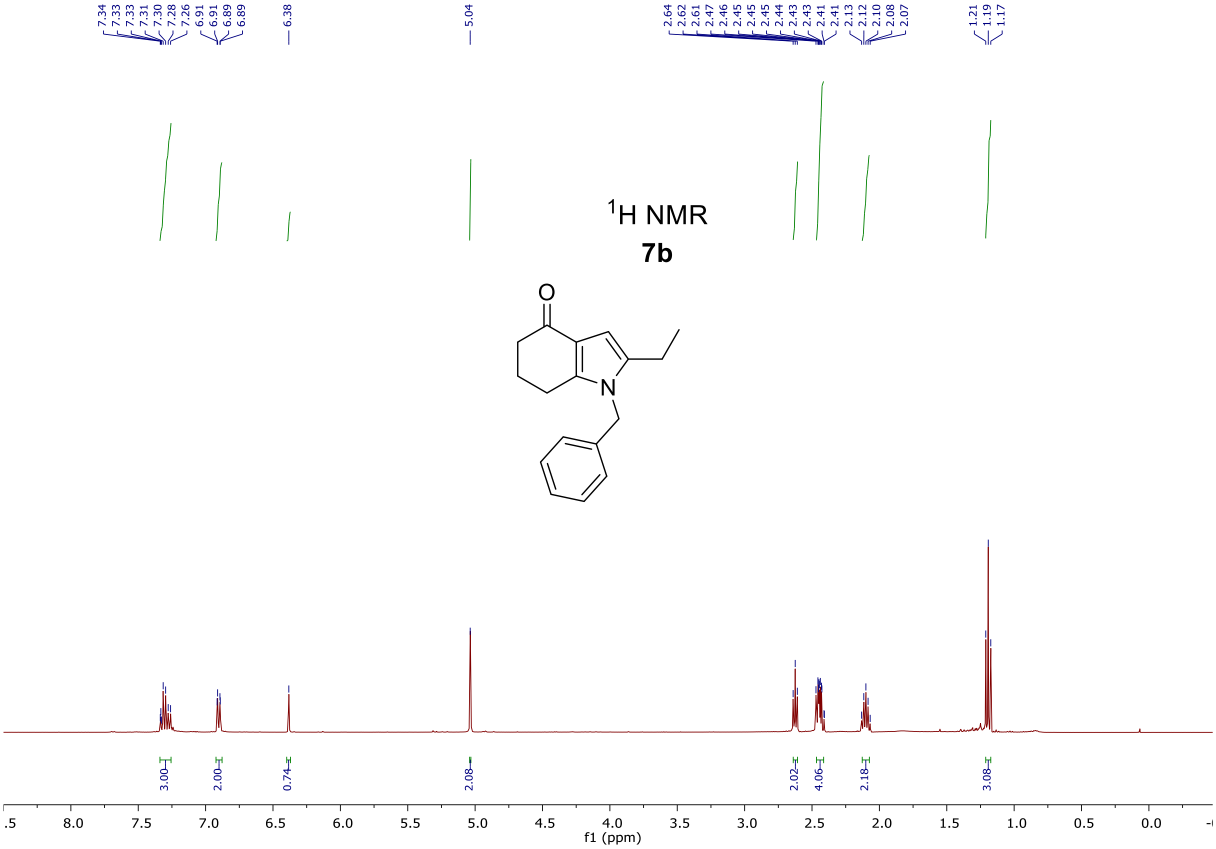

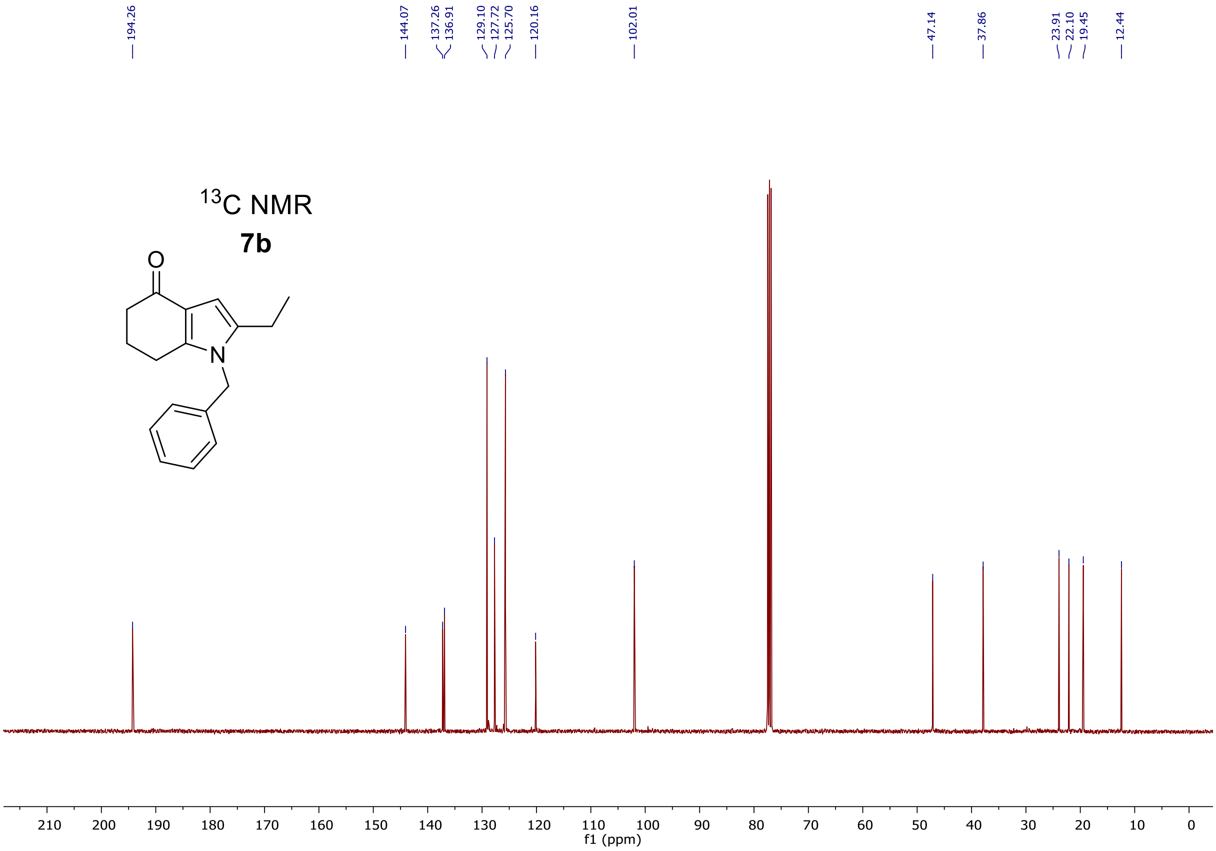

- ***2-Ethyl-1-phenethyl-1,5,6,7-tetrahydro-4H-indol-4-one (7c)***

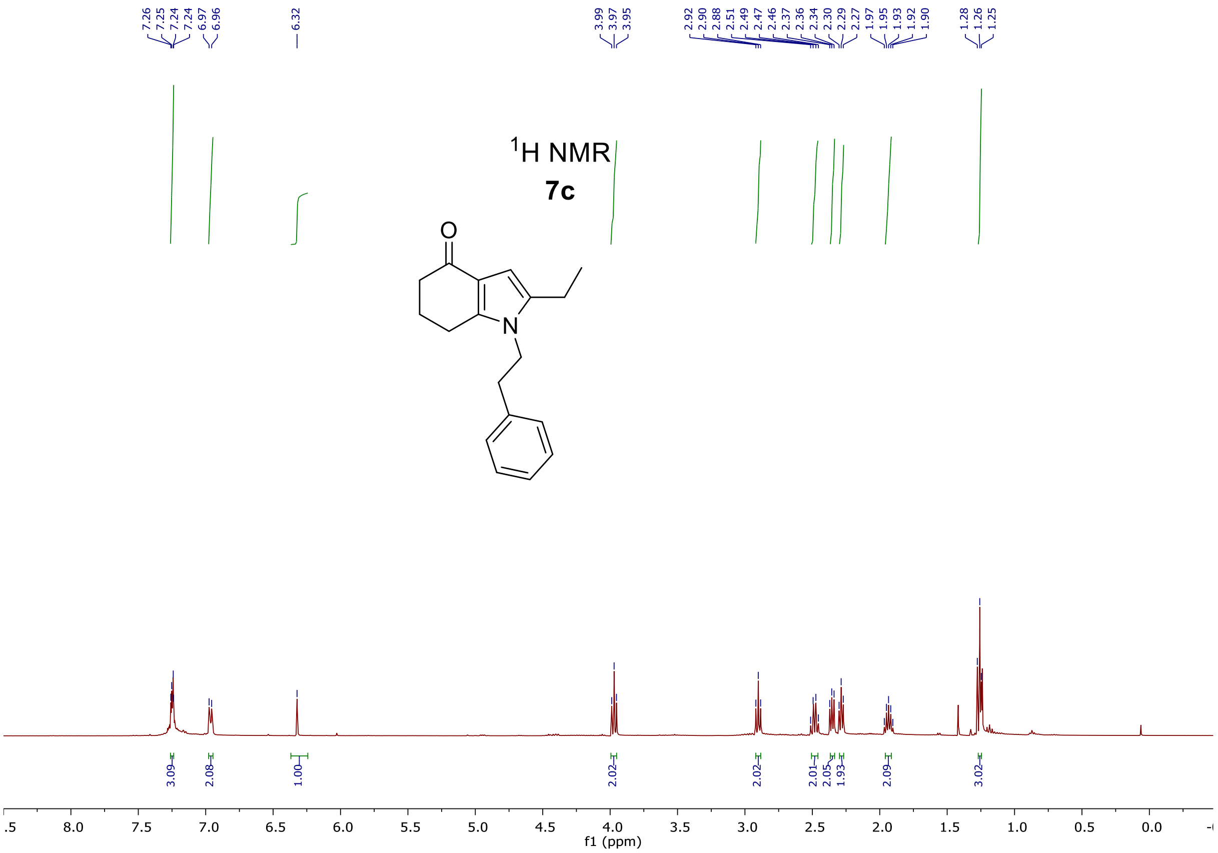

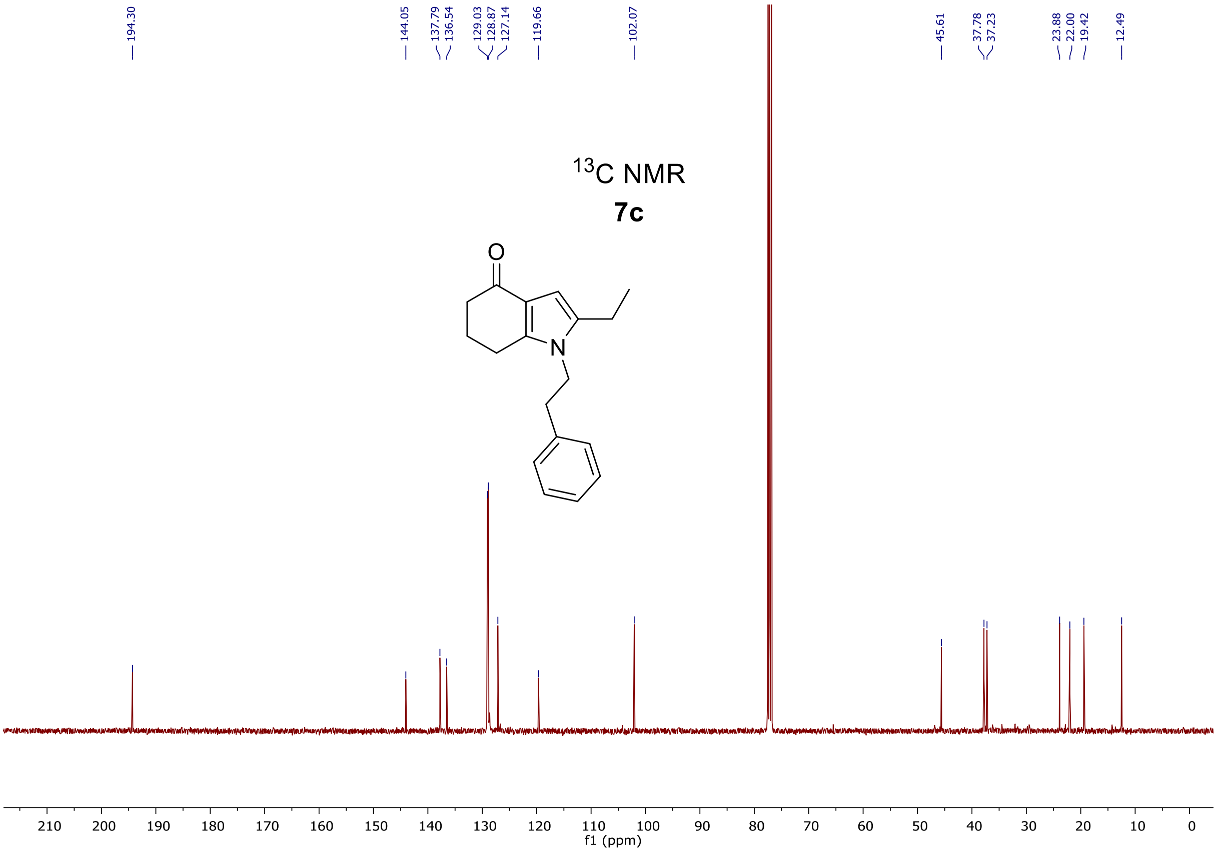

- ***2-Ethyl-1-(4-methoxybenzyl)-1,5,6,7-tetrahydro-4H-indol-4-one (7d)***

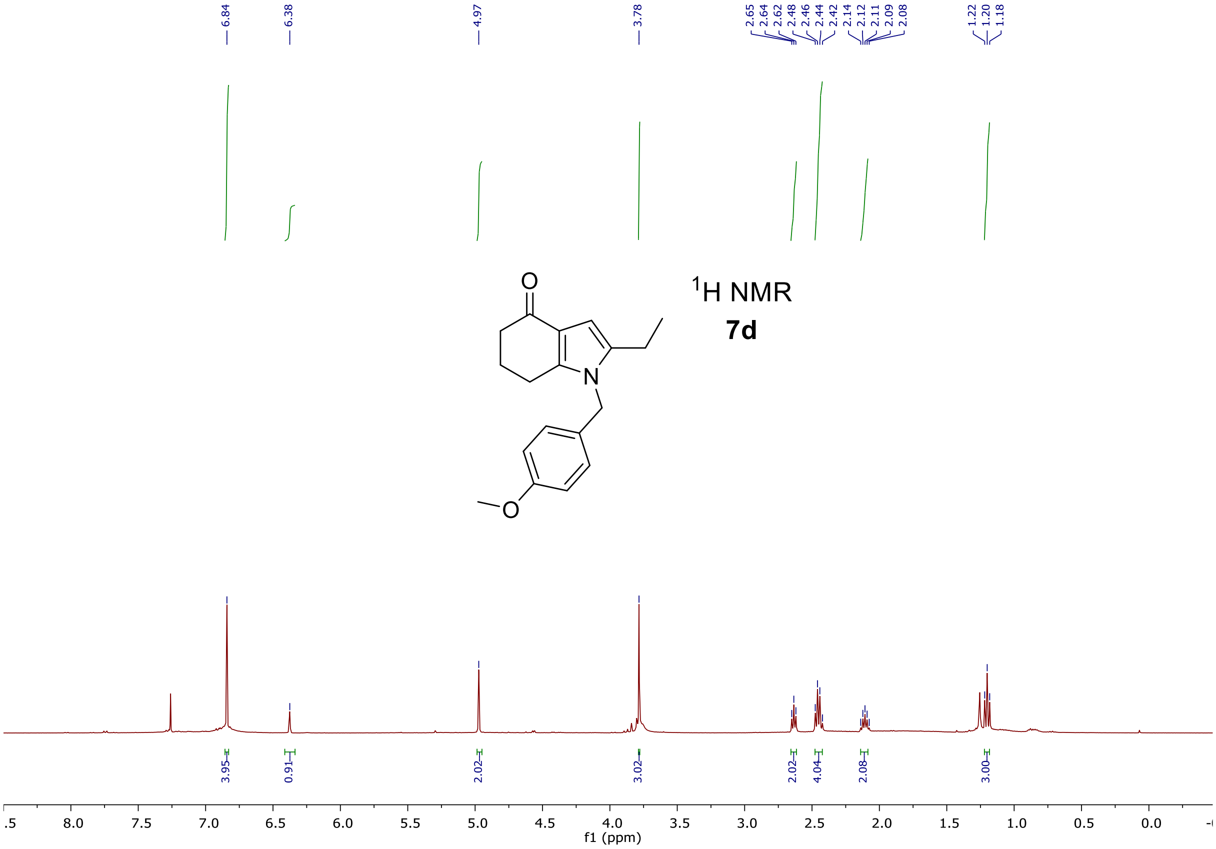

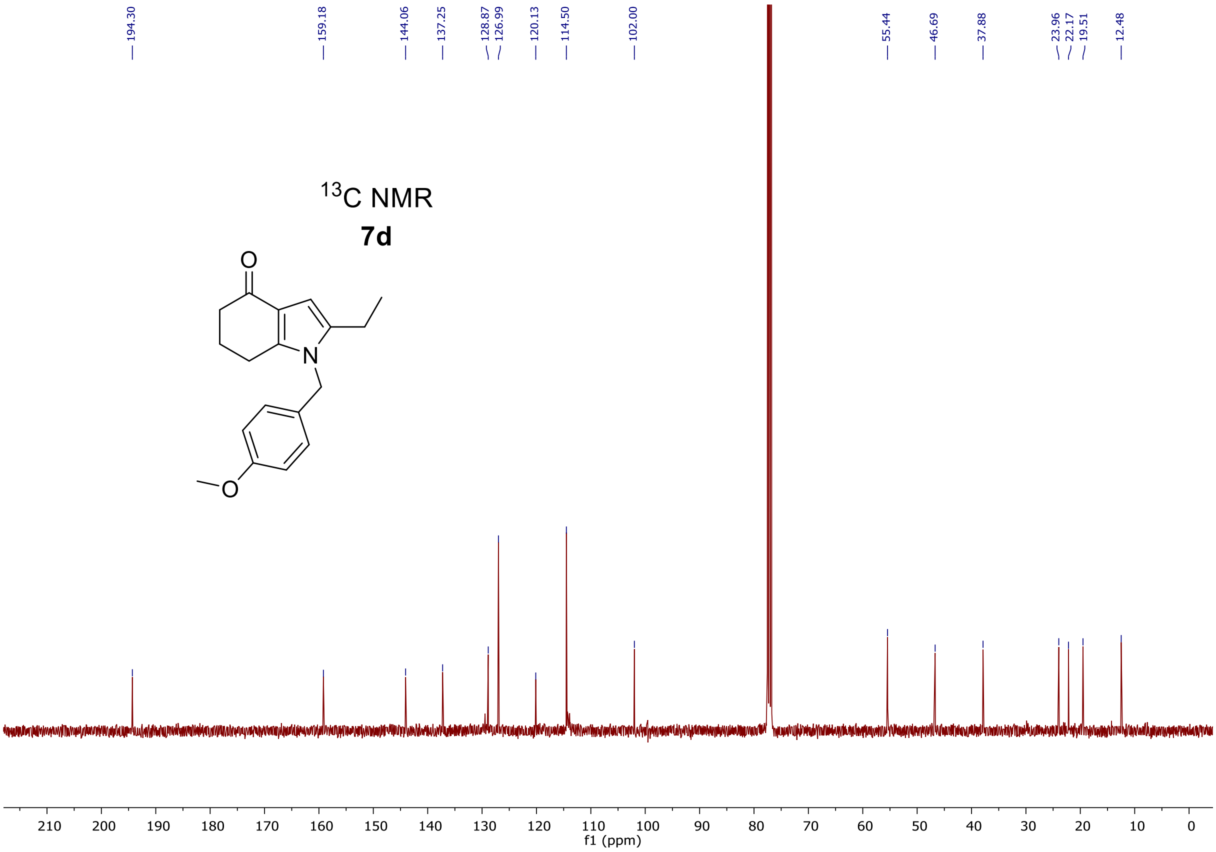

- ***2-Ethyl-1-(1-phenylethyl)-1,5,6,7-tetrahydro-4H-indol-4-one (7e)***

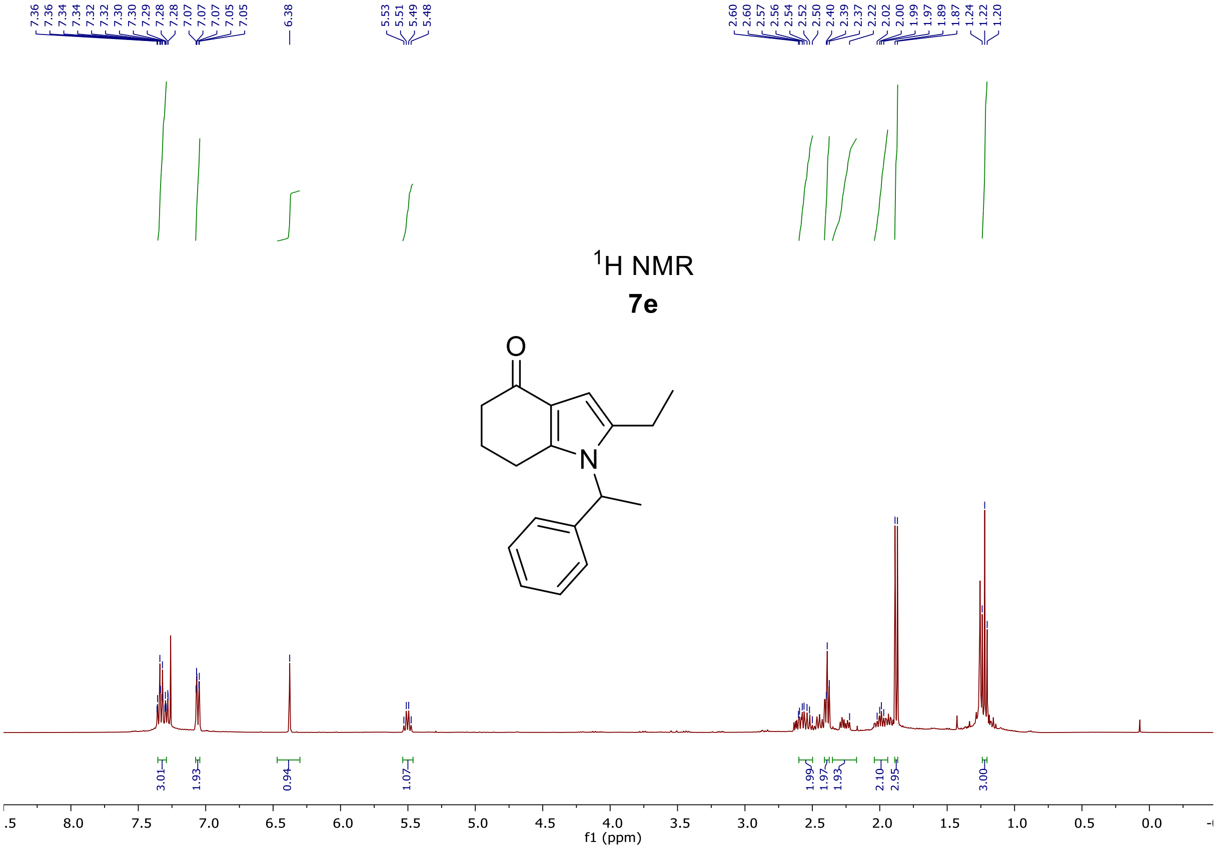

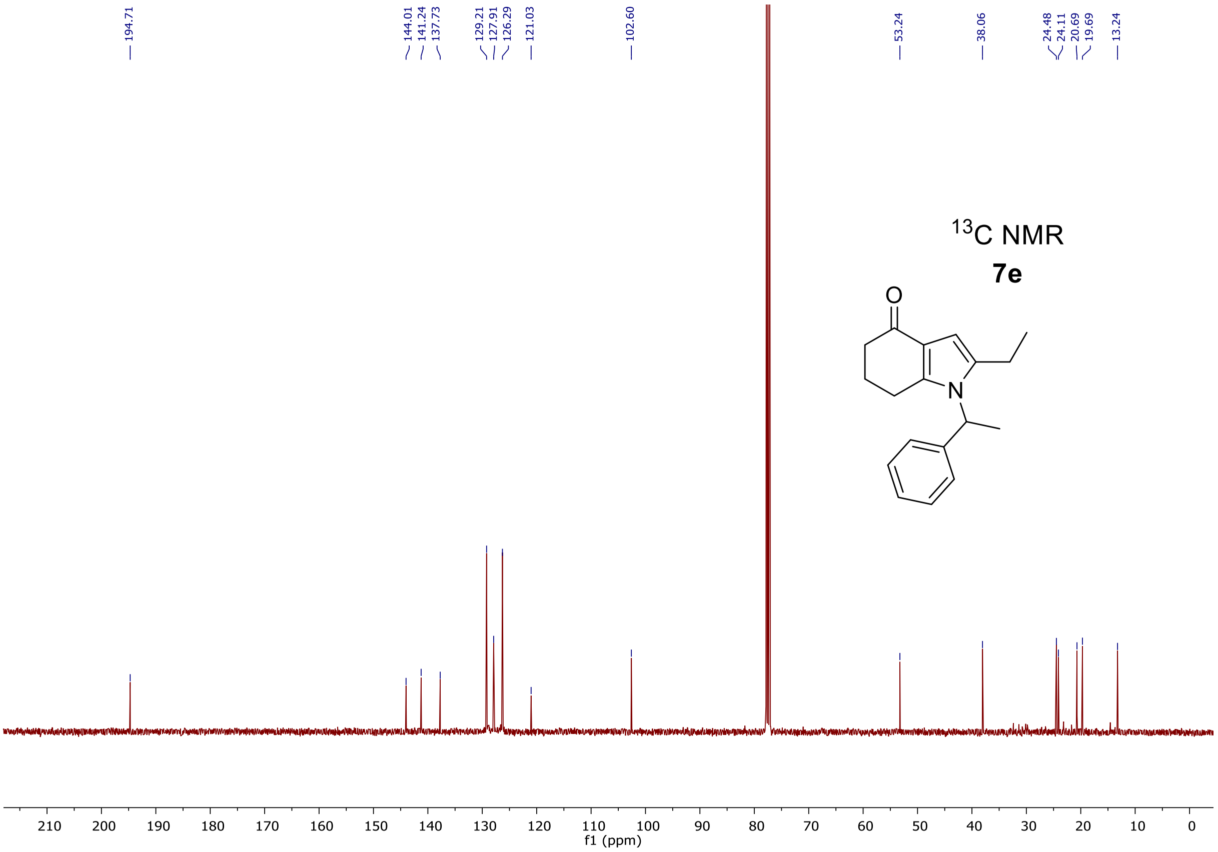

- ***2-Ethyl-1-(furan-3-ylmethyl)-1,5,6,7-tetrahydro-4H-indol-4-one (7f)***

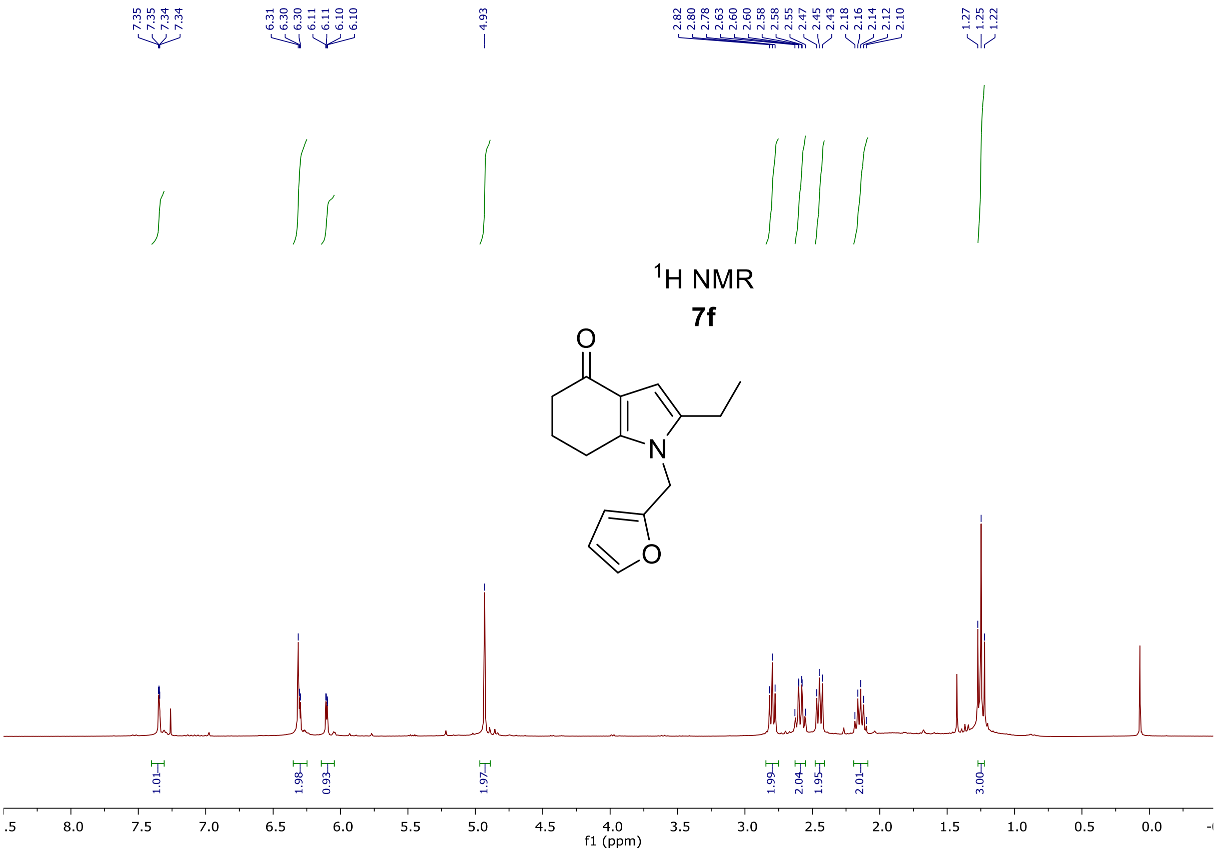

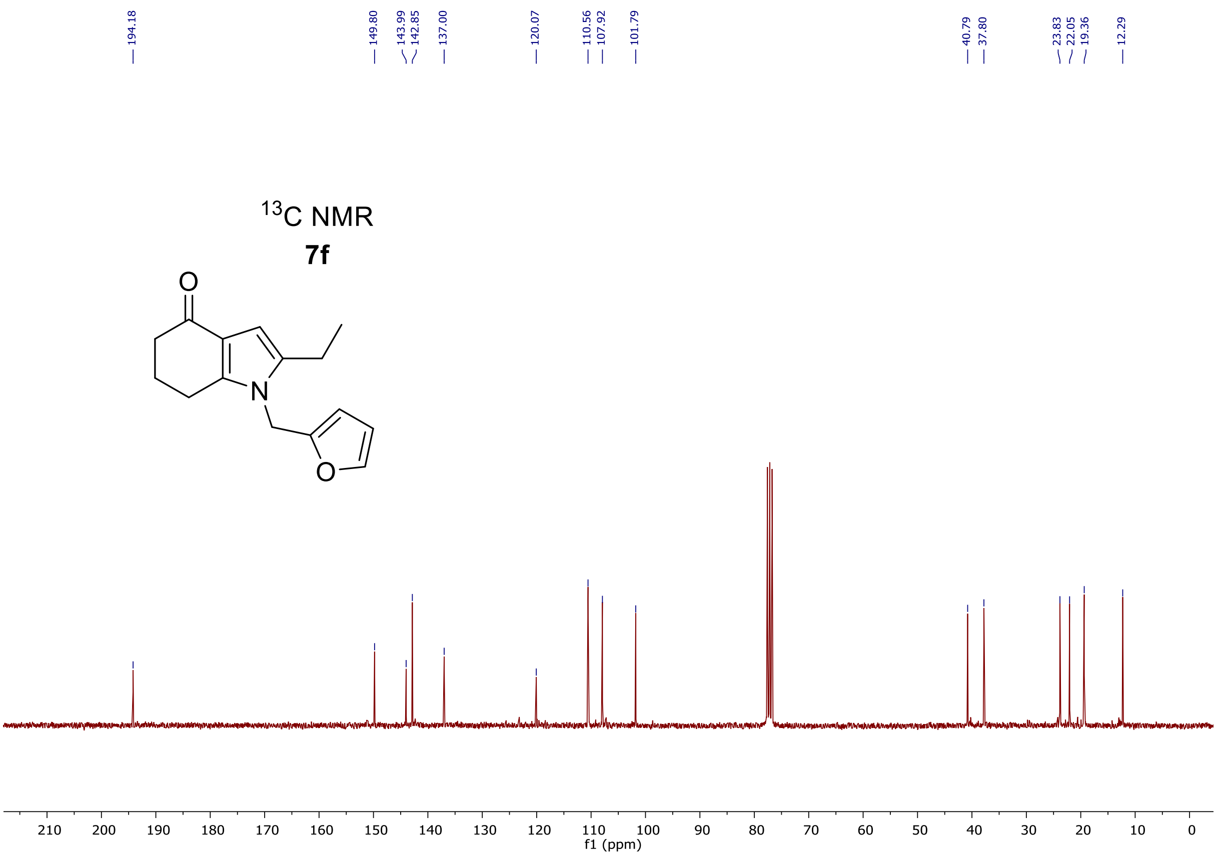

- ***1-(4-Bromobenzyl)-2-methyl-1,5,6,7-tetrahydro-4H-indol-4-one (7g)***

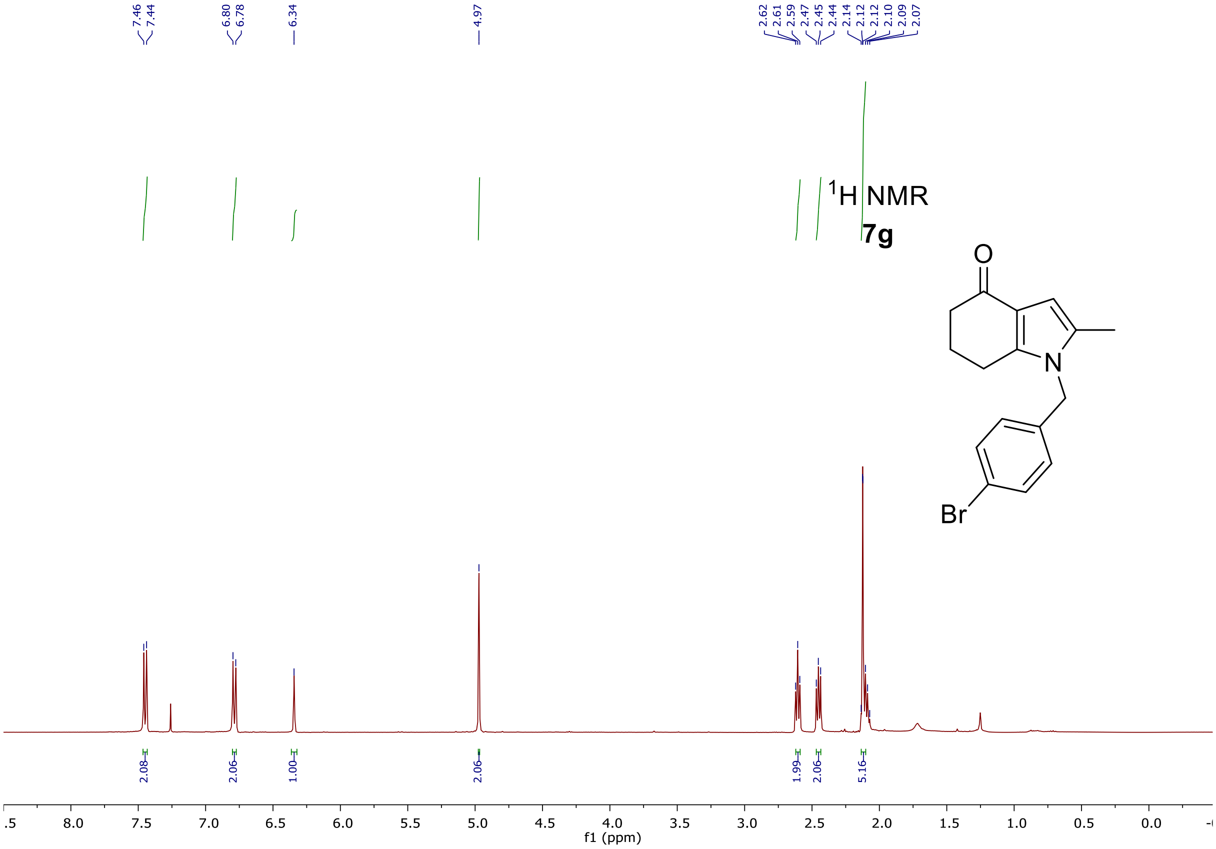

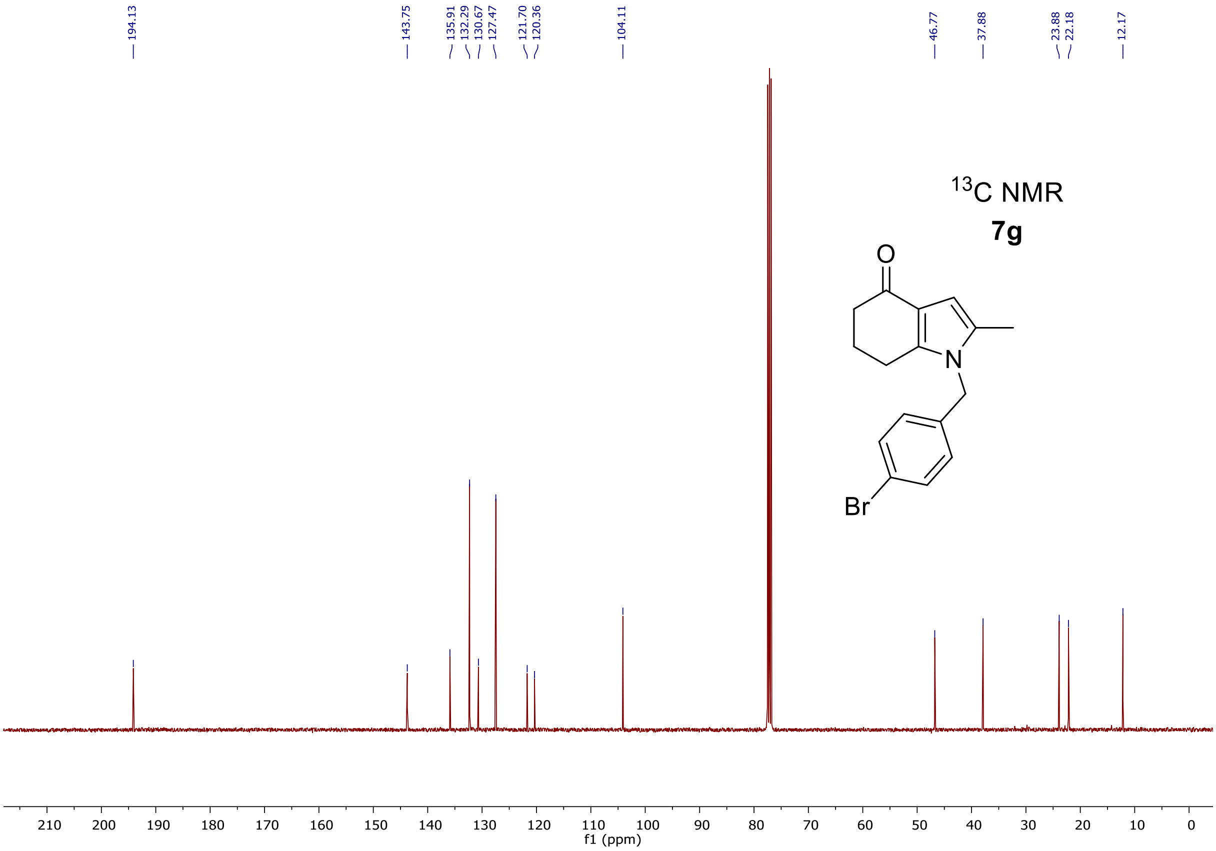

- ***1-(Furan-2-ylmethyl)-2-methyl-1,5,6,7-tetrahydro-4H-indol-4-one (7h)***

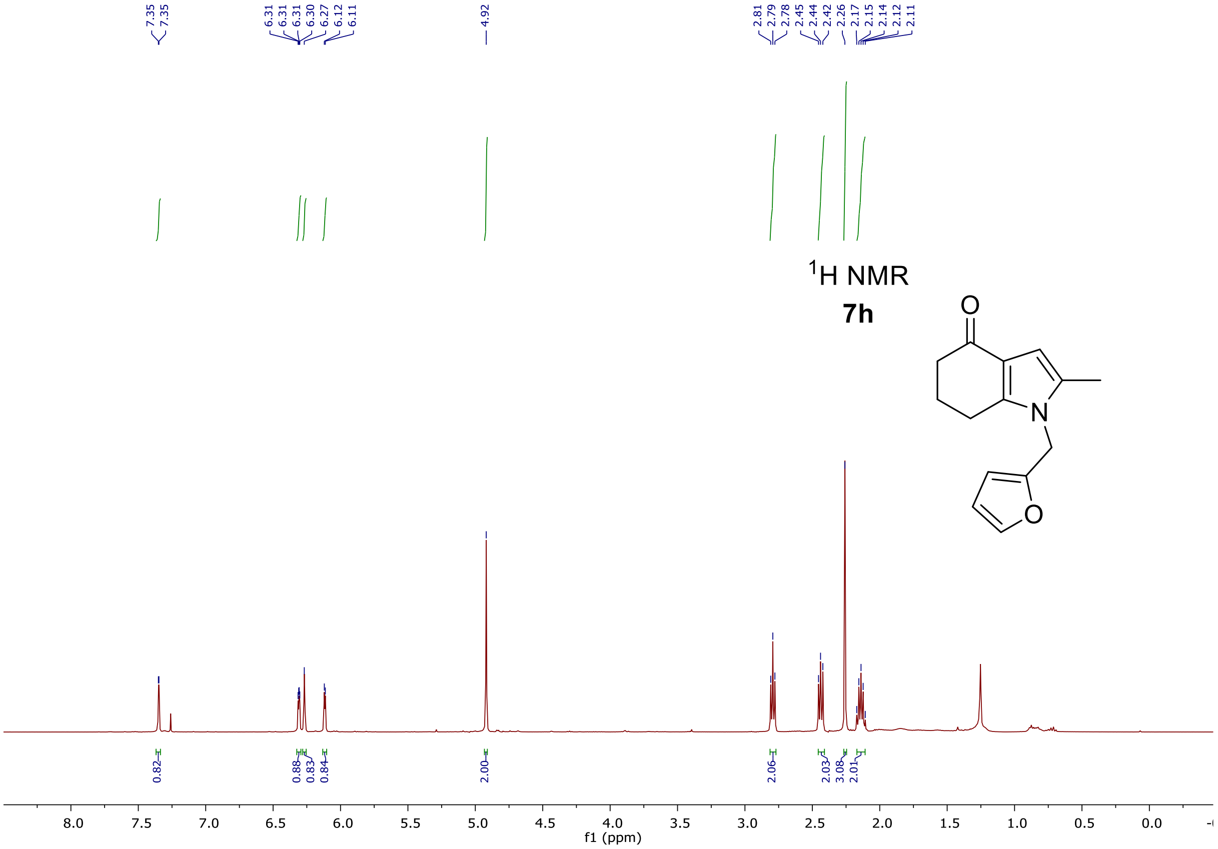

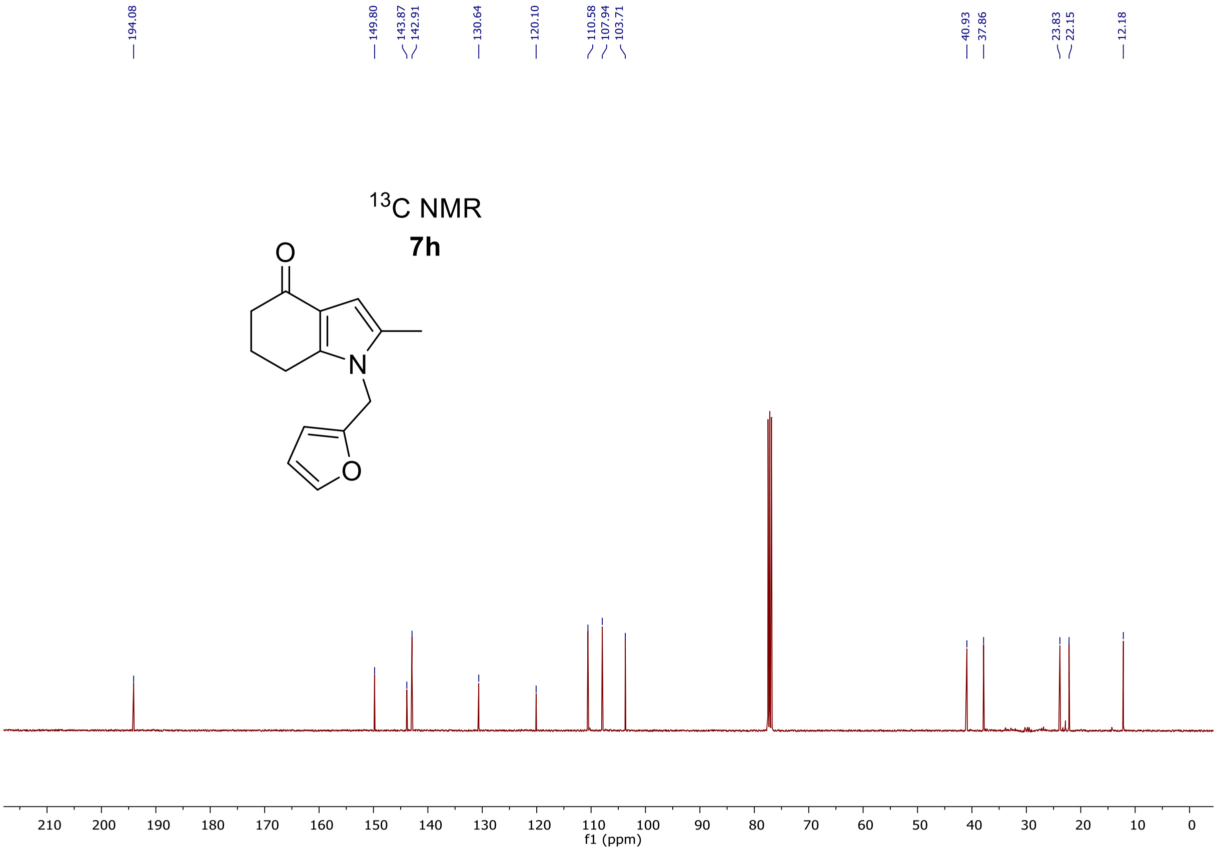

- ***1-(Pyridin-4-ylmethyl)-2-methyl-1,5,6,7-tetrahydro-4H-indol-4-one (7i)***

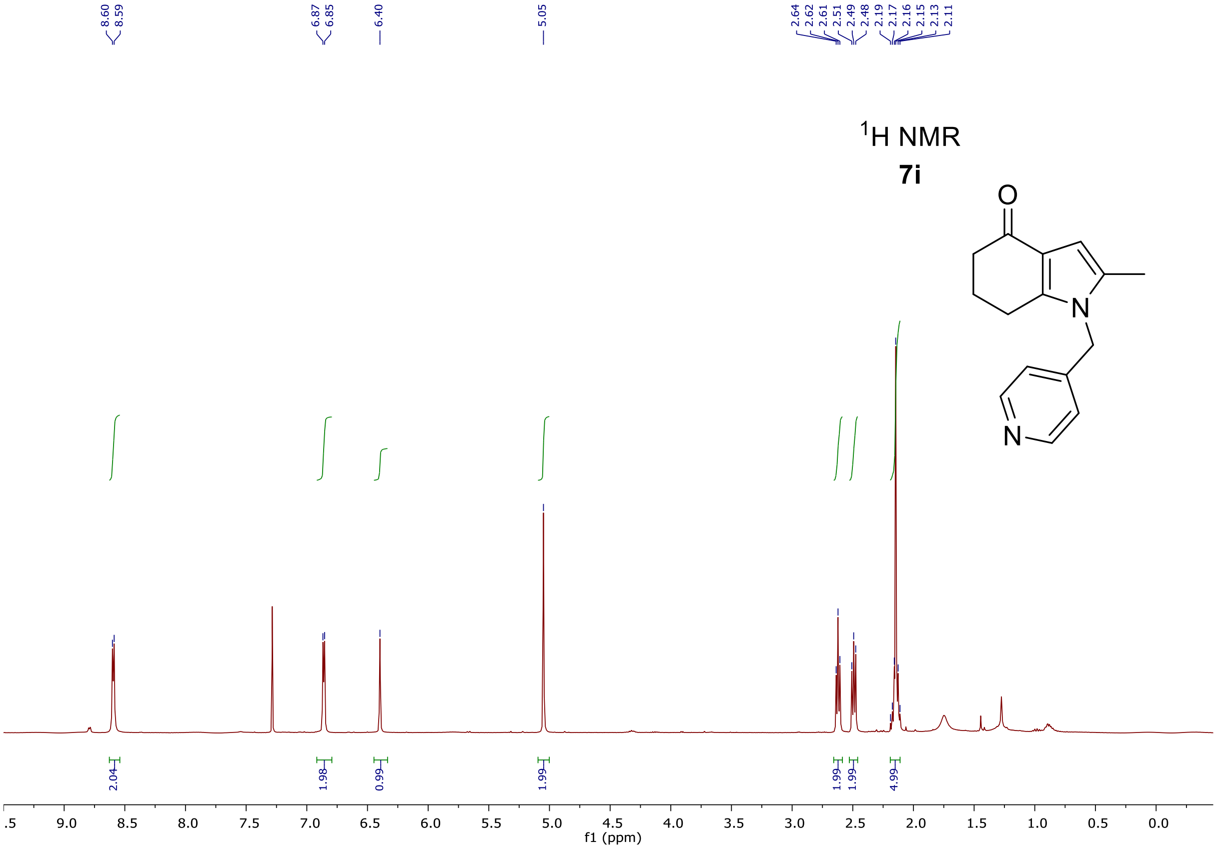

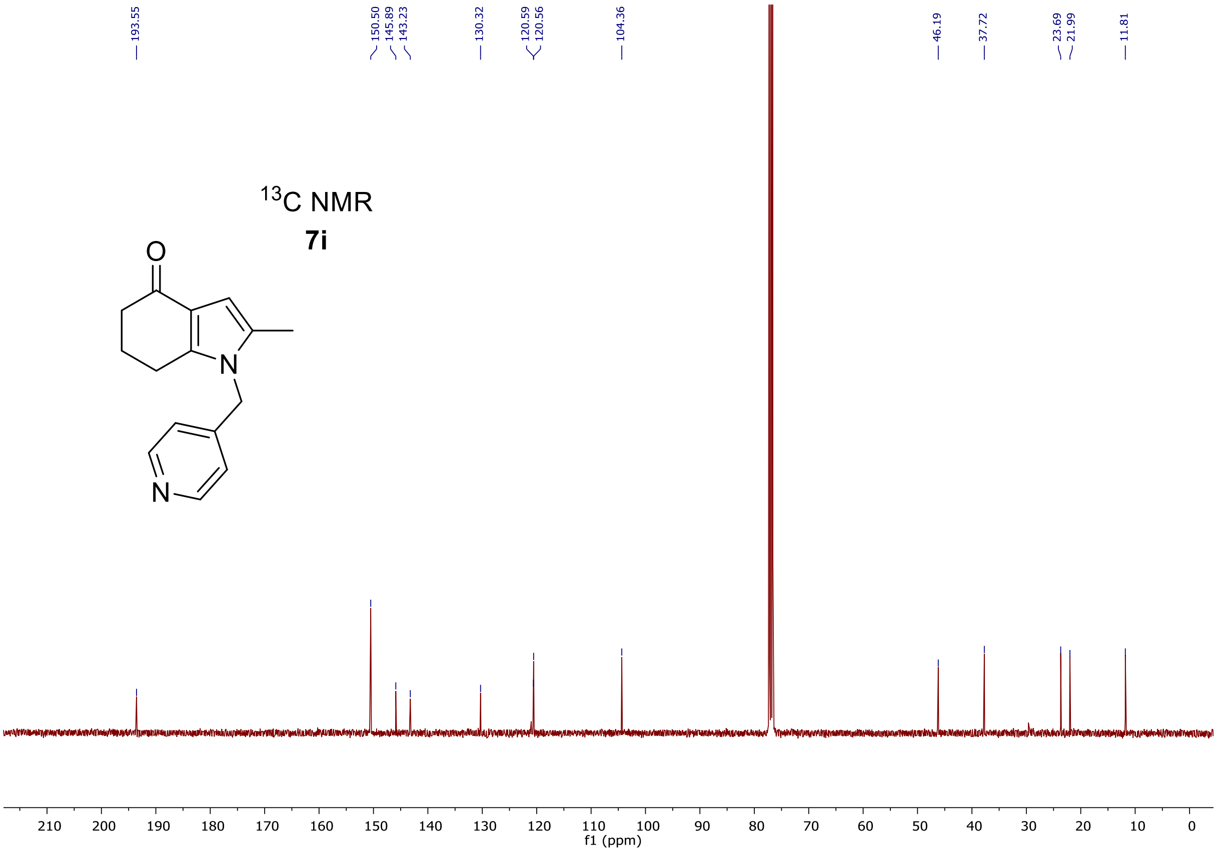

- ***2-Methyl-1-phenethyl-1,5,6,7-tetrahydro-4H-indol-4-one (7j)***

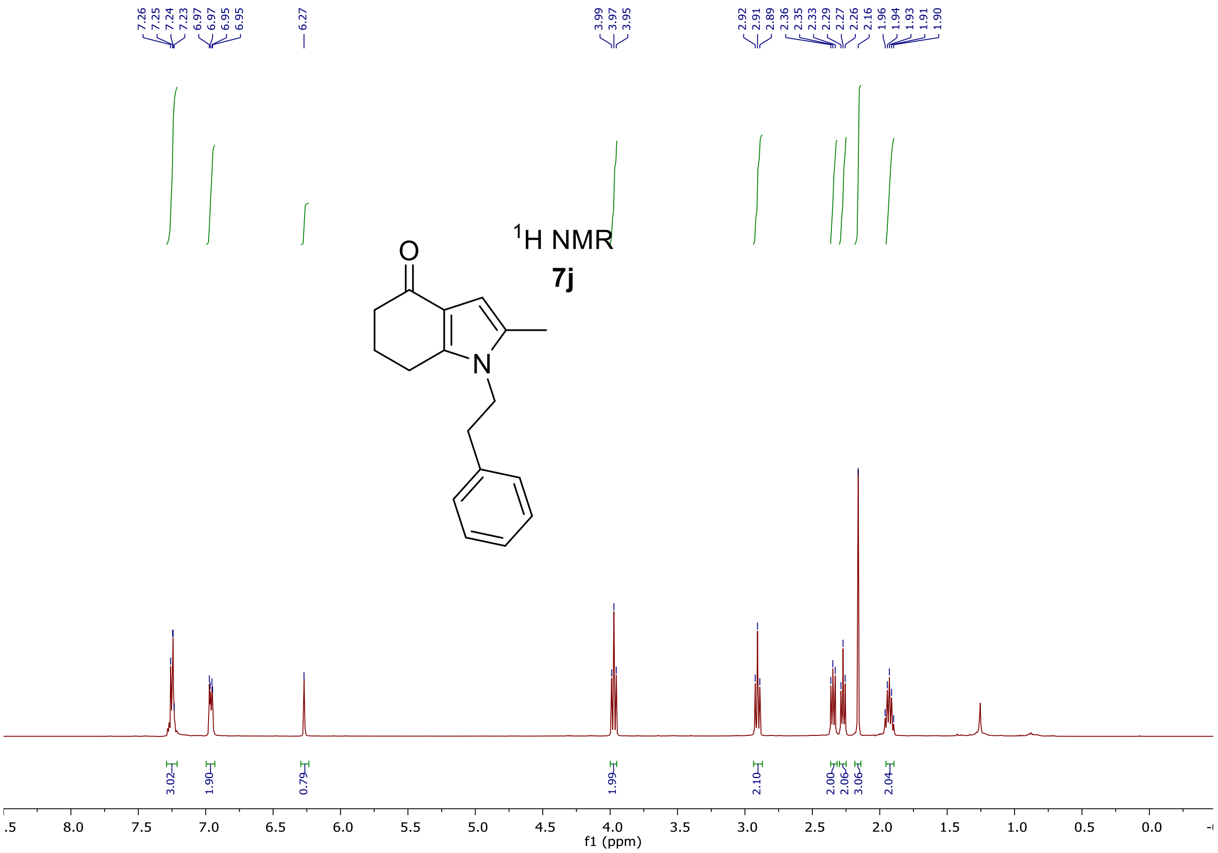

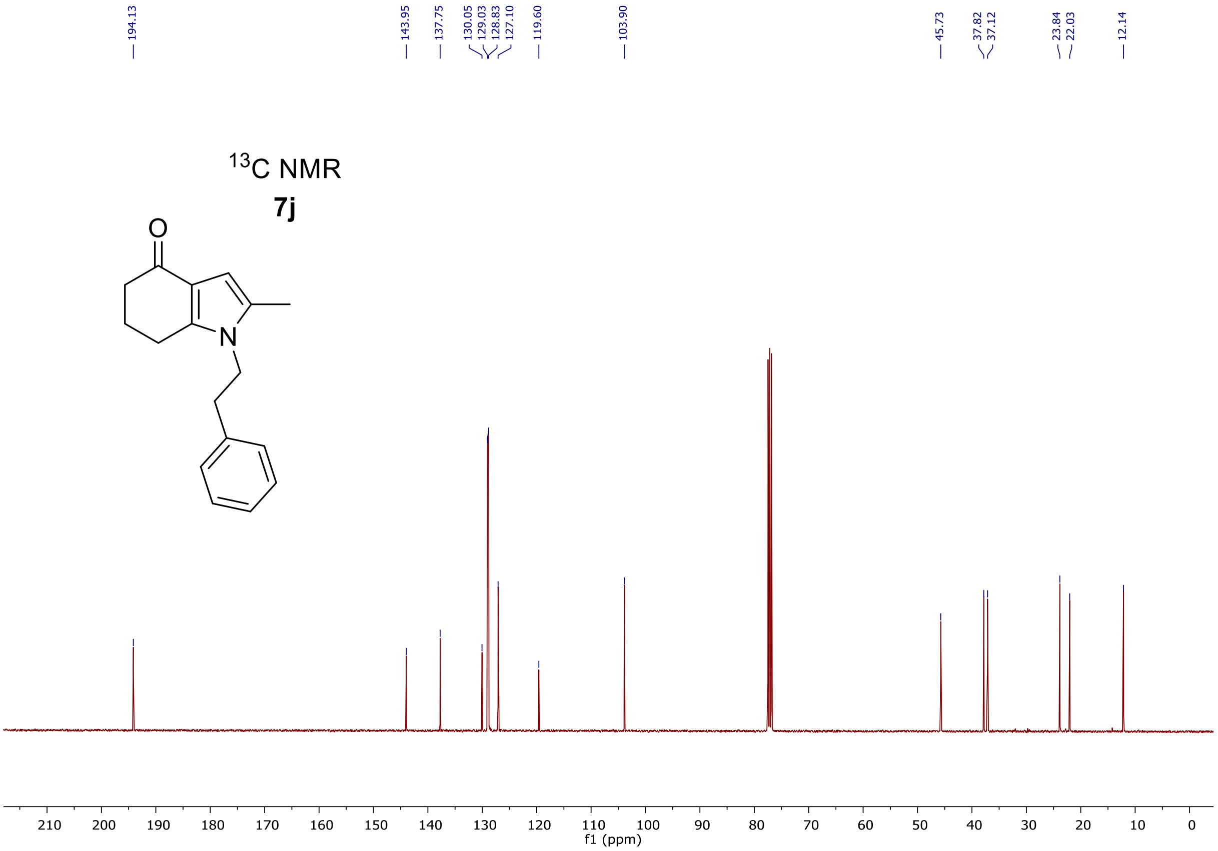

- ***1-Phenethyl-2-phenyl-1,5,6,7-tetrahydro-4H-indol-4-one (7k)***

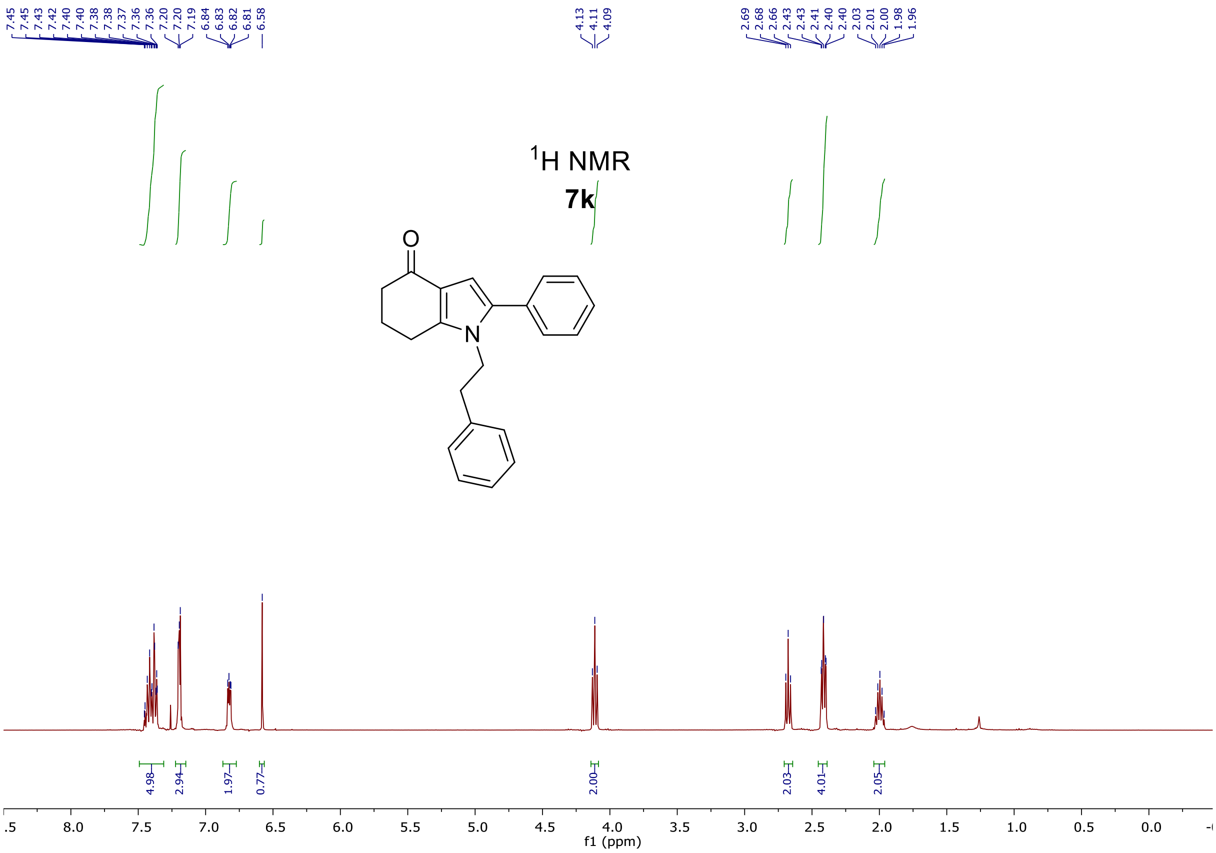

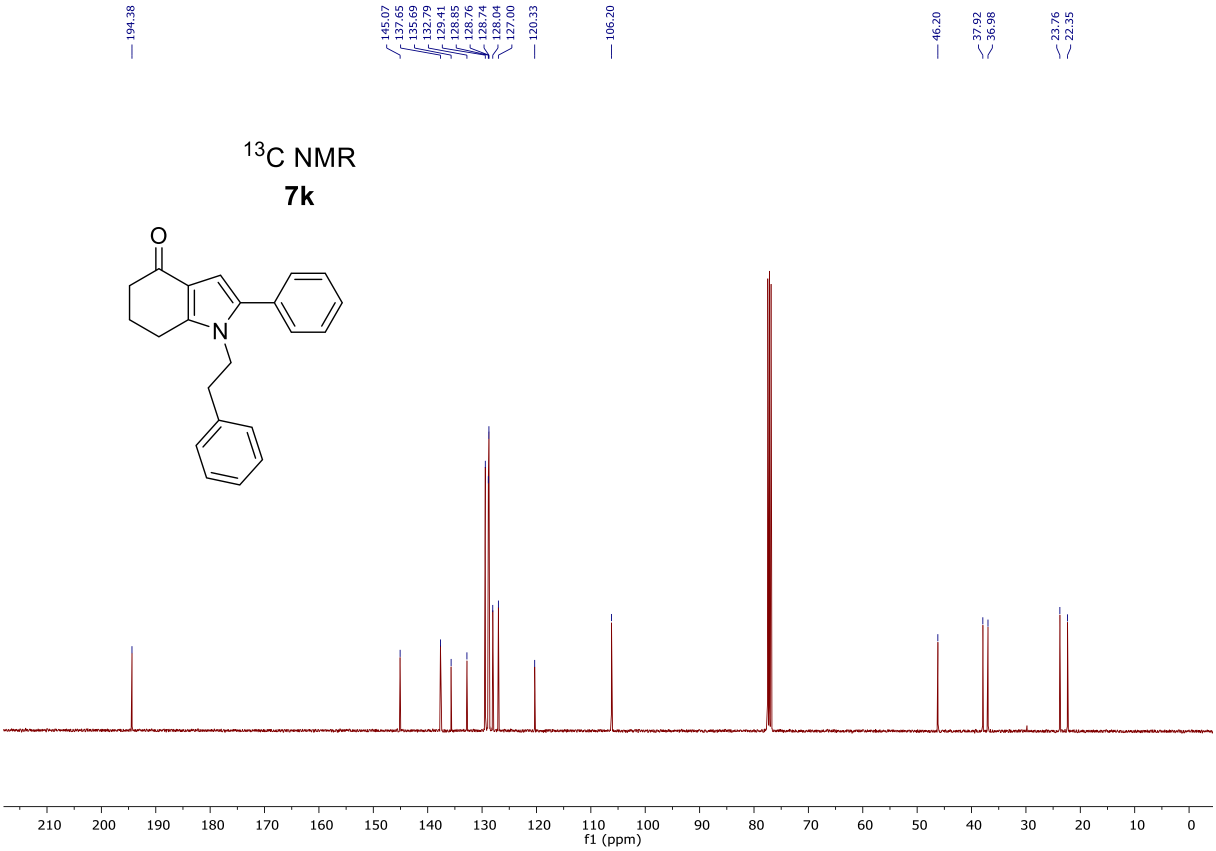

f. 2-ethyl-1-(3-(2-ethyl-1*H*-indol-1-yl)propyl)-1,5,6,7-tetrahydro-4*H*-indol-4-one (9).

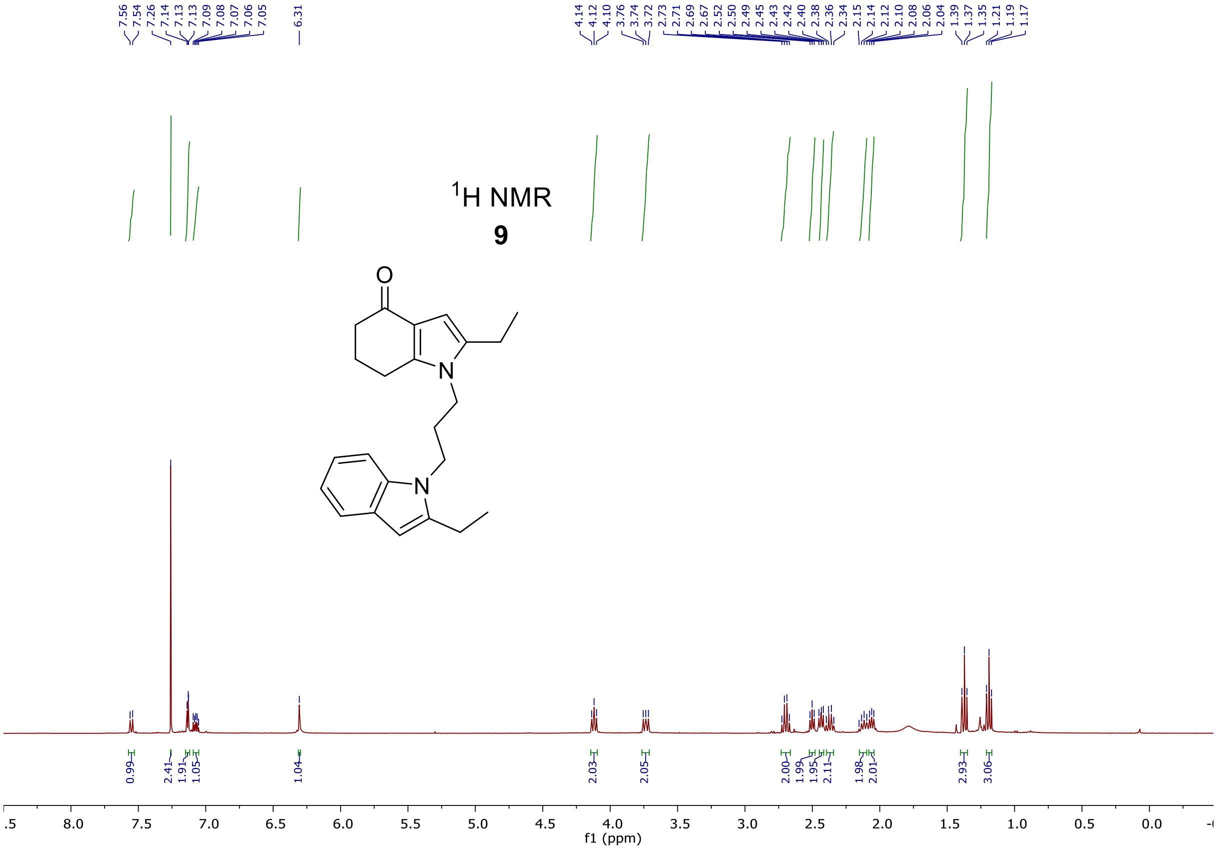

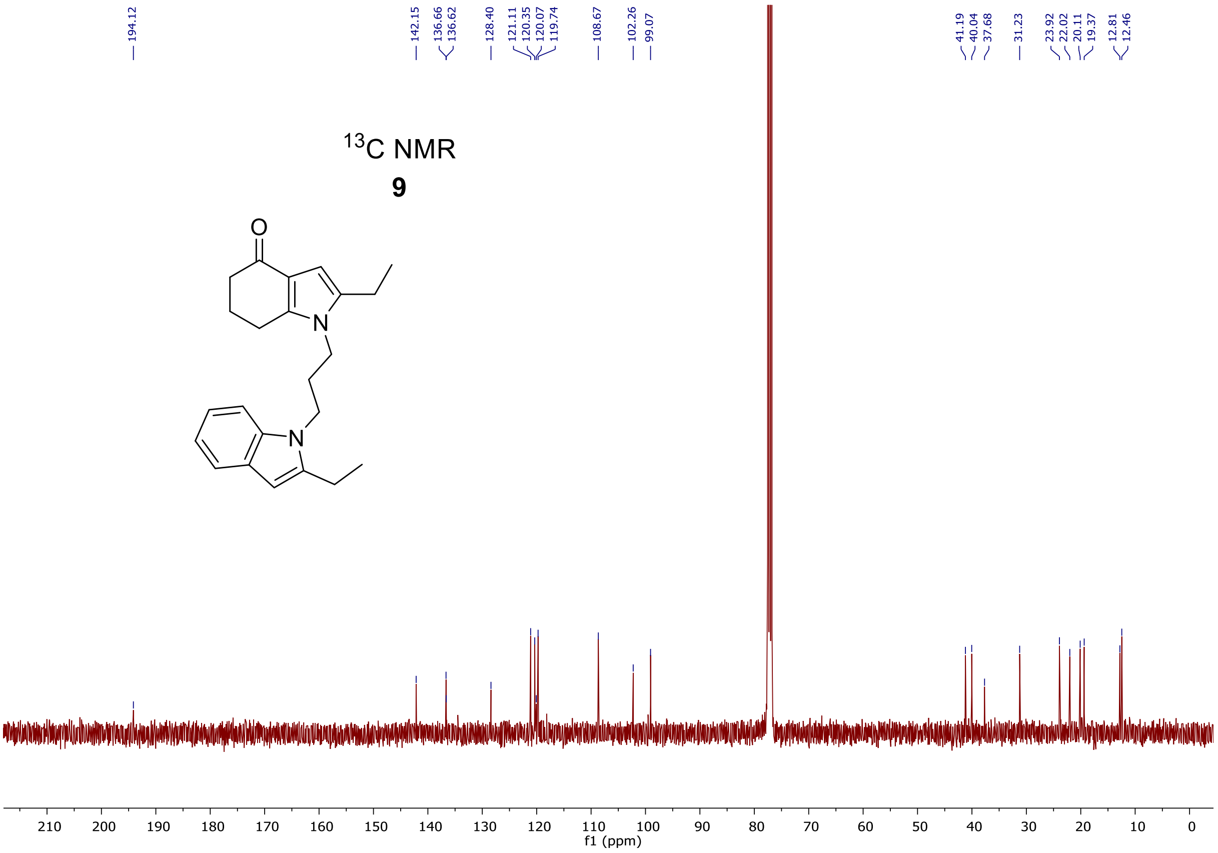

g. Cinnolines 8a–k.

- ***3-Methyl-5,6,7,8-tetrahydrocinnoline (8a)***

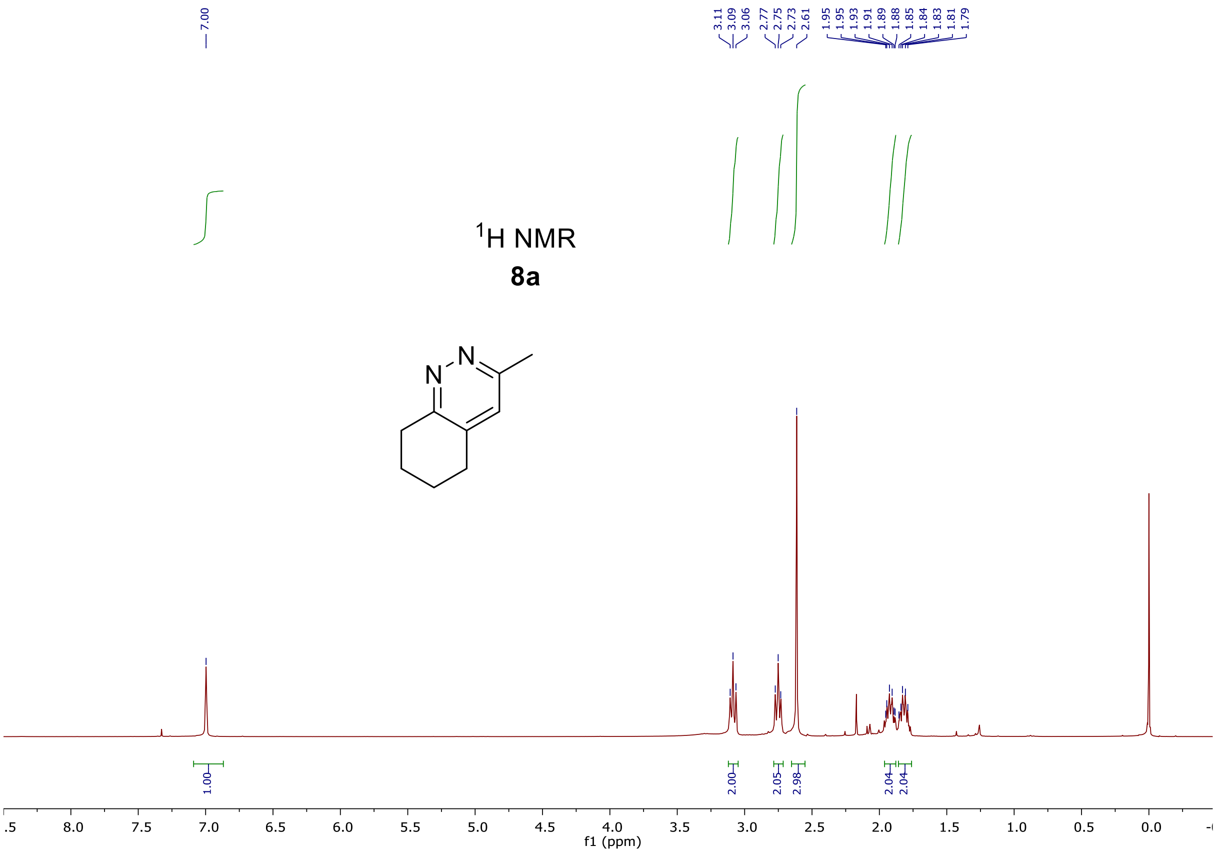

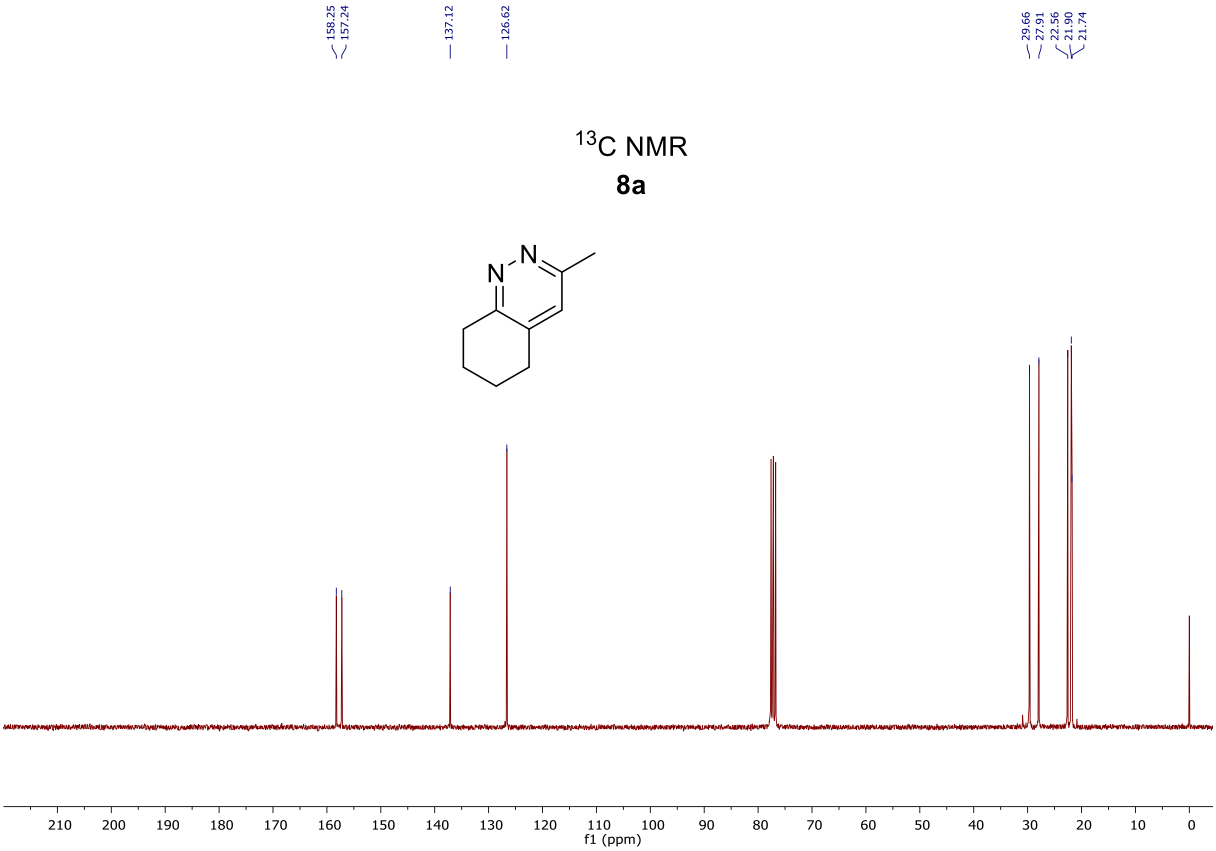

- ***3-Ethyl-5,6,7,8-tetrahydrocinnoline (8b)***

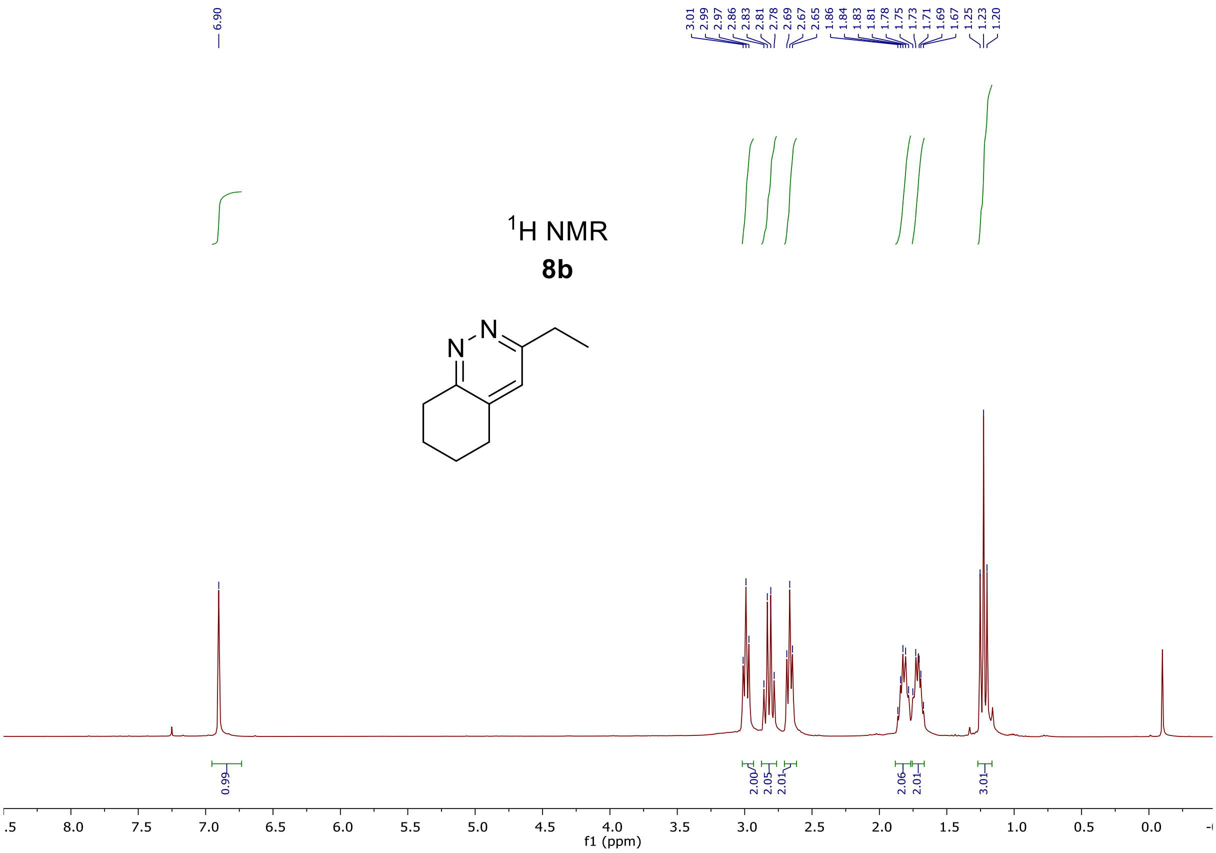

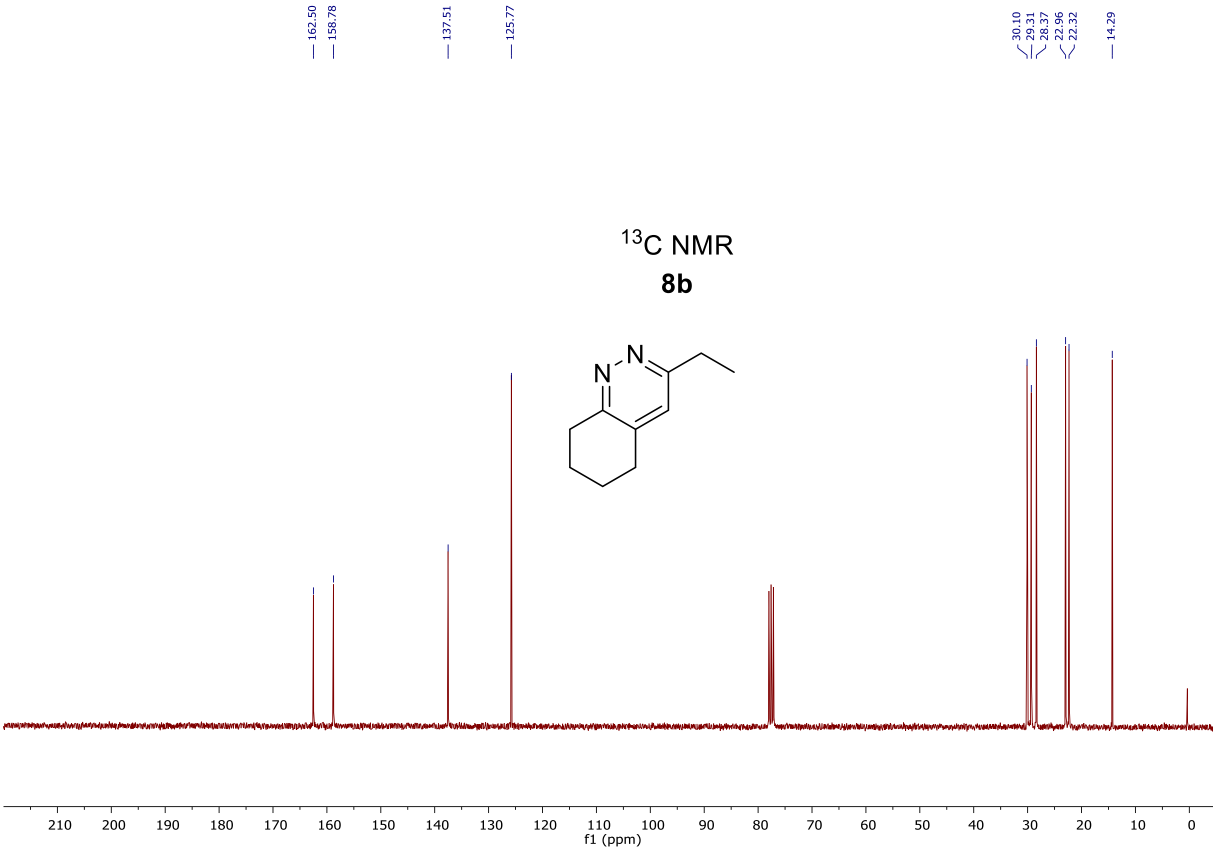

- ***3,6,6-Trimethyl-5,6,7,8-tetrahydrocinnoline (8c)***

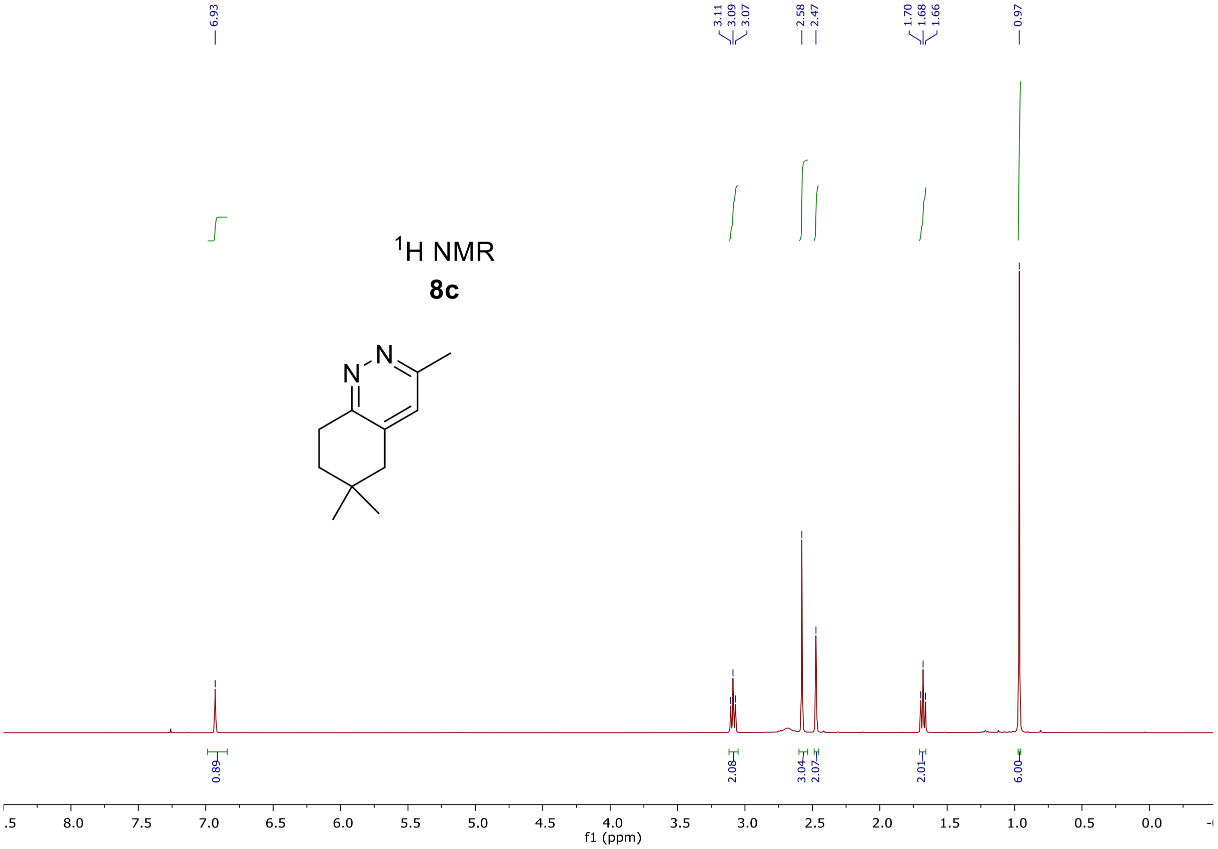

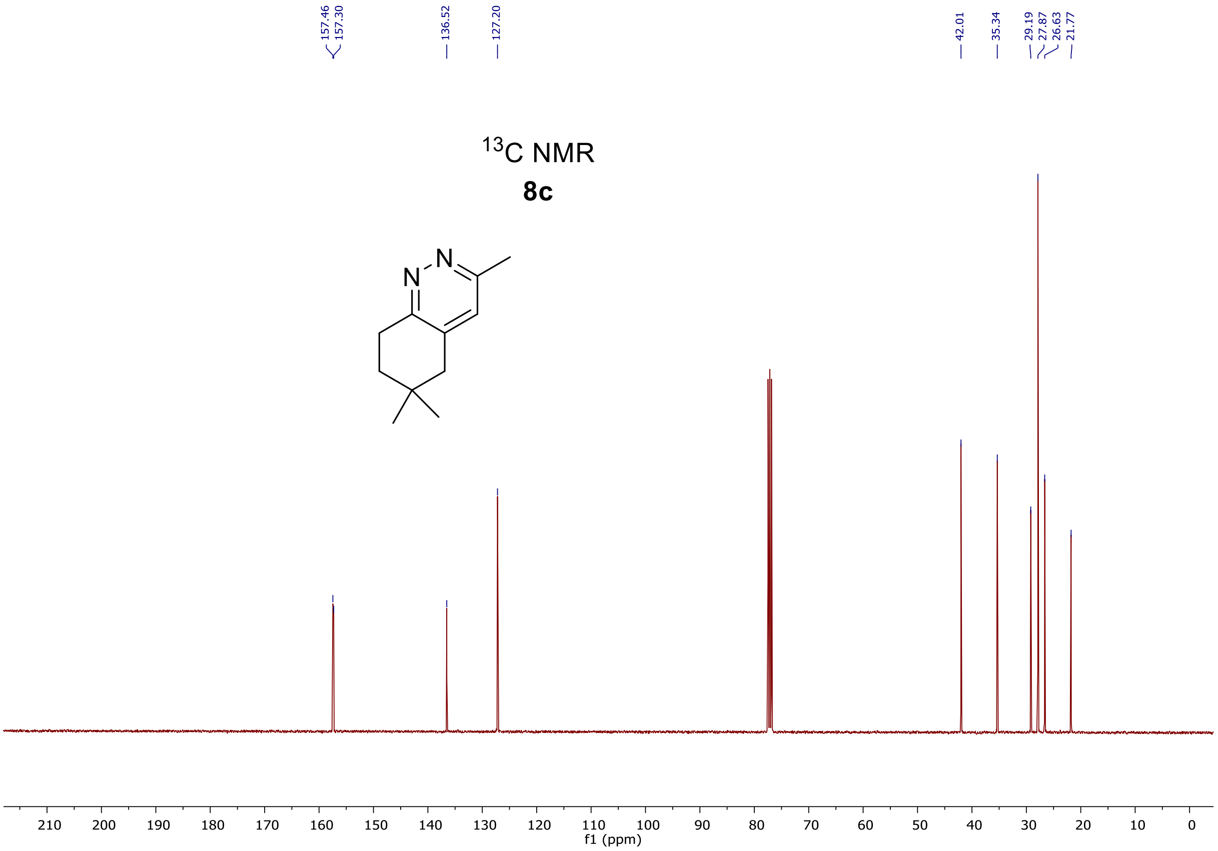

- ***3-Ethyl-6,6-dimethyl-5,6,7,8-tetrahydrocinnoline (8d)***

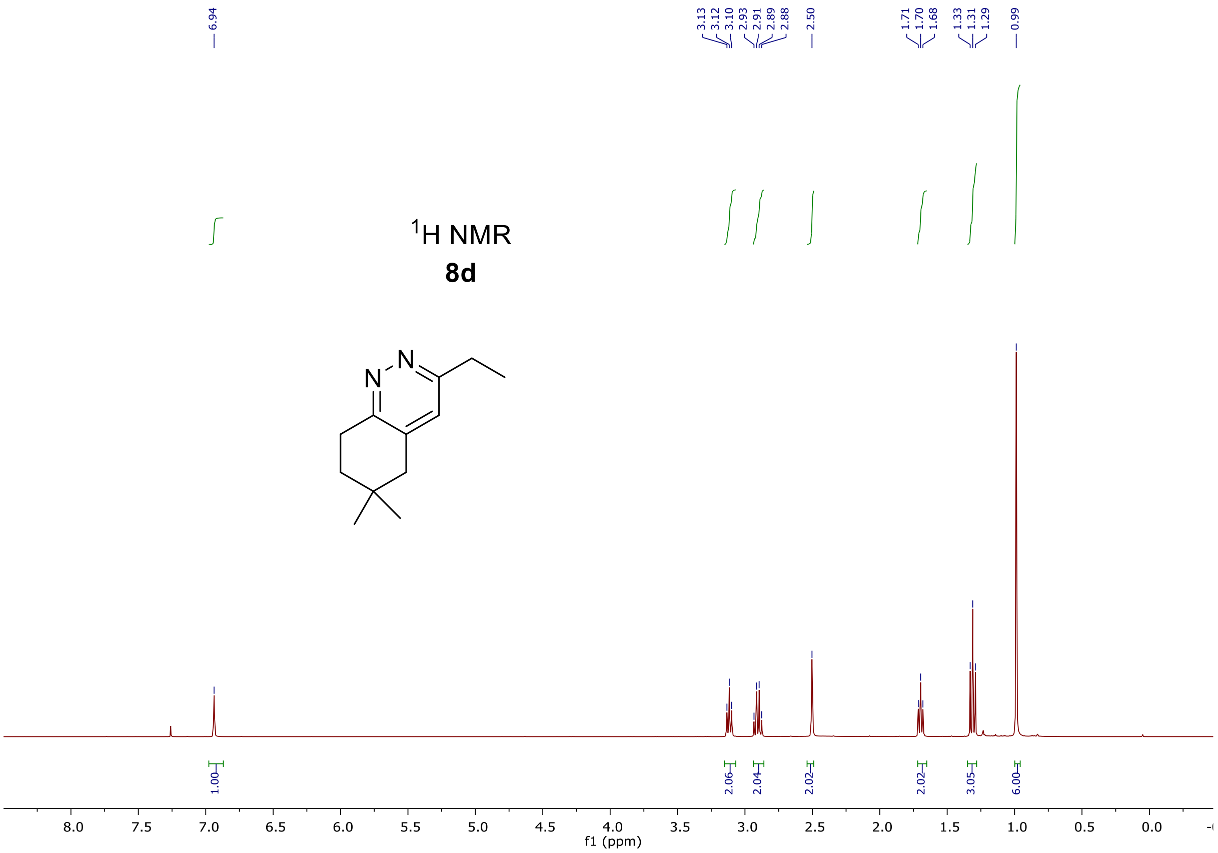

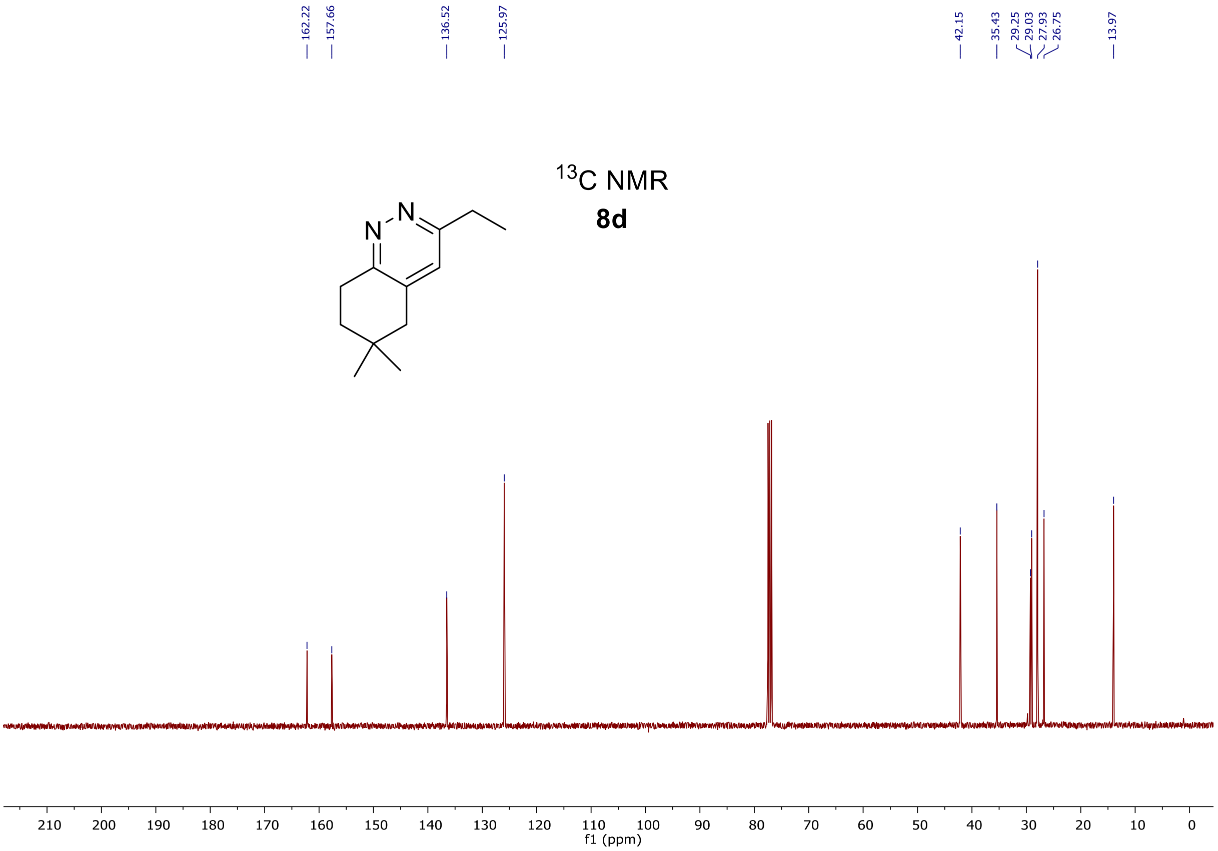

- ***3-(tert-Butyl)-5,6,7,8-tetrahydrocinnoline (8e)***

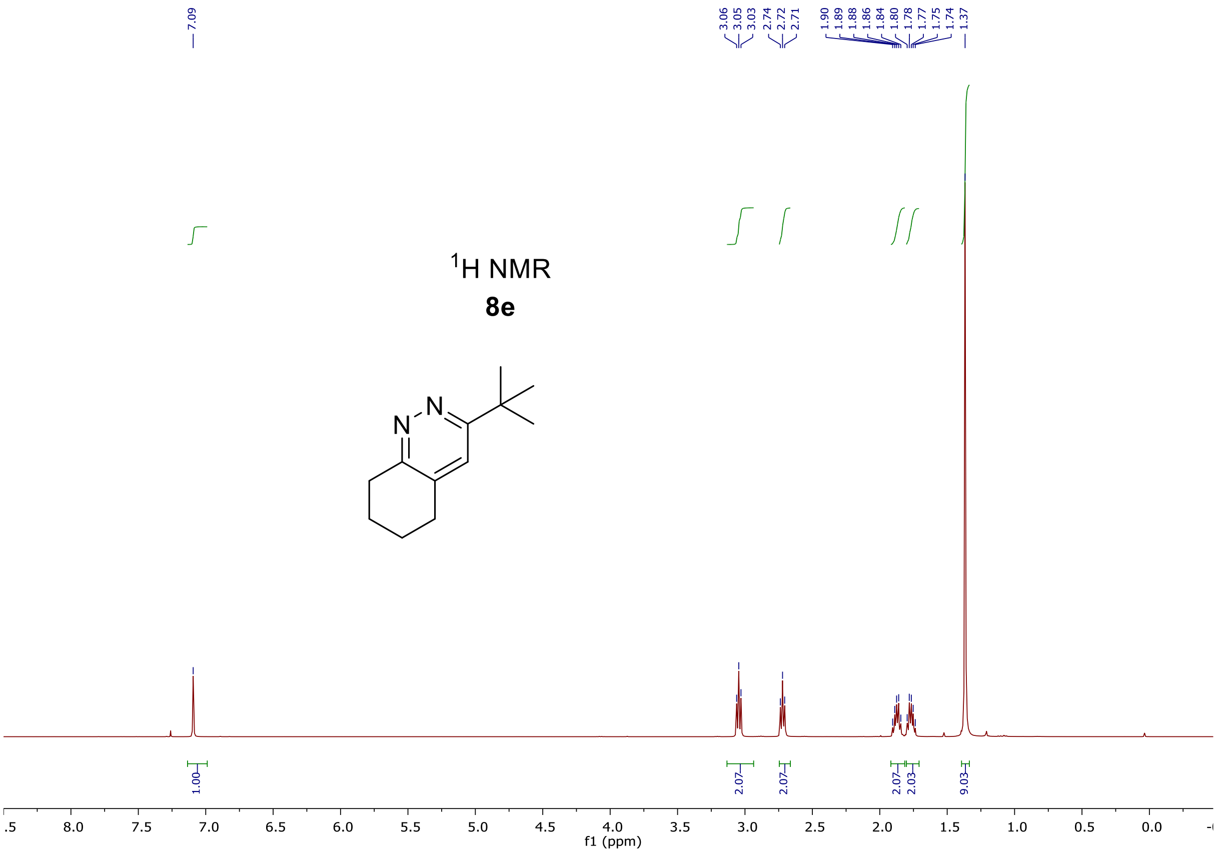

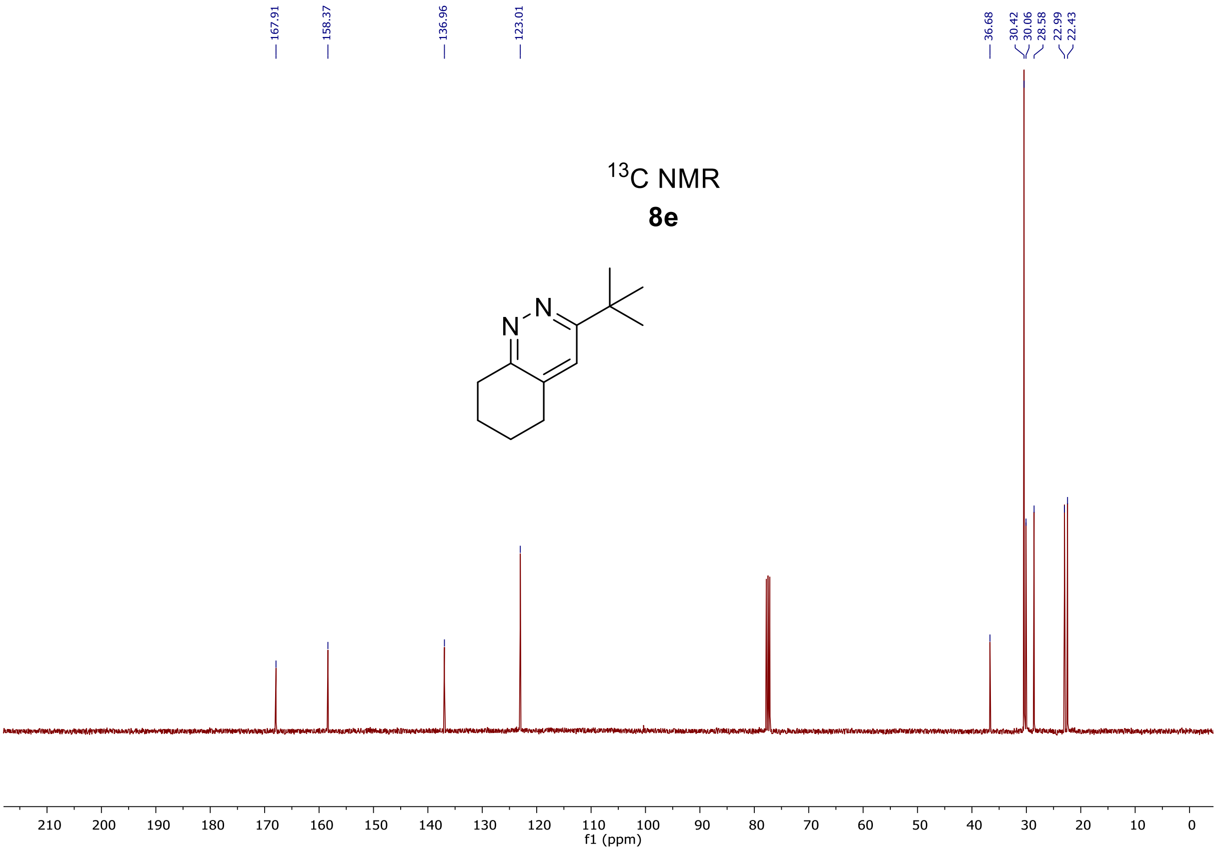

- ***3-Phenyl-5,6,7,8-tetrahydrocinnoline (8f)***

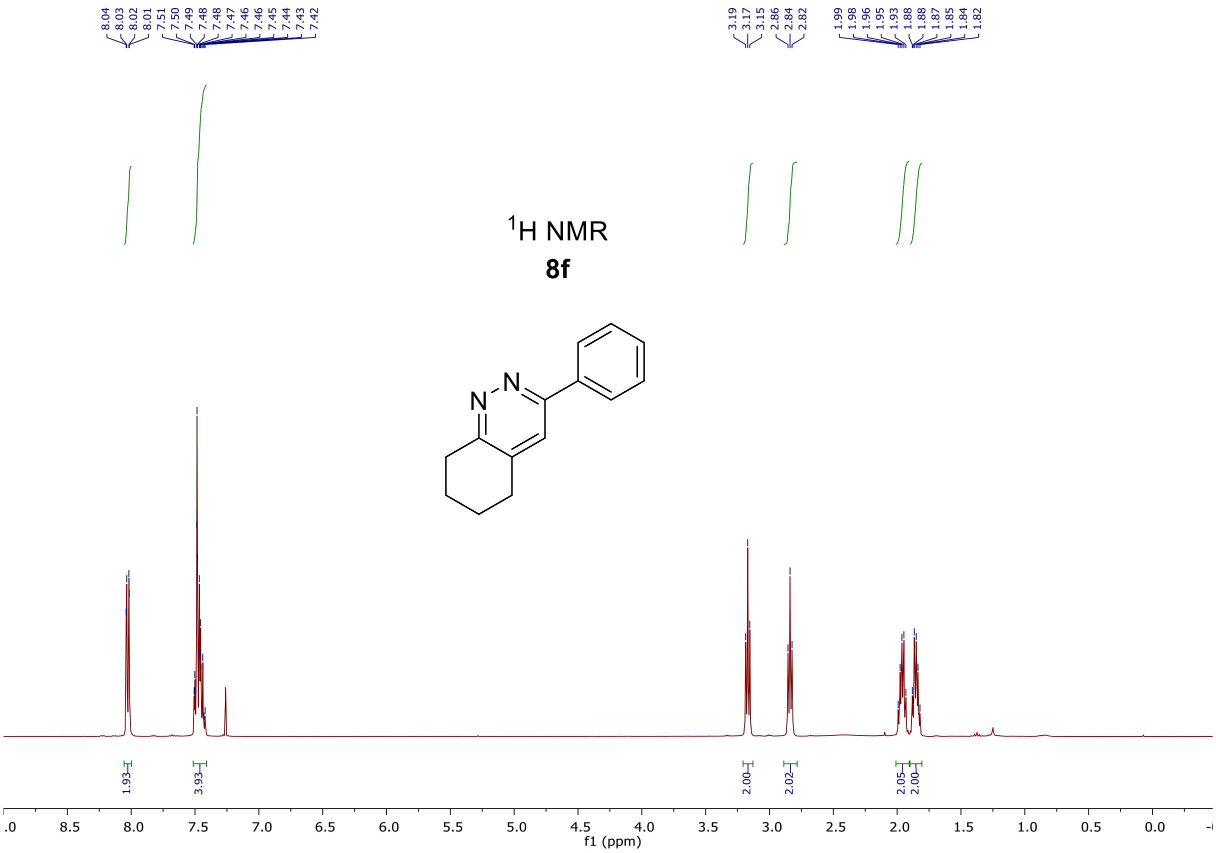

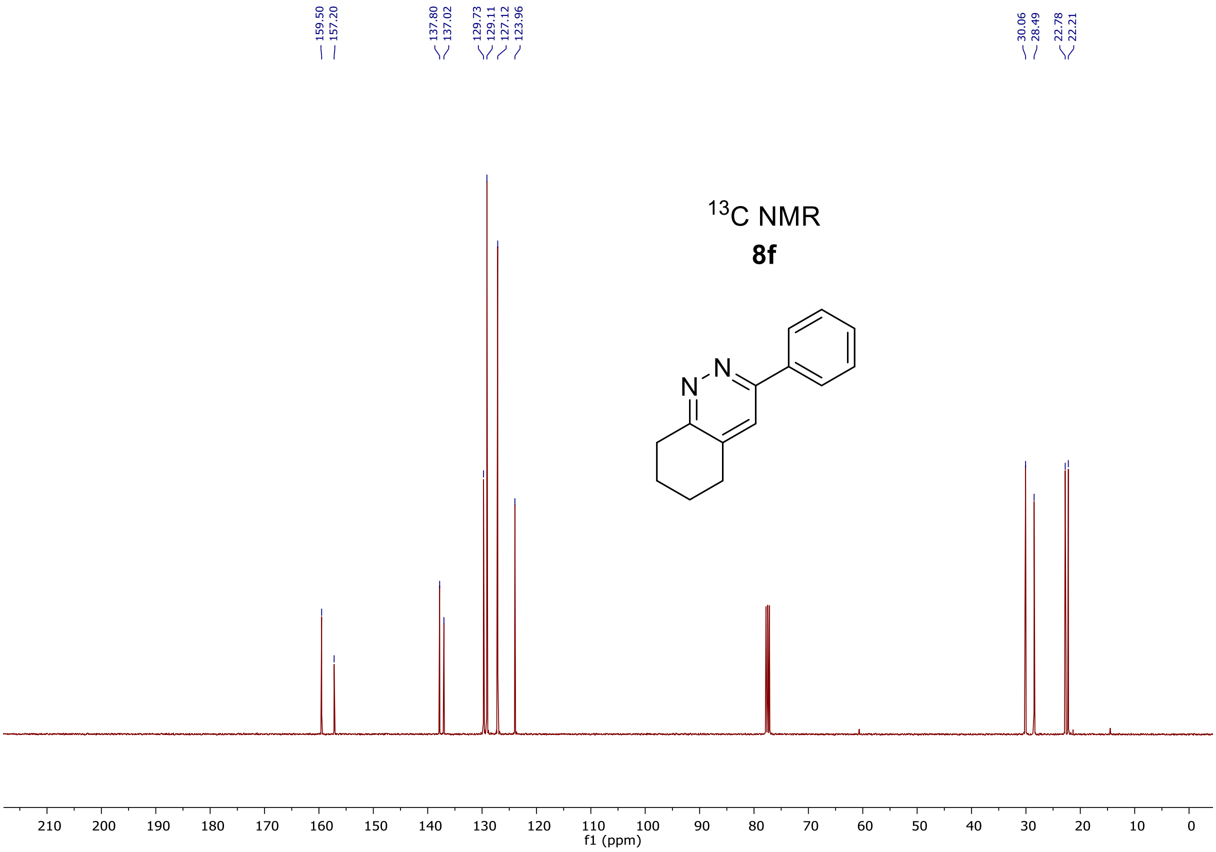

- ***3-([1,1'-Biphenyl]-4-yl)-5,6,7,8-tetrahydrocinnoline (8g)***

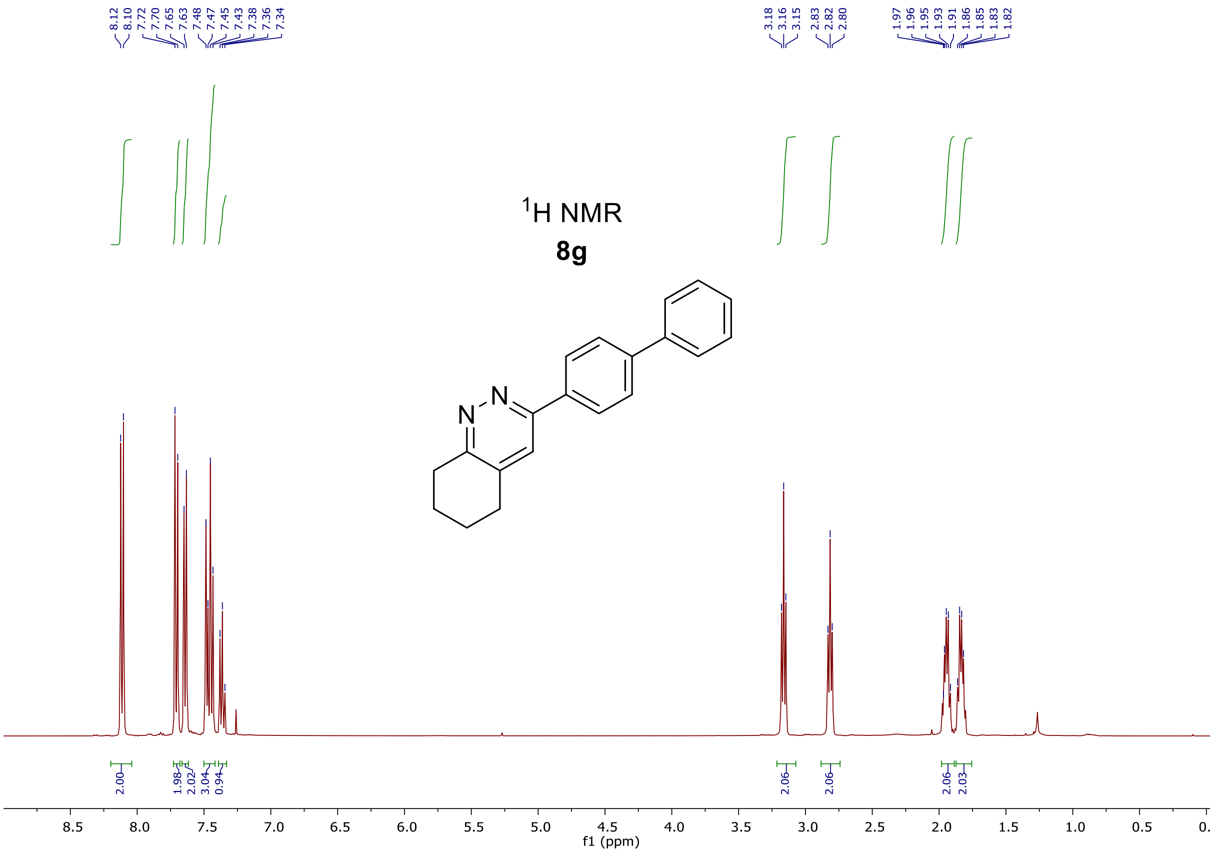

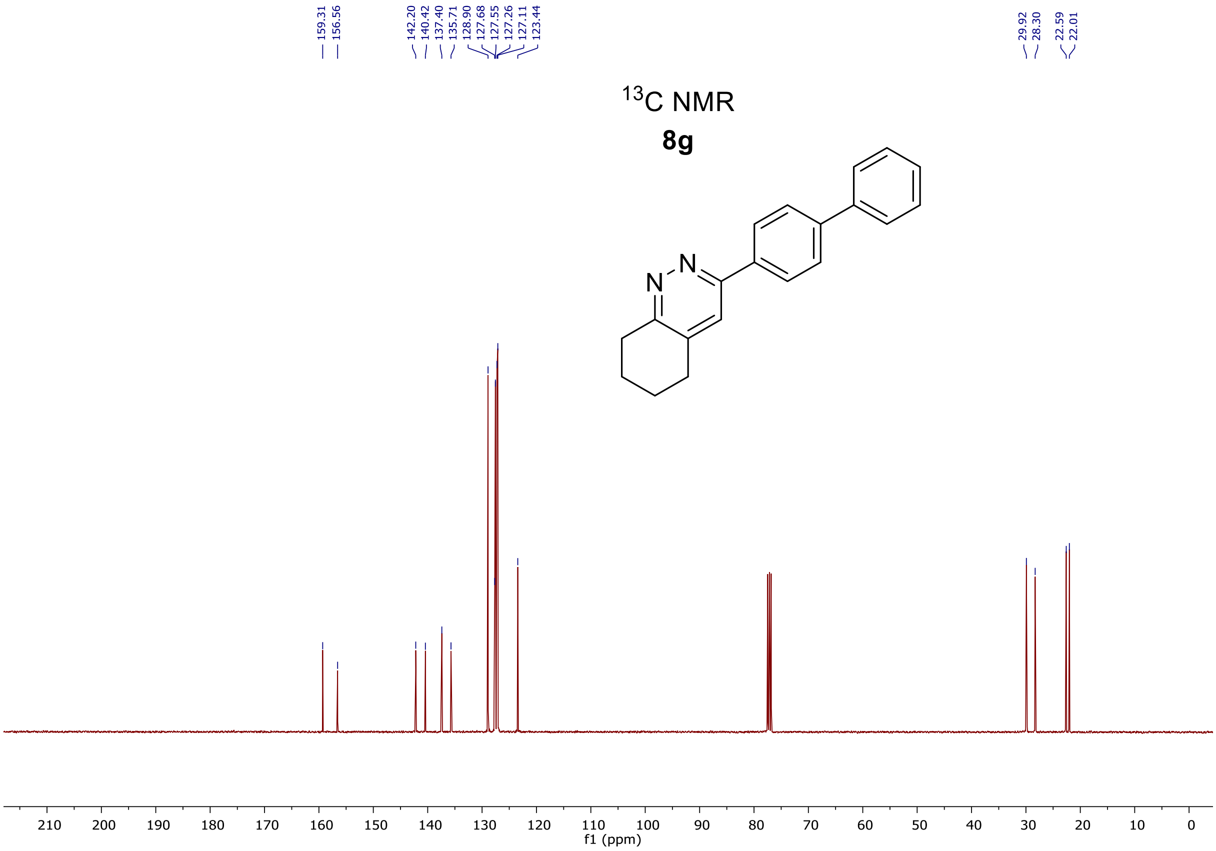

- ***3-(4-Iodophenyl)-5,6,7,8-tetrahydrocinnoline (8h)***

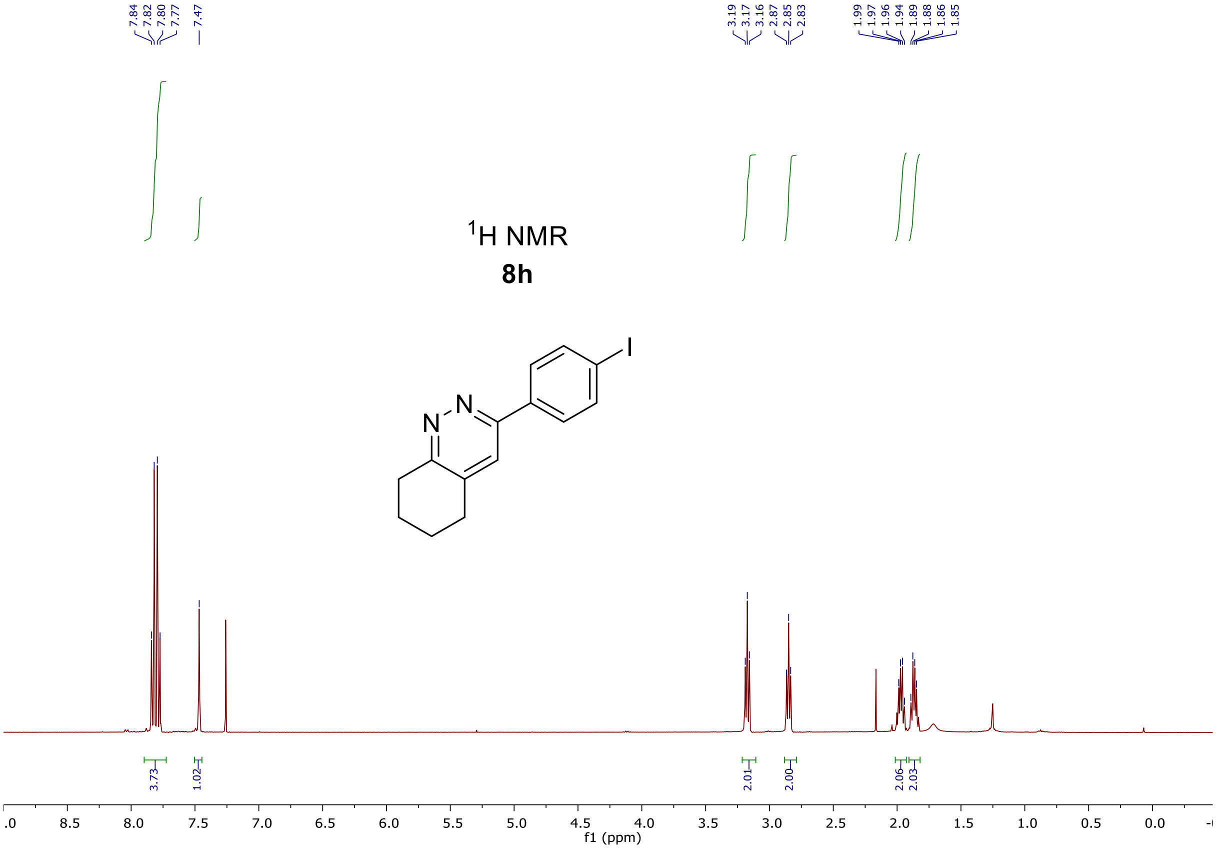

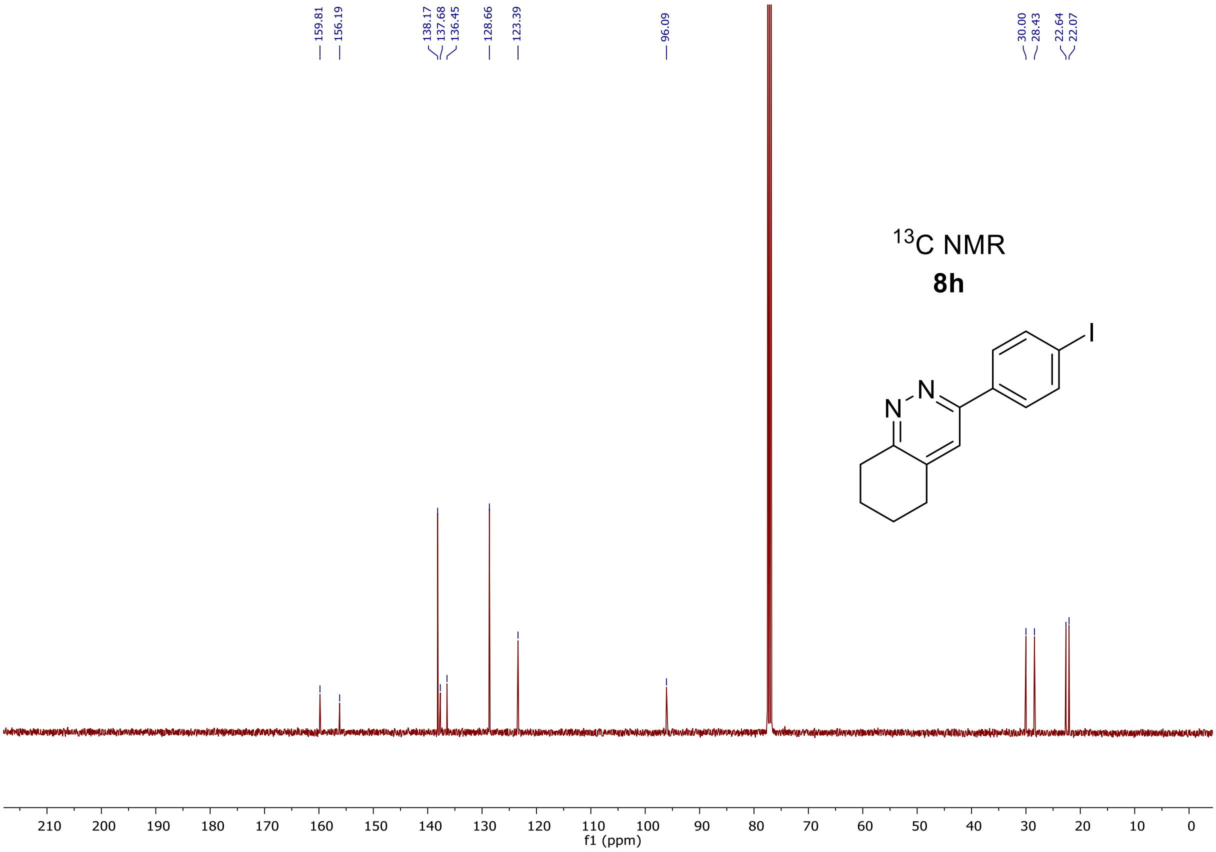

- ***3-(4-Fluorophenyl)-5,6,7,8-tetrahydrocinnoline (8i)***

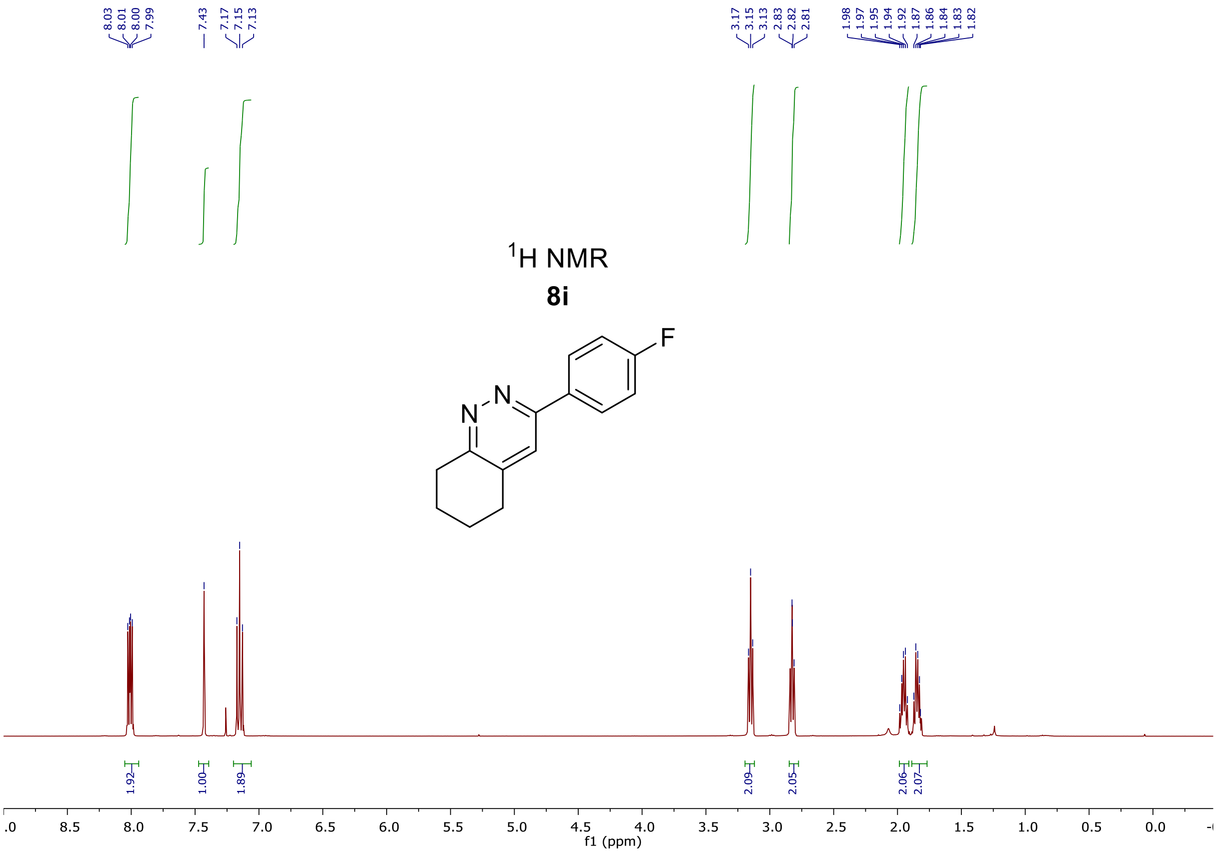

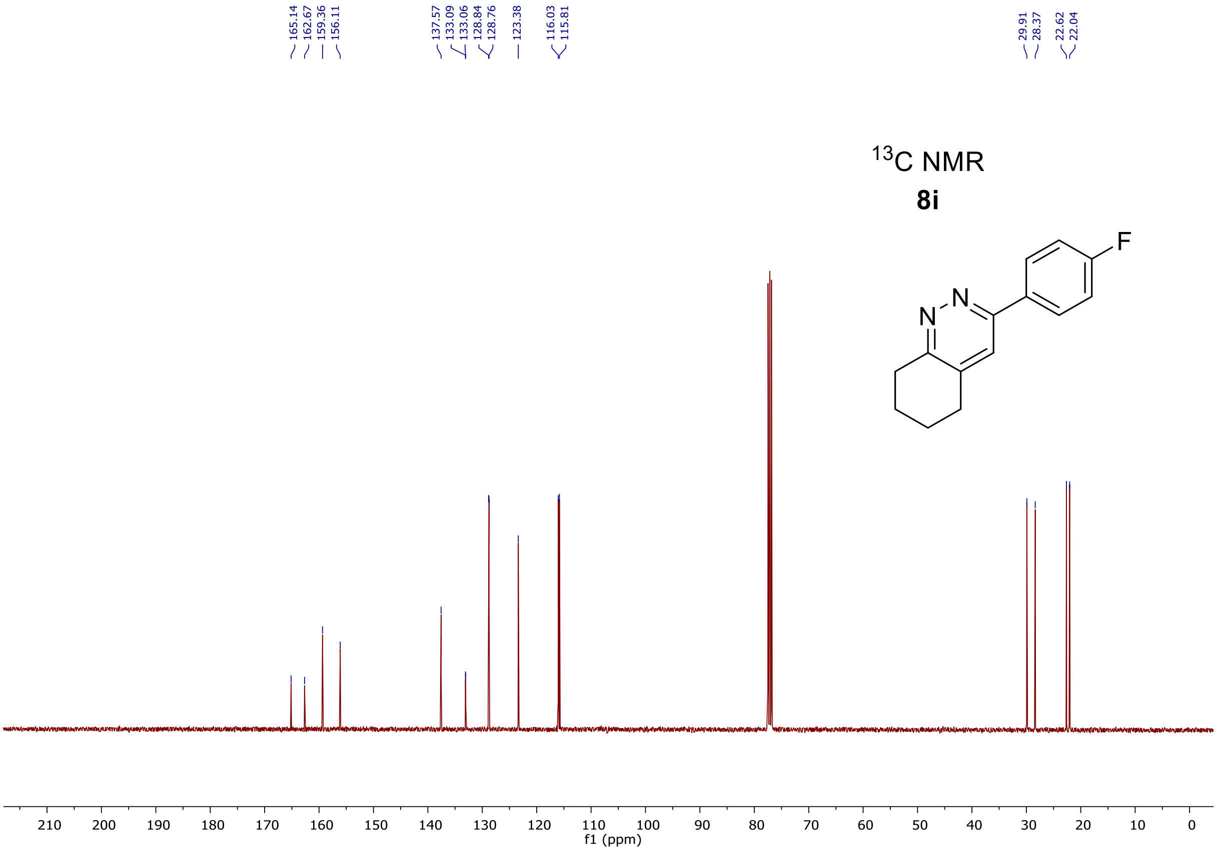

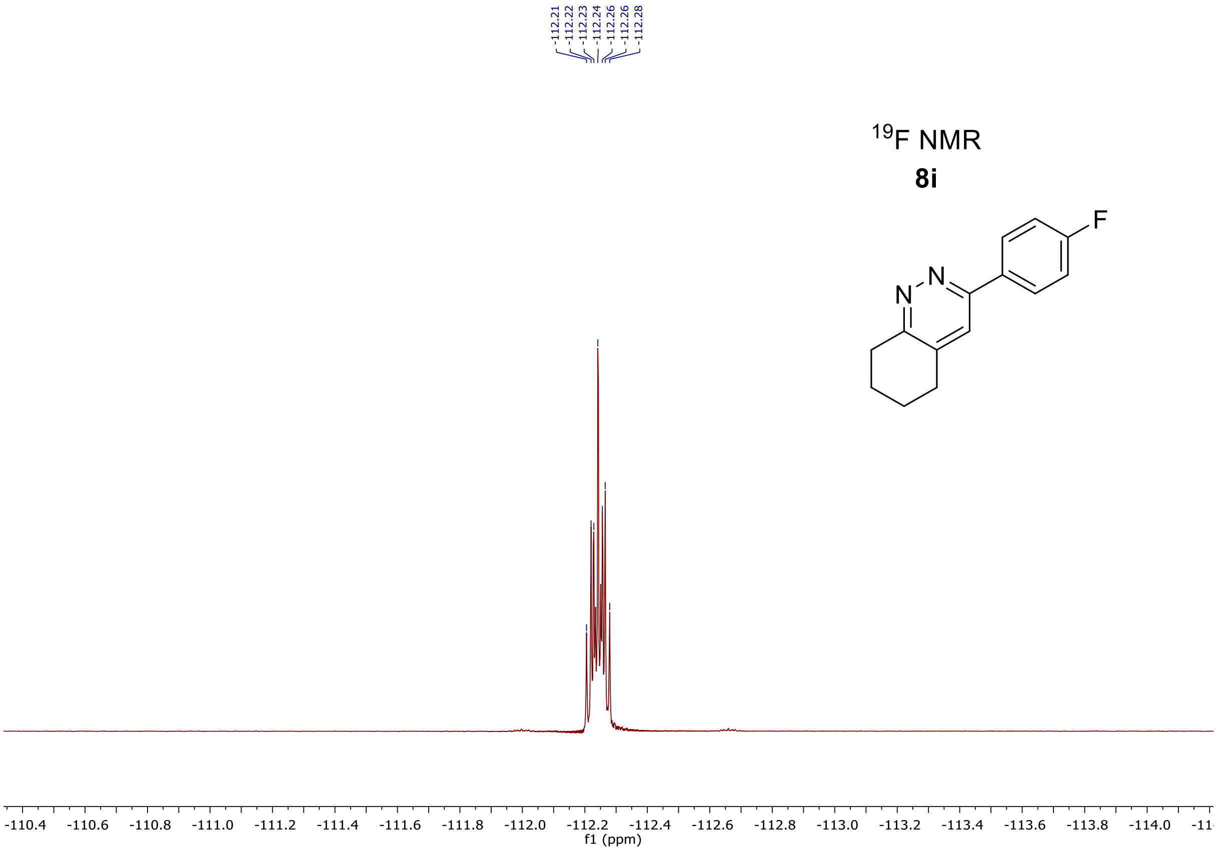
-112.21
-112.22
-112.23
-112.24
-112.26
-112.26
-112.28
^{19}F NMR
8i
F
N
N
-110.4 -110.6 -110.8 -111.0 -111.2 -111.4 -111.6 -111.8 -112.0 -112.2 -112.4 -112.6 -112.8 -113.0 -113.2 -113.4 -113.6 -113.8 -114.0
f1 (ppm)

- ***3-(Thiophen-2-yl)-5,6,7,8-tetrahydrocinnoline (8j)***

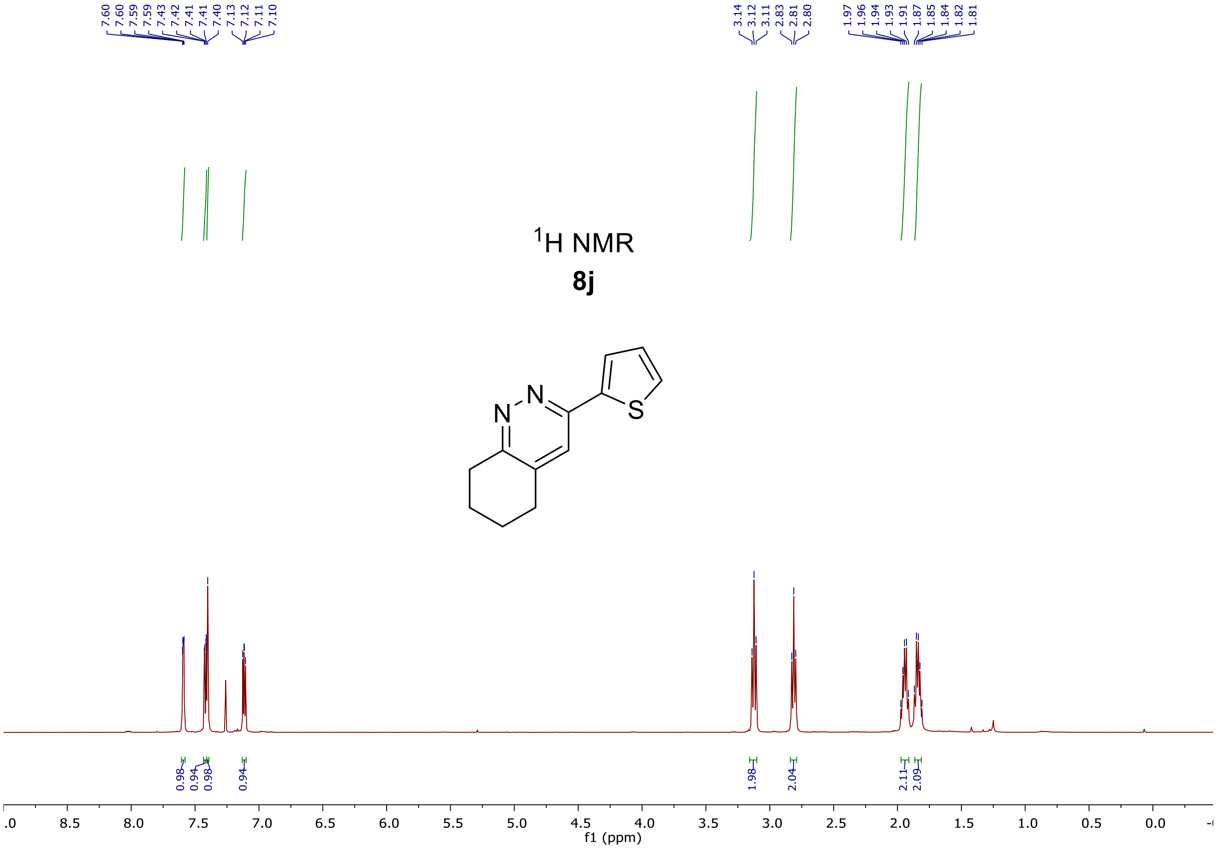

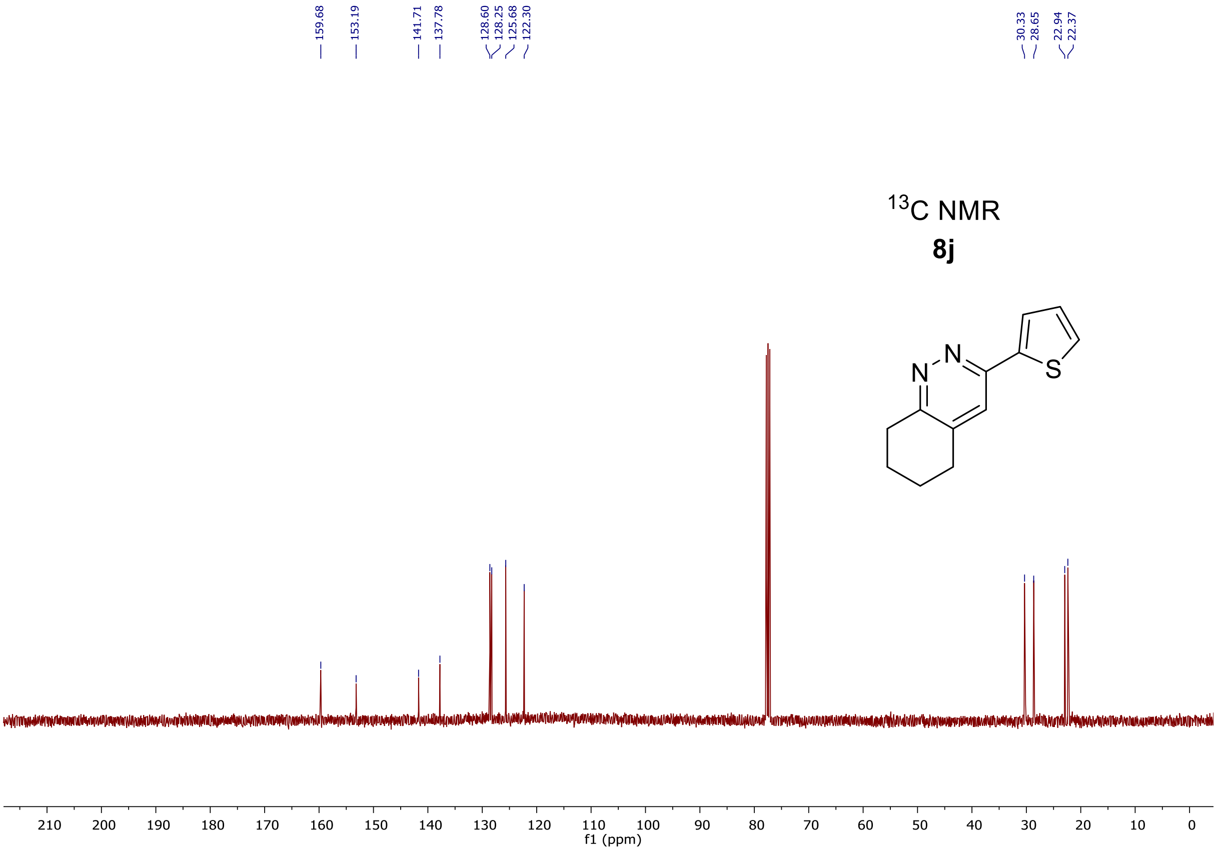

- ***3-(Pyridin-2-yl)-5,6,7,8-tetrahydrocinnoline (8k)***

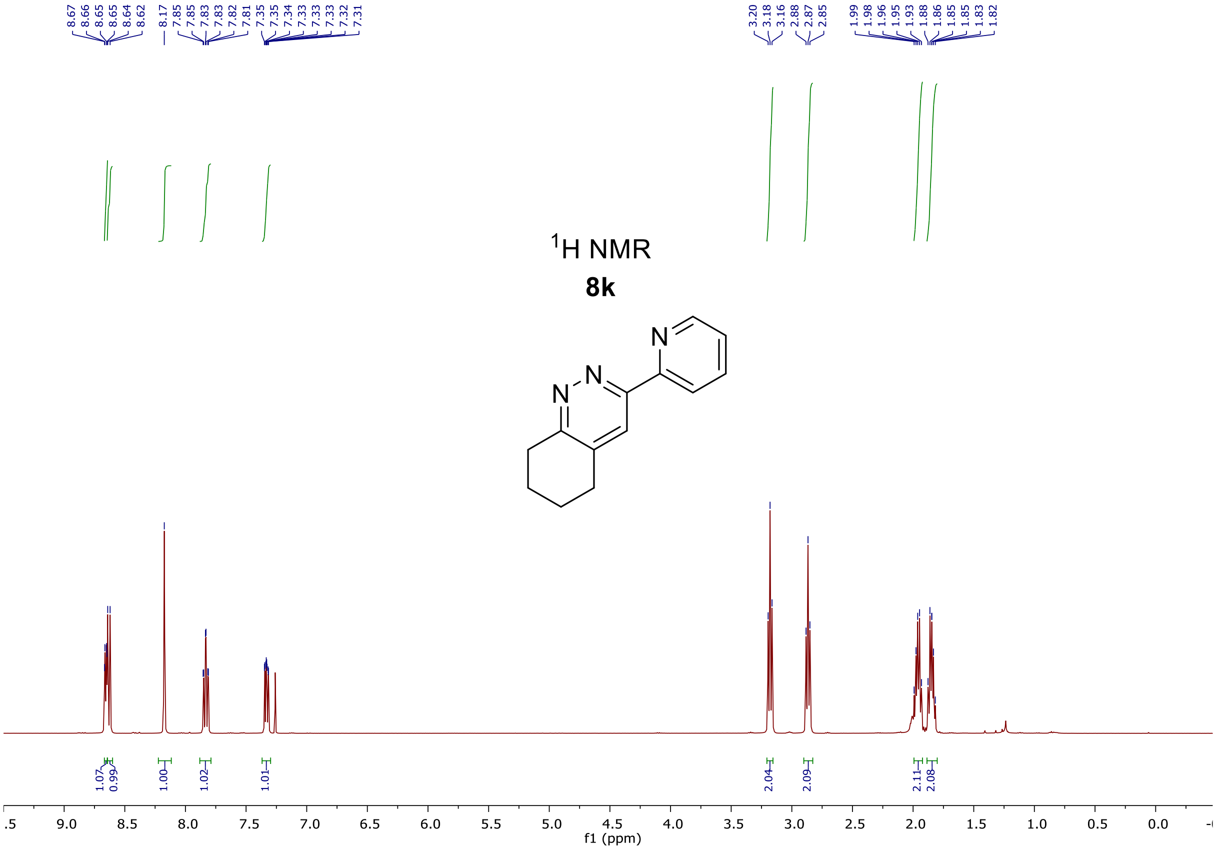

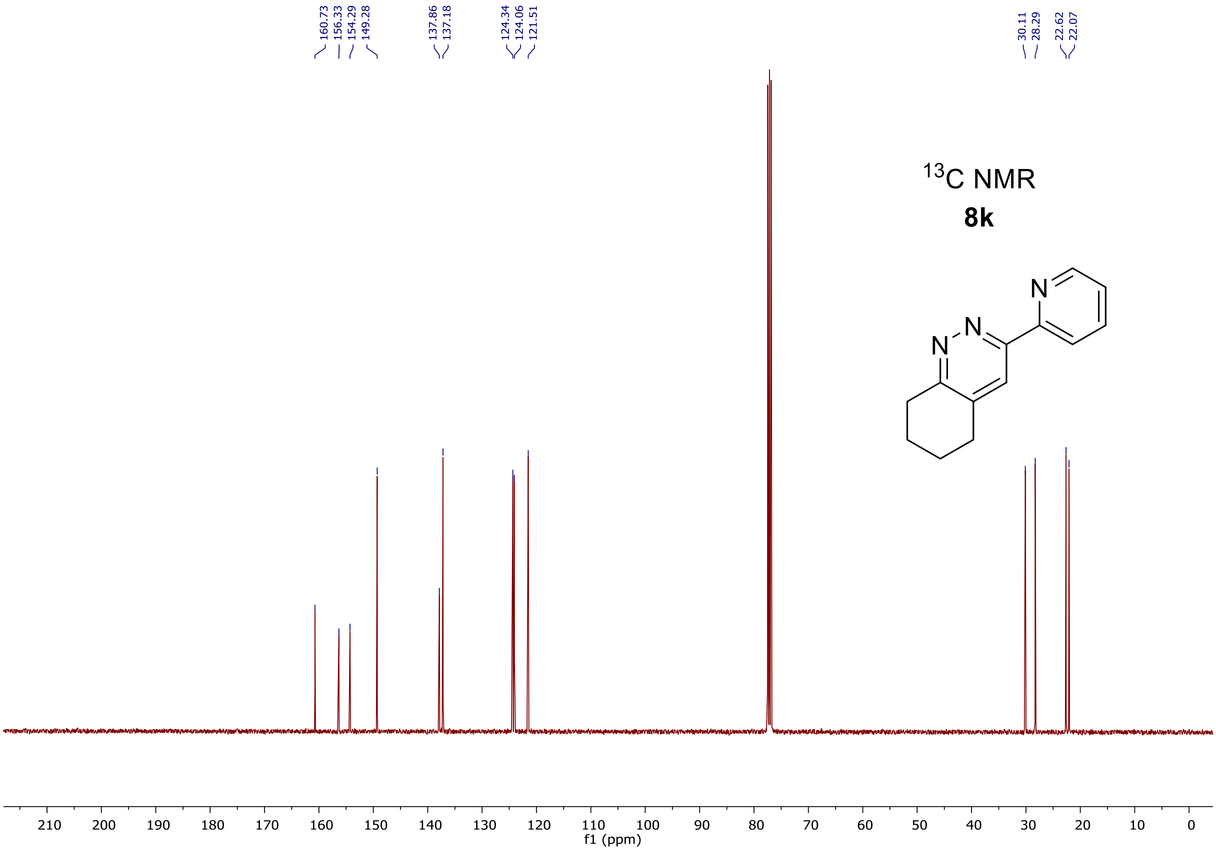

VI. References

(1) Rezgui, F.; El Gaied, M. M. *Tetrahedron Lett.* **1998**, *39*, 5965–5966.
(2) Rezgui, F.; El Gaïed, M. M. *Tetrahedron* **1997**, *53*, 15711–15716.
(3) Mhasni, O.; Rezgui, F. *Tetrahedron Lett.* **2010**, *51*, 586–587.
(4) Chamakh, A.; M'hirsi, M.; Villiéras, J.; Lebreton, J.; Amri, H. *Synthesis* **2000**, 295–299.
(5) Teske, J.; Plietker, B. *Org. Lett.* **2018**, *20* (8), 2257–2260.
(6) Mawaziny, S.; Lawny, A. M. *Phosphorus, Sulfur, Silicon Relat. Elem.* **2000**, *163*, 99–120.
(7) Porzelle, A.; Williams, C. M.; Schwartz, B. D.; Gentle, I. R. *Synlett* **2005**, *2005*, 2923–2926.
(8) Porzelle, A.; Williams, C. M. *Synthesis* **2006**, *2006*, 3025–3030.
(9) Belmessieri, D.; Cordes, D. B.; Slawin, A. M. Z.; Smith, A. D. *Org. Lett.* **2013**, *15*, 3472–3475.
(10) Aitken, R. A.; Atherton, J. I. *J. Chem. Soc., Perkin Trans. 1* **1994**, No. 10, 1281–1284.
(11) Yang, X.-Y.; Tay, W. S.; Li, Y.; Pullarkat, S. A.; Leung, P.-H. *Organometallics* **2015**, *34*, 5196–5201.
(12) Yuvaraj, T. V.; Unnissa, S. H.; Surendiran, N. S.; Ur rahman, M. A.; Binumon, V. *AJRC* **2010**, *3*, 853–858
(13) Roman, G.; Vlahakis, J. Z.; Vukomanovic, D.; Nakatsu, K.; Szarek, W. A. *ChemMedChem* **2010**, *5*, 1541–1555.
(14) Karami, K. *J. Chem. Res.* **2007**, *2007*, 725–727.
(15) Aeluri, R.; Alla, M.; Polepalli, S.; Jain, N. *Eur. J. Med. Chem.* **2015**, *100*, 18–23.
(16) Guan, X.-Y.; Al-Misba'a, Z.; Huang, K.-W. *Arab. J. Chem.* **2015**, *8*, 892–896.
(17) Wang, Z.-H.; Wu, Z.-J.; Huang, X.-Q.; Yue, D.-F.; You, Y.; Xu, X.-Y.; Zhang, X.-M.; Yuan, W.-C. *Chem. Commun.* **2015**, *51*, 15835–15838.
(18) Hay, M. P.; Turcotte, S.; Flanagan, J. U.; Bonnet, M.; Chan, D. A.; Sutphin, P. D.; Nguyen, P.; Giaccia, A. J.; Denny, W. A. *J. Med. Chem.* **2010**, *53*, 787–797.
(19) Zhu, L.; Zhou, C.; Yang, W.; He, S.; Cheng, G.-J.; Zhang, X.; Lee, C.-S. *J. Org. Chem.* **2013**, *78*, 7912–7929.
(20) Kay, I. T.; Glue, S. E. J. *Tetrahedron Letters* **1986**, *27*, 113–116.
(21) Mandal, T.; Chakraborti, G.; Karmakar, S.; Dash, J. *Org. Lett.* **2018**, *20*, 4759–4763.
(22) Prabhakaran, E. N.; Nugent, B. M.; Williams, A. L.; Nailor, K. E.; Johnston, J. N. *Org. Lett.* **2002**, *4*, 4197–4200.
(23) Bell, M. R.; D'Ambra, T. E.; Kumar, V.; Eissenstat, M. A.; Herrmann, J. L.; Wetzel, J. R.; Rosi, D.; Philion, R. E.; Daum, S. J. *J. Med. Chem.* **1991**, *34*, 1099–1110.
(24) Belll, M. R. 3-Carbonyl-1-Aminoalkyl-1H-Indoles Useful as Analgesics. US patent 637931, 1984.
(25) Wang, Y.; Bi, X.; Li, D.; Liao, P.; Wang, Y.; Yang, J.; Zhang, Q.; Liu, Q. *Chem. Commun.* **2011**, *47*, 809–811.
(26) Li, W.; Usman, M.; Wu, L.-Y.; Liu, W.-B. *J. Org. Chem.* **2019**, *84*, 15754–15763.
(27) Chang, M.-Y.; Lu, Y.-J.; Cheng, Y.-C. *Tetrahedron* **2015**, *71*, 6840–6845.
(28) South, M. S.; Jakuboski, T. L.; Westmeyer, M. D.; Dukesherer, D. R. *J. Org. Chem.* **1996**, *61*, 8921–8934.
